# Estimates of global, regional, and national incidence, prevalence, and mortality of HIV, 1980–2015: the Global Burden of Disease Study 2015

**DOI:** 10.1016/S2352-3018(16)30087-X

**Published:** 2016-07-19

**Authors:** GBD 2015 HIV Collaborators, Haidong Wang, Tim M Wolock, Austin Carter, Grant Nguyen, Hmwe Hmwe Kyu, Emmanuela Gakidou, Simon I Hay, Edward J Mills, Adam Trickey, William Msemburi, Matthew M Coates, Meghan D Mooney, Maya S Fraser, Amber Sligar, Joshua Salomon, Heidi J Larson, Joseph Friedman, Amanuel Alemu Abajobir, Kalkidan Hassen Abate, Kaja M Abbas, Mohamed Magdy Abd El Razek, Foad Abd-Allah, Abdishakur M Abdulle, Semaw Ferede Abera, Ibrahim Abubakar, Laith J Abu-Raddad, Niveen M E Abu-Rmeileh, Gebre Yitayih Abyu, Akindele Olupelumi Adebiyi, Isaac Akinkunmi Adedeji, Ademola Lukman Adelekan, Koranteng Adofo, Arsène Kouablan Adou, Oluremi N Ajala, Tomi F Akinyemiju, Nadia Akseer, Faris Hasan Al Lami, Ziyad Al-Aly, Khurshid Alam, Noore K M Alam, Deena Alasfoor, Saleh Fahed S Aldhahri, Robert William Aldridge, Miguel Angel Alegretti, Alicia V Aleman, Zewdie Aderaw Alemu, Rafael Alfonso-Cristancho, Raghib Ali, Ala'a Alkerwi, François Alla, Rajaa Mohammad, Salem Al-Raddadi, Ubai Alsharif, Elena Alvarez, Nelson Alvis-Guzman, Azmeraw T Amare, Alemayehu Amberbir, Adeladza Kofi Amegah, Walid Ammar, Stephen Marc Amrock, Carl Abelardo T Antonio, Palwasha Anwari, Johan Ärnlöv, Al Artaman, Hamid Asayesh, Rana Jawad Asghar, Reza Assadi, Suleman Atique, Lydia S Atkins, Euripide Frinel G Arthur Avokpaho, Ashish Awasthi, Beatriz Paulina Ayala Quintanilla, Umar Bacha, Alaa Badawi, Aleksandra Barac, Till Bärnighausen, Arindam Basu, Tigist Assefa Bayou, Yibeltal Tebekaw Bayou, Shahrzad Bazargan-Hejazi, Justin Beardsley, Neeraj Bedi, Derrick A Bennett, Isabela M Bensenor, Balem Demtsu Betsu, Addisu Shunu Beyene, Eesh Bhatia, Zulfiqar A Bhutta, Sibhatu Biadgilign, Boris Bikbov, Sait Mentes Birlik, Donal Bisanzio, Michael Brainin, Alexandra Brazinova, Nicholas J K Breitborde, Alexandria Brown, Michael Burch, Zahid A Butt, Julio Cesar Campuzano, Rosario Cárdenas, Juan Jesus Carrero, Carlos A Castañeda-Orjuela, Jacqueline Castillo Rivas, Ferrán Catalá-López, Hsing-Yi Chang, Jung-chen Chang, Laxmikant Chavan, Wanqing Chen, Peggy Pei-Chia Chiang, Mirriam Chibalabala, Vesper Hichilombwe Chisumpa, Jee-Young Jasmine Choi, Devasahayam Jesudas Christopher, Liliana G Ciobanu, Cyrus Cooper, Tukur Dahiru, Solomon Abrha Damtew, Lalit Dandona, Rakhi Dandona, José das Neves, Pieter de Jager, Diego De Leo, Louisa Degenhardt, Robert P Dellavalle, Kebede Deribe, Amare Deribew, Don C Des Jarlais, Samath D Dharmaratne, Eric L Ding, Pratik Pinal Doshi, Kerrie E Doyle, Tim R Driscoll, Manisha Dubey, Yousef Mohamed Elshrek, Iqbal Elyazar, Aman Yesuf Endries, Sergey Petrovich Ermakov, Babak Eshrati, Alireza Esteghamati, Imad D A Faghmous, Carla Sofia e Sa Farinha, Andre Faro, Maryam S Farvid, Farshad Farzadfar, Seyed-Mohammad Fereshtehnejad, Joao C Fernandes, Florian Fischer, Joseph Robert Anderson Fitchett, Nataliya Foigt, Nancy Fullman, Thomas Fürst, Fortuné Gbètoho Gankpé, Teshome Gebre, Amanuel Tesfay Gebremedhin, Alemseged Aregay Gebru, Johanna M Geleijnse, Bradford D Gessner, Peter W Gething, Tsegaye Tewelde Ghiwot, Maurice Giroud, Melkamu Dedefo Gishu, Elizabeth Glaser, Shifalika Goenka, Amador Goodridge, Sameer Vali Gopalani, Atsushi Goto, Harish Chander Gugnani, Mark D C Guimaraes, Rahul Gupta, Rajeev Gupta, Vipin Gupta, Juanita Haagsma, Nima Hafezi-Nejad, Holly Hagan, Gessessew Bugssa Hailu, Randah Ribhi Hamadeh, Samer Hamidi, Mouhanad Hammami, Graeme J Hankey, Yuantao Hao, Hilda L Harb, Sivadasanpillai Harikrishnan, Josep Maria Haro, Kimani M Harun, Rasmus Havmoeller, Mohammad T Hedayati, Ileana Beatriz Heredia-Pi, Hans W Hoek, Masako Horino, Nobuyuki Horita, H Dean Hosgood, Damian G Hoy, Mohamed Hsairi, Guoqing Hu, Hsiang Huang, John J Huang, Kim Moesgaard Iburg, Bulat T Idrisov, Kaire Innos, Veena J Iyer, Kathryn H Jacobsen, Nader Jahanmehr, Mihajlo B Jakovljevic, Mehdi Javanbakht, Achala Upendra Jayatilleke, Panniyammakal Jeemon, Vivekanand Jha, Guohong Jiang, Ying Jiang, Tariku Jibat, Jost B Jonas, Zubair Kabir, Ritul Kamal, Haidong Kan, André Karch, Corine Kakizi Karema, Dimitris Karletsos, Amir Kasaeian, Anil Kaul, Norito Kawakami, Jeanne Françoise Kayibanda, Peter Njenga Keiyoro, Andrew Haddon Kemp, Andre Pascal Kengne, Chandrasekharan Nair Kesavachandran, Yousef Saleh Khader, Ibrahim Khalil, Abdur Rahman Khan, Ejaz Ahmad Khan, Young-Ho Khang, Jagdish Khubchandani, Yun Jin Kim, Yohannes Kinfu, Miia Kivipelto, Yoshihiro Kokubo, Soewarta Kosen, Parvaiz A Koul, Ai Koyanagi, Barthelemy Kuate Defo, Burcu Kucuk Bicer, Veena S Kulkarni, G Anil Kumar, Dharmesh Kumar Lal, Hilton Lam, Jennifer O Lam, Sinead M Langan, Van C Lansingh, Anders Larsson, James Leigh, Ricky Leung, Yongmei Li, Stephen S Lim, Steven E Lipshultz, Shiwei Liu, Belinda K Lloyd, Giancarlo Logroscino, Paulo A Lotufo, Raimundas Lunevicius, Hassan Magdy Abd El Razek, Mahdi Mahdavi, P A Mahesh, Marek Majdan, Azeem Majeed, Carla Makhlouf, Reza Malekzadeh, Chabila C Mapoma, Wagner Marcenes, Jose Martinez-Raga, Melvin Barrientos Marzan, Felix Masiye, Amanda J Mason-Jones, Bongani M Mayosi, Martin McKee, Peter A Meaney, Man Mohan Mehndiratta, Alemayehu B Mekonnen, Yohannes Adama Melaku, Peter Memiah, Ziad A Memish, Walter Mendoza, Atte Meretoja, Tuomo J Meretoja, Francis Apolinary Mhimbira, Ted R Miller, Joseph Mikesell, Mojde Mirarefin, Karzan Abdulmuhsin Mohammad, Shafiu Mohammed, Ali H Mokdad, Lorenzo Monasta, Maziar Moradi-Lakeh, Rintaro Mori, Ulrich O Mueller, Brighton Murimira, Gudlavalleti Venkata Satyanarayana Murthy, Aliya Naheed, Luigi Naldi, Vinay Nangia, Denis Nash, Haseeb Nawaz, Chakib Nejjari, Frida Namnyak Ngalesoni, Jean de Dieu Ngirabega, Quyen Le Nguyen, Muhammad Imran Nisar, Ole F Norheim, Rosana E Norman, Luke Nyakarahuka, Felix Akpojene Ogbo, In-Hwan Oh, Foluke Adetola Ojelabi, Bolajoko Olubukunola Olusanya, Jacob Olusegun Olusanya, John Nelson Opio, Eyal Oren, Erika Ota, Hye-Youn Park, Jae-Hyun Park, Snehal T Patil, Scott B Patten, Vinod K Paul, Katherine Pearson, Emmanuel Kwame Peprah, David M Pereira, Norberto Perico, Konrad Pesudovs, Max Petzold, Michael Robert Phillips, Julian David Pillay, Dietrich Plass, Suzanne Polinder, Farshad Pourmalek, David M Prokop, Mostafa Qorbani, Anwar Rafay, Kazem Rahimi, Vafa Rahimi-Movaghar, Mahfuzar Rahman, Mohammad Hifz Ur Rahman, Sajjad Ur Rahman, Rajesh Kumar Rai, Sasa Rajsic, Usha Ram, Saleem M Rana, Paturi Vishnupriya Rao, Giuseppe Remuzzi, David Rojas-Rueda, Luca Ronfani, Gholamreza Roshandel, Ambuj Roy, George Mugambage Ruhago, Mohammad Yahya Saeedi, Rajesh Sagar, Muhammad Muhammad Saleh, Juan R Sanabria, Itamar S Santos, Rodrigo Sarmiento-Suarez, Benn Sartorius, Monika Sawhney, Aletta E Schutte, David C Schwebel, Soraya Seedat, Sadaf G Sepanlou, Edson E Servan-Mori, Masood Ali Shaikh, Rajesh Sharma, Jun She, Sara Sheikhbahaei, Jiabin Shen, Kenji Shibuya, Hwashin Hyun Shin, Inga Dora Sigfusdottir, Naris Silpakit, Diego Augusto Santos Silva, Dayane Gabriele Alves Silveira, Edgar P Simard, Shireen Sindi, Jasvinder A Singh, Om Prakash Singh, Prashant Kumar Singh, Vegard Skirbekk, Karen Sliwa, Samir Soneji, Reed J D Sorensen, Joan B Soriano, David O Soti, Chandrashekhar T Sreeramareddy, Vasiliki Stathopoulou, Nicholas Steel, Bruno F Sunguya, Soumya Swaminathan, Bryan L Sykes, Rafael Tabarés-Seisdedos, Roberto Tchio Talongwa, Mohammad Tavakkoli, Bineyam Taye, Bemnet Amare Tedla, Tesfaye Tekle, Girma Temam Shifa, Awoke Misganaw Temesgen, Abdullah Sulieman Terkawi, Fisaha Haile Tesfay, Gizachew Assefa Tessema, Kiran Thapa, Alan J Thomson, Andrew L Thorne-Lyman, Ruoyan Tobe-Gai, Roman Topor-Madry, Jeffrey Allen Towbin, Bach Xuan Tran, Zacharie Tsala Dimbuene, Nikolaos Tsilimparis, Abera Kenay Tura, Kingsley Nnanna Ukwaja, Chigozie Jesse Uneke, Olalekan A Uthman, N Venketasubramanian, Sergey K Vladimirov, Vasiliy Victorovich Vlassov, Stein Emil Vollset, Linhong Wang, Elisabete Weiderpass, Robert G Weintraub, Andrea Werdecker, Ronny Westerman, Tissa Wijeratne, James D Wilkinson, Charles Shey Wiysonge, Charles D A Wolfe, Sungho Won, John Q Wong, Gelin Xu, Ajit Kumar Yadav, Bereket Yakob, Ayalnesh Zemene Yalew, Yuichiro Yano, Mehdi Yaseri, Henock Gebremedhin Yebyo, Paul Yip, Naohiro Yonemoto, Seok-Jun Yoon, Mustafa Z Younis, Chuanhua Yu, Shicheng Yu, Zoubida Zaidi, Maysaa El Sayed Zaki, Hajo Zeeb, Hao Zhang, Yong Zhao, Sanjay Zodpey, Leo Zoeckler, Liesl Joanna Zuhlke, Alan D Lopez, Christopher J L Murray

## Abstract

**Background:**

Timely assessment of the burden of HIV/AIDS is essential for policy setting and programme evaluation. In this report from the Global Burden of Disease Study 2015 (GBD 2015), we provide national estimates of levels and trends of HIV/AIDS incidence, prevalence, coverage of antiretroviral therapy (ART), and mortality for 195 countries and territories from 1980 to 2015.

**Methods:**

For countries without high-quality vital registration data, we estimated prevalence and incidence with data from antenatal care clinics and population-based seroprevalence surveys, and with assumptions by age and sex on initial CD4 distribution at infection, CD4 progression rates (probability of progression from higher to lower CD4 cell-count category), on and off antiretroviral therapy (ART) mortality, and mortality from all other causes. Our estimation strategy links the GBD 2015 assessment of all-cause mortality and estimation of incidence and prevalence so that for each draw from the uncertainty distribution all assumptions used in each step are internally consistent. We estimated incidence, prevalence, and death with GBD versions of the Estimation and Projection Package (EPP) and Spectrum software originally developed by the Joint United Nations Programme on HIV/AIDS (UNAIDS). We used an open-source version of EPP and recoded Spectrum for speed, and used updated assumptions from systematic reviews of the literature and GBD demographic data. For countries with high-quality vital registration data, we developed the cohort incidence bias adjustment model to estimate HIV incidence and prevalence largely from the number of deaths caused by HIV recorded in cause-of-death statistics. We corrected these statistics for garbage coding and HIV misclassification.

**Findings:**

Global HIV incidence reached its peak in 1997, at 3·3 million new infections (95% uncertainty interval [UI] 3·1–3·4 million). Annual incidence has stayed relatively constant at about 2·6 million per year (range 2·5–2·8 million) since 2005, after a period of fast decline between 1997 and 2005. The number of people living with HIV/AIDS has been steadily increasing and reached 38·8 million (95% UI 37·6–40·4 million) in 2015. At the same time, HIV/AIDS mortality has been declining at a steady pace, from a peak of 1·8 million deaths (95% UI 1·7–1·9 million) in 2005, to 1·2 million deaths (1·1–1·3 million) in 2015. We recorded substantial heterogeneity in the levels and trends of HIV/AIDS across countries. Although many countries have experienced decreases in HIV/AIDS mortality and in annual new infections, other countries have had slowdowns or increases in rates of change in annual new infections.

**Interpretation:**

Scale-up of ART and prevention of mother-to-child transmission has been one of the great successes of global health in the past two decades. However, in the past decade, progress in reducing new infections has been slow, development assistance for health devoted to HIV has stagnated, and resources for health in low-income countries have grown slowly. Achievement of the new ambitious goals for HIV enshrined in Sustainable Development Goal 3 and the 90-90-90 UNAIDS targets will be challenging, and will need continued efforts from governments and international agencies in the next 15 years to end AIDS by 2030.

**Funding:**

Bill & Melinda Gates Foundation, and National Institute of Mental Health and National Institute on Aging, National Institutes of Health.

## Introduction

HIV/AIDS is a leading cause of death and disease burden, especially in sub-Saharan Africa.^1^, ^2^, ^3^, ^4^, ^5^ Introduction of antiretroviral therapy (ART) in 1996 greatly reduced HIV-related mortality.^6^, ^7^ Creation of the Joint United Nations Programme on HIV/AIDS (UNAIDS) in 1996; the Global Fund to Fight AIDS, Tuberculosis and Malaria in 2002; and the US President's Emergency Plan for AIDS Relief (PEPFAR) in 2003, galvanised the mobilisation of resources to combat the HIV epidemic. In the past 15 years, the global community has provided US$109·8 billion of development assistance to curb the HIV/AIDS epidemic.^8^ As a result, HIV mortality has declined overall in low-income and middle-income countries since 2004.^1^

The success of ART and prevention of mother-to-child transmission programmes led to ambitious calls to eliminate HIV as a public health threat. However, maintenance and scale-up of sufficiently funded AIDS efforts will be crucial to realise the goal of ending the AIDS epidemic as a public health threat by 2030.^9^ Achievement of these goals, including the UNAIDS 90-90-90 targets, which aim to have 90% of people living with HIV know their status, 90% of those detected treated with ART, and 90% of those receiving treatment achieving viral load suppression,^10^ requires a coordinated global scale-up of prevention programmes, pre-exposure prophylaxis (PrEP), and detection and treatment programmes.^11^ However, development assistance for health targeted for HIV has stagnated since 2010, and, in many low-income countries, national resources for health are scarce and expected to grow slowly.^12^, ^13^

**Research in context**

**Evidence before this study**

We searched PubMed between Aug 18, 2015, and April 3, 2016, for studies that comprehensively assessed the burden of HIV/AIDS globally. Our search terms included “HIV” and “global” and “mortality” or “incidence” or “prevalence”, and searches were restricted to articles published in English up to April 1, 2016. To our knowledge through the search, Global Burden of Disease (GBD) and UNAIDS are the only two sources that provide comparable evaluations of levels and trends of the HIV/AIDS epidemic at both the global and country level. UNAIDS has provided global estimates on HIV/AIDS since 1997, and has developed two epidemiological programs to estimate incidence, prevalence, and mortality: Estimation and Projection Package (EPP) and Spectrum. GBD 2013 used improved versions of Spectrum to generate comprehensive, comparable estimates of levels and trends of HIV/AIDS incidence, prevalence, and mortality across geographies. Studies from both organisations have shown rapid changes in the HIV/AIDS epidemic worldwide and that up-to-date epidemiological and demographic information is needed to more accurately assess the burden of HIV at both the country and global level.

**Added value of this study**

For GBD 2015, we systematically updated the key inputs to our HIV/AIDS estimation process, which includes prevalence from national surveys and antenatal care clinics, demographic input on fertility and migration, mortality on and off antiretroviral therapy (ART), and background HIV-free mortality; updates to these inputs were concluded in April, 2016; October, 2015; December, 2015; and April, 2016, respectively. We also improved the integration of EPP, Spectrum, and the GBD all-cause mortality estimation process to make them internally consistent. For countries with high-quality vital registration data, we developed a new method to improve the accuracy of and consistency among estimates of HIV/AIDS incidence, prevalence, and mortality leveraging the number of deaths recorded each year as caused by HIV/AIDS. This method also allowed us to use vital registration data to generate plausible incidence curves in countries that are not part of UNAIDS' results, and in subnational units where we previously only had national-level data. We developed an ensemble model to reconcile HIV mortality estimates from EPP and Spectrum and from those indicated in GBD's all-cause mortality estimation process. Remarkable progress has been made in curbing the HIV/AIDS epidemic worldwide; however, our findings emphasise the need for continued efforts from governments and international agencies in the next 15 years to end AIDS by 2030, in view of the low ART coverage and stagnation in decline of annual new infections in the past decade.

**Implications of all available evidence**

Improving on existing models of HIV/AIDS burden estimates, this study provides the most comprehensive and internally consistent assessments of the levels and trends of HIV/AIDS incidence, prevalence, and mortality worldwide so far. This timely report provides much needed assessment of achievement of Millennium Development Goal 6, and lays out the challenges facing the global community in progress towards the HIV goals enshrined in Sustainable Development Goal 3 and the 90-90-90 UNAIDS targets.

The ambitious goals set forth by the global community, and the few resources available to combat HIV/AIDS, emphasise the importance of understanding and monitoring the trends of each country's HIV/AIDS epidemic. Measurement of disease burden according to geographic units enables comparison with other major conditions, showing where the epidemic remains a dominant cause of health loss and where the burden is still rising in spite of national and global efforts. Such measurement also enables direct comparison of different HIV/AIDS metrics, emphasising the specific needs of each geographic region and allowing for a more targeted response to the epidemic.

UNAIDS produces a biannual assessment of incidence of infections, prevalence of people living with HIV, and deaths from HIV/AIDS;^14^ the Global Burden of Disease Study (GBD) provides an alternative assessment of these rates. UNAIDS and GBD estimates have increasingly converged at the global level.^2^ Nevertheless, estimates differ substantially in several countries, particularly in middle-income and high-income countries, where GBD estimates are based on data from vital registration systems and UNAIDS estimates are based on prevalence in high-risk groups and estimates of the fraction of the population in these groups. This report from GBD 2015 provides a unique perspective on the national-level epidemiology of HIV/AIDS, which includes a comprehensive assessment of HIV/AIDS incidence, prevalence, and deaths.

## Methods

### Study design

GBD is a systematic, scientific effort to quantify all-cause mortality; cause-specific mortality; and disease incidence, prevalence, and burden attributable to risk factors by age, sex, and geography over time. GBD 2015 includes 195 countries and territories and covers the time span from 1980 to 2015. Additional details of the GBD cause hierarchy, data inputs and processing, and estimation methods have been published elsewhere.^15^

In brief, the GBD estimation framework for HIV/AIDS used the general natural history epidemiological models, Estimation and Projection Package (EPP) and Spectrum, developed by UNAIDS for estimation of the burden of HIV/AIDS for their biannual report on the state of the HIV/AIDS epidemic at the global and country levels.^1^ EPP uses HIV seroprevalence estimates from surveys and antenatal care clinics to estimate incidence curves that are consistent with the input data of prevalence and other factors, including on-ART and off-ART mortality and demographic information within the given population. Spectrum, a compartmental model, is used to generate age-specific and sex-specific incidence, prevalence, and mortality by use of the incidence curves generated in EPP and other key inputs, including program data on ART and prevention of mother-to-child transmission and other key assumptions of on-ART and off-ART mortality and HIV-free background mortality. Details of methods and parameters in EPP and Spectrum have been described previously.^16^, ^17^, ^18^, ^19^, ^20^, ^21^, ^22^, ^23^

In GBD 2015, we improved on UNAIDS' estimation procedures in four ways. First, we used additional data, both from vital registration systems and population health surveys, to measure seroprevalence. Second, we used consistent estimates of HIV-free mortality in both EPP and Spectrum, and in the estimation of on-ART and off-ART mortality—key inputs to both EPP and Spectrum. These HIV-free mortality rates, generated in GBD's all-cause mortality estimation process, have linked our HIV/AIDS estimation process and the all-cause mortality estimation process. Third, we developed an adjustment process—cohort incidence bias adjustment—to ensure that incidence and prevalence estimates formulated with Spectrum are consistent with HIV mortality estimates based on vital registration systems when available. Fourth, through an expanded literature search, we updated rates of on-ART mortality (appendix pp 6–10), particularly for developed countries, in close collaboration with the Antiretroviral Therapy Cohort Collaboration.^24^

Due to the interconnected nature of the HIV modelling process and the process of estimation of mortality and causes of death, data and codes for the GBD 2015 HIV estimation process will be made available along with all the GBD 2015 results, in compliance with the Guidelines for Accurate and Transparent Health Estimates Reporting (GATHER) developed by the WHO.^25^

### Mortality estimation

The GBD estimation framework contains three sources for estimates of HIV-specific mortality: estimated HIV mortality from Spectrum; estimated excess HIV/AIDS mortality in our all-cause mortality estimation process;^15^ and space–time Gaussian process regression smoothed cause-specific HIV/AIDS mortality from vital registration systems that were adjusted for incompleteness and misclassification of causes of death. We used tailored estimation methods to produce final estimates of mortality depending on age groups, and the availability and quality of data for mortality of HIV/AIDS.

We assigned countries and territories to one of four groups, depending on data availability and quality. Group 1 included countries with prevalence data from either household surveys or antenatal care clinics, most of which have generalised epidemics. Group 2A referred to countries with high-quality vital registration systems, which in GBD 2015 included countries with more than 25 years of vital registration data with more than 95% completeness. Group 2B referred to countries with vital registration systems that were not in group 2A. Group 2C included countries for which we had no data from a vital registration system. Briefly, for adults in group 1 countries, we applied an ensemble model to average HIV/AIDS mortality rates from Spectrum and those implied by the all-cause mortality estimation process. This approach was based on the fact that our estimation processes (appendix pp 12–15) in EPP, Spectrum, and all-cause mortality models were intrinsically linked by the same HIV-free mortality rates at the draw level for group 1 countries. Because EPP and Spectrum are largely based on prevalence estimates from surveys and antenatal care clinics and various assumptions, and all-cause mortality estimation process in group 1 countries are mostly based on sibling survival data with various biases that need to be corrected for, we used our ensemble model to give equal weights to HIV mortality estimates from the two processes.

For adults in group 2A countries, we used the results from space–time Gaussian process regression for age-specific HIV mortality. For adults in group 2B and 2C countries, we used the HIV-specific mortality rates from Spectrum with cohort incidence bias adjustment. For children younger than 5 years in group 1, we applied the proportion of all HIV deaths estimated within Spectrum to the age-specific all-cause mortality estimates. For children of this age in group 2A countries, we used space–time Gaussian process regression estimates of HIV mortality. For children aged 5–14 years from countries in group 1, we used the average of the HIV-specific mortality rates from Spectrum and the implied HIV mortality from the all-cause mortality process. For group 2A countries, we used estimates of HIV mortality from space–time Gaussian process regression. For groups 2B and 2C, we used the estimates of HIV-specific mortality from Spectrum.

### Incidence and prevalence estimation

We generated incidence and prevalence estimates with the recoded Spectrum model with updated assumptions of on-ART and off-ART mortality and other program data from the UNAIDS country files.

HIV cause-specific deaths from vital registration systems and sample registration systems are among the most reliable sources for estimation of the burden of HIV/AIDS. We used our cohort incidence bias adjustment method to scale the sizes of each incidence cohort on the basis of the raw estimates of HIV mortality from Spectrum, using unadjusted incidence curves and those observed in the vital registration system with proper incompleteness and cause misclassification adjustments.^15^ For this procedure, we first ran space–time Gaussian process regression on age-specific HIV/AIDS mortality rates after correcting for garbage codes, HIV misclassification, and under-registration by use of formal demographic methods to generate complete time-series estimates by location, sex, year, and age. We then restructured Spectrum by addition of another compartment such that it could follow groups of people living with HIV/AIDS who were infected in a specific year and age group. We then ran the modified program to produce 1000 draws of incidence, prevalence, and mortality for each location and sex combination. From this step, we were able to obtain the proportion of each infection cohort dying in each year and age cell after infection. We then used these proportions to weigh the ratio of the numbers of deaths based on the age-specific mortality rates from vital registration and processed by space–time Gaussian process regression, and the population estimated with Spectrum, and those directly from Spectrum. This process greatly improves both the model fit on mortality data, closer to what the adjusted vital registration suggests, and the incidence mortality ratio. Further details of the method are described in appendix pp 13–15.

### Uncertainty analysis

We systematically propagated uncertainty across EPP, Spectrum, and the all-cause mortality estimation processes. We used 1000 draws of the quantities of interest throughout all the steps in the estimation process. Some key inputs to the HIV estimation process did not include uncertainty: these were estimates of fertility and population, HIV programme metrics (including coverage of ART and prevention of mother-to-child transmission), and behavioural factors. We present results with 95% uncertainty intervals (UIs).

### Role of the funding source

The funder of the study had no role in study design, data collection, data analysis, data interpretation, or writing of the report. The corresponding author had full access to all the data in the study and had final responsibility for the decision to submit for publication.

## Results

**Figure 1:**
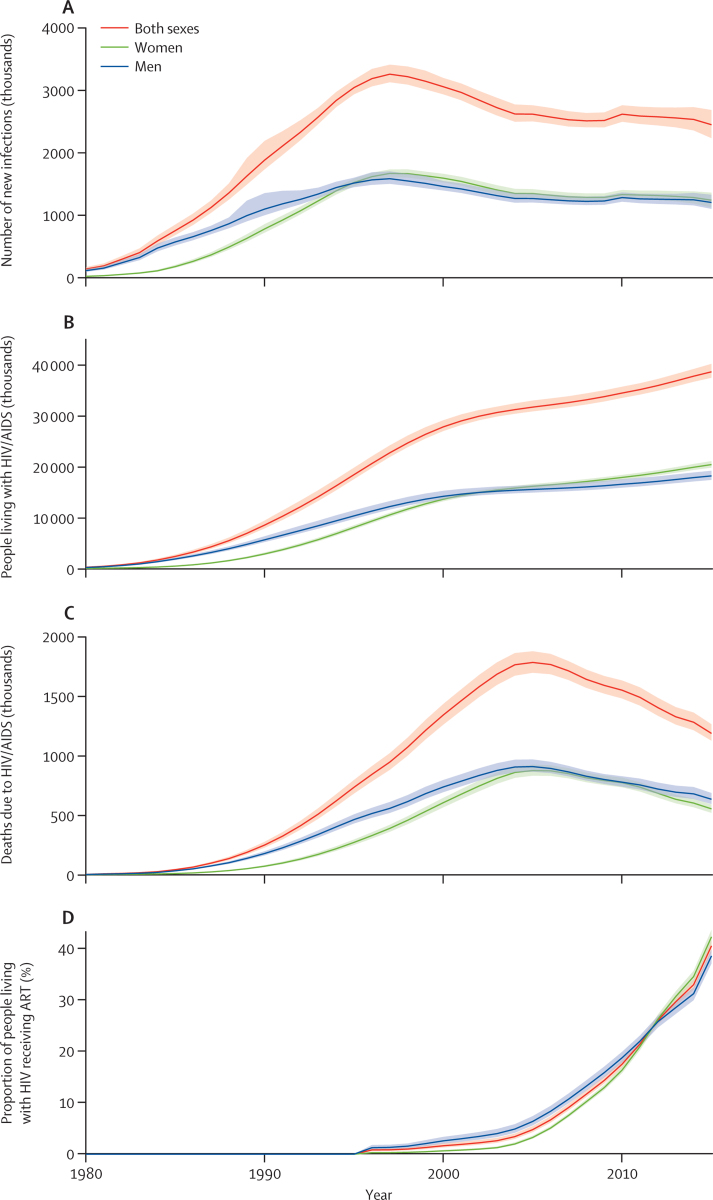
Evolution of the HIV epidemic from 1980 to 2015 Global estimates of new HIV infections (A), people living with HIV/AIDS (B), HIV/AIDS deaths (C), and proportion of people living with HIV receiving ART (D). Shaded areas show 95% uncertainty intervals. ART=antiretroviral therapy.

Global HIV incidence peaked in 1997, at 3·3 million new infections (95% UI 3·1–3·4 million), decreasing by 4·8% (4·0–5·5) per year to 2005 (figure 1A). From 2005 to 2015, the global incidence remained relatively stable, at about 2·5–2·6 million per year (figure 1A). Prevalence of people living with HIV increased rapidly, from 2·4 million (95% UI 2·1–2·8 million) in 1985, to 28·0 million (27·1–29·3 million) in 2000 (figure 1B). From 2000 to 2015, the number of people living with HIV increased by 0·8% (95% UI 0·6–1·0) per year, reaching 38·8 million (37·6–40·4 million) in 2015 (figure 1B). Global mortality peaked in 2005, at 1·8 million (95% UI 1·7–1·9 million) and subsequently fell by 5·5% (95% UI 5·0–5·9) per year to 1·2 million (1·1–1·3 million) in 2015 (figure 1C). The proportion of people living with HIV and receiving ART increased rapidly for both sexes between 2005 and 2015, from 6·4% (95% UI 5·6–7·4) to 38·6% (37·2–40·0) of men, and from 3·3% (3·0–3·6) to 42·4% (41·0–43·7) of women (figure 1D).

**Figure 2:**
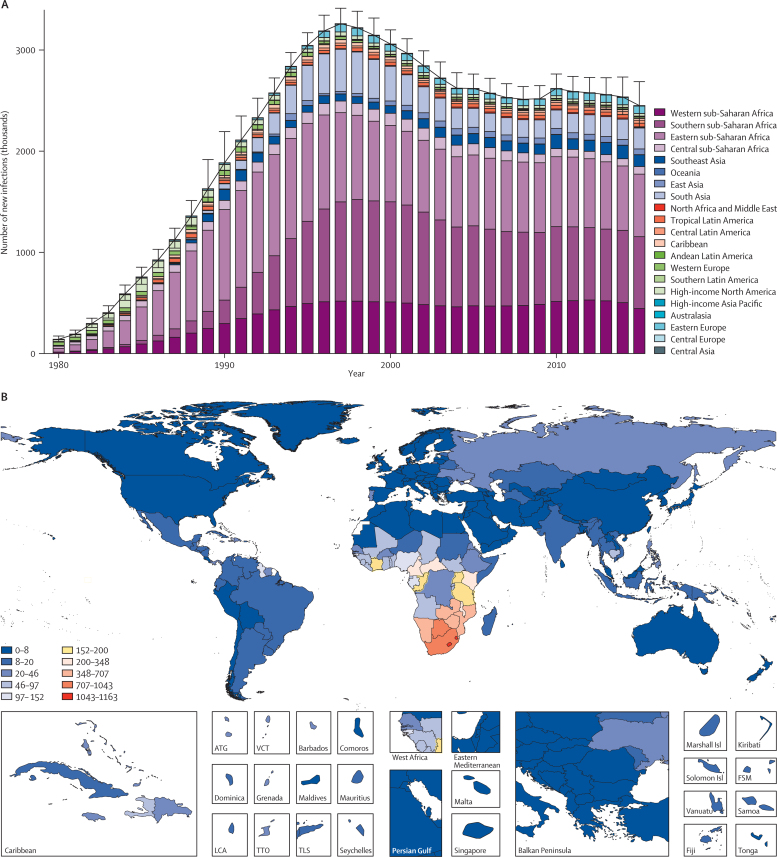
Incidence of new HIV infections from 1980 to 2015, and HIV incidence in 2015 Global number of new HIV infections by region (A). Bars show the mean number of estimated new infections within a given year. Error bars represent 95% uncertainty intervals. Each Global Burden of Disease region is represented by a separate colour. HIV incidence by country (B). We calculated incidence as cumulative new cases of HIV throughout the year divided by the total population at the mid-year. Rates are per 100 000 people. Colour bins correspond to the 0–50th, 50–70th, 70–80th, 80–90th, 90th–92nd, 92nd–94th, 96–98th, 98–99th, and 99–100th percentiles to highlight variation within sub-Saharan Africa. ATG=Antigua and Barbuda. VCT=Saint Vincent and the Grenadines. LCA=Saint Lucia. TTO=Trinidad and Tobago. TLS=Timor-Leste. FSM=Federated States of Micronesia.

In 2015, 1·8 million (95% UI 1·7–2·1 million) new HIV infections, 75·4% (71·7–78·5) of new cases, were in sub-Saharan Africa, with large proportions in western, southern, and eastern sub-Saharan Africa (figure 2A). Outside sub-Saharan Africa, south Asia accounted for 206 830 (171 790–249 700), or 8·5% (7·0–10·0), of new infections per year (figure 2A). Southeast Asia accounted for 4·7% (95% UI 2·8–8·1) of global infections in 2015, and east Asia accounted for 2·3% (1·7–3·1; figure 2A). Distributions of new infections by sex were broadly similar (appendix pp 26–38); and prevalence and mortality have also been greatest in sub-Saharan Africa (appendix pp 59, 60). HIV infection rates varied tremendously across countries in 2015 (figure 2B; see appendix pp 64, 65 for incidence for 1990 and 2005). The highest rates of infection were in southern Africa, with more than 1% of the population per year becoming infected in Botswana, Lesotho, and Swaziland (figure 2B). Within sub-Saharan Africa, rates in excess of 150 per 100 000 people occurred in a cluster of countries from Nigeria to Tanzania, with the notable exceptions of the Democratic Republic of the Congo (42·0 per 100 000; 95% UI 12·3–101·7) and Ethiopia (39·4 per 100 000; 19·7–62·5; figure 2B). The highest estimated incidence rates in Europe were recorded in Russia, and in Asia were recorded in Cambodia (figure 2B). In the Americas, only Belize, Guyana, and Haiti had rates of more than 50 per 100 000 people (figure 2B). Among the countries in the highest quintile of sociodemographic index (a composite indicator based on equally weighted estimates of lag-distributed income per capita, average years of education among populations over 15 years, and total fertility rate),^15^ countries with incidence rates of more than 15 infections per 100 000 people included Antigua and Barbuda, the Bahamas, Bermuda, Trinidad and Tobago, and Russia. Annualised rates of change show that although incidence substantially declined globally from 2005 to 2015, rates increased in 74 countries (table).

**Figure 3:**
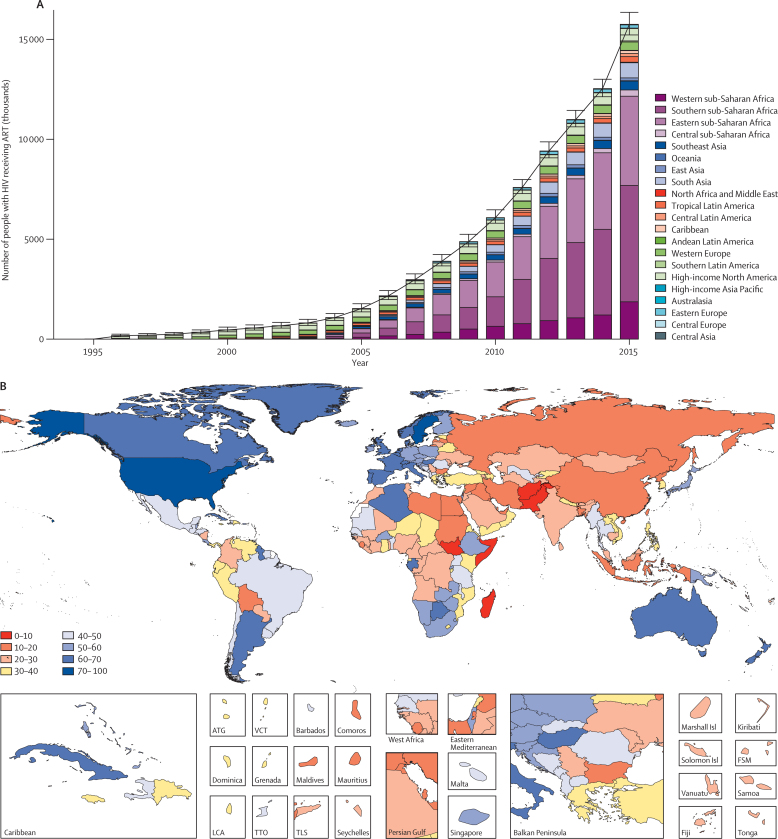
Number of people living with HIV receiving ART from 1995 to 2015, and the proportion living with HIV receiving ART in 2015 Number of people living with HIV receiving ART by region (A). Bars represent the mean number of people living with HIV who received ART within a given year. Error bars represent 95% uncertainty intervals. Each Global Burden of Disease (GBD) region is represented by a separate colour. Proportion of people living with HIV receiving ART by country (B). The number of people living with HIV receiving ART and the total number of people living with HIV are year-end point prevalences. ART=antiretroviral therapy. ATG=Antigua and Barbuda. VCT=Saint Vincent and the Grenadines. LCA=Saint Lucia. TTO=Trinidad and Tobago. TLS=Timor-Leste. FSM=Federated States of Micronesia.

Due to improved access to treatment, prevalence compared with incidence was higher in countries with a high sociodemographic index (table). Six countries (Botswana, Lesotho, Namibia, Swaziland, South Africa, and Zimbabwe) had a HIV prevalence of more than 10% of the entire population. Nine countries in sub-Saharan Africa (Central African Republic, Cameroon, Equatorial Guinea, Kenya, Mozambique, Malawi, Tanzania, Uganda, and Zambia) had a prevalence of more than 2·5% of the entire population. Outside sub-Saharan Africa, a further 11 countries (the Bahamas, Belize, Bermuda, Dominican Republic, Guyana, Haiti, Cambodia, Portugal, Suriname, Trinidad and Tobago, and Saint Vincent and the Grenadines) had prevalence rates between 0·5% and 2·5% (appendix p 57). In the past 10 years, global scale-up of ART has been extraordinary, especially in eastern and southern sub-Saharan Africa (figure 3A). However, despite these increases, the proportion of people living with HIV and receiving ART is highly variable and remains at very low levels in many countries, particularly in the Middle East and North Africa, eastern Europe, central Asia, east Asia, and some countries in southeast Asia (figure 3B). We recorded coverage in excess of 40% in North America, western Europe, Australasia; the arc of countries in eastern South America, from Guyana to Argentina and Chile; and the corridor of countries from Uganda to South Africa (figure 3B).

**Figure 4:**
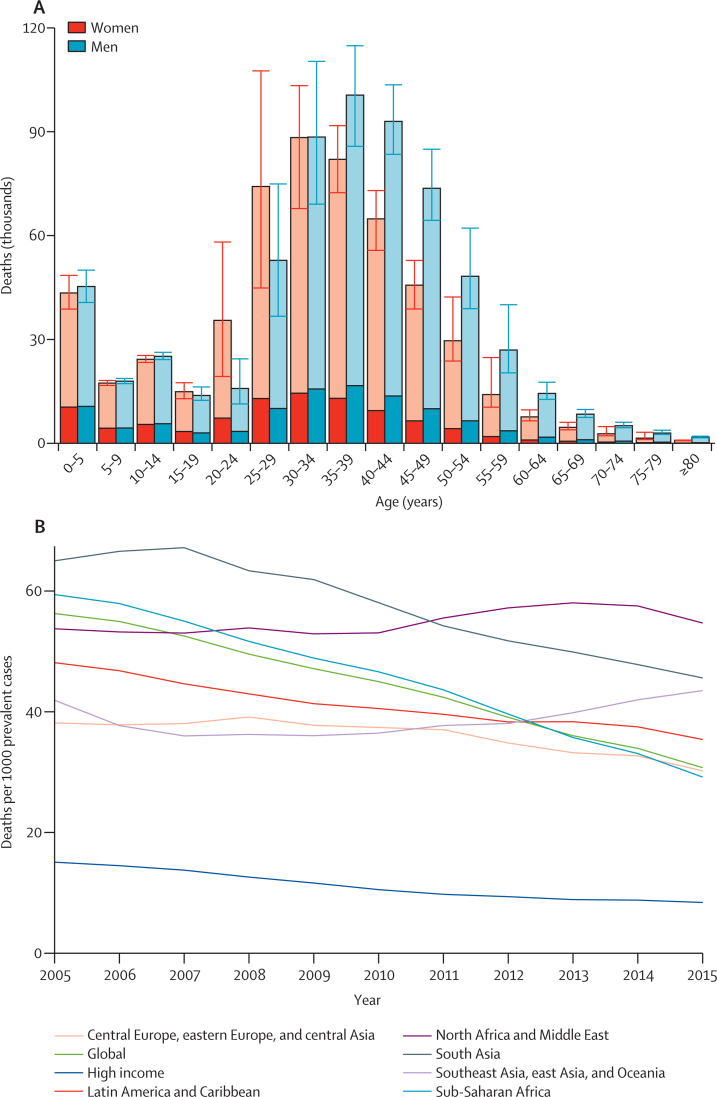
Global HIV/AIDS deaths, 2005–15 Global deaths caused by HIV/AIDS resulting in either mycobacterial infection (tuberculosis) or other diseases, by age and sex in 2015 (A); dark shading indicates deaths caused by tuberculosis associated with HIV; light shading indicates deaths caused by other diseases resulting from HIV; error bars show 95% uncertainty intervals. Mean estimates of global and super-regional HIV/AIDS deaths per prevalent case fom 2005 to 2015 (B).

HIV death rates and recent time trends vary greatly across countries (table). Deaths vary substantially by age, showing both the patterns of incidence by age, differential rates of progression by sex and age, and differential ART coverage (figure 4A). More women than men died in people aged 15–29 years; after age 35 years, there were more deaths in men (figure 4A). Deaths in people aged 50 years and older account for 10% (95% UI 3·8–11·8) of deaths in men and 7·6% (1·5–9·8) of deaths in women (figure 4A). We recorded substantial heterogeneity in HIV mortality among countries in 2015 (appendix p 63). Among HIV/AIDS deaths in 2015, 17·8% were caused by HIV and tuberculosis co-infection, down from 19·6% in 2005 (figure 4A). We compared HIV deaths with the number of people living with HIV to provide a simple estimate of the annual excess mortality rate (figure 4B). This ratio is a function of the timing of the epidemic and the access to and quality of ART and other care. Of note, this ratio was much lower in GBD high-income regions than in other GBD super-regions.

**Figure 5:**
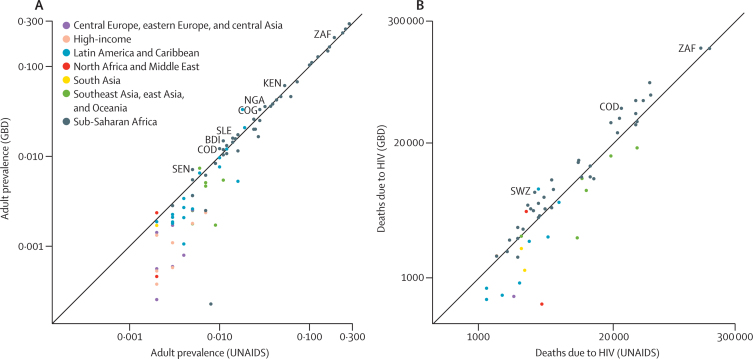
Comparison of GBD 2015 and UNAIDS 2014 estimates Adult HIV prevalence rate (A) and estimates of death caused by HIV/AIDS (B). UNAIDS' published prevalence values are limited to three decimal places. The x and y values of each point are the log transformation of the mean estimates from UNAIDS and GBD, respectively, enabling variation to be seen despite disparate values. Tick-mark labels on the x and y axes are the value of the mean estimate before log transformation (ie, the real value and not the log-transformed value is shown). Locations mentioned in the manuscript are highlighted by plotting the ISO 3 code of the location. Each location is plotted with a different colour by super-region. GBD=Global Burden of Disease. UNAIDS=the Joint United Nations Programme on HIV and AIDS. ZAF=South Africa. KEN=Kenya. NGA=Nigeria. COG=Democratic Republic of the Congo. SLE=Sierra Leone. BDI=Burkina Faso. COD=Congo. SEN=Senegal. SWZ=Swaziland.

At the time of writing, the latest available assessment from UNAIDS was published in 2016, at the global and regional level only.^26^ UNAIDS country level estimates are from their 2014 update for years up to 2014.^27^ GBD 2015 estimates of prevalence are in accordance with the UNAIDS estimates. For 2015, estimations of the people living with HIV were 38·8 million (95% UI 37·6–40·4 million) in GBD 2015, and 36·7 million (34·0–39·8 million) in UNAIDS 2016. Comparisons of prevalence estimates at the country level in 2005 and 2014, show strong concordance between the two estimate series, with an average intraclass correlation coefficient of 0·997 (figure 5A shows prevalence from both sources for 2014). The highest relative differences in prevalence among sub-Saharan African countries in 2014 were in Senegal, Burundi, Democratic Republic of the Congo, Congo, Kenya, Sierra Leone, Nigeria, and South Africa, where GBD 2015 estimates are at least 10% higher than those from UNAIDS 2014 (figure 5A). UNAIDS tends to have much higher estimates of mortality at the peak of the HIV/AIDS epidemic around 2005, and lower estimates in 2014, than GBD 2015; we noted a much higher level of heterogeneity at the country level (figure 5B). For countries in sub-Saharan Africa, GBD estimates of mortality are higher than those from UNAIDS 2014 for 26 countries. Among these countries, GBD estimates are more than 10% higher than UNAIDS 2014 estimates in 22 countries. For South Africa, our estimated deaths are 17·2% higher than UNAIDS 2014 estimates. The highest differences are in Swaziland and Democratic Republic of the Congo, where GBD 2015 estimates are more than 80% higher than UNAIDS' (figure 5B).

For estimates of annual new infections, UNAIDS 2014 has slightly higher estimates for years before 2000. Although the estimates are similar between the two series for most of the 2000s, the series have differed substantially since 2007 at the global level. UNAIDS 2014 estimated a much faster rate of decline in annual new infections than did GBD 2015. Globally, GBD 2015 estimates about 2·5 million new infections in 2014, whereas UNAIDS estimates about 2 million for the same year. UNAIDS 2016 has slightly higher estimates of incidence than the 2014 publication, at 2·1 million for 2015. In terms of annualised rate of decline in new infections between 2005 and 2014, GBD 2015 estimates about a 0·4% decline per year, whereas the UNAIDS estimates from 2014 show a 3·3% decline per year. In only seven countries (Madagascar, Democratic Republic of the Congo, Burkina Faso, Guinea-Bissau, Chad, Rwanda, and The Gambia) in sub-Saharan Africa, annualised rates of decline in new infections are faster in GBD 2015 than in UNAIDS 2014. In Côte d'Ivoire, Burundi, Eritrea, Zimbabwe, Lesotho, Nigeria, Botswana, and Kenya, GBD 2015 estimates an increase in numbers of new infections, whereas UNAIDS 2014 predicts a decline. The biggest difference is in Kenya, where results from GBD 2015 show an increase in annual new infections from 60 000 in 2005, to 146 700 in 2014, whereas UNAIDS shows a decrease from 73 000 to 56 000 during the same period.

## Discussion

Remarkable progress has been made in curbing the HIV/AIDS epidemic worldwide. HIV incidence reached its peak in 1997, and HIV deaths have been declining since the mid-2000s. However, annual incidence has stayed relatively constant since 2005, after a period of faster decline between 1997 and 2005. The number of people living with HIV/AIDS has been steadily increasing, and reached 38·8 million in 2015. At the country level, disparate levels and trends of the epidemic persist. These updated estimates at the global level are similar to those published in the GBD 2013 iteration for deaths; however, our present estimates for incidence and prevalence are lower for 2013 than in GBD 2013.^1^

The unfolding global HIV pandemic has advanced through three phases during which HIV/AIDS mortality has increased from 4·73 per 100 000 in 1995, the 39th-ranked cause of death, to 16·18 per 100 000 in 2015, the 11th-ranked cause of death worldwide. In the initial phase (1981–97), global HIV incidence and the number of people living with HIV increased, followed by huge increases in deaths related to the disease. From 1998 to 2005, incidence declined by 25·4%; however, because of the lag between infection and mortality, the number of deaths caused by HIV increased. In the third phase, mass scale-up of prevention of mother-to-child transmission and ART, particularly in low-income sub-Saharan Africa, led to a phase of declining HIV mortality, a decade of stagnation in the decline of global incidence rates, and steadily rising prevalence. These global patterns mask well documented but extraordinary heterogeneity across countries. Epidemics leading to more than 2·5% of the population being infected have happened largely in eastern, southern, and central sub-Saharan Africa. Although death rates and incidence declined in the past decade in many of these countries, they are increasing in many others where prevalence has been lower until now, such as Indonesia and the Philippines. The scale-up of ART, a key driver of the trends, has led to 41% of people living with HIV receiving ART worldwide.^7^

The scale-up of interventions for HIV/AIDS represents one of the great successes of global health collective action. This scale-up, particularly in low-income countries, has been fuelled by the increase in development assistance for HIV from $1·3 billion in 2000, to $10·8 billion in 2015.^12^, ^13^ The need for HIV programmes, particularly ART programmes, continues to grow because of both the sustained high incidence of infections and the success of ART in extending the lifespan of people living with HIV. However, since 2010, development assistance for HIV has remained nearly constant.^12^ This absence of additional funding is by stark contrast with the $36 billion needed annually to achieve the UN goal to end AIDS by 2030, as estimated by Piot and colleagues.^9^ UNAIDS and other international development agencies hope that the growing need for funding will be partly solved by expanded health spending by low-income countries.^28^, ^29^ However, Dieleman and colleagues^12^, ^13^ suggest that, on the basis of trends in the past few years, health spending in low-income countries will grow only slightly in the next 25 years. How will the impending financing gap be addressed? In middle-income countries, increased commitments to funding health programmes from national budgets could fill the gap. But in low-income countries, where, as in eastern and some countries in southern sub-Saharan Africa, HIV rates are the highest, domestic resources will not be sufficient. Dieleman and colleagues^30^ projected that government health expenditure is going to increase from $30·8 billion (95% UI 29·9–31·8 billion) in 2015, to $53·1 billion (47·5–57·9 billion) in 2030, in southern sub-Saharan Africa.

Meeting the needs of people living with HIV will require a combination of concentrating development assistance for HIV on these low-income countries, improving the efficiency of HIV programmes, increasing domestic financing, lowering the cost of treatment (including prices of ART drugs), and reducing future incidence through more concerted efforts. Development assistance efforts will also need to be scaled up if the free flow of low-cost generic drugs is hampered. Additionally, public and private sectors need to be incentivised to continue research and development of new and better prevention and treatment strategies to combat the epidemic in the long term. Special efforts need to be made in high-risk populations in both concentrated and generalised epidemic settings in view of the continued high rate of transmission among these subpopulations, including men who have sex with men and injecting drug users. However, on the basis of the epidemiological and financial trends, there is a major risk of a substantial shortfall in necessary funds to sustain life-saving ART programmes. The scarcity of adequate funds to provide ART for people living with HIV, together with the possibility of increasing drug resistance to existing ART treatments, will make achievement of the goal to end AIDS by 2030 extremely difficult.

WHO now recommends universal ART for all people with HIV.^31^, ^32^ In 2015, only 41% of people living with HIV were receiving ART; however, the 90-90-90 goals imply that 81% should be receiving ART and 73% will have viral suppression, which no country has yet achieved. Achievement of 81% ART coverage would require extension of ART coverage to at least 15·5 million additional people living with HIV by 2020, which implies an addition of 3·1 million per year between 2015 and 2020, while ensuring complete treatment adherence. Concerted efforts will be needed to scale up detection of new infections to meet the target of 90% of people knowing their status. The targeted expansion in ART coverage would play an important part in reducing the still high number of individuals dying from HIV. However, such expansion has enormous cost implications in an era when even maintenance of coverage in some low-income settings could be at risk in the presence of declining development assistance for health. Increased ART coverage might also play a part in reducing population transmission of HIV and therefore incidence.^33^, ^34^ The quality of ART embodied in the third 90 target of the UNAIDS strategy remains a major issue, as does the potential role of other care in extending survival. The simple comparison between HIV deaths and HIV prevalence shows that death rates in HIV-positive individuals are much lower in high-income countries than elsewhere. In fact, probability of death from HIV/AIDS while on ART in sub-Saharan Africa is on average 6·5 times higher than the probability in high-income countries among different age groups and time since start of ART treatment.

Calls for the end of AIDS have captured the imagination of the global health community.^35^ Largely as a result of the course of the HIV epidemic itself and spreading awareness of HIV among the general population, incidence declined between 1997, the year with peaked incidence, and 2005. However, our present estimates of HIV incidence, albeit driven mostly by prevalence data, suggest that incidence might not have declined much in the past decade. Incidence remained high, despite that much development assistance for HIV was spent on prevention programmes. Once the notable success of scale-up of prevention of mother-to-child transmission and reductions in transmission to children is accounted for, adult incidence remained even more resistant to change in the past 10 years. Effective strategies, such as male circumcision and PrEP, are available to reduce transmission even without changing sexual behaviour.^36^, ^37^, ^38^ Barrier methods for HIV prevention are also effective in reducing risk for transmission, as are some interventions targeting high-risk groups, such as needle exchanges.^39^, ^40^ Despite the existence of these approaches, incidence has not changed substantially. Although incidence has declined from 40·2 to 33·2 per 100 000 people at an annualised rate of decline of 1·9%, annual new infections have stayed relatively constant at about 2·5 million a year for the past decade. This finding could be explained by many factors, including that viral load suppression might be lower than the estimated 70% in low-income and middle-income countries, that ART coverage might be exaggerated in some countries, or that the rate of unsafe sex could be increasing in settings where the perceived risk of HIV has been reduced.

Worldwide, Millennium Development Goal 6, to halt and reverse the spread of HIV and provide universal access to treatment for those who need it by 2015, has not been achieved. Between 2005 and 2015, 102 of 195 countries have experienced an increase in annual new infections. In sub-Saharan Africa, 15 of 46 countries managed to decrease annual infection during the same period. No countries had achieved the 81% target in 2015, and most developing countries have important gaps to fill by 2020.^1^ Sustainable Development Goal 3 aims to end HIV/AIDS by 2030. Achievement of such an ambitious goal will require great improvements in prevention efforts. The PEPFAR pivot, with its focus on high-transmission areas, might provide one such strategy,^35^ but the effectiveness of this approach is unproven, and the planning and evaluation for such programmes needs more granular data on the epidemic level and trends at the subnational level, which are still largely missing in most countries. To further reduce mortality from HIV/AIDS, another priority should be towards prevention, detection, and treatment of tuberculosis among people living with HIV as part of a strategy to reduce HIV disease progression and transmission, because tuberculosis, although largely preventable and treatable, is one of the most common opportunistic infections and the leading cause of death among HIV-infected individuals, as our study has shown.

Our assessment of HIV incidence and mortality in countries without vital registration data is driven by prevalence surveys and surveillance data on the prevalence of HIV among individuals attending antenatal care clinics. Estimation of incidence from prevalence is based on a set of assumptions about CD4 progression rates, off-ART and on-ART HIV death rates, and ART coverage. Such statistical back-estimation is inherently uncertain for recent time periods for which changes in incidence will not have changed prevalence as quickly. So far, efforts to develop incidence assays that can differentiate new from old infections have not been sufficiently robust or widely enough deployed to include in our or UNAIDS' estimation efforts, and the necessary sample sizes to track incidence could be challenging to obtain.^41^, ^42^, ^43^ Repeated measurements, such as the Swaziland HIV Incidence Measurement Survey (SHIMS), provide information about incidence in very few settings.^44^ Compared with the prevalence-based calculations, the SHIMS results show 2·4 infections per 100 person-years (95% UI 2·06–2·75) for 2011, which is consistent with GBD 2015 estimations of 2·15 infections per 100 people (1·91–2·46) for the same year. In view of the heightened focus on reducing HIV incidence as part of the end-of-AIDS vision, more efforts are needed to systematically supplement the approach of estimating incidence with prevalence data by use of additional information about case notifications with CD4 status, HIV viral load, and alternative assays as they emerge.

Our models, in addition to UNAIDS models for estimating HIV incidence and mortality, depend heavily on estimates of prevention of mother-to-child transmission and ART coverage. These numbers are developed by UNAIDS in consultation with national governments, the Global Fund, and PEPFAR.^45^ However, the underlying data at the facility or provider level are not available for inspection, critical appraisal, or validation. Evidence from countries where survey data for use of ART are available, such as Kenya, suggests that national assessments of numbers of people on ART collated by UNAIDS might be too high.^46^, ^47^, ^48^ If these findings were true in other countries, our estimates of ART coverage could likewise be exaggerated, as could our estimates of deaths from HIV. Data transparency for models used in global health estimation is rapidly increasing. GBD have adopted the GATHER guidelines developed by WHO and other partners.^25^ In the future, having input data on ART and prevention of mother-to-child transmission meet the GATHER guideline bar of transparency would be highly beneficial. Political sensitivities in some countries have restricted the transparency of UNAIDS on this issue; even the basic incidence and prevalence estimates generated by UNAIDS cannot be released for some countries such as India and Russia because of such issues. Fostering a culture of greater transparency and accountability for HIV prevention and treatment programmes will benefit everyone concerned with tackling HIV more effectively in the future.

Subnational assessments, when available, suggest much spatial heterogeneity of HIV incidence, prevalence, and death.^49^, ^50^, ^51^ Use of more disaggregated assessments of the HIV epidemic will hopefully improve the quality of the results and the relevance to HIV prevention and treatment programmes. Disaggregated assessments of prevalence derived from survey data and surveillance data from antenatal care clinics are feasible. Progress will be needed on the availability of data for prevention of mother-to-child transmission and ART at the local level. Perhaps even more challenging is the need for estimates of the various demographic inputs required for the modelling efforts, including migration, fertility, and HIV-free mortality. The push toward district-level or even more fine-grained estimation is one of the most promising directions for improved estimation of the epidemic overall.

Substantial differences between men and women remain in many aspects of the HIV epidemic. Our analysis shows that the age pattern of HIV/AIDS mortality is younger in women than in men. This finding is largely thought to result from age-disparate relationships in which men tend to have sex with women younger than them.^52^, ^53^ Furthermore, more women use ART, as shown by the roughly 15·4% higher ART coverage for women than men in 2015. We also recorded a high level of heterogeneity at the country level, with female ART coverage 50% higher than male coverage in countries such as Gabon, The Gambia, Nigeria, and Sierra Leone; at the same time, in India, Lithuania, and Maldives, male ART coverage was 50% higher.

The GBD 2015 estimates of prevalence are in line with those from UNAIDS 2014. The high concordance of country-level prevalence estimates between the two series is unsurprising given that they are both based on similar input datasets for prevalence. Much of the difference between the two estimates is a result of different assumptions of on-ART and off-ART mortality, background HIV-free mortality rate, initial CD4 distribution, and CD4 progression ratios. Further studies and close collaboration between UNAIDS and GBD are needed to fully understand the relative contribution of each of the aforementioned factors to the difference in estimates of incidence, prevalence, and mortality at the country level.

This analysis of data for HIV has several further limitations. First, we have used data from the Antiretroviral Therapy Cohort Collaboration to improve the assessment of death rates of patients on ART in high-income countries. However, we have not been able to incorporate data for the variation in quality of ART programmes such as viral load suppression by country. This information is not widely available. Second, our novel cohort incidence bias adjustment method leverages cause-of-death data to correct past incidence for each cohort. Although our testing shows that the method works fairly well, the information content in the approach for adjustments in the most recent time period is much more restricted than for earlier periods. Estimates of incidence with this approach in the most recent time periods might be biased. Third, we have attempted to propagate multiple sources of uncertainty into our final estimates. Because we did not include uncertainty caused by variation in the quality of ART and might underestimate uncertainty in ART coverage, our final uncertainty intervals could be too narrow. At the global level, our uncertainty levels might also be too narrow because we assume uncertainty in each country is independent of other countries. Fourth, we have not used the surveillance data for new cases in our analysis. Integration of such information in the future will probably increase the accuracy of incidence and prevalence estimates. Fifth, prevalence and programme data are still sparse in most countries. Prevalence estimates are largely determined by adjusted antenatal clinic data and national surveys. To depict the epidemic in populations accurately, rigorous data collection is needed. For example, ART coverage data should be directly collected in surveys through viral load testing, such as in the Lesotho 2014 Demographic and Health Survey,^54^ and questions about ART. Sixth, our study focuses on deaths with HIV/AIDS as the underlying cause of death and does not account for excess mortality from other non-communicable causes of deaths among people living with HIV. Seventh, input data tend to be sparse for the most recent time period, and our models might have not captured the recent progress and lack thereof in some countries. Eighth, our models have not directly used other important variables, such as prevalence of sexually transmitted infections or rates of ART adherence, ART treatment failure, and HIV testing, as used by Optima (appendix p 56).^55^ These variables should be included in future updates to improve the precision of our estimates. Finally, although we integrated HIV cause-specific mortality data in our modelling framework for a large group of countries with vital registration data, inclusion of additional data sources such as HIV surveillance and case report in our analytical framework could improve the accuracy of our incidence estimates.

Enormous progress has been made in reducing HIV deaths, especially in low-income countries, through the expansion of prevention of mother-to-child transmission and ART programmes funded largely through development assistance for HIV. However, achievement of the UNAIDS 90-90-90 targets will require major changes in how programmes are delivered and financed. Global efforts have had less impact on the incidence of new infections than on HIV mortality. Ending the AIDS epidemic by 2030 will require a dramatic change in how HIV prevention is pursued.

Correspondence to: Dr Haidong Wang, Institute for Health Metrics and Evaluation, University of Washington, Seattle, WA 98121, USA haidong@uw.edu

**This online publication has been corrected. The corrected version first appeared at thelancet.com/hiv on August 22, 2016**

## Figures and Tables

**Table:** Country-specific estimates of new HIV infections, people living with HIV, HIV/AIDS deaths, and ART coverage in 2015, and ARCs of age-standardised incidence, prevalence, and mortality from 2005 to 2015

			**New infections (in thousands)**	**People living with HIV (in thousands)**	**HIV/AIDS deaths (in thousands)**	**ART coverage per 100 people living with HIV (%)**	**Age-standardised incidence ARC from 2005 to 2015**	**Age-standardised prevalence ARC from 2005 to 2015**	**Age-standardised mortality ARC from 2005 to 2015**
Global			2450·92 (2236·13 to 2686·79)	38 802·50 (37 635·88 to 40 371·67)	1192·57 (1131·11 to 1270·05)	40·60 (39·36 to 41·80)	−0·02 (−0·03 to −0·01)	0·01 (0·00 to 0·01)	−0·05 (−0·06 to −0·05)
	High SDI		101·75 (75·18 to 146·96)	2204·18 (1751·36 to 2799·27)	33·51 (31·96 to 35·43)	51·49 (43·90 to 57·55)	0·01 (−0·01 to 0·04)	0·01 (0·00 to 0·02)	−0·01 (−0·02 to −0·01)
	High-to-middle SDI		646·76 (557·07 to 748·55)	10 421·94 (9873·26 to 10 989·83)	240·15 (224·08 to 259·28)	48·01 (45·99 to 50·13)	−0·02 (−0·03 to −0·01)	0·01 (0·01 to 0·02)	−0·05 (−0·06 to −0·05)
	Middle SDI		298·33 (238·18 to 394·91)	4155·45 (3616·14 to 5163·64)	131·57 (111·27 to 183·00)	37·66 (32·68 to 40·83)	0·00 (−0·02 to 0·02)	0·02 (0·01 to 0·03)	−0·02 (−0·04 to −0·00)
	Low-to-middle SDI		796·30 (655·25 to 951·76)	11 783·44 (11 251·57 to 12 472·97)	408·87 (368·10 to 457·42)	35·48 (33·62 to 37·52)	−0·01 (−0·03 to 0·01)	−0·00 (−0·01 to 0·00)	−0·06 (−0·07 to −0·06)
	Low SDI		606·54 (510·14 to 707·36)	10 213·36 (9762·90 to 10 684·37)	377·68 (350·43 to 408·08)	37·89 (35·93 to 39·79)	−0·05 (−0·07 to −0·03)	−0·01 (−0·02 to −0·01)	−0·08 (−0·09 to −0·07)
High-income			45·67 (37·88 to 53·92)	1660·18 (1359·94 to 1997·98)	13·95 (13·79 to 14·11)	66·91 (64·76 to 69·43)	−0·01 (−0·02 to −0·00)	−0·00 (−0·01 to 0·00)	−0·06 (−0·06 to −0·05)
	High-income North America		24·16 (18·76 to 31·10)	882·60 (692·93 to 1136·45)	7·89 (7·79 to 7·98)	69·86 (66·81 to 73·51)	−0·02 (−0·04 to −0·01)	−0·00 (−0·01 to 0·00)	−0·07 (−0·07 to −0·07)
		Canada	1·11 (0·18 to 2·81)	49·25 (15·89 to 102·34)	0·31 (0·29 to 0·33)	64·14 (56·58 to 73·43)	−0·03 (−0·14 to 0·02)	−0·01 (−0·03 to 0·01)	−0·06 (−0·07 to −0·05)
		Greenland	0·00 (0·00 to 0·01)	0·23 (0·06 to 0·55)	0·00 (0·00 to 0·00)	61·88 (52·84 to 69·43)	−0·10 (−0·65 to −0·02)	−0·01 (−0·03 to 0·00)	−0·03 (−0·07 to 0·01)
		USA	23·04 (17·68 to 29·96)	833·03 (648·62 to 1078·06)	7·57 (7·48 to 7·67)	70·18 (67·09 to 74·00)	−0·02 (−0·04 to −0·01)	−0·00 (−0·01 to 0·00)	−0·07 (−0·07 to −0·07)
	Australasia		0·45 (0·19 to 0·89)	18·69 (7·37 to 37·10)	0·10 (0·09 to 0·10)	62·24 (57·73 to 67·54)	−0·02 (−0·04 to −0·01)	−0·00 (−0·01 to 0·00)	−0·04 (−0·05 to −0·03)
		Australia	0·39 (0·15 to 0·84)	16·24 (5·20 to 34·28)	0·09 (0·08 to 0·09)	62·38 (57·07 to 68·35)	−0·02 (−0·03 to −0·01)	−0·01 (−0·02 to 0·00)	−0·04 (−0·05 to −0·03)
		New Zealand	0·06 (0·02 to 0·13)	2·45 (0·71 to 5·38)	0·01 (0·01 to 0·01)	60·68 (54·45 to 68·06)	−0·03 (−0·10 to −0·01)	0·00 (−0·01 to 0·01)	−0·04 (−0·05 to −0·03)
	High-income Asia Pacific		0·75 (0·55 to 1·02)	22·06 (14·82 to 35·17)	0·32 (0·31 to 0·33)	49·98 (45·98 to 53·54)	−0·03 (−0·09 to −0·00)	0·02 (0·01 to 0·03)	−0·01 (−0·01 to −0·01)
		Brunei	0·01 (0·00 to 0·03)	0·26 (0·09 to 0·59)	0·00 (0·00 to 0·00)	37·66 (29·23 to 47·75)	−0·03 (−0·16 to 0·02)	0·02 (−0·00 to 0·04)	−0·02 (−0·06 to 0·01)
		Japan	0·50 (0·40 to 0·60)	10·41 (8·40 to 12·69)	0·17 (0·17 to 0·17)	57·43 (55·29 to 60·02)	0·01 (−0·00 to 0·02)	0·04 (0·03 to 0·04)	−0·03 (−0·03 to −0·03)
		Singapore	0·05 (0·02 to 0·10)	1·85 (0·60 to 4·06)	0·01 (0·01 to 0·01)	54·61 (45·56 to 64·61)	0·01 (−0·05 to 0·05)	0·01 (−0·01 to 0·02)	0·12 (0·11 to 0·12)
		South Korea	0·19 (0·02 to 0·43)	9·54 (2·92 to 21·96)	0·14 (0·13 to 0·14)	39·34 (31·76 to 47·08)	−0·12 (−0·32 to −0·04)	0·00 (−0·01 to 0·02)	0·01 (0·01 to 0·02)
	Western Europe		12·89 (9·48 to 16·95)	651·38 (448·53 to 896·75)	3·42 (3·35 to 3·50)	63·81 (60·91 to 67·06)	−0·03 (−0·04 to −0·02)	−0·01 (−0·01 to −0·00)	−0·06 (−0·06 to −0·05)
		Andorra	0·00 (0·00 to 0·01)	0·21 (0·02 to 1·37)	0·00 (0·00 to 0·01)	57·49 (32·52 to 80·56)	−0·04 (−0·72 to 0·10)	0·01 (−0·03 to 0·08)	−0·01 (−0·08 to 0·08)
		Austria	0·31 (0·10 to 0·71)	11·65 (2·72 to 28·30)	0·04 (0·04 to 0·04)	55·15 (48·70 to 62·52)	−0·04 (−0·09 to −0·01)	0·01 (−0·00 to 0·02)	−0·06 (−0·06 to −0·05)
		Belgium	0·21 (0·06 to 0·47)	10·68 (2·90 to 25·23)	0·05 (0·05 to 0·05)	61·74 (55·20 to 68·73)	−0·03 (−0·12 to 0·00)	−0·00 (−0·02 to 0·01)	−0·04 (−0·05 to −0·04)
		Cyprus	0·01 (0·00 to 0·03)	0·39 (0·11 to 0·88)	0·00 (0·00 to 0·00)	48·50 (40·52 to 58·86)	−0·06 (−0·67 to 0·02)	0·01 (−0·01 to 0·03)	0·00 (−0·04 to 0·03)
		Denmark	0·13 (0·03 to 0·30)	7·67 (2·13 to 15·27)	0·03 (0·02 to 0·03)	62·61 (55·63 to 70·23)	−0·06 (−0·19 to −0·01)	−0·00 (−0·02 to 0·01)	−0·05 (−0·06 to −0·04)
		Finland	0·03 (0·01 to 0·08)	1·35 (0·36 to 3·09)	0·01 (0·01 to 0·01)	57·85 (51·54 to 64·82)	−0·06 (−0·19 to −0·02)	0·00 (−0·01 to 0·02)	−0·04 (−0·05 to −0·04)
		France	0·96 (0·36 to 2·04)	79·17 (23·19 to 175·70)	0·49 (0·46 to 0·52)	63·37 (54·81 to 71·07)	−0·04 (−0·08 to −0·02)	−0·02 (−0·04 to −0·01)	−0·07 (−0·08 to −0·07)
		Germany	1·76 (0·65 to 3·66)	60·55 (17·98 to 129·32)	0·43 (0·41 to 0·46)	55·55 (47·85 to 64·54)	−0·01 (−0·04 to 0·01)	0·01 (−0·00 to 0·03)	−0·03 (−0·04 to −0·03)
		Greece	0·05 (0·03 to 0·09)	1·22 (0·59 to 2·18)	0·02 (0·02 to 0·02)	39·67 (30·76 to 49·54)	0·01 (−0·02 to 0·03)	0·01 (−0·00 to 0·02)	−0·02 (−0·03 to −0·01)
		Iceland	0·01 (0·00 to 0·02)	0·18 (0·05 to 0·42)	0·00 (0·00 to 0·00)	50·06 (40·56 to 61·16)	−0·01 (−0·18 to 0·03)	0·01 (−0·01 to 0·03)	−0·04 (−0·05 to −0·04)
		Ireland	0·06 (0·01 to 0·14)	2·55 (0·70 to 5·85)	0·01 (0·01 to 0·01)	58·51 (51·76 to 66·14)	−0·03 (−0·15 to −0·00)	−0·00 (−0·02 to 0·01)	−0·00 (−0·01 to 0·01)
		Israel	0·17 (0·05 to 0·35)	4·85 (1·39 to 10·45)	0·04 (0·03 to 0·04)	50·92 (44·16 to 58·93)	−0·01 (−0·09 to 0·01)	0·01 (−0·01 to 0·02)	−0·02 (−0·03 to −0·01)
		Italy	1·96 (0·76 to 4·19)	137·07 (43·51 to 276·32)	0·61 (0·57 to 0·64)	67·08 (62·08 to 72·04)	−0·05 (−0·07 to −0·03)	−0·01 (−0·02 to −0·00)	−0·03 (−0·03 to −0·02)
		Luxembourg	0·01 (0·00 to 0·03)	0·41 (0·12 to 0·96)	0·00 (0·00 to 0·00)	52·04 (43·46 to 61·91)	0·00 (−0·09 to 0·03)	0·01 (−0·01 to 0·02)	−0·05 (−0·05 to −0·04)
		Malta	0·01 (0·00 to 0·02)	0·26 (0·08 to 0·58)	0·00 (0·00 to 0·00)	48·30 (38·71 to 59·59)	0·01 (−0·08 to 0·04)	0·02 (−0·00 to 0·04)	−0·03 (−0·03 to −0·02)
		Netherlands	0·20 (0·07 to 0·47)	14·56 (4·14 to 32·34)	0·05 (0·05 to 0·05)	69·53 (62·81 to 76·01)	−0·02 (−0·07 to 0·01)	−0·02 (−0·03 to −0·01)	−0·07 (−0·07 to −0·06)
		Norway	0·05 (0·02 to 0·11)	2·77 (0·78 to 6·18)	0·01 (0·01 to 0·01)	63·51 (57·61 to 69·83)	−0·02 (−0·09 to −0·00)	−0·01 (−0·02 to 0·00)	−0·08 (−0·09 to −0·07)
		Portugal	2·22 (0·53 to 4·91)	115·25 (32·31 to 263·86)	0·53 (0·50 to 0·56)	60·58 (54·02 to 66·88)	−0·04 (−0·13 to −0·01)	−0·01 (−0·02 to 0·00)	−0·07 (−0·08 to −0·07)
		Spain	2·35 (0·99 to 4·76)	130·33 (39·66 to 281·12)	0·82 (0·77 to 0·87)	65·54 (56·76 to 73·75)	0·01 (−0·01 to 0·02)	−0·02 (−0·03 to −0·01)	−0·08 (−0·09 to −0·07)
		Sweden	0·08 (0·03 to 0·15)	3·69 (1·62 to 6·61)	0·02 (0·02 to 0·02)	76·01 (71·06 to 82·01)	−0·01 (−0·06 to 0·01)	−0·00 (−0·02 to 0·01)	−0·05 (−0·05 to −0·04)
		Switzerland	0·20 (0·05 to 0·45)	13·03 (3·77 to 28·24)	0·04 (0·04 to 0·04)	69·48 (64·10 to 75·77)	0·00 (−0·09 to 0·03)	−0·01 (−0·03 to −0·01)	−0·06 (−0·07 to −0·06)
		UK	2·06 (1·66 to 2·54)	52·67 (41·67 to 66·15)	0·22 (0·21 to 0·22)	61·21 (58·45 to 64·08)	−0·04 (−0·04 to −0·03)	0·02 (0·02 to 0·03)	−0·03 (−0·04 to −0·03)
	Southern Latin America		7·42 (3·55 to 10·30)	85·45 (56·64 to 122·34)	2·23 (2·13 to 2·32)	63·83 (58·62 to 69·84)	0·04 (−0·05 to 0·07)	0·02 (0·00 to 0·03)	−0·01 (−0·02 to −0·01)
		Argentina	6·32 (2·58 to 9·20)	62·94 (36·49 to 96·26)	1·60 (1·51 to 1·70)	69·73 (64·04 to 76·37)	0·07 (−0·05 to 0·09)	0·03 (0·00 to 0·04)	−0·01 (−0·02 to −0·01)
		Chile	0·71 (0·43 to 1·15)	16·25 (7·33 to 32·90)	0·46 (0·43 to 0·49)	45·88 (33·79 to 57·54)	−0·05 (−0·08 to −0·01)	−0·01 (−0·02 to 0·01)	−0·00 (−0·01 to 0·00)
		Uruguay	0·38 (0·20 to 0·64)	6·26 (2·83 to 11·94)	0·16 (0·15 to 0·18)	46·96 (39·24 to 56·35)	−0·01 (−0·05 to 0·03)	0·01 (−0·01 to 0·02)	−0·01 (−0·02 to −0·00)
Eastern Europe, central Europe, and central Asia			78·25 (52·91 to 122·49)	940·86 (617·41 to 1490·53)	28·38 (26·94 to 30·12)	20·07 (16·88 to 24·38)	0·02 (−0·01 to 0·06)	0·03 (0·02 to 0·04)	0·01 (0·00 to 0·01)
	Eastern Europe		73·10 (48·14 to 117·64)	864·89 (547·01 to 1413·14)	26·09 (24·67 to 27·65)	18·69 (15·34 to 23·40)	0·02 (−0·01 to 0·06)	0·03 (0·02 to 0·05)	0·01 (0·01 to 0·02)
		Belarus	1·37 (0·76 to 2·29)	17·50 (8·74 to 29·52)	0·59 (0·41 to 0·96)	35·42 (27·69 to 46·87)	0·01 (−0·03 to 0·05)	0·04 (0·02 to 0·06)	0·02 (−0·00 to 0·05)
		Estonia	0·11 (0·06 to 0·19)	1·62 (0·81 to 2·93)	0·03 (0·03 to 0·04)	31·07 (25·55 to 36·79)	−0·02 (−0·04 to 0·00)	0·05 (0·03 to 0·06)	−0·01 (−0·02 to 0·01)
		Latvia	0·17 (0·05 to 0·35)	2·93 (1·42 to 5·82)	0·11 (0·10 to 0·12)	16·62 (11·92 to 23·32)	−0·05 (−0·16 to 0·00)	0·00 (−0·03 to 0·04)	0·04 (0·03 to 0·05)
		Lithuania	0·08 (0·01 to 0·17)	1·67 (0·83 to 3·17)	0·06 (0·06 to 0·07)	22·13 (16·73 to 29·50)	−0·06 (−0·21 to −0·01)	0·00 (−0·03 to 0·03)	0·01 (−0·01 to 0·02)
		Moldova	0·54 (0·31 to 0·92)	7·94 (3·67 to 14·59)	0·18 (0·16 to 0·21)	21·43 (15·09 to 30·21)	−0·01 (−0·03 to 0·01)	0·03 (0·02 to 0·04)	−0·03 (−0·04 to −0·02)
		Russia	57·34 (32·75 to 102·27)	607·05 (312·14 to 1107·70)	17·89 (16·58 to 19·33)	13·91 (10·90 to 17·43)	0·05 (0·01 to 0·10)	0·05 (0·03 to 0·06)	0·03 (0·02 to 0·04)
		Ukraine	13·49 (9·92 to 18·67)	226·16 (132·70 to 360·43)	7·22 (6·52 to 8·01)	28·19 (21·83 to 36·31)	−0·04 (−0·06 to −0·02)	0·01 (−0·00 to 0·01)	−0·01 (−0·03 to −0·00)
	Central Europe		1·19 (0·82 to 1·55)	19·79 (14·35 to 26·53)	0·42 (0·39 to 0·49)	46·47 (41·14 to 52·17)	0·00 (−0·03 to 0·02)	0·02 (0·00 to 0·03)	−0·04 (−0·04 to −0·02)
		Albania	0·00 (0·00 to 0·01)	0·07 (0·02 to 0·14)	0·00 (0·00 to 0·00)	46·34 (32·88 to 63·31)	−0·07 (−0·57 to 0·04)	0·00 (−0·04 to 0·04)	−0·00 (−0·04 to 0·04)
		Bosnia and Herzegovina	0·00 (0·00 to 0·01)	0·10 (0·03 to 0·21)	0·00 (0·00 to 0·01)	48·18 (35·41 to 63·19)	−0·06 (−0·50 to 0·04)	0·00 (−0·03 to 0·03)	−0·00 (−0·04 to 0·06)
		Bulgaria	0·14 (0·06 to 0·26)	1·86 (0·83 to 4·00)	0·05 (0·05 to 0·06)	17·02 (12·25 to 22·96)	−0·00 (−0·06 to 0·03)	0·01 (−0·02 to 0·03)	−0·05 (−0·06 to −0·03)
		Croatia	0·02 (0·00 to 0·02)	0·34 (0·16 to 0·60)	0·01 (0·01 to 0·01)	53·33 (43·15 to 65·99)	−0·02 (−0·12 to 0·01)	0·01 (−0·01 to 0·03)	0·01 (−0·00 to 0·02)
		Czech Republic	0·04 (0·01 to 0·07)	0·75 (0·37 to 1·21)	0·01 (0·01 to 0·02)	53·80 (46·23 to 62·90)	0·01 (−0·10 to 0·05)	0·02 (−0·00 to 0·04)	0·03 (0·01 to 0·04)
		Hungary	0·06 (0·04 to 0·08)	1·24 (0·66 to 1·99)	0·04 (0·04 to 0·05)	45·77 (37·00 to 56·61)	−0·02 (−0·05 to 0·00)	−0·02 (−0·03 to −0·00)	−0·08 (−0·09 to −0·06)
		Macedonia	0·01 (0·00 to 0·01)	0·09 (0·03 to 0·18)	0·00 (0·00 to 0·00)	40·71 (29·03 to 59·30)	−0·06 (−0·53 to 0·04)	0·03 (−0·02 to 0·07)	0·02 (−0·02 to 0·08)
		Montenegro	0·00 (0·00 to 0·01)	0·05 (0·02 to 0·10)	0·00 (0·00 to 0·00)	42·44 (30·59 to 60·43)	−0·04 (−0·53 to 0·07)	0·02 (−0·02 to 0·06)	0·01 (−0·03 to 0·07)
		Poland	0·41 (0·13 to 0·67)	7·71 (3·88 to 12·56)	0·14 (0·13 to 0·15)	56·60 (48·73 to 67·12)	−0·00 (−0·11 to 0·03)	0·01 (−0·01 to 0·03)	−0·02 (−0·03 to −0·01)
		Romania	0·45 (0·15 to 0·69)	6·33 (3·17 to 10·39)	0·09 (0·08 to 0·10)	43·39 (34·99 to 54·03)	0·03 (−0·07 to 0·06)	0·04 (0·01 to 0·06)	−0·06 (−0·07 to −0·05)
		Serbia	0·04 (0·02 to 0·08)	0·87 (0·38 to 2·21)	0·05 (0·03 to 0·12)	26·76 (20·80 to 32·60)	−0·08 (−0·11 to −0·05)	−0·00 (−0·02 to 0·01)	0·08 (0·03 to 0·13)
		Slovakia	0·02 (0·01 to 0·03)	0·24 (0·11 to 0·42)	0·01 (0·00 to 0·01)	46·16 (36·95 to 55·76)	0·02 (0·00 to 0·04)	0·04 (0·01 to 0·06)	0·00 (−0·03 to 0·02)
		Slovenia	0·01 (0·00 to 0·01)	0·14 (0·06 to 0·27)	0·00 (0·00 to 0·00)	58·71 (45·13 to 71·99)	0·02 (−0·01 to 0·04)	−0·00 (−0·02 to 0·02)	−0·07 (−0·08 to −0·06)
	Central Asia		3·96 (2·64 to 5·58)	56·19 (39·49 to 79·69)	1·87 (1·52 to 2·57)	30·50 (25·59 to 36·68)	−0·01 (−0·05 to 0·03)	0·01 (−0·01 to 0·02)	−0·03 (−0·05 to −0·02)
		Armenia	0·07 (0·03 to 0·13)	0·57 (0·29 to 1·04)	0·02 (0·01 to 0·02)	21·59 (17·29 to 27·60)	0·07 (−0·03 to 0·26)	0·07 (0·02 to 0·12)	0·05 (0·00 to 0·09)
		Azerbaijan	0·36 (0·17 to 0·58)	4·06 (1·89 to 7·45)	0·11 (0·07 to 0·21)	32·83 (23·57 to 47·48)	0·04 (−0·07 to 0·09)	0·02 (−0·01 to 0·05)	−0·06 (−0·09 to −0·02)
		Georgia	0·15 (0·08 to 0·25)	1·70 (0·98 to 2·56)	0·03 (0·03 to 0·04)	38·75 (33·41 to 44·83)	0·04 (−0·01 to 0·09)	0·13 (0·10 to 0·15)	0·12 (0·09 to 0·15)
		Kazakhstan	1·63 (0·88 to 2·64)	17·70 (8·32 to 31·95)	0·31 (0·27 to 0·36)	24·79 (19·36 to 32·88)	0·09 (0·06 to 0·14)	0·04 (0·02 to 0·05)	−0·05 (−0·07 to −0·04)
		Kyrgyzstan	0·32 (0·16 to 0·62)	6·62 (3·03 to 13·08)	0·30 (0·21 to 0·46)	32·24 (26·28 to 38·78)	−0·07 (−0·14 to −0·01)	0·03 (0·01 to 0·05)	0·02 (0·01 to 0·04)
		Mongolia	0·01 (0·00 to 0·01)	0·08 (0·03 to 0·17)	0·00 (0·00 to 0·01)	26·46 (17·72 to 41·13)	0·03 (−0·12 to 0·18)	−0·01 (−0·06 to 0·02)	−0·06 (−0·09 to −0·03)
		Tajikistan	0·32 (0·14 to 0·61)	4·64 (2·20 to 8·86)	0·18 (0·13 to 0·30)	27·27 (20·60 to 36·20)	−0·03 (−0·12 to 0·06)	−0·01 (−0·03 to 0·02)	−0·05 (−0·08 to −0·02)
		Turkmenistan	0·79 (0·10 to 1·96)	9·22 (3·17 to 19·29)	0·35 (0·22 to 0·56)	21·76 (13·98 to 36·05)	0·01 (−0·18 to 0·16)	0·02 (−0·05 to 0·07)	−0·01 (−0·05 to 0·04)
		Uzbekistan	0·31 (0·10 to 0·68)	11·59 (5·53 to 26·29)	0·57 (0·35 to 1·16)	40·92 (29·74 to 52·77)	−0·17 (−0·29 to −0·08)	−0·05 (−0·06 to −0·03)	−0·06 (−0·09 to −0·02)
Latin America and Caribbean			85·47 (77·62 to 94·22)	1322·07 (1194·38 to 1474·60)	46·81 (43·27 to 50·98)	45·10 (43·68 to 46·49)	−0·00 (−0·01 to 0·00)	0·01 (0·00 to 0·01)	−0·02 (−0·03 to −0·02)
	Central Latin America		29·38 (24·91 to 34·23)	394·06 (328·88 to 465·79)	12·31 (12·01 to 12·71)	40·01 (38·29 to 41·84)	0·01 (0·00 to 0·03)	0·02 (0·02 to 0·03)	−0·02 (−0·02 to −0·02)
		Colombia	6·15 (3·42 to 10·00)	73·95 (36·96 to 131·07)	2·42 (2·30 to 2·56)	29·75 (24·34 to 36·98)	0·03 (0·00 to 0·07)	0·02 (0·01 to 0·04)	−0·02 (−0·03 to −0·02)
		Costa Rica	0·35 (0·22 to 0·50)	6·66 (3·38 to 10·89)	0·15 (0·14 to 0·16)	50·08 (43·35 to 56·40)	−0·03 (−0·05 to −0·02)	0·01 (0·01 to 0·02)	−0·02 (−0·03 to −0·01)
		El Salvador	0·80 (0·47 to 1·21)	16·11 (8·09 to 27·60)	0·55 (0·42 to 0·77)	46·22 (40·88 to 50·96)	−0·05 (−0·07 to −0·03)	0·01 (−0·00 to 0·02)	0·02 (−0·01 to 0·04)
		Guatemala	1·67 (0·84 to 2·96)	27·74 (12·99 to 49·27)	0·68 (0·65 to 0·71)	42·04 (36·62 to 47·33)	−0·03 (−0·10 to 0·03)	−0·00 (−0·02 to 0·02)	−0·06 (−0·06 to −0·05)
		Honduras	1·48 (0·88 to 2·29)	19·82 (12·09 to 30·07)	0·59 (0·50 to 0·73)	40·41 (35·61 to 45·45)	0·01 (−0·04 to 0·05)	0·02 (−0·01 to 0·04)	−0·03 (−0·06 to 0·00)
		Mexico	12·47 (11·23 to 13·83)	169·52 (147·48 to 194·76)	5·17 (5·11 to 5·24)	45·67 (43·88 to 47·67)	0·01 (0·00 to 0·02)	0·03 (0·02 to 0·03)	−0·02 (−0·03 to −0·02)
		Nicaragua	0·97 (0·49 to 1·68)	7·93 (4·03 to 13·74)	0·19 (0·16 to 0·25)	22·22 (19·37 to 25·34)	0·09 (0·04 to 0·14)	0·09 (0·06 to 0·11)	0·02 (−0·00 to 0·05)
		Panama	1·80 (0·96 to 3·16)	18·92 (9·91 to 31·50)	0·51 (0·38 to 0·75)	38·52 (33·38 to 43·53)	0·12 (0·05 to 0·25)	0·02 (0·01 to 0·04)	−0·02 (−0·05 to 0·01)
		Venezuela	3·68 (1·64 to 6·45)	53·41 (27·48 to 95·77)	2·04 (1·94 to 2·15)	33·10 (28·61 to 38·45)	−0·01 (−0·10 to 0·03)	0·02 (−0·00 to 0·04)	0·01 (0·00 to 0·02)
	Andean Latin America		3·83 (2·68 to 5·33)	49·31 (32·11 to 71·86)	1·81 (1·54 to 2·20)	34·59 (31·27 to 38·93)	0·01 (−0·01 to 0·04)	0·02 (0·01 to 0·04)	−0·01 (−0·03 to 0·00)
		Bolivia	0·19 (0·10 to 0·31)	2·23 (1·01 to 4·17)	0·10 (0·07 to 0·14)	18·69 (15·60 to 21·98)	0·02 (−0·02 to 0·07)	0·02 (−0·00 to 0·04)	−0·01 (−0·03 to 0·02)
		Ecuador	2·02 (1·14 to 3·19)	23·39 (12·33 to 39·59)	0·79 (0·64 to 1·09)	34·40 (30·49 to 38·80)	0·02 (−0·01 to 0·06)	0·04 (0·01 to 0·06)	0·01 (−0·02 to 0·03)
		Peru	1·62 (0·96 to 2·50)	23·70 (12·56 to 41·07)	0·92 (0·75 to 1·22)	35·94 (30·57 to 42·07)	0·00 (−0·03 to 0·03)	0·01 (−0·00 to 0·02)	−0·03 (−0·04 to −0·02)
	Caribbean		17·29 (12·72 to 23·36)	307·45 (272·69 to 342·05)	11·28 (9·73 to 12·89)	46·11 (42·49 to 49·62)	−0·02 (−0·04 to 0·01)	−0·01 (−0·02 to 0·00)	−0·07 (−0·08 to −0·06)
		Antigua and Barbuda	0·02 (0·01 to 0·04)	0·34 (0·14 to 0·71)	0·01 (0·01 to 0·01)	39·68 (29·72 to 49·29)	0·01 (−0·07 to 0·14)	0·00 (−0·02 to 0·05)	−0·03 (−0·04 to −0·03)
		The Bahamas	0·11 (0·07 to 0·18)	3·33 (1·75 to 5·39)	0·11 (0·07 to 0·17)	51·08 (42·61 to 61·04)	−0·07 (−0·10 to −0·03)	−0·01 (−0·02 to 0·01)	−0·05 (−0·07 to −0·03)
		Barbados	0·06 (0·03 to 0·10)	1·07 (0·50 to 1·86)	0·03 (0·02 to 0·03)	46·43 (38·95 to 55·50)	0·02 (−0·02 to 0·05)	0·00 (−0·01 to 0·01)	−0·04 (−0·05 to −0·03)
		Belize	0·19 (0·12 to 0·28)	3·03 (1·58 to 5·21)	0·10 (0·06 to 0·17)	58·12 (53·15 to 63·65)	0·02 (−0·02 to 0·05)	0·00 (−0·01 to 0·01)	0·00 (−0·04 to 0·04)
		Bermuda	0·02 (0·01 to 0·04)	0·36 (0·15 to 0·77)	0·01 (0·01 to 0·01)	40·56 (30·34 to 50·33)	0·01 (−0·08 to 0·14)	−0·00 (−0·02 to 0·04)	−0·03 (−0·04 to −0·02)
		Cuba	1·14 (0·70 to 1·78)	18·71 (9·53 to 30·35)	0·32 (0·31 to 0·34)	62·31 (56·48 to 68·60)	−0·00 (−0·03 to 0·02)	0·09 (0·06 to 0·11)	0·07 (0·06 to 0·08)
		Dominica	0·01 (0·00 to 0·03)	0·20 (0·08 to 0·41)	0·01 (0·00 to 0·01)	37·59 (28·28 to 46·01)	0·01 (−0·08 to 0·16)	0·01 (−0·01 to 0·06)	−0·01 (−0·04 to 0·04)
		Dominican Republic	2·82 (2·03 to 3·84)	55·93 (48·99 to 62·58)	1·99 (1·34 to 2·55)	39·80 (35·00 to 45·75)	0·01 (−0·05 to 0·12)	−0·04 (−0·05 to −0·03)	−0·11 (−0·15 to −0·08)
		Grenada	0·02 (0·01 to 0·05)	0·33 (0·14 to 0·70)	0·01 (0·01 to 0·02)	33·58 (25·44 to 41·30)	0·00 (−0·08 to 0·13)	0·01 (−0·01 to 0·06)	−0·01 (−0·04 to 0·04)
		Guyana	1·14 (0·65 to 1·78)	18·89 (8·80 to 32·29)	0·47 (0·30 to 0·71)	62·25 (54·32 to 69·67)	−0·01 (−0·03 to 0·01)	0·02 (0·00 to 0·05)	0·02 (−0·01 to 0·05)
		Haiti	9·49 (5·37 to 15·10)	157·01 (132·94 to 183·68)	6·72 (5·38 to 8·10)	44·09 (38·75 to 49·87)	−0·04 (−0·08 to 0·01)	−0·02 (−0·03 to −0·00)	−0·09 (−0·10 to −0·07)
		Jamaica	0·66 (0·41 to 0·97)	11·08 (5·47 to 17·67)	0·42 (0·30 to 0·63)	39·55 (34·00 to 46·05)	−0·00 (−0·03 to 0·02)	−0·00 (−0·02 to 0·01)	−0·04 (−0·06 to −0·01)
		Puerto Rico	0·44 (0·17 to 1·10)	12·01 (4·45 to 28·56)	0·28 (0·26 to 0·30)	50·14 (35·87 to 63·33)	−0·01 (−0·10 to 0·14)	−0·03 (−0·05 to 0·01)	−0·07 (−0·08 to −0·06)
		Saint Lucia	0·02 (0·01 to 0·05)	0·39 (0·16 to 0·82)	0·01 (0·01 to 0·01)	37·89 (28·04 to 46·64)	0·01 (−0·08 to 0·15)	0·01 (−0·01 to 0·05)	−0·04 (−0·05 to −0·03)
		Saint Vincent and the Grenadines	0·05 (0·02 to 0·11)	0·82 (0·34 to 1·70)	0·02 (0·02 to 0·02)	36·75 (27·32 to 45·46)	0·01 (−0·08 to 0·15)	0·01 (−0·01 to 0·06)	−0·03 (−0·04 to −0·02)
		Suriname	0·19 (0·11 to 0·32)	3·81 (1·79 to 6·76)	0·14 (0·10 to 0·21)	44·05 (35·93 to 50·95)	−0·01 (−0·04 to 0·02)	−0·01 (−0·02 to 0·00)	0·00 (−0·03 to 0·03)
		Trinidad and Tobago	0·31 (0·19 to 0·50)	8·20 (3·80 to 15·61)	0·22 (0·20 to 0·23)	41·38 (33·87 to 48·39)	−0·05 (−0·07 to −0·03)	−0·01 (−0·02 to −0·00)	−0·05 (−0·05 to −0·04)
		US Virgin Islands	0·01 (0·01 to 0·03)	0·27 (0·11 to 0·55)	0·01 (0·01 to 0·02)	43·23 (31·95 to 53·07)	0·01 (−0·08 to 0·16)	0·01 (−0·01 to 0·06)	−0·01 (−0·05 to 0·04)
	Tropical Latin America		34·97 (31·13 to 38·94)	571·24 (468·17 to 701·82)	21·41 (18·29 to 25·43)	48·93 (47·39 to 50·56)	−0·01 (−0·01 to −0·00)	0·01 (0·00 to 0·01)	0·02 (0·01 to 0·03)
		Brazil	33·76 (30·24 to 37·52)	558·84 (454·38 to 687·40)	21·05 (17·92 to 25·13)	49·37 (47·89 to 50·94)	−0·01 (−0·01 to −0·00)	0·01 (0·00 to 0·01)	0·02 (0·01 to 0·02)
		Paraguay	1·21 (0·48 to 2·42)	12·40 (5·81 to 22·65)	0·36 (0·26 to 0·46)	29·48 (24·14 to 36·67)	0·02 (−0·04 to 0·06)	0·06 (0·03 to 0·08)	0·05 (0·01 to 0·07)
Southeast Asia, east Asia, and Oceania			174·31 (121·70 to 266·48)	2335·91 (1716·35 to 3511·41)	101·62 (77·06 to 158·16)	25·88 (20·76 to 32·27)	0·00 (−0·03 to 0·03)	0·04 (0·01 to 0·06)	0·04 (−0·01 to 0·07)
	East Asia		56·50 (41·02 to 77·70)	796·14 (591·71 to 1043·33)	42·74 (38·28 to 47·27)	17·90 (15·89 to 20·29)	−0·01 (−0·04 to 0·00)	0·04 (0·03 to 0·05)	0·08 (0·07 to 0·09)
		China	55·20 (39·82 to 75·98)	779·48 (573·77 to 1024·76)	41·82 (37·43 to 46·26)	17·90 (15·84 to 20·33)	−0·01 (−0·04 to 0·00)	0·04 (0·03 to 0·05)	0·08 (0·07 to 0·09)
		North Korea	0·91 (0·10 to 3·08)	11·16 (2·27 to 39·91)	0·62 (0·12 to 2·43)	17·20 (6·82 to 33·39)	0·01 (−0·14 to 0·11)	0·05 (−0·02 to 0·13)	0·08 (−0·01 to 0·17)
		Taiwan	0·39 (0·12 to 0·84)	5·51 (2·23 to 11·10)	0·29 (0·21 to 0·38)	17·08 (11·69 to 23·37)	−0·02 (−0·11 to 0·03)	0·04 (0·01 to 0·07)	0·09 (0·06 to 0·11)
	Southeast Asia		116·19 (66·38 to 204·40)	1510·91 (925·80 to 2625·43)	57·90 (34·09 to 115·40)	29·77 (21·90 to 38·69)	0·00 (−0·04 to 0·05)	0·03 (−0·01 to 0·06)	0·01 (−0·05 to 0·06)
		Cambodia	7·83 (3·55 to 14·42)	82·97 (32·84 to 153·51)	2·60 (1·80 to 3·64)	29·52 (22·70 to 36·25)	0·04 (0·00 to 0·07)	0·01 (−0·00 to 0·03)	0·01 (−0·02 to 0·03)
		Indonesia	43·39 (8·51 to 123·93)	440·51 (90·19 to 1391·78)	18·56 (3·60 to 68·98)	11·67 (8·08 to 15·97)	0·02 (−0·04 to 0·09)	0·10 (0·06 to 0·16)	0·17 (0·11 to 0·23)
		Laos	0·51 (0·12 to 1·58)	6·93 (1·68 to 23·47)	0·18 (0·04 to 0·69)	32·94 (23·92 to 42·06)	−0·04 (−0·09 to 0·05)	0·06 (0·02 to 0·11)	0·08 (0·02 to 0·15)
		Malaysia	2·04 (1·56 to 2·81)	39·53 (22·31 to 70·73)	2·29 (1·83 to 3·35)	29·22 (22·89 to 36·80)	−0·07 (−0·08 to −0·05)	−0·03 (−0·04 to −0·02)	−0·01 (−0·03 to 0·01)
		Maldives	0·00 (0·00 to 0·00)	0·02 (0·01 to 0·03)	0·00 (0·00 to 0·00)	10·72 (7·60 to 15·10)	−0·01 (−0·03 to 0·01)	−0·02 (−0·04 to 0·00)	−0·03 (−0·05 to −0·02)
		Mauritius	0·12 (0·08 to 0·19)	1·57 (0·94 to 2·56)	0·08 (0·07 to 0·09)	19·51 (15·45 to 23·79)	−0·00 (−0·03 to 0·02)	0·04 (0·03 to 0·06)	0·12 (0·11 to 0·14)
		Myanmar	6·75 (1·35 to 18·92)	177·74 (39·79 to 645·94)	8·62 (1·68 to 36·64)	40·35 (30·88 to 51·89)	−0·07 (−0·13 to 0·00)	−0·01 (−0·07 to 0·05)	−0·04 (−0·12 to 0·02)
		Philippines	33·31 (12·50 to 82·46)	273·65 (127·88 to 476·60)	3·55 (3·31 to 3·82)	32·57 (24·42 to 42·40)	0·09 (0·03 to 0·19)	0·09 (0·07 to 0·12)	−0·05 (−0·06 to −0·04)
		Sri Lanka	0·21 (0·10 to 0·38)	2·21 (1·00 to 3·95)	0·05 (0·05 to 0·06)	25·91 (23·17 to 28·86)	0·07 (0·03 to 0·11)	0·03 (0·01 to 0·05)	−0·02 (−0·03 to −0·01)
		Seychelles	0·01 (0·00 to 0·03)	0·14 (0·04 to 0·30)	0·01 (0·00 to 0·01)	29·89 (16·29 to 44·37)	−0·02 (−0·40 to 0·15)	0·02 (−0·04 to 0·10)	0·01 (−0·03 to 0·05)
		Thailand	10·06 (2·85 to 21·66)	288·25 (139·33 to 514·46)	14·74 (10·34 to 21·49)	40·71 (34·54 to 51·96)	−0·07 (−0·20 to 0·01)	−0·01 (−0·03 to 0·00)	−0·01 (−0·03 to 0·01)
		Timor-Leste	0·10 (0·00 to 0·44)	1·46 (0·03 to 6·86)	0·07 (0·00 to 0·37)	25·29 (9·12 to 49·66)	0·01 (−0·40 to 0·27)	0·05 (−0·07 to 0·20)	0·03 (−0·08 to 0·17)
		Vietnam	11·73 (2·48 to 33·85)	193·97 (38·99 to 718·95)	7·05 (1·39 to 29·18)	33·48 (23·62 to 44·85)	−0·03 (−0·09 to 0·05)	0·03 (−0·01 to 0·08)	0·02 (−0·03 to 0·08)
	Oceania		1·62 (1·13 to 2·28)	28·85 (24·68 to 33·04)	0·99 (0·78 to 1·27)	52·65 (46·71 to 59·02)	−0·05 (−0·08 to −0·01)	0·02 (−0·00 to 0·04)	−0·04 (−0·06 to −0·02)
		American Samoa	0·00 (0·00 to 0·00)	0·02 (0·01 to 0·04)	0·00 (0·00 to 0·00)	28·49 (22·61 to 36·97)	0·04 (−0·06 to 0·09)	0·05 (0·01 to 0·08)	0·00 (−0·02 to 0·03)
		Federated States of Micronesia	0·01 (0·00 to 0·03)	0·11 (0·02 to 0·42)	0·00 (0·00 to 0·02)	24·74 (14·47 to 45·45)	0·07 (−0·10 to 0·20)	0·07 (−0·03 to 0·18)	0·02 (−0·08 to 0·13)
		Fiji	0·07 (0·04 to 0·13)	0·68 (0·32 to 1·24)	0·02 (0·02 to 0·03)	24·73 (21·67 to 28·32)	0·05 (0·03 to 0·07)	0·05 (0·04 to 0·06)	0·04 (0·02 to 0·06)
		Guam	0·01 (0·00 to 0·03)	0·14 (0·06 to 0·28)	0·00 (0·00 to 0·01)	30·04 (22·88 to 40·09)	0·04 (−0·05 to 0·09)	0·05 (0·01 to 0·08)	−0·00 (−0·03 to 0·03)
		Kiribati	0·00 (0·00 to 0·00)	0·02 (0·01 to 0·04)	0·00 (0·00 to 0·00)	29·00 (21·80 to 38·81)	0·03 (−0·06 to 0·08)	0·03 (−0·01 to 0·06)	−0·02 (−0·04 to 0·01)
		Marshall Islands	0·01 (0·00 to 0·02)	0·09 (0·01 to 0·39)	0·00 (0·00 to 0·02)	25·91 (15·20 to 47·44)	0·07 (−0·10 to 0·19)	0·07 (−0·05 to 0·19)	0·02 (−0·08 to 0·14)
		Northern Mariana Islands	0·01 (0·00 to 0·01)	0·05 (0·02 to 0·10)	0·00 (0·00 to 0·00)	24·00 (19·14 to 32·23)	0·04 (−0·06 to 0·08)	0·05 (0·01 to 0·08)	0·02 (−0·01 to 0·04)
		Papua New Guinea	1·31 (0·86 to 1·94)	24·57 (21·27 to 27·37)	0·84 (0·66 to 1·04)	54·46 (48·01 to 61·46)	−0·06 (−0·10 to −0·02)	0·02 (−0·01 to 0·03)	−0·05 (−0·07 to −0·02)
		Samoa	0·02 (0·00 to 0·05)	0·21 (0·03 to 0·92)	0·01 (0·00 to 0·04)	27·11 (16·02 to 47·08)	0·07 (−0·10 to 0·20)	0·07 (−0·04 to 0·18)	0·02 (−0·07 to 0·13)
		Solomon Islands	0·05 (0·01 to 0·16)	0·64 (0·08 to 2·87)	0·02 (0·00 to 0·15)	25·34 (15·33 to 44·47)	0·08 (−0·10 to 0·20)	0·07 (−0·04 to 0·18)	0·02 (−0·08 to 0·13)
		Tonga	0·01 (0·00 to 0·02)	0·06 (0·02 to 0·11)	0·00 (0·00 to 0·00)	22·65 (18·24 to 30·85)	0·08 (−0·02 to 0·14)	0·10 (0·05 to 0·14)	0·06 (0·02 to 0·09)
		Vanuatu	0·02 (0·00 to 0·08)	0·31 (0·04 to 1·28)	0·01 (0·00 to 0·07)	25·63 (15·26 to 45·39)	0·07 (−0·09 to 0·20)	0·07 (−0·05 to 0·19)	0·02 (−0·08 to 0·14)
North Africa and Middle East			12·39 (8·53 to 17·51)	137·94 (113·08 to 172·80)	7·54 (6·25 to 9·30)	19·07 (15·88 to 22·65)	−0·02 (−0·05 to 0·01)	0·02 (−0·01 to 0·03)	0·01 (−0·01 to 0·03)
		Afghanistan	0·70 (0·14 to 2·04)	4·50 (0·92 to 13·98)	0·21 (0·04 to 0·73)	4·17 (3·08 to 5·86)	0·09 (0·01 to 0·17)	0·06 (−0·05 to 0·14)	0·02 (−0·11 to 0·12)
		Algeria	0·31 (0·01 to 0·95)	6·47 (2·15 to 11·52)	0·23 (0·12 to 0·38)	61·55 (54·76 to 68·03)	−0·13 (−0·48 to 0·04)	0·04 (−0·02 to 0·06)	0·01 (−0·04 to 0·05)
		Bahrain	0·06 (0·01 to 0·12)	0·53 (0·23 to 1·02)	0·02 (0·01 to 0·03)	17·69 (13·63 to 23·05)	0·04 (−0·08 to 0·11)	0·03 (−0·01 to 0·07)	−0·01 (−0·04 to 0·02)
		Egypt	0·95 (0·48 to 1·65)	6·80 (3·37 to 11·62)	0·21 (0·17 to 0·26)	17·68 (15·09 to 20·70)	0·09 (0·06 to 0·13)	0·09 (0·07 to 0·11)	0·03 (0·02 to 0·05)
		Iran	1·13 (0·62 to 2·06)	11·49 (5·78 to 21·26)	0·55 (0·43 to 0·76)	15·31 (13·65 to 17·17)	0·03 (−0·02 to 0·07)	0·03 (0·01 to 0·05)	0·04 (0·01 to 0·08)
		Iraq	0·51 (0·13 to 1·05)	3·63 (1·66 to 6·87)	0·11 (0·08 to 0·15)	16·20 (12·70 to 21·54)	0·07 (−0·07 to 0·14)	0·09 (0·04 to 0·13)	0·06 (0·03 to 0·10)
		Jordan	0·03 (0·01 to 0·06)	0·28 (0·12 to 0·56)	0·01 (0·01 to 0·02)	20·24 (15·73 to 26·00)	0·02 (−0·12 to 0·09)	0·02 (−0·01 to 0·06)	−0·00 (−0·03 to 0·03)
		Kuwait	0·01 (0·00 to 0·03)	0·14 (0·06 to 0·28)	0·01 (0·01 to 0·01)	19·37 (15·23 to 25·32)	0·03 (−0·11 to 0·09)	0·01 (−0·03 to 0·04)	−0·10 (−0·11 to −0·08)
		Lebanon	0·13 (0·02 to 0·38)	1·94 (0·41 to 7·96)	0·09 (0·02 to 0·45)	35·17 (22·65 to 62·67)	0·02 (−0·04 to 0·09)	0·01 (−0·06 to 0·08)	0·01 (−0·07 to 0·06)
		Libya	0·23 (0·01 to 0·94)	2·43 (0·15 to 10·48)	0·11 (0·00 to 0·55)	19·73 (13·40 to 28·26)	0·04 (−0·14 to 0·16)	0·05 (−0·06 to 0·16)	0·02 (−0·09 to 0·13)
		Morocco	0·67 (0·39 to 1·06)	8·62 (4·20 to 15·19)	0·36 (0·27 to 0·47)	24·58 (22·74 to 26·56)	−0·01 (−0·03 to 0·02)	0·04 (0·02 to 0·05)	0·08 (0·04 to 0·11)
		Oman	0·14 (0·09 to 0·19)	1·83 (0·97 to 2·95)	0·07 (0·05 to 0·09)	33·16 (27·89 to 39·27)	−0·02 (−0·05 to 0·01)	0·01 (−0·01 to 0·02)	0·04 (−0·01 to 0·07)
		Palestine	0·06 (0·02 to 0·12)	0·45 (0·21 to 0·90)	0·02 (0·01 to 0·02)	17·23 (13·38 to 22·07)	0·03 (−0·08 to 0·10)	0·06 (0·02 to 0·10)	0·04 (0·01 to 0·08)
		Qatar	0·01 (0·00 to 0·03)	0·14 (0·06 to 0·27)	0·01 (0·00 to 0·01)	17·24 (13·30 to 22·43)	0·02 (−0·11 to 0·08)	−0·03 (−0·06 to 0·00)	−0·07 (−0·09 to −0·03)
		Saudi Arabia	1·06 (0·50 to 2·04)	11·58 (5·82 to 25·18)	0·49 (0·25 to 1·34)	23·37 (19·48 to 28·20)	0·02 (−0·05 to 0·08)	0·03 (−0·03 to 0·07)	0·01 (−0·06 to 0·05)
		Sudan	4·31 (1·25 to 8·66)	55·38 (41·64 to 70·69)	4·32 (3·20 to 5·31)	10·02 (7·44 to 13·08)	−0·09 (−0·20 to −0·02)	−0·01 (−0·05 to 0·02)	0·01 (−0·02 to 0·04)
		Syria	0·04 (0·01 to 0·07)	0·66 (0·15 to 1·51)	0·03 (0·01 to 0·05)	18·22 (15·12 to 22·01)	0·10 (−0·02 to 0·14)	0·11 (0·01 to 0·17)	0·06 (−0·02 to 0·11)
		Tunisia	0·28 (0·13 to 0·53)	2·62 (1·16 to 4·69)	0·09 (0·07 to 0·12)	23·64 (20·75 to 27·28)	0·05 (0·00 to 0·09)	0·07 (0·05 to 0·09)	0·10 (0·07 to 0·13)
		Turkey	0·72 (0·28 to 1·28)	8·07 (3·64 to 13·75)	0·19 (0·14 to 0·24)	32·60 (26·92 to 39·90)	0·01 (−0·06 to 0·05)	0·08 (0·04 to 0·10)	0·02 (−0·01 to 0·05)
		United Arab Emirates	0·54 (0·03 to 2·02)	5·69 (0·34 to 25·28)	0·27 (0·01 to 1·35)	16·98 (12·17 to 23·71)	0·04 (−0·15 to 0·16)	0·05 (−0·06 to 0·16)	0·02 (−0·09 to 0·13)
		Yemen	0·51 (0·10 to 1·46)	4·52 (1·02 to 14·33)	0·16 (0·03 to 0·60)	35·75 (28·14 to 45·07)	0·04 (−0·04 to 0·12)	0·04 (−0·05 to 0·12)	−0·02 (−0·15 to 0·09)
South Asia			206·83 (171·79 to 249·70)	2966·01 (2767·85 to 3183·75)	135·26 (127·05 to 144·88)	25·55 (23·78 to 27·11)	−0·01 (−0·02 to 0·01)	−0·02 (−0·03 to −0·01)	−0·06 (−0·07 to −0·05)
		Bangladesh	0·51 (0·11 to 1·54)	6·69 (1·44 to 22·77)	0·27 (0·05 to 1·11)	15·57 (12·24 to 19·89)	−0·02 (−0·07 to 0·06)	0·09 (0·04 to 0·14)	0·13 (0·07 to 0·19)
		Bhutan	0·06 (0·01 to 0·18)	0·64 (0·14 to 2·16)	0·02 (0·00 to 0·06)	28·44 (18·86 to 44·60)	0·02 (−0·05 to 0·09)	0·04 (−0·04 to 0·12)	−0·04 (−0·17 to 0·07)
		India	196·60 (164·77 to 237·89)	2881·13 (2702·40 to 3078·82)	131·56 (124·14 to 138·94)	25·82 (24·18 to 27·36)	−0·01 (−0·03 to 0·01)	−0·02 (−0·03 to −0·02)	−0·06 (−0·07 to −0·05)
		Nepal	1·11 (0·23 to 3·34)	31·55 (6·90 to 118·68)	1·92 (0·37 to 8·04)	31·21 (24·54 to 39·54)	−0·13 (−0·19 to −0·06)	−0·01 (−0·05 to 0·03)	0·03 (−0·03 to 0·09)
		Pakistan	8·55 (1·66 to 25·89)	45·99 (9·66 to 139·53)	1·48 (0·25 to 5·20)	5·87 (4·00 to 8·40)	0·15 (0·07 to 0·24)	0·15 (0·06 to 0·24)	0·14 (0·01 to 0·24)
Sub-Saharan Africa			1847·99 (1656·94 to 2051·52)	29 439·54 (28 678·60 to 30 195·25)	859·00 (804·61 to 912·93)	42·35 (41·05 to 43·58)	−0·03 (−0·05 to −0·02)	−0·00 (−0·00 to 0·00)	−0·08 (−0·09 to −0·08)
	Southern sub–Saharan Africa		710·08 (604·65 to 836·17)	11 408·43 (10 926·35 to 11 882·56)	228·94 (211·90 to 250·82)	51·04 (48·91 to 53·20)	−0·03 (−0·04 to −0·01)	0·01 (0·01 to 0·01)	−0·09 (−0·09 to −0·08)
		Botswana	23·51 (14·49 to 33·86)	431·89 (394·30 to 473·07)	8·07 (5·00 to 10·58)	61·72 (54·17 to 69·76)	−0·01 (−0·06 to 0·04)	0·01 (0·00 to 0·01)	−0·08 (−0·11 to −0·04)
		Lesotho	24·75 (17·77 to 34·10)	354·36 (323·40 to 389·73)	12·57 (9·82 to 16·14)	36·43 (32·75 to 40·32)	−0·02 (−0·05 to 0·01)	0·02 (0·01 to 0·02)	−0·04 (−0·05 to −0·02)
		Namibia	14·05 (10·04 to 18·47)	253·41 (238·00 to 268·89)	5·09 (3·73 to 6·50)	51·55 (46·41 to 57·20)	−0·03 (−0·06 to 0·00)	0·01 (0·01 to 0·01)	−0·09 (−0·11 to −0·07)
		South Africa	529·67 (440·94 to 630·39)	8409·55 (7978·87 to 8850·42)	155·19 (140·96 to 172·68)	50·95 (48·45 to 53·56)	−0·03 (−0·05 to −0·01)	0·02 (0·02 to 0·02)	−0·08 (−0·08 to −0·06)
		Swaziland	13·91 (9·02 to 18·32)	263·04 (244·42 to 280·64)	5·89 (4·58 to 7·59)	52·50 (46·87 to 58·25)	−0·06 (−0·10 to −0·03)	0·02 (0·01 to 0·02)	−0·07 (−0·09 to −0·05)
		Zimbabwe	104·20 (53·05 to 173·34)	1696·17 (1543·57 to 1878·76)	42·12 (34·03 to 52·38)	51·64 (45·42 to 58·51)	−0·01 (−0·08 to 0·06)	−0·01 (−0·02 to −0·01)	−0·13 (−0·15 to −0·10)
	Western sub-Saharan Africa		444·71 (334·29 to 571·29)	6417·10 (6036·15 to 6873·38)	249·30 (212·45 to 288·59)	29·09 (26·33 to 31·90)	−0·03 (−0·06 to −0·00)	−0·00 (−0·01 to 0·01)	−0·05 (−0·07 to −0·04)
		Benin	5·40 (3·57 to 8·07)	83·05 (72·48 to 94·41)	2·36 (1·71 to 3·17)	43·94 (39·15 to 49·42)	−0·02 (−0·07 to 0·03)	−0·00 (−0·01 to 0·01)	−0·10 (−0·13 to −0·08)
		Burkina Faso	6·02 (3·20 to 9·53)	101·91 (85·21 to 121·99)	3·19 (2·44 to 3·93)	51·64 (45·07 to 59·57)	−0·01 (−0·08 to 0·05)	−0·03 (−0·05 to −0·02)	−0·16 (−0·18 to −0·14)
		Cameroon	48·59 (28·44 to 73·57)	659·83 (570·86 to 754·45)	33·19 (23·70 to 42·93)	21·55 (18·54 to 25·09)	−0·03 (−0·08 to 0·01)	−0·00 (−0·02 to 0·01)	−0·02 (−0·04 to −0·01)
		Cape Verde	0·32 (0·14 to 0·84)	3·83 (2·92 to 5·18)	0·10 (0·07 to 0·14)	32·10 (22·93 to 41·72)	−0·01 (−0·07 to 0·13)	0·03 (0·00 to 0·07)	−0·04 (−0·07 to −0·01)
		Chad	9·24 (3·70 to 17·19)	166·86 (127·28 to 214·51)	8·92 (5·99 to 11·87)	33·57 (25·61 to 43·33)	−0·09 (−0·19 to −0·02)	−0·02 (−0·04 to −0·00)	−0·06 (−0·08 to −0·03)
		Côte d'Ivoire	41·71 (24·65 to 64·08)	547·27 (464·67 to 633·88)	22·38 (17·99 to 28·08)	29·06 (23·81 to 34·68)	−0·02 (−0·06 to 0·03)	−0·01 (−0·03 to 0·00)	−0·07 (−0·09 to −0·06)
		The Gambia	0·91 (0·35 to 1·68)	17·82 (14·35 to 22·44)	0·66 (0·43 to 0·95)	22·24 (17·69 to 27·59)	−0·10 (−0·21 to −0·03)	0·01 (−0·01 to 0·03)	−0·01 (−0·03 to 0·01)
		Ghana	17·30 (9·85 to 26·44)	282·24 (238·35 to 330·83)	11·25 (8·22 to 14·98)	37·00 (30·63 to 44·34)	−0·04 (−0·09 to 0·01)	−0·03 (−0·04 to −0·02)	−0·09 (−0·12 to −0·07)
		Guinea	8·68 (3·79 to 14·06)	133·76 (109·08 to 158·21)	5·15 (4·06 to 6·69)	25·87 (21·95 to 30·18)	−0·05 (−0·12 to 0·00)	0·01 (−0·02 to 0·03)	−0·03 (−0·05 to −0·02)
		Guinea-Bissau	1·91 (0·69 to 3·61)	41·33 (34·66 to 48·64)	1·76 (0·98 to 2·56)	25·34 (22·37 to 28·67)	−0·11 (−0·21 to −0·03)	0·02 (0·01 to 0·04)	0·02 (−0·02 to 0·07)
		Liberia	2·65 (1·23 to 4·58)	36·97 (31·57 to 44·16)	2·17 (1·74 to 2·64)	22·36 (18·09 to 26·50)	−0·02 (−0·10 to 0·07)	−0·04 (−0·06 to −0·02)	−0·07 (−0·09 to −0·05)
		Mali	11·43 (5·99 to 17·86)	148·12 (111·67 to 187·99)	6·74 (4·96 to 9·08)	23·07 (19·78 to 26·82)	−0·03 (−0·09 to 0·00)	−0·00 (−0·02 to 0·02)	−0·03 (−0·06 to −0·01)
		Mauritania	0·12 (0·01 to 0·42)	7·01 (1·37 to 29·75)	0·36 (0·06 to 1·70)	40·28 (26·39 to 55·32)	−0·19 (−0·33 to −0·07)	−0·04 (−0·07 to 0·00)	−0·05 (−0·10 to −0·00)
		Niger	2·47 (0·79 to 4·96)	64·26 (53·92 to 76·75)	3·46 (2·94 to 4·06)	34·12 (28·38 to 40·03)	−0·11 (−0·23 to −0·03)	−0·06 (−0·08 to −0·04)	−0·09 (−0·11 to −0·07)
		Nigeria	274·19 (168·30 to 396·73)	3874·25 (3517·41 to 4307·34)	136·42 (100·74 to 174·02)	28·98 (24·68 to 33·55)	−0·02 (−0·07 to 0·01)	0·01 (−0·00 to 0·02)	−0·04 (−0·07 to −0·01)
		São Tomé and Príncipe	0·00 (0·00 to 0·00)	0·03 (0·02 to 0·04)	0·00 (0·00 to 0·00)	54·78 (48·49 to 61·57)	−0·01 (−0·07 to 0·04)	0·03 (0·01 to 0·05)	−0·06 (−0·09 to −0·02)
		Senegal	4·43 (1·36 to 7·44)	66·44 (52·59 to 81·28)	2·36 (1·70 to 3·01)	47·05 (40·37 to 55·06)	−0·05 (−0·16 to −0·00)	−0·00 (−0·02 to 0·01)	−0·05 (−0·09 to −0·02)
		Sierra Leone	4·13 (1·23 to 7·09)	61·20 (50·35 to 72·68)	2·79 (2·11 to 3·76)	18·47 (14·21 to 23·83)	−0·06 (−0·18 to 0·00)	0·01 (−0·02 to 0·03)	−0·00 (−0·02 to 0·02)
		Togo	5·20 (2·59 to 9·20)	120·86 (105·83 to 138·66)	6·05 (4·77 to 7·59)	29·99 (25·43 to 34·83)	−0·09 (−0·17 to −0·02)	−0·04 (−0·05 to −0·02)	−0·06 (−0·07 to −0·05)
	Eastern sub-Saharan Africa		618·52 (527·49 to 714·55)	10 437·57 (10 025·86 to 10 889·54)	318·68 (293·79 to 347·87)	42·82 (40·73 to 44·74)	−0·04 (−0·05 to −0·02)	−0·01 (−0·01 to −0·00)	−0·10 (−0·11 to −0·09)
		Burundi	6·65 (3·45 to 11·37)	111·53 (94·00 to 130·90)	3·68 (2·79 to 4·79)	37·92 (31·85 to 44·95)	−0·02 (−0·09 to 0·06)	−0·03 (−0·04 to −0·01)	−0·12 (−0·14 to −0·10)
		Comoros	0·06 (0·01 to 0·19)	0·41 (0·09 to 1·31)	0·02 (0·00 to 0·08)	12·81 (7·27 to 20·97)	0·02 (−0·09 to 0·13)	0·01 (−0·06 to 0·08)	0·03 (−0·05 to 0·11)
		Djibouti	0·51 (0·18 to 0·99)	7·37 (5·10 to 10·70)	0·38 (0·25 to 0·52)	24·81 (20·41 to 29·94)	−0·02 (−0·10 to 0·05)	−0·02 (−0·05 to −0·00)	−0·06 (−0·10 to −0·02)
		Eritrea	1·49 (0·68 to 2·51)	21·22 (16·16 to 28·23)	0·80 (0·52 to 1·21)	36·89 (26·24 to 49·02)	−0·00 (−0·08 to 0·07)	−0·02 (−0·04 to 0·00)	−0·09 (−0·13 to −0·05)
		Ethiopia	39·14 (19·59 to 62·13)	768·04 (651·03 to 904·91)	28·65 (22·04 to 34·85)	51·92 (45·82 to 59·09)	0·01 (−0·10 to 0·11)	−0·05 (−0·06 to −0·04)	−0·16 (−0·19 to −0·13)
		Kenya	137·20 (112·46 to 166·10)	1883·96 (1787·64 to 1987·73)	51·70 (48·19 to 55·64)	38·60 (36·42 to 40·72)	0·06 (0·04 to 0·08)	−0·01 (−0·02 to −0·01)	−0·13 (−0·13 to −0·12)
		Madagascar	2·00 (0·33 to 6·34)	42·51 (8·58 to 169·61)	4·42 (0·76 to 19·79)	1·35 (0·76 to 2·72)	−0·13 (−0·22 to −0·03)	−0·05 (−0·14 to 0·01)	−0·03 (−0·11 to 0·04)
		Malawi	55·62 (25·29 to 80·90)	1126·77 (988·92 to 1234·61)	28·41 (22·67 to 35·54)	49·58 (42·68 to 58·00)	−0·07 (−0·15 to −0·02)	−0·01 (−0·03 to −0·00)	−0·13 (−0·14 to −0·10)
		Mozambique	122·32 (74·39 to 176·33)	1833·02 (1593·99 to 2073·78)	70·06 (58·14 to 83·59)	30·66 (26·49 to 35·28)	−0·06 (−0·10 to −0·02)	0·01 (0·00 to 0·02)	−0·02 (−0·04 to −0·00)
		Rwanda	8·02 (4·46 to 12·17)	202·70 (180·73 to 224·59)	4·52 (3·29 to 5·88)	58·05 (51·98 to 64·37)	−0·08 (−0·14 to −0·03)	−0·01 (−0·02 to −0·00)	−0·15 (−0·18 to −0·13)
		Somalia	1·69 (0·73 to 3·13)	24·24 (16·88 to 33·77)	1·58 (1·17 to 2·09)	8·65 (5·53 to 13·34)	−0·08 (−0·18 to −0·01)	−0·02 (−0·06 to 0·01)	−0·01 (−0·04 to 0·02)
		South Sudan	9·76 (3·69 to 16·82)	122·52 (79·09 to 174·43)	8·93 (4·58 to 12·48)	8·59 (5·58 to 13·19)	−0·05 (−0·14 to 0·01)	−0·01 (−0·05 to 0·03)	−0·01 (−0·05 to 0·05)
		Tanzania	86·66 (51·18 to 135·07)	1494·12 (1315·85 to 1672·30)	47·86 (34·24 to 61·09)	47·58 (41·18 to 54·48)	−0·04 (−0·08 to 0·00)	−0·01 (−0·03 to −0·00)	−0·11 (−0·14 to −0·09)
		Uganda	77·87 (33·96 to 126·32)	1491·60 (1310·27 to 1708·04)	36·32 (27·59 to 51·43)	43·44 (38·37 to 48·94)	−0·07 (−0·15 to −0·01)	0·02 (0·00 to 0·03)	−0·09 (−0·11 to −0·06)
		Zambia	69·14 (50·55 to 88·86)	1300·28 (1215·65 to 1382·85)	31·12 (26·20 to 36·90)	52·70 (47·08 to 58·71)	−0·06 (−0·10 to −0·03)	0·01 (0·00 to 0·01)	−0·11 (−0·13 to −0·10)
	Central sub-Saharan Africa		74·67 (46·65 to 121·85)	1176·44 (1054·44 to 1312·53)	62·08 (54·72 to 70·25)	26·41 (23·29 to 29·65)	−0·07 (−0·12 to −0·02)	−0·03 (−0·04 to −0·02)	−0·06 (−0·07 to −0·05)
		Angola	22·35 (11·92 to 36·24)	285·93 (229·98 to 350·10)	11·10 (6·36 to 16·37)	28·29 (23·41 to 33·79)	−0·03 (−0·10 to 0·02)	0·03 (0·01 to 0·05)	−0·01 (−0·05 to 0·02)
		Central African Republic	9·87 (4·92 to 17·25)	137·53 (115·36 to 162·34)	8·20 (6·84 to 9·68)	22·21 (18·48 to 26·35)	−0·02 (−0·08 to 0·03)	−0·04 (−0·05 to −0·02)	−0·06 (−0·08 to −0·04)
		Congo	7·08 (3·54 to 10·54)	97·57 (73·72 to 119·26)	4·83 (3·57 to 6·10)	21·03 (18·08 to 26·01)	−0·03 (−0·07 to 0·01)	−0·01 (−0·03 to 0·01)	−0·06 (−0·08 to −0·04)
		Democratic Republic of the Congo	32·55 (9·52 to 78·70)	588·53 (492·83 to 709·81)	35·90 (30·47 to 42·09)	24·84 (19·80 to 30·10)	−0·12 (−0·26 to −0·01)	−0·06 (−0·07 to −0·04)	−0·07 (−0·09 to −0·05)
		Equatorial Guinea	0·63 (0·17 to 1·55)	24·45 (20·84 to 28·49)	0·81 (0·42 to 1·18)	31·82 (26·45 to 39·19)	−0·18 (−0·33 to −0·06)	0·03 (0·01 to 0·04)	−0·02 (−0·07 to 0·03)
		Gabon	2·19 (0·70 to 4·39)	42·43 (35·47 to 50·80)	1·24 (0·82 to 1·55)	60·25 (52·88 to 67·83)	−0·07 (−0·18 to 0·02)	−0·02 (−0·04 to 0·00)	−0·07 (−0·11 to −0·04)

Data in parentheses are 95% uncertainty intervals. New infections and HIV/AIDS deaths are cumulative for the whole of 2015. The number of people living with HIV is the point prevalence (as a count) at the end of 2015. The number of people living with HIV receiving ART and the total number of people living with HIV are year-end point prevalences. We calculated numerators for incidence, prevalence, and mortality rates with counts as previously described. The denominator for each rate was population at mid-year. We age-standardised rates with the WHO age standard. We calculated ARC as the slope from the log of the value in 2015, to the log of the value in 2005: (log[value 2015]–log[value 2005])/10. ART=antiretroviral therapy. ARC=annualised rate of change. SDI=sociodemographic index.
