# Correction to *Lancet HIV* 2016; 3: e361–87

**DOI:** 10.1016/S2352-3018(16)30125-4

**Published:** 2016-08-22

*GBD 2015 HIV Collaborators. Estimates of global, regional, and national incidence, prevalence, and mortality of HIV, 1980–2015: the Global Burden of Disease Study 2015.* Lancet HIV *2016; **3:** e361–87—*In this Article, Kerrie E Doyle and David M Pereira have been added to the list of collaborators and Claudia C Pereira has been removed. These corrections have been made as of Aug 22, 2016.

