# Supplementary material for: Estimates of global, regional, and national incidence, prevalence, and mortality of HIV, 1980–2015: the Global Burden of Disease Study 2015
Source: Lancet HIV. 2016 Jul 19;3(8):e361–87. doi: 10.1016/S2352-3018(16)30087-X (PMC5056319; doi:10.1016/S2352-3018(16)30087-X)

THE LANCET HIV

Supplementary appendix

This appendix formed part of the original submission and has been peer reviewed. We post it as supplied by the authors.

Supplement to: GBD 2015 HIV Collaborators. Estimates of global, regional, and national incidence, prevalence, and mortality of HIV, 1980–2015: the Global Burden of Disease Study 2015. *Lancet HIV* 2015; published online July 19. http://dx.doi.org/10.1016/S2352-3018(16)30087-X.

Appendix to Global, regional, and national incidence, prevalence, and mortality for HIV, 1980–2015: estimates from the Global Burden of Disease Study 2015

This appendix provides methodological detail, supplemental figures and more detailed results for HIV estimation.

This study complies with the Guidelines for Accurate and Transparent Health Estimates Reporting (GATHER) recommendations, and this appendix is more comprehensive and encyclopedic than previous Global Burden of Disease appendices. It includes detailed tables, figures, and information on HIV modeling and data in an effort to maximize transparency in our estimation processes and provide a comprehensive description of analytical steps. Components of this document are the same as described in the appendix to our "Global, regional, and national incidence and mortality for HIV, tuberculosis, and malaria during 1990–2013: a systematic analysis for the Global Burden of Disease Study 2013" paper; much more of this appendix is new text. We intend this to be a living document, to be updated with each annual iteration of the Global Burden of Disease.

Contents

Part 1. HIV Estimation Process in GBD 2015....................3
Part 2. GBD Results and Data Input Sources....................25
Part 3. Figures and Tables....................26

Figures and Tables

Appendix Table 1. Country-specific estimates of new HIV infections, counts of PLWH, counts of HIV/AIDS deaths, ART coverage per person LWH in 2015, and ARCs of age-standardized incidence, prevalence, and mortality rates from 2005 to 2015 among women and girls

Appendix Table 2. Country-specific estimates of new HIV infections, counts of PLWH, counts of HIV/AIDS deaths, ART coverage per person LWH in 2015, and ARCs of age-standardized incidence, prevalence, and mortality rates from 2005 to 2015 among men and boys

Appendix Table 3. Data sources used for GBD2015 On-ART Mortality Analysis

Appendix Table 4. Appendix Table 4. Data sources used in GBD 2015 off-ART analysis

Appendix Table 5. HIV-specific mortality for patients not receiving ART in all locations by initial CD4 and age (per 100)

Appendix Table 6A. HIV-specific mortality for patients on ART in all sub-Saharan African sites by time since ART initiation, sex, initial CD4 and age (per 100)

Appendix Table 6B. HIV-specific mortality for patients on ART in all developed sites by time since ART initiation, sex, initial CD4 and age (per 100)

Appendix Table 6C. Appendix Table 6C. HIV-specific mortality for patients on ART in all developing sites outside of sub-Saharan African by time since ART initiation, sex, initial CD4 and age (per 100)

Appendix Table 7. Socio-Demographic Index (SDI) values for countries and territories, 1980, 1985, 1990, 1995, 2000, 2005, 2010, 2015.

Appendix Table 8. HIV Comparison of GBD, Optima, and AEM

Appendix Figure 1. HIV Prevalence by country, 2015

Appendix Figure 2. Difference between 81% ART coverage and ART coverage by country, 2015

Appendix Figure 3. Global number of deaths due to HIV split by region with global uncertainty intervals from 1980 to 2015

Appendix Figure 4. Global number of people living with HIV split by region with global uncertainty intervals from 1980 to 2015

Appendix Figure 5. Comparing GBD and UNAIDS estimates of deaths due to HIV in Sub-Saharan Africa in 2014

Appendix Figure 6. Comparing GBD and UNAIDS estimates of adult HIV prevalence rate in Sub-Saharan Africa in 2014

Appendix Figure 7. HIV/AIDS mortality rate by country, 2015

Appendix Figure 8. HIV incidence rate by country, 1990

Appendix Figure 9. HIV incidence rate by country, 2005

Part 1. HIV Estimation Process in GBD 2015

The goal of the all-cause mortality estimation process for the GBD is to produce the most accurate time series estimates for 1970 to 2015 for all-cause deaths and death rates broken down into the GBD age groups and by sex for all 591 geographies in the GBD 2015. This task is necessarily complicated by the diversity of data sources available for all-cause mortality for different age groups, the known biases in some sources, and the powerful effects of the HIV epidemic in countries with large epidemics on the age pattern of mortality.

In GBD 2015, we have significantly improved the analytical links between HIV/AIDS estimation process and the all-cause mortality estimation process as the great interdependency between the two. GBD analysis of all-cause mortality can be categorized into five distinct but interconnected areas: under-5 mortality estimation, adult mortality rate estimation, a model life table system that estimates full age pattern of mortality rates, estimation of HIV mortality, and final estimates of age-specific mortality including HIV, and fatal discontinuities.

Details on all-cause mortality estimation process is described in GBD 2015 Mortality and Causes of Death capstone paper and elsewhere.[1] The all-cause mortality estimation process in GBD 2015 generates HIV-free mortality which is one of the key inputs to estimation of on- and off-ART mortality used in both EPP and Spectrum, and the Spectrum model for background mortality. Here in this article, we focus on the details on the input to and estimation process of HIV/AIDS within the GBD analytical framework.

In the following sections, we introduce data and methods used in HIV/AIDS estimation process in detail, as illustrated by the flowchart below.

Fig. A: HIV/AIDS Estimation Flowchart

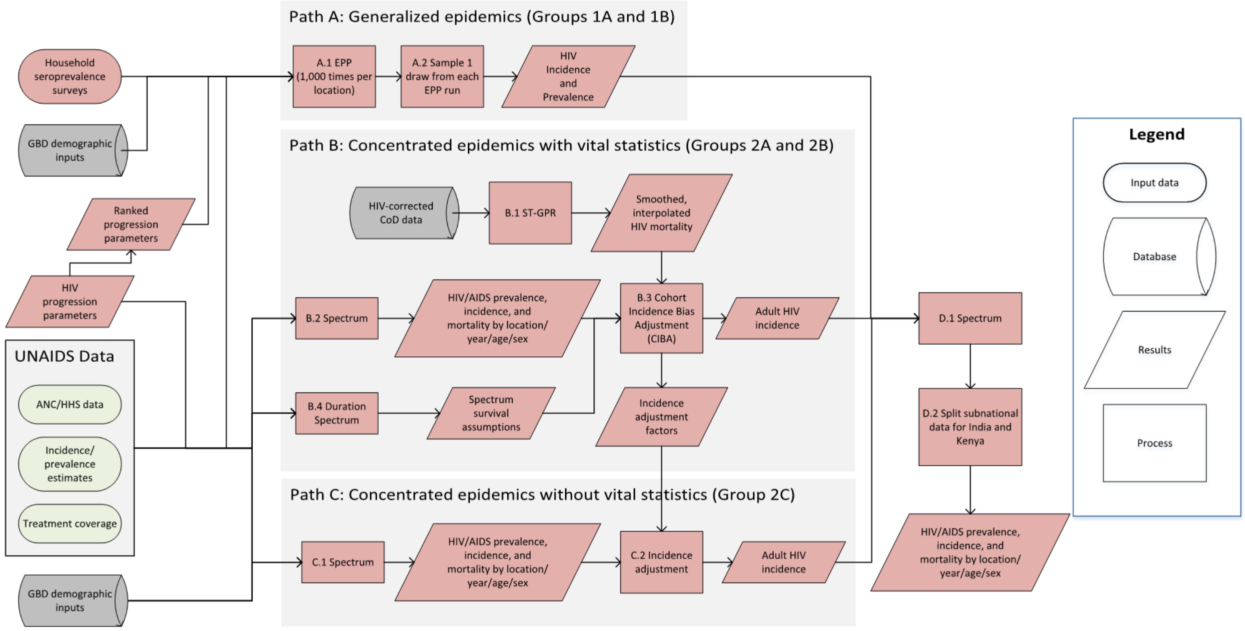

Section 1. Input data

1.1 Household seroprevalence surveys

Geographically representative HIV seroprevalence survey results were used as inputs to the model for countries with generalized HIV epidemics where available in addition to what have been included in the UNAIDS country files.

1.2 Demographic inputs from Global Burden of Disease 2015

Location-specific population, fertility, and HIV-free survival rates from GBD 2015 and migration data from UNAIDS were used as inputs in modeling all locations.

1.3 Program data and other key inputs to EPP/Spectrum

Antenatal care, incidence, ART treatment coverage data and all model parameters except on- and off-ART mortality are from UNAIDS country files and were used in modeling for all locations.

1.4 HIV cause specific mortality from vital registration systems

HIV cause specific mortality from vital registration system is one of the more reliable sources for estimating burden of HIV/AIDS. In GBD2015, we have extensively used HIV mortality data for estimating HIV burden in countries with a working vital registration system. We have corrected for both incompleteness of the vital registration system and erroneous coding for deaths related HIV/AIDS.

1.4.1. Adjustment of vital registration data to account for under-registration

For the all-cause mortality analysis in GBD, we have evaluated the completeness of each vital registration system by year using the improved data synthesis methodology developed for GBD. Details of the methods are discussed elsewhere.[1] Essentially, we apply the formal demographic methods, Death Distribution Methods, to generate point estimates of completeness using mortality data from vital registration systems and population from census. These raw estimates are then synthesized with the implied completeness for the under-5 age group from GBD's assessment of under-5 mortality rate to generate final time series estimates of complete of vital registration system for a country using space-time regression.[2] The figure below show the analytical steps for the estimation of completeness for Brazil.

Fig B: Vital Registration Completeness in Brazil

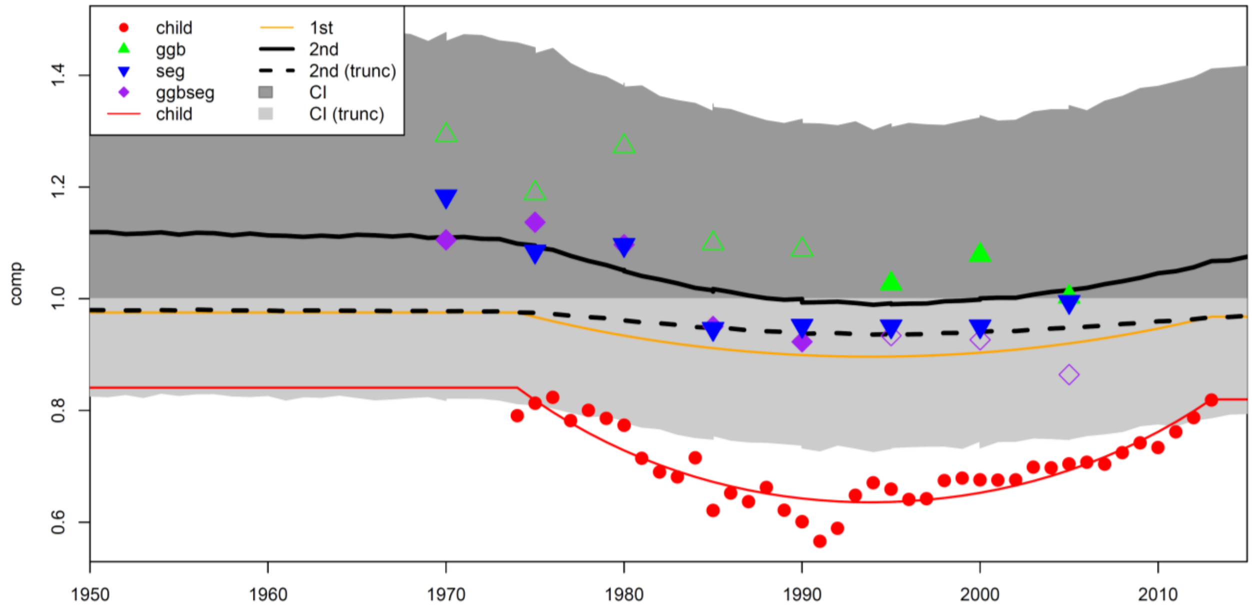

In the figure, Generalized Growth Balance (ggb), Synthetic Extinct Generation (seg), and the combination of the two (ggbseg) are the death distribution methods we use to generate point estimates for completeness. Child is the ratio of under-5 mortality rate from vital registration system compared to estimated under-5 mortality rate by GBD2015. The dash line in black is the final estimate of completeness for Brazil.

1.4.2. Corrections to HIV cause specific mortality

To account for the various erroneous coding for HIV related deaths, three distinctive process are applied to arrive at accurate count of deaths due to HIV for populations with vital registration systems, namely HIV-related garbage redistribution, redistribution to HIV as part of overall garbage redistribution, and HIV misclassification correction.

1. Before garbage code redistribution for all cause specific deaths, specific HIV-related garbage redistribution is performed to account for the disparate nature of HIV/AIDS mortality among geographies and over time. To capture such distribution, age band (under 1 month, 1-59 months, 5-19 years, 20-49 years, 50-59 years, 60-69 years , 70-79 years, and 80 plus years), time (five year time period) and sex specific target proportions are generated for each garbage code group. The redistribution of death from garbage code groups is based on the regional increase in mortality rate of death rate in the garbage code group relative to the death rates observed in the period 1980 to 1984. Any relative increase exceeding 5% is deemed to come from deaths related to HIV/AIDS and these excess death exceeding 5% are redistributed to HIV/AIDS. If the increase is smaller than 5%, then the excess deaths are all redistributed to reminder target that is non-HIV related.
2. We also apply the general non-HIV related garbage code redistribution. This in general has minor impact on the final HIV-related mortality. Details of the method are described in Murray CJL, Ortblad KF, Guinovart C, et al. Global, regional, and national incidence and mortality for HIV, tuberculosis, and malaria during 1990–2013: a systematic analysis for the Global Burden of Disease Study 2013. Lancet 2014; 384: 1005–70.

3. As we have observed in many vital registration systems, specific causes of deaths that are commonly comorbid with HIV/AIDS such as tuberculosis have age patterns that deviate from those observed in country-years without the HIV/AIDS epidemics. Such devastation are likely due to the trends in HIV/AIDS mortality. In this study, we follow the method developed by Birnbaum et al (Birnbaum, Jeanette Kurian, Christopher JL Murray, and Rafael Lozano. "Exposing misclassified HIV/AIDS deaths in South Africa." Bulletin of the World Health Organization 89.4 (2011): 278-285.) To correct to misclassified causes of death to HIV related deaths. Essentially, we generate global standard relative risk due to specific causes of deaths by age using all vital registration data from country years with HIV prevalence lower than 1%. The reference in generating the relative age pattern is the average mortality rates in age groups 65-69, 70-74, and 75-79. Using these global relative age pattern, expected cause and specific mortality rate can be generated using the observed cause specific mortality rate in the reference age groups (ages 65-79. We then compare these expected age specific rates with the observe age specific mortality rates for a specific causes of death. The excess age specific mortality, which is the difference between observed and expected age specific rate, from a specific cause of death is reallocated to HIV/AIDS.

The graph below shows the raw numbers of death due to HIV based on originally assigned HIV mortality in the vital registration system from Russia, and the impacts of HIV related redistribution procedures on the final HIV related deaths in our analysis.

Fig. C: Corrections to HIV-related deaths in Russia, 2001-2014

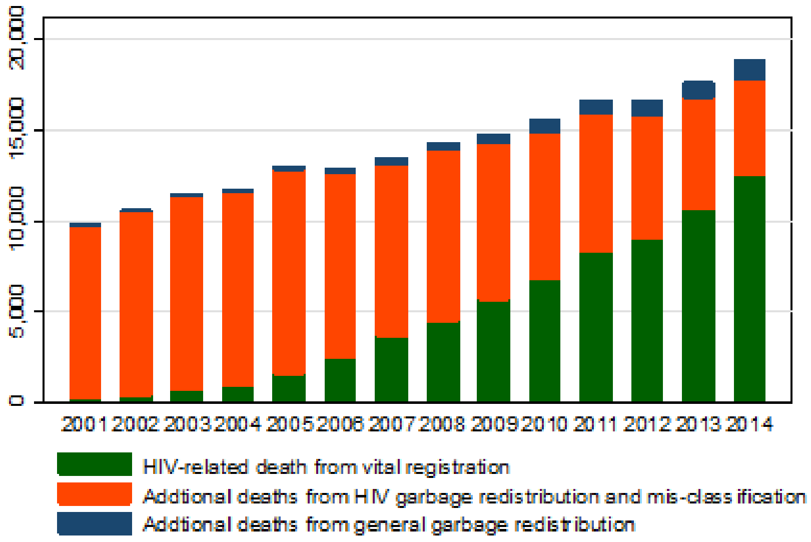

Section 2. Key parameter updates: On-ART and Off-ART mortality

2.1 On-ART mortality synthesis

2.1.1 On-ART mortality literature data

A PubMed search was conducted using the terms: *("hiv"[MeSH Terms] OR "hiv"[All Fields]) AND*

("mortality"[Subheading] OR "mortality"[All Fields] OR "mortality"[MeSH Terms]) AND antiretroviral[All Fields] AND ("therapy"[Subheading] OR "therapy"[All Fields] OR "therapeutics"[MeSH Terms] OR "therapeutics"[All Fields]). The articles identified by this search were considered for inclusion in three separate meta-analyses: (1) probability of death by initial CD4 count; (2) age hazard ratios for mortality; and (3) sex hazard ratios for mortality. Exclusion criteria for all studies included non-ART-naïve populations, children, and special populations such as hospitalized patients. For probability of death, 41 articles were included, contributing data for 62 distinct cohorts. For sex hazard ratios, 90 articles were included, contributing data for 98 cohorts. For age hazard ratios, 49 articles were included, contributing data for 50 cohorts. Included in this analysis, we have received cohort data through collaboration with the Antiretroviral Therapy Cohort Collaboration (ART-CC) for 10 developed countries.[3] Table 3 shows the data sources included in these analyses.

For duration-specific survival data, studies must report uncertainty on mortality estimates or provide stratum-specific sample sizes and must include duration-specific data to allow for calculation of 0-6, 7-12, or 13-24 month conditional mortality. In addition, studies must either report separate mortality and loss-to-follow-up (LTFU) curves, be corrected for LTFU using vital registration data, or be conducted in a high-income setting. Finally, studies must report the percent of participants who are male, the median age of participants, and either data with specific data on the number of CD4 T lymphocytes (CD4 counts) or the median CD4 count used for the data.

Hazard ratio data for ages or sexes can only be used if the hazard ratios are controlled for other variables of interest (age, sex, and CD4 category).

In GBD 2013, we identified 102 papers for extraction. For GBD 2015, we included 13 additional studies informing the duration-specific mortality estimation process and 26 studies informing the age and sex hazard ratio estimation process (some studies were used and counted in both). We also added one study to our LTFU analysis. In addition, we updated our data from the Antiretroviral Therapy Cohort Collaboration (ART-CC) with country-specific data pre- and post-2001 for enhanced use in estimating time trends for high-income countries. We excluded nine hazard ratio and four duration-specific mortality studies used in GBD 2013 which reported results on populations already present in other extracted studies. The inclusion of new ART-CC data necessitated the exclusion of four additional studies used in GBD 2013.

We also included on-ART cohort mortality data from 10 high-income nations with collaboration from ART-CC. These countries include Austria, Denmark, France, Germany, Italy, the Netherlands, Spain, Switzerland, the United Kingdom, and the United States. We excluded the US data because they were not fully representative of the complete with-HIV on-ART population at the time.

2.1.2 On-ART mortality data synthesis methods

First, we corrected reported probabilities of death for loss to follow-up using an update of the approach developed by Verguet and colleagues.[4] Verguet and colleagues used tracing and follow-up studies to empirically estimate the relationship between death in LTFU and the rate of LTFU. The relationship was estimated using the following equation:

$$ln\left(\frac{M_{LTFU}}{1-M_{LTFU}}\right) = \beta_0 + \beta_1 ln\left(\frac{P_{LTFU}}{1-P_{LTFU}}\right) + \varepsilon$$

Where M_{LTFU} is the proportion dying among those LTFU and P_{LTFU} is the proportion of the study LTFU.

After extracting the survival data into duration-specific conditional mortality, we used DisMod-MR 2.0 to synthesize the data into estimates of conditional probability of death over initial CD4 count.[5] We modeled the data separately by duration and added a fixed effect on whether the study was conducted prior to 2002. Each analysis was conducted separately for high-income countries, GBD low-income countries outside of sub-Saharan Africa, and sub-Saharan Africa.

To create estimates of age-specific hazard ratios, we synthesized hazard ratio data in five broad age groups: 15-25, 25-35, 35-45, 45-55, 55-100, and modeled the data using DisMod-MR 2.0.

To create estimates of sex-specific hazard ratios, we use the *metan* function in Stata to create estimates of relative risks separately by region, using female age groups as the reference group.

The CD4-specific mortality rates were multiplied by the age hazard ratios to generate age-specific estimates. The sex hazard ratios were applied using the following equations:

$$M_f = \left(\frac{M_{all}}{HR * p_m - p_m + 1}\right)$$

$$M_m = M_f * HR$$

Where M_f is the female mortality rate, M_{all} is the overall mortality rate found in the mortality studies, HR is the hazard for males compared to females, p_m is the proportion male, and M_m is the male mortality rate.

We then subtracted HIV-free mortality from the model life table process to calculate HIV-specific mortality, and used 1,000 draws from the posterior distribution for each age, sex, and CD4 category for conditional probabilities of death for 0-6 months, 7-12 months, and 13-24 months after initiation of ART as inputs into Spectrum. Tables 6a, 6b, and 6c show on-ART mortality in Sub-Saharan Africa, High Income countries, and developing countries outside of Sub-Saharan Africa.

In GBD 2015, our primary methodological change was the analysis of on-ART mortality using a fixed effect on studies before/after 2002, only in the high-income region, to estimate conditional probability of death in DisMod-MR 2.0. By doing so, we incorporated changes over time in the quality of on-ART care which may improve on-ART mortality. This change was also complemented by the inclusion of time-split data from ART-CC, which allowed us to incorporate this time trend across the large cohort. We then used the estimated post-2002 on-ART mortality through the rest of the on-ART estimation process.

2.2 Off-ART mortality synthesis

2.2.1 Off-ART literature data

In GBD 2013, we reviewed the literature of HIV mortality in the absence of ART using the PubMed search terms *(((((HIV) AND (mortality OR survival OR death) AND (seroconverters OR seroconversion OR seropositive OR seropositivity)))* and also screened the references of articles discovered by the search. These terms identified 2809 studies. These titles were screened and 145 abstracts were flagged for review. Studies were considered if they contained all-cause adult survival curves from time of sero-

conversion. In total, we included 13 studies as shown in Table 4 below. All pooled, age, and sex-specific survival data was extracted at one-year intervals up to 12 years since sero-conversion, after which data availability was sparse.

2.2.2 Off-ART mortality data synthesis method

Following UNAIDS assumptions, off-ART mortality is modeled as shown in the figure below.[5]

Fig. D: Off-ART mortality modelling

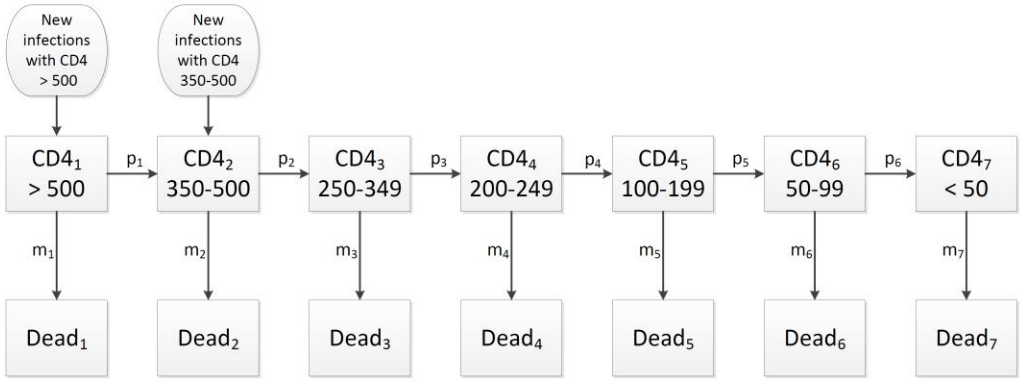

Mortality

$$m_i = ae^{bi}$$

Progression

$$p_i = \frac{c + di}{w_i}$$

Δt = time step interval

i = CD4 bin index

w_i = CD4 bin range

a, b, c, d = optimized constants

The death and progression rates between CD4 categories vary by age according to four age-groups, 15–24 years, 25–34 years, 35–44 years, and 45 years or older. We modeled the logit of the conditional probability of death between years in these studies using the following formula:

$$logit\ (m_{ijk}) = \beta_0 + \sum_{i=1}^{4} \beta_{1i}\, a_i + \sum_{j=1}^{12} \beta_{2j} t_j + u_k + \varepsilon_{ijk}$$

In the formula, m is conditional probability of death from year t_j to t_{j+1}, a_i is an indicator variable for age group at seroconversion (15–24 years, 25–34 years, 35–44 years, and 45 years or older), t_j is an indicator variable of year since seroconversion, and u_k is a study-level random effect.

By sampling the variance-covariance matrix of the regression coefficients and the study-level random effect, we generated 1,000 survival curves for each age group that capture the systematic variation in survival across the available studies. The AIDS Impact Model (AIM) component of Spectrum takes age and CD4-specific mortality and progression probabilities as inputs for epidemic estimates. These parameters are determined by optimizing a compartmental model such that the output matches Weibull-distribution fitted to survival data from three East African cohorts and a South African miners cohort.[6] We programmed a version of the UNAIDS compartmental model into R in order to quickly cycle through survival curve draws from our statistical model. For each of these survival curves, we assume exponentially increasing mortality hazards and linearly increasing progression hazards across CD4 bins. We calculated loss as the sum of the squared errors between the predicted survival curve and the survival draw from the statistical model. This loss function was optimized using the optim package in R.

Table 2 shows the off-ART mortality we have used in estimating burden of HIV/AIDS.

Section 3. HIV/AIDS Modeling strategy

3.1 HIV burden estimation overview

UNAIDS uses two key analytical components in their epidemiological estimation. EPP is used to estimate incidence trajectories that are consistent with prevalence surveys and other prevalence measurements such as antenatal clinic serosurveillance. Spectrum is a compartmental HIV progression model used to generate age-specific incidence, prevalence, and death rates from the EPP incidence curves and assumptions about intervention scale-up and local variation in epidemiology.

For GBD 2013, we created an exact replica of Spectrum in Python. This enabled us to run thousands of iterations of the model at once on our computing cluster and allowed for more flexible input data structures. Additionally, in order to generate estimates with more realistic ranges of uncertainty than those in UNAIDS 2012, we adjusted all input data by uniformly sampled factors between 0.9 and 1.1.

Our general modeling strategy for estimating HIV incidence, prevalence, and mortality is similar in many ways to the strategy used by Murray et al for GBD2013.[7] In the current study, we continue to use the Spectrum program rewritten in Python for GBD 2013 to facilitate faster and more flexible execution necessary for our more intensive computational needs. We made several changes to Spectrum's assumptions comparing to the Spectrum software used by UNAIDS. A key change in GBD 2015 is the application of EPP using an open-source computer program in R written by Jeffrey Eaton.[8] We ran EPP for all group 1 countries, defined in the following section, in order to produce incidence curves that were consistent with the demographic and epidemiological assumptions used in GBD 2015. This differed from GBD 2013, where we used the incidence curves provided by UNAIDS. The integration of EPP into our modeling process when feasible enables more robust and internally consistent incorporation of parameter uncertainty in generalized epidemics, and we have vastly improved the accuracy of the incidence adjustment used to fit Spectrum to high-quality vital registration data. Details of the impacts are included in the descriptions of the appropriate country strategies.

As part of the HIV/AIDS estimation process, all GBD 2015 locations were assigned to a modeling strategy group as shown in section 3 depending on the level of HIV within the country and the availability and quality of HIV and VR data. Groups were used to determine which sources to use for HIV-specific mortality data and how to calculate final estimates of HIV and all-cause mortality.

Group 1 includes much of Sub-Saharan Africa and other locations where HIV prevalence survey data is available to generate incidence curves using EPP. In Group 1 locations, demographic assessments depend substantially on the sibling history data analysis which have large uncertainty intervals and for which there may be local variation in biases in sibling history responses. All Group 1 locations except for India are classified as Group 1A, while India and its states are classified as Group 1B, where we maintained more influence from the SRS system used in all-cause mortality estimation.

Table: Group 1 Locations

Angola	DR Congo	Guinea Bissau	Niger	Swaziland
Benin	Djibouti	Haiti	Nigeria	Tanzania
Botswana	Dominican Republic	India (1B)	Papua New Guinea	Togo
Burkina Faso	Equatorial Guinea	Kenya	Rep of the Congo	Uganda
Burundi	Eritrea	Lesotho	Rwanda	Zambia
Cameroon	Ethiopia	Liberia	Senegal	Zimbabwe
Cape Verde	Gabon	Malawi	Sierra Leone	
Central African Rep	Gambia	Mali	Somalia	
Chad	Ghana	Mozambique	South Africa	
Cote D'Ivoire	Guinea	Namibia	South Sudan	

Group 2A locations have at least 25 years of complete vital registration since 1980, as assessed by GBD's VR completeness synthesis method described in Naghavi et al.[9] Since these locations have high-quality vital registration data, all-cause mortality estimates are largely driven by national data and the vital registration system is a robust source of data for HIV deaths.

Table B: Group 2A Locations

Antigua and Barbuda	Croatia	Ireland	Mexico	Slovenia
Argentina	Cuba	Israel	Moldova	Spain
Australia	Czech Republic	Italy	Netherlands	South Korea
Austria	Denmark	Japan	Philippines	Sweden
Barbados	Estonia	Kazakhstan	Poland	Switzerland
Belgium	Finland	Kuwait	Portugal	Trinidad and Tobago
Bermuda	France	Latvia	Puerto Rico	Ukraine
Bulgaria	Germany	Lithuania	Romania	United Kingdom
Canada	Greece	Luxembourg	Russia	United States of America
Chile	Guatemala	Macao	Saint Lucia	Uruguay
Colombia	Hong Kong	Malta	St. Vincent and the Grenadines	Venezuela
Costa Rica	Hungary	Mauritius	Singapore	

Remaining locations with any VR make up Group 2B, and locations without any VR data are Group 2C. Exceptions were made for Madagascar, which was reclassified from Group 2B to 2C due to data quality concerns, and Cambodia, which was treated as a group 2B instead of 2C because of its available HIV prevalence data from the 2005 DHS survey (see the HIV/AIDS modeling write-up in Part 3 for more details). All subnational locations were classified according to the national-level group.

Table: Group 2B Locations

Albania	Cambodia	Honduras	Nicaragua	Sri Lanka
Algeria	China	Iran	Northern Mariana Isl	Suriname
American Samoa	Cyprus	Iraq	Oman	Syria
Armenia	Dominica	Jamaica	Palestine	Taiwan
Azerbaijan	Ecuador	Jordan	Panama	Tajikistan
Bahamas	Egypt	Kiribati	Paraguay	Thailand
Bahrain	El Salvador	Kyrgyzstan	Peru	Tonga
Belarus	Fiji	Malaysia	Qatar	Tunisia
Belize	FYR Macedonia	Maldives	Sao Tome and Principe	Turkey
Bolivia	Georgia	Mongolia	Saudi Arabia	Turkmenistan
Bosnia and Herzegovina	Greenland	Montenegro	Serbia	U.S. Virgin Isl
Brazil	Guam	Morocco	Seychelles	Uzbekistan
Brunei	Guyana	Myanmar	Slovakia	

Table: Group 2C Locations

Afghanistan	Indonesia	Mauritania	Timor-Leste
Andorra	Laos	Nepal	United Arab Emirates
Bangladesh	Lebanon	North Korea	Vanuatu
Bhutan	Libya	Pakistan	Vietnam
Comoros	Madagascar	Samoa	Yemen
Federated States of Micronesia	Marshall Islands	Solomon Islands	

Due to the substantial differences in the quality and types of data available across different countries, we used three different methodologies to produce year-, age-, and sex-specific estimates of HIV incidence, prevalence, and mortality.

3.2 HIV/AIDS estimation strategy for Countries with seroprevalence surveys and antenatal clinic data (Groups 1A and 1B)

We identified 43 countries – as well as 48 subnational locations from India, Kenya, Mozambique, and South Africa – with at least one geographically representative HIV seroprevalence survey. In order to ensure that our estimates of incidence and prevalence in these places were consistent with our estimates of HIV progression, we used a version of EPP written in R and C++ by Jeffrey Eaton to create new fits to the prevalence data in the UNAIDS files. By substituting in our own assumptions about HIV progression, we were able to ensure that the implied relationship between incidence and mortality/prevalence in EPP is similar to that in Spectrum.

In these locations, most of which experience generalized HIV epidemics, we expect estimates of HIV burden to exhibit substantial uncertainty. To reflect this, we induced a perfect correlation between the previously independent draws of HIV mortality with and without ART and CD4 progression. We paired the draws of the three parameter sets internally and with each other in the following way: we sorted

without-ART mortality and CD4 progression internally by age (not CD4), meaning the highest draw of HIV mortality without ART for age a_i and CD4 category c_l will be paired with the highest draw of HIV mortality without ART for age a_k and CD4 category c_l. In the same way, we sorted with-ART mortality internally by age, sex, CD4 count at treatment initiation, and duration on treatment. After this sorting process, the lowest indexed draw of each parameter has the highest values and vice versa. This means that we will use the most extreme possible parameter sets in EPP and Spectrum and should see a commensurate expansion in the range of the uncertainty.

To ensure that this expanded uncertainty is replicated in EPP, we fit the model once for every set of paired draws of the progression parameters for every location. This means that the first iteration of EPP for Uganda sees the highest draws of all three sets of progression parameters. Such a procedure is necessary because EPP currently has no mechanism for incorporating uncertainty in any inputs except prevalence data. This process (Process A.1 in the HIV/AIDS Estimation Flowchart), produced 1,000 sets of EPP output for each of the locations that make up the 47 countries in the group. Every set of EPP outputs contains 500 consistent draws of HIV incidence and prevalence in adults aged 15-49. In many cases, the algorithm used to fit EPP, incremental mixture importance sampling, failed, resulting in fewer than 1,000 sets of EPP results.

For every location in the group, we sampled one of the 500 incidence/prevalence draws from each of the sets of EPP results (Process A.2 in the HIV/AIDS Estimation Flowchart). By sampling one draw from each set, we ensured that the distribution of progression parameters dictating the relationship between incidence and prevalence was exactly the same as the distribution of the sorted parameters generated in the previous step. In locations where not all 1,000 iterations of EPP fit successfully, we sampled one draw from every iteration that did succeed and then resampled with replacement from that set of draws. To maintain the link between the input progression draws and the resulting incidence and prevalence draws from EPP, we replaced any parameter draw associated with a failed run of EPP with the parameter draw that that failed draw was replaced with. At the end of this process, for every location in the set of 47 countries, we were left with 1,000 linked draws of adult incidence and prevalence and the exact progression parameters that generated those draws.

We then ran these results, along with the previously described demographic and HIV-specific inputs, through Spectrum to produce location-, year-, age-, and sex-specific estimates of HIV incidence, prevalence, and mortality (Process D.1 in the HIV/AIDS Estimation Flowchart).

3.3. HIV/AIDS estimation strategy for countries with vital registration data (Group 2A and 2B)

Vital registration is one of the highest-quality sources of data on HIV burden in many countries, so generating estimates that are consistent with these data, with necessary adjustment to account for any potential underreporting, is critical. We identified 116 countries – as well as 208 subnational locations from Brazil, China, Japan, Mexico, Saudi Arabia, Sweden, the United Kingdom, and the United States – with vital registration data or sample registration systems such as the Disease Surveillance Points in China.

We imputed missing years of data to generate a complete time series for HIV from the estimated start year of the epidemic using ST-GPR. We analyzed mortality trends using ST-GPR starting in 1981, the year that HIV was first identified in the United States.[10] For ST-GPR, we adjusted the lambda (time weight) and GPR scale according to the completeness of vital registration data, based on whether a country had

10 or more years of complete VR data as analyzed by the Death Distribution Methods (DDM) model. We produced separate splines by country/age group, up to the peak year of death rate. We then ran a linear regression with random effects on region, age, and sex. Following this, we ran space-time residual smoothing, in which time, age, and space weights are used to inform smoothing of the residuals between data points and the linear regression estimate. From this process, we generated space-time estimates with the applied weights, along with the median absolute deviation (MAD) of the space-time estimates from the data. The MAD was calculated at various levels of the geographic hierarchy (e.g., subnational and national), and was added into the data variance term. The data variance and space-time estimates were then analyzed using Gaussian Process Regression to return a final estimate of mortality along with uncertainty.

Although Spectrum produces HIV mortality estimates that are within the realm of possibility in most countries using the incidence curves provided in the UNAIDS 2012/2015 country files, it is a deterministic model that has not yet been integrated into an optimizable framework. Therefore, in order to “fit” it to vital registration data, we needed to adjust input incidence. For GBD 2013, we used a process that assumed several different durations between HIV infection and HIV death and adjusted incidence based on death some number of years in the future. Although that method worked relatively well and substantially reduced the disconnect between Spectrum and the VR data, it required very rigid and unrealistic assumptions about these survival durations. For GBD 2015, we have improved the performance of this method, allowing Spectrum to fit to the VR data more closely.

To improve the fit of this process, we restructured Spectrum to add compartments that identify groups of people living with HIV by year of infection (Process B.4 in the HIV/AIDS Estimation Flowchart). With this version of Spectrum we can output, among many other metrics, HIV deaths by year, age, sex, and infection cohort. This enables us to adjust incidence to fit to death much more precisely and without making any rigid assumptions about the time from HIV infection to HIV death.

We have incorporated these improvements into a cohort incidence bias adjustment (CIBA) process. First, we ran Spectrum normally to produce 1,000 draws of incidence, prevalence and mortality (Process B.2 in the HIV/AIDS Estimation Flowchart). Then, by year, age, and sex, we took the ratio of VR deaths to Spectrum deaths to quantify the amount of bias in Spectrum. Using the mean duration data from the new version of Spectrum, for every year-, age-, and sex-specific infection cohort, we calculated the share of all HIV deaths observed over the course of the projection period in that cohort that would occur in each year after the year of infection. For example, projecting from 1970 through 2015, we identified the cohort of men infected in 1992 at the age of 16, calculated the total number of HIV deaths in that cohort in all subsequent years through the end of 2015, and divided the annual number of deaths by that total. This showed us the distribution of deaths among that cohort over the projection period. In the most extreme case (infections in 2014), we could only produce one point of that distribution (2015), so that single value is exactly 1.0; 100% of the deaths observed in that cohort occurred in 2015.

We then used these distributions of death to weigh the ratio of VR deaths to Spectrum deaths, meaning that ratios in the years where we expect the largest share of deaths were weighed most heavily. We then multiplied the initial size of that cohort from the normal run of Spectrum by the sum of the combined ratios to get a new estimate of new cases in that year/age/sex combination.

We can write this method mathematically in the following way:

$$r_t = \frac{VR_t}{D_t}$$

$$\rho_t^{t-i} = \frac{d_t^{t-i}}{\sum_{k=t-i+1}^{n} d_k^{t-i}}$$

$$\alpha^{t-i} = \sum_{k=t-i+1}^{n} r_k * \rho_t^{t-i}$$

$$n_{\text{adjusted}}^{t-i} = \alpha^{t-i} * n^{t-i}$$

VR_t is the number of HIV/AIDS deaths in year t from ST-GPR, and D_t is the number of HIV/AIDS deaths from the first run of Spectrum. In the second equation, d_t^{t-i} is the number of HIV/AIDS deaths among members of infection cohort $t-i$ in year t, with $i \geq 1$, from the new, duration-tracking version of Spectrum, and n is final year of the projection. Therefore, ρ_t^{t-i} is the share of observed deaths in cohort $t-i$ that we expect to occur in year t. It follows, that α^{t-i} is the weighted adjustment ratio described above, which we multiply by the estimated initial size of infection cohort $t-i$ as calculated in the first stage Spectrum run to get the adjusted nubmer of new cases, $n_{\text{adjusted}}^{t-i}$. This process is run separately for every sex and single-age pair.

CIBA (Process B.3 in the HIV/AIDS Estimation Flowchart) allows ratios in each year after a given infection year to influence the final adjustment to incidence. The size of that influence is determined by the relative importance of that year in the cohort-year's distribution of deaths over time. The result is a new set of 1,000 draws of incidence and a set of 1,000 ratios of post-adjustment incidence to pre-adjustment incidence. We perform this adjustment using mean durations from the new version of Spectrum in order to try to shift the mean of the regular distribution of deaths.

Finally, to produce location-, year-, age-, and sex-specific estimates of HIV incidence, prevalence and mortality, we ran the new estimates of incidence and all previously input data through Spectrum (Process D.1 in the HIV/AIDS Estimation Flowchart).

3.4 HIV/AIDS estimation strategy for countries without survey data and vital registration data (Group 2C)

The remaining 24 countries – as well as nine subnational locations from China and Saudi Arabia – had neither geographically representative seroprevalence surveys nor reliable vital registration systems. To produce estimates of HIV burden in these countries, we assumed that Spectrum is similarly biased as in other Group 2 countries. This involved running Spectrum (Process C.1 in the HIV/AIDS Estimation Flowchart), adjusting incidence using 1,000 adjustment ratios randomly sampled from the entire set of CIBA results (Process C.2), and rerunning Spectrum using the new draws of adjusted incidence (Process D.1). As above, the estimates of incidence, prevalence, and mortality were incorporated into the rest of the machinery via the reckoning process.

Originally, Cambodia, which does have a prevalence survey, was included in this group because we have not yet coded the machinery necessary to reproduce the Asian Epidemic Model used by UNAIDS to model prevalence and incidence in Southeast Asian countries.[11] The 2005 DHS survey in Cambodia made clear that we were underestimating the burden due to HIV there by not using survey data during the

modeling process. In order to more accurately represent the epidemic, we used the mortality profile from Thailand and scaled it by 80%, the ratio of estimated prevalence rate in Thailand in 2005 and the prevalence rate from the DHS survey in Cambodia. We then treated the scaled death series as VR data and added Cambodia to the 2B group that is run through CIBA.

3.5 Subnational splitting for India and Kenya

Spectrum results for India and Kenya subnational locations are modeled at higher levels of geography than our GBD locations. For example, Spectrum results for India are produced at the state level, while GBD 2015 estimates were produced at the state urban-rural level. Similarly, Spectrum is modeled at the province level, while we compute Kenyan subnational estimates for the 47 counties. To split the Spectrum results into more granular results for processing, we assign each GBD subnational unit to a Spectrum modeling unit. HIV deaths, incidence, births, prevalence, and population were split according to population, given the lack of HIV-specific data to inform the split. HIV-free mortality was split according to the HIV-free mortality as estimated by the model life table system.

3.6. HIV/AIDS resulting in other diseases

There are two Level 4 causes under the HIV/AIDS Level 3 cause in the GBD 2015 cause hierarchy. The modeling process for HIV/AIDS-tuberculosis is detailed in GBD2015 Mortality and Causes of Death capstone paper.[12] We computed deaths for HIV resulting in other diseases by subtracting HIV/AIDS-tuberculosis deaths from all HIV deaths at the 1,000 draw level.

Section 4. HIV/Mortality Reckoning

The Reckoning process is intended as a method of reconciling separate estimates of HIV mortality (and its resulting effect on estimates of HIV-free and all-cause mortality) due to two separate estimation processes within the GBD all-cause and HIV estimation framework: those from the model life table system as a way to capture the impact of HIV on age pattern of all-cause mortality, and the those from the natural history model of EPP-Spectrum as used by GBD and UNAIDS. In addition, we also utilize space-time GPR smoothed VR data on HIV specific mortality for countries with good quality VR instead of using mortality estimates from Spectrum based on back-calculated incidence using case report data.

For HIV mortality data, Group 2A locations used mortality output from the ST-GPR process due to the high quality of their vital registration systems. Group 1A and 1B locations used Spectrum output, while Group 2B and 2C locations used output from cohort incidence bias adjusted (CIBA) deaths due to HIV/AIDS from the Spectrum model.

Outputs were modeled to include an under-1 age group without the early-, late-, and post-neonatal groups. To attribute under-1 deaths from Spectrum to these neonatal groups, we make the assumption that all HIV deaths that occur in the first year of a child's life occur in the post-neonatal stage (after 28 days), since the literature on HIV in these age groups is still unclear but seems to indicate higher mortality in the post-neonatal stage, and there is no clear evidence to guide alternative methods of age-splitting under-1 deaths due to HIV.[13,14]

In general, the all-cause and HIV-deleted envelopes were generated by synthesizing the results from the with-HIV and HIV-free life tables, along with selected HIV mortality from ST-GPR, Spectrum output, or

cohort incidence bias adjusted Spectrum output (CIBA-Spectrum). We used with-HIV and HIV-free lifetables in order to ascertain the implied HIV from the model life table (MLT) system.

For age groups under age 5, we used the under-5 results from the lifetable age-sex process. In Group 1A and 1B locations, we generated a scalar based on Spectrum results of HIV-specific and non-HIV deaths to generate HIV-deleted envelope deaths based on the all-cause results from the envelope. In other locations, we directly subtracted mortality from ST-GPR or Spectrum, after capping HIV at 90% of the all-cause envelope, to generate the HIV-deleted envelope. As mentioned previously, we made the assumption that all under-1 HIV-specific deaths occur in the post-neonatal stage.

In all groups except Group 1A, for all ages above 5 and under 15, we subtracted mortality from Spectrum or ST-GPR directly from the all-cause with-HIV envelope, after capping HIV at 90% of the all-cause envelope. In Group 1A, we directly use mortality from Spectrum, without capping it as it is added directly to the HIV-free envelope.

For ages above 15 and below 80, we applied separate approaches for Group 1A and 1B locations compared to Group 2A, 2B, and 2C locations. Group 1A and 1B locations used HIV mortality determined by an ensemble model where we averaged the implied HIV mortality from the model life table process and the HIV mortality output by Spectrum, which are intrinsically linked by the draw-level HIV-free mortality age pattern. Group 2A, 2B, and 2C locations used HIV mortality directly from ST-GPR and CIBA-Spectrum.

For the age group 80 and above, we first approximated the over-80 mortality rate by using our lifetable output and divided lx by Tx from the age 80-84 values in the country-specific lifetables. We then calculated a scalar from the approximated all-cause mortality rate from the lifetable to the all-cause mortality rate by taking the envelope deaths/populations. We applied this scalar to both the with-HIV and HIV-free lifetables to rescale these numbers and the implied HIV death rate to the envelope space.

Section 5. Country examples

To illustrate the HIV/AIDS burden estimation process used in this study, in the following sections, we use Canada and Botswana as examples to demonstrate in details the analytical steps involved for group 1 and group 2 countries.

5.1. Estimating burden of HIV/AIDS for Canada

Canada is used as an example here to show how our analytical framework works when a country has vital registration data. Here we outline the analytical steps applied:

1. We extracted HIV-specific input data (including adult incidence and prevalence estimates, ART coverage, and PMTCT coverage) from UNAIDS' Spectrum file for Canada from their 2012 estimation process. We used GBD 2015 demographic inputs and estimates of HIV progression parameters for the remaining input data necessary for the demographic projection and natural history model.
2. We used data from the Canadian vital registration system retrieved from the WHO Mortality Database to estimate HIV/AIDS mortality and to inform our estimates of incidence and prevalence. To account for under-diagnosis of HIV/AIDS as a cause of death, we performed the

"HIV correction" (described in detail elsewhere). This process reallocates deaths from causes as which HIV/AIDS deaths are likely to have been misclassified to HIV/AIDS. In Canada specifically, the raw vital registration data identify zero HIV/AIDS deaths prior to 2000. The correction produces substantially

3. To fill in gaps in the mortality data, we then estimated HIV/AIDS mortality rates using ST-GPR. The data from the Canadian vital registration system include all required years and ages, so we fit the model to the data as closely as possible, as illustrated in the below figure.

Fig. E: HIV/AIDS death rates among males in Canada: corrected vital registration data with ST-GPR estimates

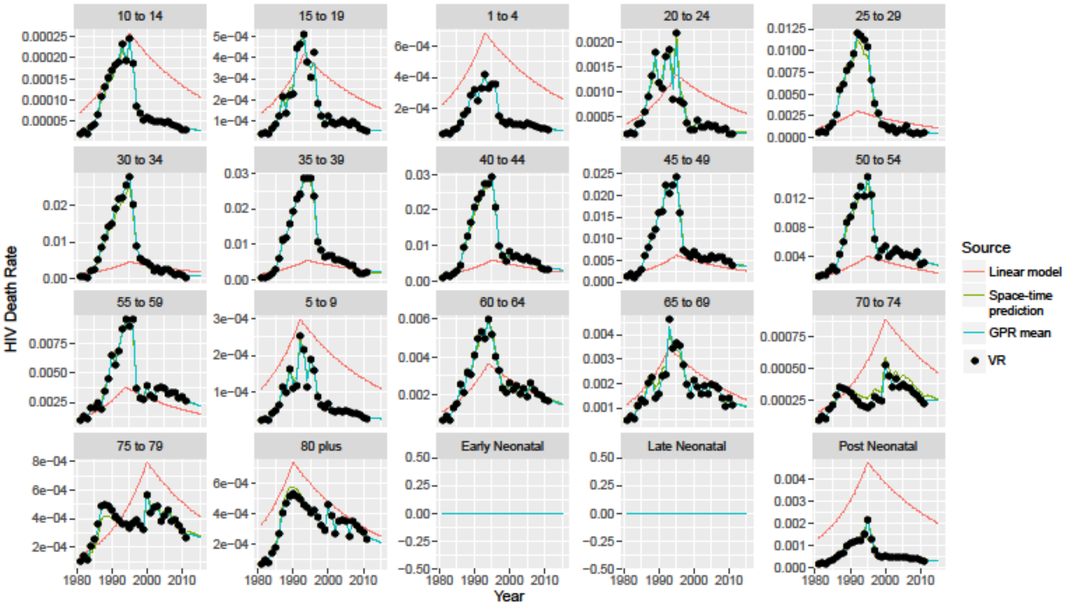

Concurrently, we used our version Spectrum that tracks compartments by year of infection to generate the inputs required to run CIBA. Specifically, we divided the number of HIV/AIDS deaths among a particular infection cohort in a particular year by the total number of HIV/AIDS deaths we observed in that cohort during the projection period. This gave us the distribution of deaths among a cohort over time. As can be seen in the figure below, people infected in 2014 have only one year in which they could have died due to HIV/AIDS (2015), so the value of this quantity in 2015 for that cohort is 1.0.

Fig. F: Share of observed HIV/AIDS deaths in a cohort by year in CAN

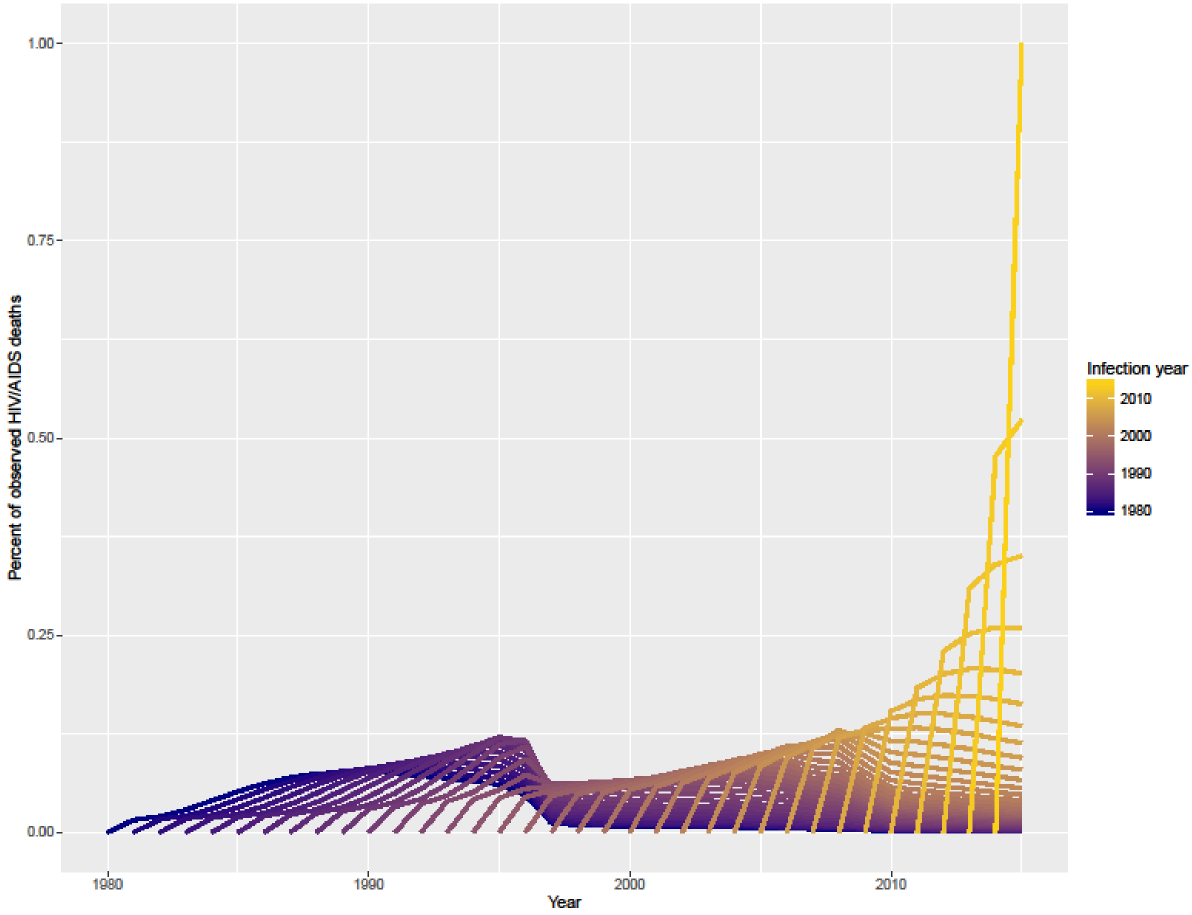

4. To generate initial estimates of adult incidence and corresponding mortality in Canada, we ran Spectrum 1,000 times. This produced what we referred to as "Stage 1" estimates.
5. We ran these Stage 1 estimates and the previously estimated death distributions through CIBA. As discussed elsewhere in the appendix, CIBA essentially attributes the bias in Spectrum's estimates of deaths in a particular year to each preceding year's incidence cohort. This allowed us to generate new adult incidence that enabled Spectrum to estimate deaths that match observed deaths extremely closely. We believe that the resulting incidence and prevalence curves are therefore more valid that the original UNAIDS curves.
6. We then ran the adjusted incidence curves through Spectrum 1,000 times to generate new estimates of incidence, prevalence, and mortality. We referred to this as "Stage 2." The figure below shows the change between Stage 1 and Stage 2.

Fig. G: GBD HIV estimation process in Canada

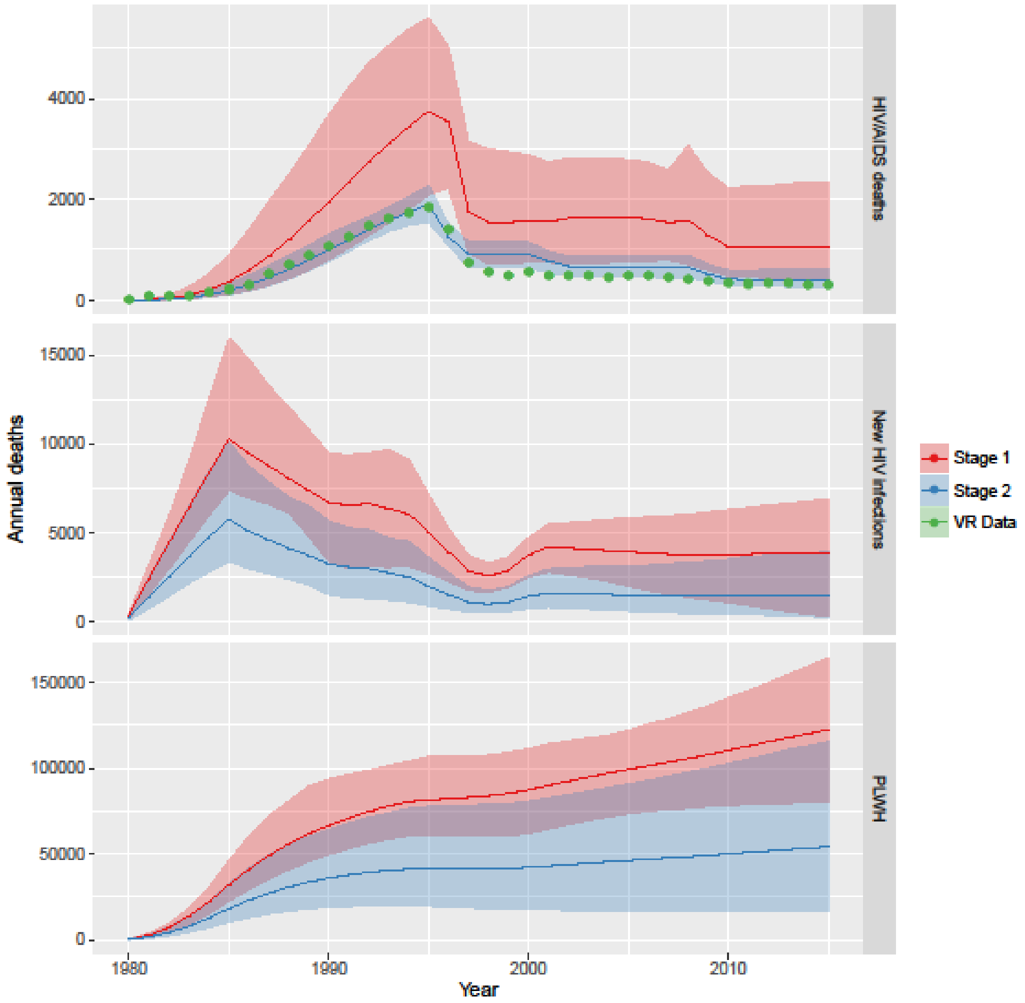

7. Stage 2 produces our final estimates of incidence and prevalence. Given that Canada's vital registration system is generally thought to be complete and that Spectrum will never be able to reproduce all of the variation in the data, we used the ST-GPR results as our estimates of mortality.

5.2 HIV burden estimation process for Botswana

For countries in group 1, or broadly speaking countries with generalized epidemics, we use Botswana as an example to illustrate the analytical steps.

1. We used the Estimation and Projection Package (EPP) to estimate 1000 draws of a complete time series of incidence and prevalence for Botswana. This process takes in draws of on- and off-ART mortality and CD4 progression ratios, as well as data such as ART coverage and PMTCT extracted from UNAIDS' 2015 Spectrum files for Botswana. Prevalence is fit to adjusted Antenatal Care (ANC) clinic data and prevalence from nationally representative surveys. All estimates are made for both sexes within the 15 to 49 age group. The figure below shows both input prevalence data and estimates from EPP.

Fig. H: Prevalence estimates for Botswana from EPP and Spectrum along with input data

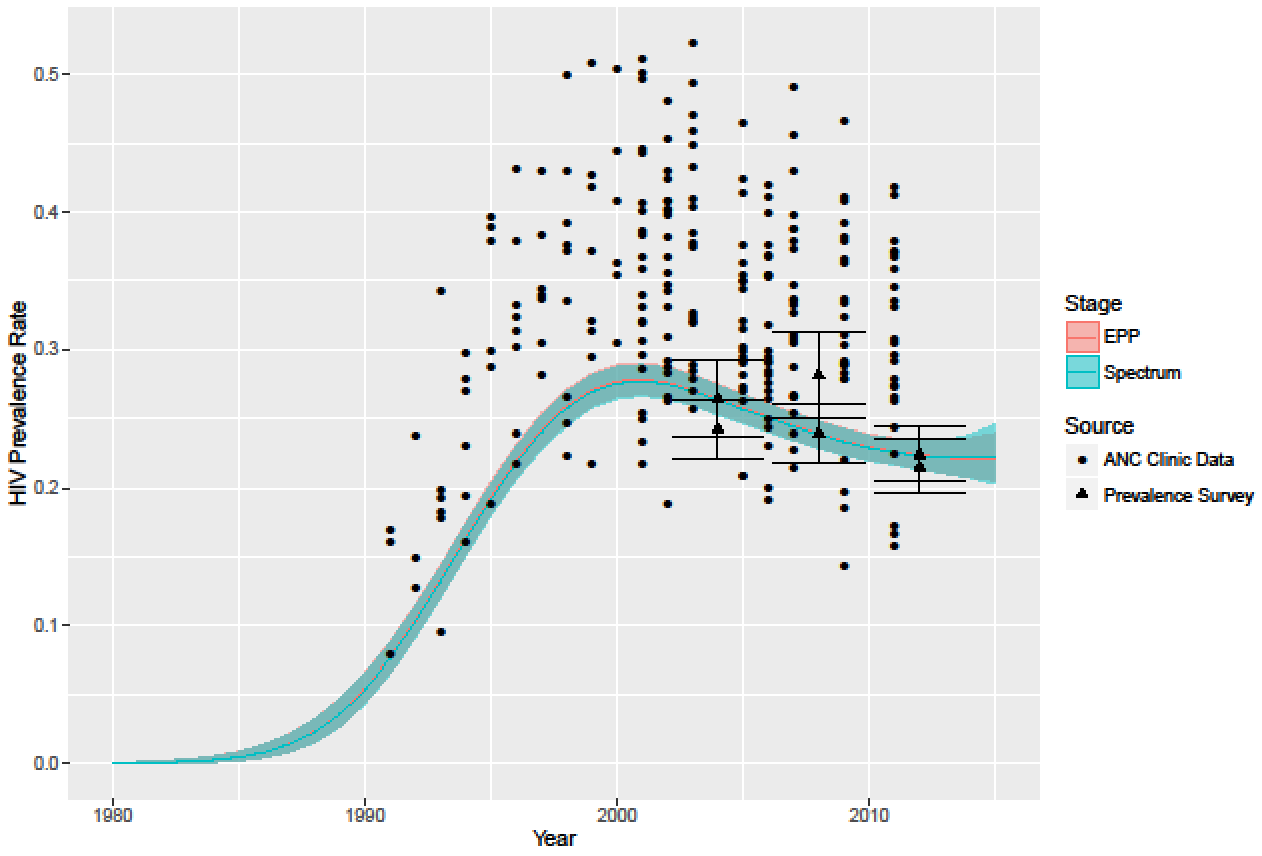

2. We used the GBD's version Spectrum recoded using Python to generate internally consistent estimates of incidence, prevalence, and death for both sexes and by five year age groups up to over 80 years of age. We also apply the draw level HIV-free mortality estimated by the GBD all-cause mortality estimation process. This ensures that draw level estimates from EPP/Spectrum and all-cause mortality process from GBD are internally consistent and intrinsically linked. Spectrum outputs include incidence, prevalence and mortality by age and sex over time.
3. In the GBD all-cause mortality process, we generate death due to all causes with implied HIV specific mortality.[1] While Spectrum estimates are driven by incidence derived from prevalence as informed by survey and ANC data, and assumption of HIV mortality on- and off-ART from limited cohort studies, all cause mortality estimates largely come from indirectly estimated raw data using sibling survival modules and sparse and most likely incomplete VR. In our current study, we applied an ensemble model on HIV specific mortality by averaging draw level HIV mortality estimates from Spectrum and the all-cause mortality process. By applying the ensemble, we effectively are giving equal weights to estimates from Spectrum and all-cause mortality process (most importantly the model life table system developed for GBD). The figure below shows the effect of the ensemble model for mortality in adult age groups for Botswana.

Fig. I: Mortality estimates for Botswana from Spectrum and Model Life Table

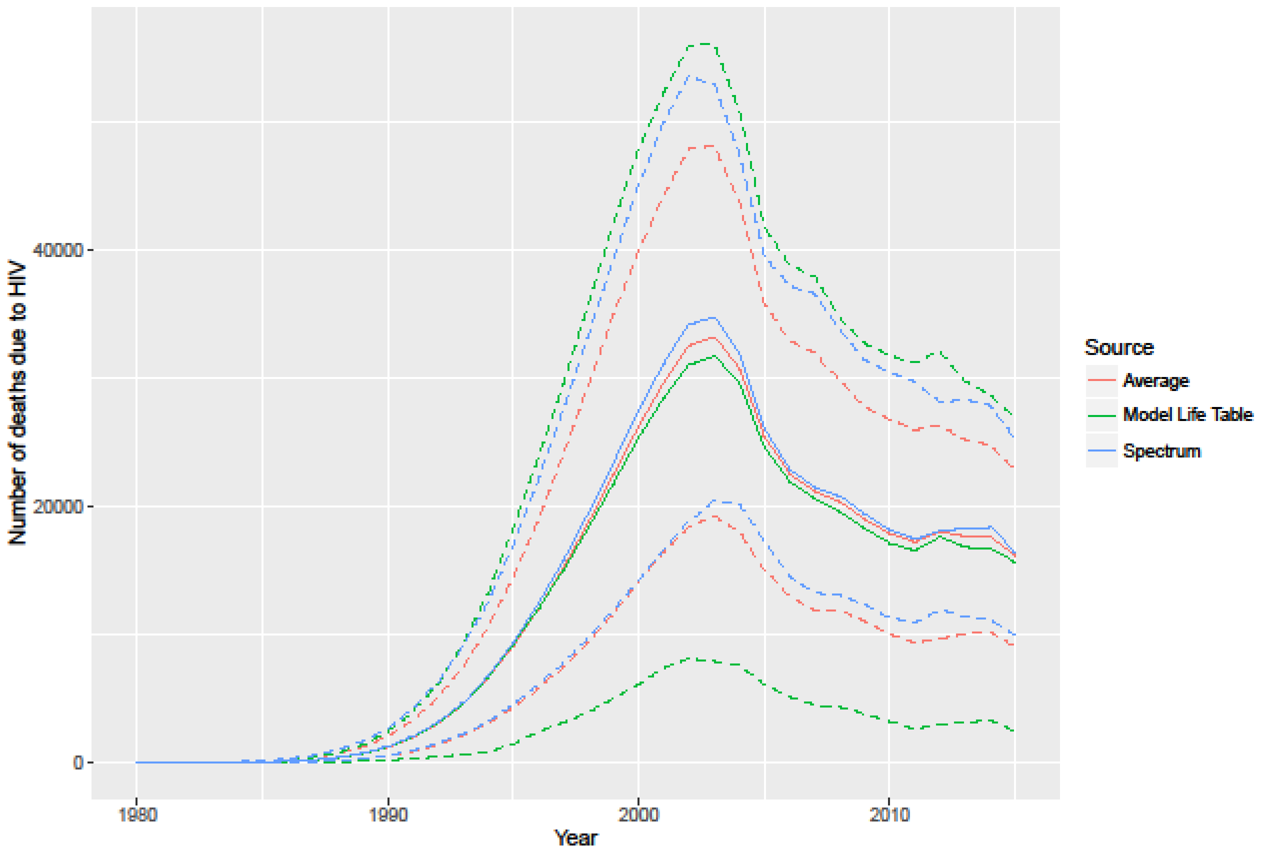

4. For incidence and prevalence, estimates are based on the model output from Spectrum.

References

1 Naghavi M, Wang H, Lozano R, *et al.* Global, regional, and national age–sex specific all-cause and cause-specific mortality for 240 causes of death, 1990–2013: a systematic analysis for the Global Burden of Disease Study 2013. *The Lancet* 2015; **385**: 117–71.

2 Wang H, Liddell CA, Coates MM, *et al.* Global, regional, and national levels of neonatal, infant, and under-5 mortality during 1990-2013: a systematic analysis for the Global Burden of Disease Study 2013. *Lancet Lond Engl* 2014; **384**: 957–79.

3 May MT, Ingle SM, Costagliola D, *et al.* Cohort profile: Antiretroviral Therapy Cohort Collaboration (ART-CC). *Int J Epidemiol* 2014; **43**: 691–702.

4 Gelman A, Carlin JB, Stern HS, Rubin DB. Bayesian Data Analysis, Second Edition, 2 edition. Boca Raton, Fla: Chapman and Hall/CRC, 2003.

5 Rajaratnam JK, Tran LN, Lopez AD, Murray CJL. Measuring Under-Five Mortality: Validation of New Low-Cost Methods. *PLOS Med* 2010; **7**: e1000253.

6 USAID. AIM: A computer program for making HIV/AIDS projections and examining the demographic and social impacts of AIDS. http://data.unaids.org/pub/Manual/2009/20090414_aim_manual_2009_en.pdf (accessed June 8, 2016).

7 Murray CJL, Ortblad KF, Guinovart C, *et al.* Global, regional, and national incidence and mortality for HIV, tuberculosis, and malaria during 1990–2013: a systematic analysis for the Global Burden of Disease Study 2013. *The Lancet* 2014; **384**: 1005–70.

8 jeffeaton/tasp-and-early-infection. GitHub. https://github.com/jeffeaton/tasp-and-early-infection (accessed June 9, 2016).

9 Wang H, Dwyer-Lindgren L, Lofgren KT, *et al.* Age-specific and sex-specific mortality in 187 countries, 1970-2010: a systematic analysis for the Global Burden of Disease Study 2010. *Lancet Lond Engl* 2012; **380**: 2071–94.

10 Kerber KJ, Lawn JE, Johnson LF, *et al.* South African child deaths 1990-2011: have HIV services reversed the trend enough to meet Millennium Development Goal 4? *AIDS Lond Engl* 2013; **27**: 2637–48.

11 Marston M, Becquet R, Zaba B, *et al.* Net survival of perinatally and postnatally HIV-infected children: a pooled analysis of individual data from sub-Saharan Africa. *Int J Epidemiol* 2011; **40**: 385–96.

12 GBD Mortality and Causes of Death Collaborators. Global, regional, and national life expectancy, all-cause and cause-specific mortality for 249 causes of death, 1980-2015: a systematic analysis of the Global Burden of Disease Study 2015. *Lancet* 2016; published online Forthcoming.

13 Brocklehurst P, French R. The association between maternal HIV infection and perinatal outcome: a systematic review of the literature and meta-analysis. *Br J Obstet Gynaecol* 1998; **105**: 836–48.

14 Kim H-Y, Kasonde P, Mwiya M, *et al.* Pregnancy loss and role of infant HIV status on perinatal mortality among HIV-infected women. *BMC Pediatr* 2012; **12**: 138.

Part 2. GBD Results and Data Input Sources

GBD Results

Results from the Global Burden of Disease Study (GBD 2015) for mortality, causes of death and illness, and risk factors for 195 countries and territories from 1990-2015 are now measured in terabytes. On the paper's publication date, results will be available in an interactive data downloading tool available in the Global Health Data exchange (GHDx). The tool will contain the complete set of results from all summary papers; however, specialized tables from the papers will available as separate entries in the GHDx as was made available in GBD 2013.

The current version of this data download tool is available in the GHDx and it contains core summary results for the Global Burden of Disease Study 2013 (GBD 2013): http://ghdx.healthdata.org/gbd-data-tool. The core summary results include deaths, YLLs, YLDs, and DALYs. It includes data for causes, risks, cause-risk attribution, etiologies, and impairments.

In the GBD 2015 version, the tool will contain additional measures such as prevalence and incidence as well as rate of change data and ranks. Data above a certain size cannot be viewed online but can be downloaded. Depending on the size of the download, users may need to enter an email address and a download location will be sent to them when the files are prepared.

Data input sources

The Global Burden of Disease Study 2015 (GBD 2015) incorporated a large number and wide variety of input sources to estimate mortality, causes of death and illness, and risk factors for 195 countries and territories from 1990-2015. On the paper's publication date, these input sources will be accessible through an interactive citation tool available in IHME's Global Health Data Exchange (GHDx).

The current version of this interactive citation tool is available in the GHDx and it provides input source information for the Global Burden of Disease Study 2013 (GBD 2013): http://ghdx.healthdata.org/gbd-2013-data-citations.

Users can retrieve citations for a specific GBD component, cause or risk, and geography by choosing from the available selection boxes. They can then view and access GHDx records for input sources and export a CSV file that includes the GHDx metadata, citations, and information about where the data were used in GBD.

Additional metadata for each input source will be available through the 2015 version of the citation tool, as required by the GATHER statement.

Appendix Table 1. Country-specific estimates of new HIV infections, counts of PLWH, counts of HIV/AIDS deaths, ART coverage per person LWH in 2015, and ARCs of age-standardized incidence, prevalence, and mortality rates from 2005 to 2015 among women and girls

Location	New infections in thousands (95% UI)	PLWH in thousands (95% UI)	HIV/AIDS deaths in thousands (95% UI)	ART coverage per 100 PLWH (95% UI)	Age-standardized incidence rate ARC from 2005 to 2015 (95% UI)	Age-standardized prevalence rate ARC from 2005 to 2015 (95% UI)	Age-standardized mortality rate ARC from 2005 to 2015 (95% UI)
Global	1243·85 (1130·43 to 1364·47)	20517·80 (19906·15 to 21207·76)	555·78 (521·94 to 591·96)	42·35 (40·97 to 43·73)	-0·02 (-0·03 to -0·01)	0·01 (0·01 to 0·01)	-0·06 (-0·06 to -0·05)
High SDI	20·96 (15·75 to 29·44)	497·87 (404·05 to 619·55)	9·31 (8·80 to 9·90)	52·09 (46·13 to 57·02)	0·01 (-0·01 to 0·03)	0·02 (0·01 to 0·02)	-0·01 (-0·01 to -0·00)
High-middle SDI	319·91 (270·95 to 375·83)	5503·54 (5197·44 to 5839·01)	104·11 (95·44 to 114·98)	50·23 (47·48 to 52·99)	-0·02 (-0·04 to -0·00)	0·02 (0·01 to 0·02)	-0·06 (-0·07 to -0·05)
Middle SDI	128·20 (102·99 to 162·04)	1932·53 (1745·41 to 2276·42)	51·39 (45·21 to 67·17)	40·74 (36·37 to 44·06)	-0·00 (-0·02 to 0·01)	0·02 (0·01 to 0·03)	-0·04 (-0·05 to -0·02)
Low-middle SDI	434·49 (355·07 to 521·77)	6617·77 (6257·14 to 7019·47)	203·58 (177·83 to 228·07)	37·17 (34·75 to 39·51)	-0·01 (-0·03 to 0·01)	0·00 (-0·00 to 0·01)	-0·07 (-0·08 to -0·06)
Low SDI	339·69 (284·99 to 397·31)	5954·07 (5680·61 to 6259·16)	187·08 (169·14 to 205·68)	40·57 (38·32 to 42·83)	-0·05 (-0·07 to -0·03)	-0·00 (-0·01 to 0·00)	-0·08 (-0·09 to -0·07)
High-income	10·65 (8·81 to 12·78)	370·96 (306·55 to 447·29)	3·69 (3·63 to 3·74)	64·73 (62·32 to 67·46)	-0·01 (-0·02 to 0·00)	0·00 (-0·00 to 0·01)	-0·05 (-0·05 to -0·05)
High-income North America	5·77 (4·38 to 7·54)	198·88 (157·39 to 254·79)	2·22 (2·18 to 2·25)	66·60 (62·95 to 70·53)	-0·02 (-0·04 to -0·00)	0·00 (-0·00 to 0·01)	-0·07 (-0·07 to -0·06)
Canada	0·18 (0·03 to 0·45)	8·02 (2·70 to 16·94)	0·07 (0·07 to 0·08)	62·57 (53·43 to 73·10)	-0·02 (-0·14 to 0·02)	-0·01 (-0·02 to 0·01)	-0·06 (-0·07 to -0·05)
Greenland	0·00 (0·00 to 0·00)	0·05 (0·01 to 0·13)	0·00 (0·00 to 0·00)	57·60 (48·82 to 66·78)	-0·10 (-0·60 to -0·02)	-0·01 (-0·02 to 0·01)	-0·02 (-0·06 to 0·02)
United States	5·59 (4·26 to 7·31)	190·79 (149·49 to 245·11)	2·14 (2·11 to 2·18)	66·76 (62·94 to 70·86)	-0·02 (-0·04 to -0·00)	0·00 (-0·00 to 0·01)	-0·07 (-0·07 to -0·06)
Australasia	0·05 (0·02 to 0·11)	2·26 (0·97 to 4·23)	0·02 (0·02 to 0·02)	59·51 (53·45 to 65·35)	-0·02 (-0·04 to -0·01)	-0·00 (-0·01 to 0·01)	-0·01 (-0·02 to -0·00)
Australia	0·04 (0·02 to 0·10)	1·85 (0·62 to 3·90)	0·02 (0·02 to 0·02)	59·83 (52·88 to 66·45)	-0·02 (-0·03 to -0·00)	-0·00 (-0·01 to 0·01)	-0·02 (-0·03 to -0·01)
New Zealand	0·01 (0·00 to 0·02)	0·41 (0·12 to 0·92)	0·00 (0·00 to 0·00)	57·88 (49·60 to 66·13)	-0·03 (-0·10 to -0·00)	0·00 (-0·01 to 0·02)	0·04 (0·02 to 0·05)
High-income Asia Pacific	0·22 (0·15 to 0·31)	6·65 (4·21 to 11·48)	0·08 (0·08 to 0·08)	45·83 (41·30 to 50·03)	-0·04 (-0·10 to -0·00)	0·02 (0·01 to 0·04)	-0·02 (-0·02 to -0·02)
Brunei	0·00 (0·00 to 0·01)	0·06 (0·02 to 0·15)	0·00 (0·00 to 0·00)	35·18 (26·48 to 45·98)	-0·02 (-0·15 to 0·02)	0·03 (0·00 to 0·04)	-0·01 (-0·05 to 0·02)
Japan	0·13 (0·10 to 0·16)	2·78 (2·25 to 3·40)	0·06 (0·06 to 0·06)	55·32 (53·05 to 57·88)	0·01 (-0·00 to 0·02)	0·04 (0·04 to 0·05)	-0·02 (-0·03 to -0·02)
Singapore	0·02 (0·01 to 0·03)	0·56 (0·19 to 1·20)	0·00 (0·00 to 0·00)	50·27 (40·11 to 61·27)	0·01 (-0·05 to 0·05)	0·02 (-0·00 to 0·04)	0·01 (-0·00 to 0·01)
South Korea	0·07 (0·01 to 0·16)	3·25 (0·98 to 7·95)	0·02 (0·01 to 0·02)	35·17 (26·72 to 43·25)	-0·11 (-0·32 to -0·04)	0·01 (-0·01 to 0·03)	-0·01 (-0·01 to -0·00)
Western Europe	2·70 (2·03 to 3·55)	139·72 (97·19 to 192·88)	0·78 (0·75 to 0·80)	62·02 (58·56 to 65·48)	-0·03 (-0·04 to -0·02)	-0·00 (-0·01 to 0·00)	-0·05 (-0·06 to -0·05)
Andorra	0·00 (0·00 to 0·00)	0·04 (0·00 to 0·24)	0·00 (0·00 to 0·00)	54·70 (29·58 to 79·36)	-0·03 (-0·62 to 0·09)	0·01 (-0·03 to 0·08)	-0·00 (-0·08 to 0·08)
Austria	0·06 (0·02 to 0·14)	2·42 (0·58 to 6·29)	0·01 (0·01 to 0·01)	53·29 (45·47 to 61·40)	-0·04 (-0·09 to -0·00)	0·01 (-0·00 to 0·02)	-0·02 (-0·03 to -0·01)
Belgium	0·06 (0·02 to 0·14)	3·26 (0·91 to 7·77)	0·02 (0·02 to 0·02)	60·20 (52·41 to 67·24)	-0·02 (-0·12 to 0·00)	-0·00 (-0·02 to 0·01)	-0·04 (-0·05 to -0·03)
Cyprus	0·00 (0·00 to 0·01)	0·13 (0·04 to 0·30)	0·00 (0·00 to 0·00)	45·48 (36·67 to 56·18)	-0·05 (-0·63 to 0·02)	0·02 (-0·01 to 0·04)	0·01 (-0·03 to 0·04)
Denmark	0·02 (0·00 to 0·05)	1·28 (0·34 to 2·52)	0·01 (0·00 to 0·01)	60·28 (51·90 to 68·35)	-0·05 (-0·18 to -0·01)	-0·00 (-0·01 to 0·01)	-0·06 (-0·07 to -0·05)
Finland	0·01 (0·00 to 0·02)	0·29 (0·08 to 0·66)	0·00 (0·00 to 0·00)	55·53 (47·72 to 63·14)	-0·06 (-0·19 to -0·02)	0·01 (-0·01 to 0·02)	0·00 (-0·01 to 0·01)
France	0·21 (0·08 to 0·46)	17·21 (5·22 to 37·91)	0·12 (0·11 to 0·13)	60·93 (49·82 to 69·56)	-0·04 (-0·08 to -0·02)	-0·02 (-0·04 to -0·01)	-0·07 (-0·08 to -0·06)
Germany	0·32 (0·11 to 0·67)	11·22 (3·48 to 24·68)	0·09 (0·08 to 0·09)	53·68 (44·15 to 63·03)	-0·01 (-0·03 to 0·01)	0·02 (0·00 to 0·03)	-0·03 (-0·04 to -0·03)
Greece	0·01 (0·01 to 0·02)	0·33 (0·16 to 0·57)	0·00 (0·00 to 0·00)	43·56 (32·43 to 53·91)	0·01 (-0·02 to 0·03)	0·02 (0·00 to 0·03)	0·03 (0·02 to 0·04)
Iceland	0·00 (0·00 to 0·00)	0·04 (0·01 to 0·09)	0·00 (0·00 to 0·00)	47·50 (37·71 to 58·60)	-0·01 (-0·18 to 0·03)	0·01 (-0·01 to 0·03)	-0·04 (-0·05 to -0·03)
Ireland	0·02 (0·00 to 0·04)	0·79 (0·21 to 1·82)	0·00 (0·00 to 0·00)	56·62 (48·97 to 64·63)	-0·03 (-0·14 to 0·00)	-0·00 (-0·02 to 0·01)	-0·06 (-0·07 to -0·04)
Israel	0·09 (0·03 to 0·19)	2·57 (0·73 to 5·55)	0·01 (0·01 to 0·01)	48·21 (40·42 to 56·94)	-0·01 (-0·09 to 0·01)	0·01 (-0·00 to 0·03)	-0·05 (-0·06 to -0·04)
Italy	0·41 (0·15 to 0·88)	31·00 (10·08 to 65·65)	0·14 (0·13 to 0·15)	65·88 (58·04 to 72·12)	-0·05 (-0·07 to -0·03)	-0·01 (-0·02 to 0·00)	-0·04 (-0·05 to -0·03)
Luxembourg	0·01 (0·00 to 0·01)	0·21 (0·06 to 0·48)	0·00 (0·00 to 0·00)	50·11 (39·72 to 61·22)	0·00 (-0·09 to 0·03)	0·01 (-0·01 to 0·02)	-0·04 (-0·05 to -0·03)
Malta	0·00 (0·00 to 0·00)	0·05 (0·02 to 0·12)	0·00 (0·00 to 0·00)	44·47 (35·03 to 55·42)	0·02 (-0·08 to 0·05)	0·03 (0·00 to 0·05)	-0·04 (-0·05 to -0·03)
Netherlands	0·04 (0·01 to 0·09)	2·63 (0·75 to 5·84)	0·01 (0·01 to 0·01)	67·74 (59·78 to 74·77)	-0·01 (-0·07 to 0·01)	-0·01 (-0·03 to -0·00)	-0·06 (-0·07 to -0·05)
Norway	0·02 (0·01 to 0·05)	1·29 (0·36 to 2·91)	0·00 (0·00 to 0·00)	61·66 (53·52 to 69·21)	-0·02 (-0·09 to -0·00)	-0·01 (-0·02 to 0·00)	-0·10 (-0·12 to -0·09)

Appendix Table 1. Country-specific estimates of new HIV infections, counts of PLWH, counts of HIV/AIDS deaths, ART coverage per person LWH in 2015, and ARCs of age-standardized incidence, prevalence, and mortality rates from 2005 to 2015 among women and girls

Location	New infections in thousands (95% UI)	PLWH in thousands (95% UI)	HIV/AIDS deaths in thousands (95% UI)	ART coverage per 100 PLWH (95% UI)	Age-standardized incidence rate ARC from 2005 to 2015 (95% UI)	Age-standardized prevalence rate ARC from 2005 to 2015 (95% UI)	Age-standardized mortality rate ARC from 2005 to 2015 (95% UI)
Portugal	0·37 (0·09 to 0·85)	20·93 (5·66 to 48·51)	0·10 (0·10 to 0·11)	58·99 (50·01 to 66·91)	-0·03 (-0·13 to -0·01)	-0·01 (-0·02 to 0·01)	-0·07 (-0·08 to -0·06)
Spain	0·41 (0·16 to 0·87)	24·75 (7·39 to 55·47)	0·16 (0·15 to 0·18)	64·30 (52·96 to 73·32)	0·01 (-0·01 to 0·02)	-0·02 (-0·03 to -0·01)	-0·07 (-0·09 to -0·06)
Sweden	0·02 (0·01 to 0·04)	0·88 (0·39 to 1·60)	0·01 (0·01 to 0·01)	73·66 (67·13 to 80·63)	-0·01 (-0·06 to 0·01)	0·00 (-0·01 to 0·01)	-0·07 (-0·07 to -0·06)
Switzerland	0·06 (0·01 to 0·14)	4·11 (1·18 to 8·81)	0·01 (0·01 to 0·01)	68·14 (60·53 to 74·81)	0·00 (-0·09 to 0·03)	-0·01 (-0·03 to -0·00)	-0·06 (-0·07 to -0·05)
United Kingdom	0·55 (0·43 to 0·69)	14·10 (10·91 to 17·86)	0·07 (0·07 to 0·08)	58·90 (56·00 to 61·67)	-0·03 (-0·04 to -0·03)	0·03 (0·02 to 0·03)	-0·02 (-0·03 to -0·02)
Southern Latin America	1·90 (0·91 to 2·75)	23·46 (14·74 to 35·13)	0·59 (0·56 to 0·63)	69·93 (63·84 to 76·35)	0·05 (-0·04 to 0·08)	0·03 (0·01 to 0·04)	-0·01 (-0·02 to -0·00)
Argentina	1·72 (0·71 to 2·55)	19·35 (10·87 to 31·05)	0·48 (0·45 to 0·52)	73·08 (66·03 to 79·79)	0·06 (-0·05 to 0·09)	0·03 (0·01 to 0·04)	-0·01 (-0·02 to -0·01)
Chile	0·10 (0·06 to 0·17)	2·42 (1·07 to 4·92)	0·08 (0·07 to 0·08)	49·88 (37·36 to 61·41)	-0·05 (-0·08 to -0·01)	0·00 (-0·01 to 0·01)	0·01 (0·00 to 0·02)
Uruguay	0·09 (0·05 to 0·15)	1·69 (0·79 to 3·23)	0·03 (0·03 to 0·03)	59·97 (49·31 to 71·70)	-0·01 (-0·05 to 0·03)	0·02 (-0·00 to 0·03)	-0·02 (-0·03 to -0·01)
Central Europe, Eastern Europe, and Central Asia	16·16 (11·37 to 24·76)	237·47 (161·91 to 358·29)	8·23 (7·70 to 8·88)	29·38 (24·70 to 35·85)	0·01 (-0·02 to 0·04)	0·03 (0·02 to 0·05)	0·02 (0·01 to 0·02)
Eastern Europe	14·75 (10·04 to 23·14)	213·63 (140·82 to 333·90)	7·69 (7·18 to 8·29)	27·83 (22·74 to 35·05)	0·01 (-0·02 to 0·05)	0·04 (0·02 to 0·05)	0·02 (0·02 to 0·03)
Belarus	0·24 (0·13 to 0·41)	3·73 (1·85 to 6·31)	0·07 (0·05 to 0·10)	41·86 (34·63 to 51·14)	0·01 (-0·03 to 0·05)	0·05 (0·03 to 0·07)	-0·01 (-0·04 to 0·01)
Estonia	0·02 (0·01 to 0·04)	0·38 (0·19 to 0·68)	0·01 (0·01 to 0·01)	39·43 (33·10 to 46·20)	-0·02 (-0·04 to 0·00)	0·05 (0·04 to 0·07)	-0·02 (-0·04 to -0·00)
Latvia	0·08 (0·02 to 0·16)	1·40 (0·66 to 2·83)	0·05 (0·04 to 0·05)	11·59 (8·01 to 16·83)	-0·05 (-0·16 to 0·00)	0·00 (-0·03 to 0·04)	0·05 (0·03 to 0·07)
Lithuania	0·05 (0·01 to 0·11)	1·05 (0·49 to 2·05)	0·04 (0·03 to 0·04)	13·58 (9·71 to 20·03)	-0·06 (-0·22 to -0·00)	-0·00 (-0·04 to 0·03)	-0·03 (-0·05 to -0·01)
Moldova	0·09 (0·05 to 0·16)	1·63 (0·76 to 2·98)	0·04 (0·03 to 0·04)	26·45 (19·36 to 34·91)	-0·01 (-0·03 to 0·01)	0·04 (0·02 to 0·05)	-0·02 (-0·04 to -0·00)
Russia	9·99 (5·47 to 18·48)	122·39 (61·79 to 226·99)	5·10 (4·67 to 5·62)	21·10 (16·60 to 26·50)	0·05 (0·01 to 0·10)	0·06 (0·04 to 0·07)	0·05 (0·04 to 0·06)
Ukraine	4·27 (2·99 to 6·00)	83·04 (47·29 to 136·65)	2·39 (2·11 to 2·73)	36·23 (28·20 to 47·05)	-0·04 (-0·06 to -0·03)	0·02 (0·01 to 0·02)	-0·01 (-0·02 to 0·01)
Central Europe	0·35 (0·22 to 0·47)	6·20 (4·33 to 8·40)	0·11 (0·10 to 0·13)	46·74 (41·44 to 52·88)	0·01 (-0·03 to 0·03)	0·03 (0·01 to 0·04)	-0·03 (-0·04 to -0·02)
Albania	0·00 (0·00 to 0·00)	0·02 (0·01 to 0·04)	0·00 (0·00 to 0·00)	45·88 (32·17 to 63·28)	-0·06 (-0·52 to 0·04)	0·01 (-0·03 to 0·04)	0·01 (-0·03 to 0·05)
Bosnia and Herzegovina	0·00 (0·00 to 0·01)	0·05 (0·02 to 0·12)	0·00 (0·00 to 0·00)	48·24 (34·50 to 63·79)	-0·06 (-0·50 to 0·04)	0·01 (-0·03 to 0·04)	0·00 (-0·04 to 0·06)
Bulgaria	0·02 (0·01 to 0·05)	0·40 (0·18 to 0·84)	0·01 (0·01 to 0·01)	24·43 (18·05 to 32·21)	-0·01 (-0·07 to 0·03)	0·02 (-0·01 to 0·04)	-0·02 (-0·03 to -0·00)
Croatia	0·00 (0·00 to 0·01)	0·12 (0·05 to 0·22)	0·00 (0·00 to 0·00)	53·80 (41·95 to 67·50)	-0·01 (-0·12 to 0·01)	0·02 (-0·01 to 0·03)	0·09 (0·07 to 0·11)
Czech Republic	0·01 (0·00 to 0·02)	0·24 (0·12 to 0·40)	0·01 (0·00 to 0·01)	54·85 (46·43 to 64·10)	0·01 (-0·10 to 0·05)	0·03 (0·00 to 0·04)	0·06 (0·05 to 0·08)
Hungary	0·01 (0·01 to 0·02)	0·27 (0·14 to 0·47)	0·01 (0·01 to 0·01)	47·37 (37·54 to 58·68)	-0·02 (-0·05 to -0·00)	-0·01 (-0·02 to 0·00)	-0·06 (-0·08 to -0·04)
Macedonia	0·00 (0·00 to 0·01)	0·04 (0·01 to 0·08)	0·00 (0·00 to 0·00)	40·45 (28·26 to 58·63)	-0·06 (-0·51 to 0·04)	0·03 (-0·02 to 0·07)	0·03 (-0·02 to 0·08)
Montenegro	0·00 (0·00 to 0·00)	0·02 (0·01 to 0·04)	0·00 (0·00 to 0·00)	42·45 (29·48 to 59·10)	-0·04 (-0·51 to 0·07)	0·02 (-0·02 to 0·06)	0·02 (-0·03 to 0·07)
Poland	0·10 (0·03 to 0·16)	2·08 (1·04 to 3·42)	0·03 (0·03 to 0·04)	57·33 (49·01 to 67·99)	-0·00 (-0·11 to 0·03)	0·02 (-0·01 to 0·03)	-0·02 (-0·04 to -0·01)
Romania	0·17 (0·06 to 0·27)	2·63 (1·27 to 4·45)	0·03 (0·03 to 0·04)	41·83 (31·97 to 53·24)	0·03 (-0·08 to 0·06)	0·05 (0·01 to 0·06)	-0·06 (-0·07 to -0·04)
Serbia	0·01 (0·00 to 0·02)	0·23 (0·10 to 0·63)	0·01 (0·01 to 0·03)	24·53 (19·63 to 29·72)	-0·08 (-0·11 to -0·05)	0·00 (-0·01 to 0·02)	0·09 (0·04 to 0·14)
Slovakia	0·00 (0·00 to 0·01)	0·07 (0·03 to 0·13)	0·00 (0·00 to 0·00)	46·90 (36·44 to 57·59)	0·02 (0·00 to 0·04)	0·04 (0·02 to 0·06)	-0·01 (-0·04 to 0·02)
Slovenia	0·00 (0·00 to 0·00)	0·02 (0·01 to 0·05)	0·00 (0·00 to 0·00)	60·37 (46·78 to 73·46)	0·01 (-0·01 to 0·04)	0·01 (-0·01 to 0·02)	-0·14 (-0·16 to -0·12)
Central Asia	1·07 (0·71 to 1·51)	17·64 (12·27 to 25·36)	0·43 (0·35 to 0·59)	40·69 (33·98 to 49·86)	-0·02 (-0·06 to 0·02)	0·01 (-0·00 to 0·03)	-0·06 (-0·08 to -0·04)
Armenia	0·02 (0·01 to 0·03)	0·14 (0·07 to 0·26)	0·00 (0·00 to 0·00)	32·99 (26·13 to 40·85)	0·08 (-0·02 to 0·26)	0·08 (0·04 to 0·13)	0·02 (-0·04 to 0·07)
Azerbaijan	0·08 (0·03 to 0·12)	0·95 (0·43 to 1·73)	0·02 (0·01 to 0·03)	39·25 (29·17 to 53·31)	0·03 (-0·07 to 0·09)	0·03 (0·00 to 0·05)	-0·08 (-0·11 to -0·03)
Georgia	0·05 (0·03 to 0·08)	0·59 (0·33 to 0·95)	0·01 (0·01 to 0·01)	37·29 (31·31 to 43·97)	0·04 (-0·01 to 0·09)	0·13 (0·11 to 0·15)	0·11 (0·08 to 0·14)
Kazakhstan	0·36 (0·19 to 0·59)	4·47 (1·98 to 8·10)	0·09 (0·08 to 0·11)	33·56 (27·37 to 42·12)	0·09 (0·06 to 0·13)	0·05 (0·03 to 0·06)	-0·05 (-0·07 to -0·03)

Appendix Table 1. Country-specific estimates of new HIV infections, counts of PLWH, counts of HIV/AIDS deaths, ART coverage per person LWH in 2015, and ARCs of age-standardized incidence, prevalence, and mortality rates from 2005 to 2015 among women and girls

Location	New infections in thousands (95% UI)	PLWH in thousands (95% UI)	HIV/AIDS deaths in thousands (95% UI)	ART coverage per 100 PLWH (95% UI)	Age-standardized incidence rate ARC from 2005 to 2015 (95% UI)	Age-standardized prevalence rate ARC from 2005 to 2015 (95% UI)	Age-standardized mortality rate ARC from 2005 to 2015 (95% UI)
Kyrgyzstan	0·07 (0·04 to 0·13)	1·67 (0·74 to 3·18)	0·04 (0·02 to 0·07)	64·30 (53·53 to 74·77)	-0·07 (-0·14 to -0·01)	0·05 (0·03 to 0·06)	-0·03 (-0·07 to 0·01)
Mongolia	0·00 (0·00 to 0·00)	0·03 (0·01 to 0·06)	0·00 (0·00 to 0·00)	34·93 (23·34 to 53·01)	0·03 (-0·12 to 0·17)	-0·01 (-0·05 to 0·02)	-0·09 (-0·13 to -0·05)
Tajikistan	0·17 (0·07 to 0·32)	2·45 (1·12 to 4·79)	0·09 (0·07 to 0·16)	22·31 (16·48 to 30·19)	-0·02 (-0·12 to 0·06)	-0·00 (-0·03 to 0·02)	-0·04 (-0·07 to -0·01)
Turkmenistan	0·22 (0·03 to 0·53)	2·85 (0·99 to 5·95)	0·07 (0·04 to 0·12)	29·62 (19·52 to 48·07)	0·01 (-0·18 to 0·15)	0·03 (-0·04 to 0·07)	-0·04 (-0·09 to 0·01)
Uzbekistan	0·10 (0·03 to 0·22)	4·49 (2·09 to 10·08)	0·11 (0·06 to 0·24)	53·41 (40·14 to 67·51)	-0·17 (-0·29 to -0·09)	-0·04 (-0·05 to -0·03)	-0·12 (-0·16 to -0·06)
Latin America and Caribbean	27·12 (23·88 to 30·92)	466·76 (421·05 to 521·75)	13·54 (12·33 to 14·89)	46·77 (45·02 to 48·66)	-0·01 (-0·02 to 0·00)	0·01 (0·01 to 0·02)	-0·04 (-0·05 to -0·03)
Central Latin America	7·00 (5·85 to 8·35)	103·05 (85·29 to 124·60)	3·08 (2·98 to 3·21)	41·00 (38·80 to 43·03)	0·01 (-0·00 to 0·03)	0·03 (0·02 to 0·03)	-0·01 (-0·02 to -0·01)
Colombia	1·39 (0·75 to 2·30)	18·32 (8·94 to 32·49)	0·58 (0·54 to 0·62)	33·79 (27·29 to 41·67)	0·03 (0·00 to 0·07)	0·03 (0·01 to 0·04)	-0·02 (-0·02 to -0·01)
Costa Rica	0·07 (0·05 to 0·11)	1·51 (0·74 to 2·52)	0·03 (0·03 to 0·04)	39·35 (33·85 to 44·73)	-0·04 (-0·05 to -0·02)	0·02 (0·01 to 0·03)	0·02 (0·02 to 0·03)
El Salvador	0·25 (0·14 to 0·38)	5·61 (2·72 to 9·54)	0·12 (0·08 to 0·17)	54·16 (48·01 to 60·34)	-0·05 (-0·07 to -0·03)	0·02 (0·01 to 0·03)	-0·01 (-0·05 to 0·02)
Guatemala	0·60 (0·30 to 1·10)	11·04 (5·22 to 19·92)	0·23 (0·21 to 0·24)	47·04 (41·07 to 53·41)	-0·03 (-0·10 to 0·03)	0·01 (-0·01 to 0·02)	-0·06 (-0·07 to -0·05)
Honduras	0·86 (0·51 to 1·33)	11·73 (7·07 to 18·06)	0·31 (0·25 to 0·39)	40·84 (35·48 to 46·72)	0·01 (-0·04 to 0·06)	0·02 (-0·01 to 0·04)	-0·03 (-0·07 to 0·01)
Mexico	2·32 (2·07 to 2·59)	34·04 (29·21 to 39·32)	1·14 (1·12 to 1·16)	43·09 (41·30 to 45·18)	0·01 (0·00 to 0·02)	0·03 (0·03 to 0·04)	-0·01 (-0·02 to -0·01)
Nicaragua	0·27 (0·13 to 0·48)	2·37 (1·19 to 4·03)	0·04 (0·03 to 0·06)	28·38 (24·46 to 32·80)	0·09 (0·04 to 0·14)	0·09 (0·07 to 0·12)	0·00 (-0·03 to 0·04)
Panama	0·44 (0·23 to 0·78)	5·08 (2·64 to 8·57)	0·10 (0·07 to 0·15)	43·34 (37·36 to 49·55)	0·12 (0·04 to 0·23)	0·03 (0·01 to 0·04)	-0·03 (-0·06 to 0·01)
Venezuela	0·80 (0·33 to 1·41)	13·33 (6·98 to 23·69)	0·54 (0·50 to 0·57)	34·95 (29·85 to 41·41)	-0·01 (-0·10 to 0·03)	0·02 (0·01 to 0·04)	0·02 (0·01 to 0·03)
Andean Latin America	1·00 (0·70 to 1·41)	14·08 (9·12 to 21·00)	0·38 (0·32 to 0·47)	38·31 (34·16 to 43·04)	0·01 (-0·01 to 0·03)	0·03 (0·02 to 0·04)	-0·03 (-0·04 to -0·02)
Bolivia	0·05 (0·03 to 0·09)	0·69 (0·32 to 1·28)	0·02 (0·02 to 0·03)	24·03 (19·82 to 28·85)	0·02 (-0·02 to 0·06)	0·02 (0·00 to 0·05)	-0·02 (-0·04 to 0·01)
Ecuador	0·52 (0·29 to 0·85)	6·57 (3·45 to 11·01)	0·14 (0·10 to 0·20)	40·88 (36·00 to 46·57)	0·02 (-0·01 to 0·06)	0·05 (0·02 to 0·06)	-0·02 (-0·05 to 0·00)
Peru	0·43 (0·25 to 0·68)	6·82 (3·57 to 11·48)	0·22 (0·17 to 0·29)	36·95 (30·75 to 43·90)	-0·00 (-0·03 to 0·03)	0·02 (0·00 to 0·03)	-0·03 (-0·05 to -0·02)
Caribbean	8·21 (5·63 to 11·34)	154·52 (134·63 to 175·34)	4·62 (3·65 to 5·52)	48·47 (43·94 to 52·99)	-0·02 (-0·05 to 0·01)	-0·00 (-0·01 to 0·01)	-0·09 (-0·10 to -0·07)
Antigua and Barbuda	0·01 (0·00 to 0·02)	0·12 (0·05 to 0·25)	0·00 (0·00 to 0·00)	41·33 (31·23 to 51·43)	0·01 (-0·07 to 0·13)	0·01 (-0·01 to 0·06)	-0·04 (-0·05 to -0·03)
The Bahamas	0·04 (0·02 to 0·06)	1·23 (0·62 to 2·01)	0·03 (0·02 to 0·05)	50·09 (41·02 to 61·00)	-0·07 (-0·10 to -0·03)	0·00 (-0·01 to 0·02)	-0·06 (-0·08 to -0·04)
Barbados	0·02 (0·01 to 0·03)	0·38 (0·17 to 0·66)	0·01 (0·01 to 0·01)	47·73 (39·28 to 56·46)	0·02 (-0·02 to 0·05)	0·01 (0·00 to 0·02)	-0·04 (-0·04 to -0·03)
Belize	0·07 (0·04 to 0·11)	1·24 (0·66 to 2·13)	0·03 (0·02 to 0·05)	61·00 (54·90 to 67·09)	0·01 (-0·02 to 0·05)	0·01 (-0·00 to 0·02)	-0·02 (-0·07 to 0·02)
Bermuda	0·01 (0·00 to 0·01)	0·11 (0·05 to 0·24)	0·00 (0·00 to 0·00)	42·49 (31·09 to 52·82)	0·00 (-0·07 to 0·13)	0·01 (-0·01 to 0·05)	-0·05 (-0·06 to -0·04)
Cuba	0·27 (0·16 to 0·43)	4·64 (2·30 to 7·60)	0·07 (0·07 to 0·08)	51·46 (46·27 to 56·80)	-0·00 (-0·03 to 0·02)	0·09 (0·07 to 0·11)	0·04 (0·03 to 0·05)
Dominica	0·00 (0·00 to 0·01)	0·08 (0·03 to 0·16)	0·00 (0·00 to 0·00)	39·63 (29·41 to 48·67)	0·01 (-0·08 to 0·15)	0·02 (-0·00 to 0·07)	-0·02 (-0·05 to 0·03)
Dominican Republic	1·10 (0·77 to 1·54)	24·98 (21·17 to 28·61)	0·46 (0·26 to 0·74)	48·58 (41·23 to 57·90)	0·01 (-0·05 to 0·11)	-0·02 (-0·03 to -0·01)	-0·15 (-0·20 to -0·11)
Grenada	0·01 (0·00 to 0·02)	0·13 (0·05 to 0·27)	0·00 (0·00 to 0·01)	35·26 (26·07 to 43·86)	0·00 (-0·08 to 0·12)	0·02 (-0·00 to 0·06)	-0·01 (-0·04 to 0·04)
Guyana	0·45 (0·24 to 0·70)	7·63 (3·47 to 13·56)	0·13 (0·08 to 0·21)	63·86 (54·52 to 71·93)	-0·01 (-0·03 to 0·01)	0·03 (0·01 to 0·06)	0·01 (-0·02 to 0·04)
Haiti	5·39 (2·96 to 8·54)	94·12 (78·70 to 111·21)	3·38 (2·48 to 4·24)	46·54 (40·54 to 53·30)	-0·03 (-0·08 to 0·01)	-0·01 (-0·03 to 0·00)	-0·10 (-0·12 to -0·08)
Jamaica	0·22 (0·14 to 0·34)	4·22 (2·05 to 6·68)	0·10 (0·07 to 0·15)	42·14 (36·24 to 48·34)	-0·00 (-0·03 to 0·03)	0·01 (-0·01 to 0·02)	-0·05 (-0·07 to -0·03)
Puerto Rico	0·13 (0·05 to 0·33)	4·14 (1·53 to 9·83)	0·08 (0·07 to 0·08)	53·19 (38·58 to 66·58)	-0·02 (-0·10 to 0·12)	-0·02 (-0·04 to 0·02)	-0·06 (-0·07 to -0·05)
Saint Lucia	0·01 (0·00 to 0·02)	0·15 (0·06 to 0·33)	0·00 (0·00 to 0·00)	39·46 (29·20 to 49·04)	0·01 (-0·08 to 0·14)	0·02 (-0·01 to 0·06)	-0·04 (-0·05 to -0·04)
Saint Vincent and the Grenadines	0·02 (0·01 to 0·04)	0·31 (0·13 to 0·64)	0·01 (0·01 to 0·01)	38·23 (27·88 to 47·03)	0·01 (-0·08 to 0·14)	0·02 (-0·00 to 0·06)	-0·04 (-0·04 to -0·03)
Suriname	0·07 (0·04 to 0·12)	1·49 (0·69 to 2·68)	0·04 (0·03 to 0·07)	44·70 (35·62 to 52·62)	-0·01 (-0·04 to 0·02)	-0·00 (-0·02 to 0·01)	-0·00 (-0·03 to 0·03)

Appendix Table 1. Country-specific estimates of new HIV infections, counts of PLWH, counts of HIV/AIDS deaths, ART coverage per person LWH in 2015, and ARCs of age-standardized incidence, prevalence, and mortality rates from 2005 to 2015 among women and girls

Location	New infections in thousands (95% UI)	PLWH in thousands (95% UI)	HIV/AIDS deaths in thousands (95% UI)	ART coverage per 100 PLWH (95% UI)	Age-standardized incidence rate ARC from 2005 to 2015 (95% UI)	Age-standardized prevalence rate ARC from 2005 to 2015 (95% UI)	Age-standardized mortality rate ARC from 2005 to 2015 (95% UI)
Trinidad and Tobago	0·11 (0·06 to 0·18)	3·31 (1·54 to 6·08)	0·08 (0·08 to 0·09)	51·43 (41·85 to 60·49)	-0·05 (-0·07 to -0·03)	-0·00 (-0·01 to 0·01)	-0·05 (-0·06 to -0·04)
Virgin Islands, U·S·	0·00 (0·00 to 0·01)	0·09 (0·04 to 0·18)	0·00 (0·00 to 0·00)	44·78 (33·26 to 55·06)	0·01 (-0·08 to 0·15)	0·02 (-0·00 to 0·06)	-0·01 (-0·05 to 0·03)
Tropical Latin America	10·91 (9·65 to 12·29)	195·11 (158·64 to 241·81)	5·46 (4·70 to 6·42)	49·02 (47·25 to 50·87)	-0·01 (-0·01 to 0·00)	0·02 (0·01 to 0·02)	0·01 (0·00 to 0·02)
Brazil	10·55 (9·36 to 11·95)	191·24 (154·55 to 237·54)	5·38 (4·63 to 6·35)	49·32 (47·58 to 51·17)	-0·01 (-0·01 to -0·00)	0·02 (0·01 to 0·02)	0·01 (-0·00 to 0·02)
Paraguay	0·36 (0·14 to 0·73)	3·87 (1·80 to 7·16)	0·08 (0·05 to 0·11)	34·38 (28·03 to 43·16)	0·02 (-0·04 to 0·06)	0·07 (0·04 to 0·09)	0·02 (-0·01 to 0·05)
Southeast Asia, East Asia, and Oceania	49·96 (33·88 to 79·75)	687·42 (496·76 to 1058·07)	24·69 (18·91 to 40·69)	27·87 (21·36 to 36·39)	0·01 (-0·02 to 0·04)	0·05 (0·03 to 0·07)	0·05 (0·00 to 0·08)
East Asia	16·52 (11·81 to 22·61)	247·00 (181·89 to 327·62)	12·38 (10·95 to 13·92)	18·12 (16·10 to 20·67)	-0·01 (-0·03 to 0·01)	0·04 (0·03 to 0·05)	0·08 (0·07 to 0·09)
China	16·18 (11·60 to 22·27)	242·48 (176·95 to 320·84)	12·15 (10·70 to 13·62)	18·11 (16·07 to 20·57)	-0·01 (-0·03 to 0·01)	0·04 (0·03 to 0·05)	0·08 (0·07 to 0·09)
North Korea	0·28 (0·03 to 0·94)	3·69 (0·73 to 13·67)	0·19 (0·04 to 0·76)	17·74 (6·80 to 34·86)	0·01 (-0·14 to 0·11)	0·05 (-0·01 to 0·13)	0·09 (-0·00 to 0·17)
Taiwan	0·06 (0·02 to 0·12)	0·83 (0·32 to 1·73)	0·04 (0·03 to 0·06)	17·18 (11·62 to 23·33)	-0·01 (-0·10 to 0·03)	0·05 (0·01 to 0·07)	0·09 (0·06 to 0·12)
Southeast Asia	32·91 (17·50 to 62·58)	430·48 (257·25 to 785·33)	12·11 (6·75 to 27·96)	33·19 (22·34 to 46·43)	0·01 (-0·03 to 0·06)	0·05 (0·02 to 0·08)	0·02 (-0·05 to 0·08)
Cambodia	2·37 (1·03 to 4·52)	26·11 (10·13 to 49·83)	0·71 (0·48 to 1·00)	28·77 (21·73 to 35·36)	0·04 (0·00 to 0·07)	0·02 (0·01 to 0·04)	0·02 (-0·01 to 0·04)
Indonesia	14·70 (2·77 to 41·06)	149·52 (29·60 to 479·71)	5·45 (0·98 to 20·63)	11·38 (7·83 to 15·65)	0·03 (-0·03 to 0·10)	0·12 (0·07 to 0·17)	0·18 (0·12 to 0·24)
Laos	0·15 (0·04 to 0·46)	2·22 (0·54 to 7·35)	0·05 (0·01 to 0·18)	35·16 (25·63 to 44·55)	-0·04 (-0·09 to 0·05)	0·07 (0·03 to 0·12)	0·07 (0·01 to 0·13)
Malaysia	0·26 (0·19 to 0·38)	6·53 (3·63 to 11·61)	0·20 (0·14 to 0·30)	49·99 (40·54 to 59·30)	-0·07 (-0·08 to -0·06)	-0·00 (-0·01 to 0·00)	-0·01 (-0·04 to 0·01)
Maldives	0·00 (0·00 to 0·00)	0·01 (0·01 to 0·02)	0·00 (0·00 to 0·00)	7·54 (5·17 to 11·00)	-0·00 (-0·03 to 0·01)	-0·02 (-0·04 to 0·00)	-0·03 (-0·05 to -0·01)
Mauritius	0·02 (0·01 to 0·04)	0·32 (0·19 to 0·54)	0·01 (0·01 to 0·02)	22·85 (17·87 to 28·00)	0·00 (-0·02 to 0·03)	0·05 (0·04 to 0·07)	0·11 (0·09 to 0·12)
Myanmar	1·11 (0·24 to 3·21)	35·92 (7·90 to 140·95)	0·87 (0·14 to 3·68)	66·53 (53·60 to 80·15)	-0·08 (-0·13 to -0·00)	0·01 (-0·03 to 0·06)	-0·07 (-0·16 to -0·01)
Philippines	9·63 (3·60 to 22·99)	80·08 (35·59 to 138·46)	1·08 (1·00 to 1·16)	27·24 (20·15 to 36·23)	0·09 (0·03 to 0·18)	0·10 (0·07 to 0·12)	-0·01 (-0·02 to -0·00)
Sri Lanka	0·08 (0·04 to 0·14)	0·87 (0·39 to 1·62)	0·02 (0·02 to 0·02)	31·09 (27·33 to 35·03)	0·07 (0·03 to 0·11)	0·04 (0·02 to 0·06)	0·00 (-0·01 to 0·01)
Seychelles	0·00 (0·00 to 0·01)	0·06 (0·02 to 0·13)	0·00 (0·00 to 0·00)	33·37 (18·49 to 49·54)	-0·01 (-0·38 to 0·15)	0·02 (-0·03 to 0·10)	-0·00 (-0·04 to 0·04)
Thailand	2·97 (0·82 to 6·51)	99·38 (48·09 to 175·81)	3·09 (2·10 to 4·70)	50·56 (43·88 to 62·52)	-0·07 (-0·19 to 0·01)	-0·00 (-0·02 to 0·01)	-0·01 (-0·05 to 0·02)
Timor-Leste	0·04 (0·00 to 0·17)	0·61 (0·01 to 2·86)	0·02 (0·00 to 0·13)	27·86 (10·21 to 53·21)	0·01 (-0·37 to 0·27)	0·05 (-0·06 to 0·20)	0·03 (-0·09 to 0·16)
Vietnam	1·52 (0·31 to 4·39)	28·24 (5·67 to 110·16)	0·58 (0·11 to 2·53)	42·42 (31·16 to 54·18)	-0·03 (-0·08 to 0·05)	0·05 (0·01 to 0·10)	-0·01 (-0·07 to 0·06)
Oceania	0·53 (0·38 to 0·73)	9·95 (7·98 to 12·23)	0·20 (0·14 to 0·31)	55·95 (50·00 to 62·08)	-0·04 (-0·07 to -0·01)	0·03 (0·01 to 0·05)	-0·08 (-0·10 to -0·05)
American Samoa	0·00 (0·00 to 0·00)	0·01 (0·00 to 0·01)	0·00 (0·00 to 0·00)	29·33 (22·95 to 38·59)	0·04 (-0·06 to 0·09)	0·05 (0·01 to 0·08)	0·01 (-0·02 to 0·04)
Federated States of Micronesia	0·01 (0·00 to 0·02)	0·06 (0·01 to 0·24)	0·00 (0·00 to 0·01)	24·52 (13·91 to 45·93)	0·07 (-0·10 to 0·20)	0·07 (-0·03 to 0·18)	0·02 (-0·07 to 0·13)
Fiji	0·04 (0·02 to 0·07)	0·39 (0·18 to 0·71)	0·01 (0·01 to 0·02)	23·88 (20·58 to 27·89)	0·06 (0·03 to 0·07)	0·05 (0·04 to 0·07)	0·04 (0·02 to 0·05)
Guam	0·00 (0·00 to 0·01)	0·04 (0·02 to 0·08)	0·00 (0·00 to 0·00)	29·76 (22·41 to 40·10)	0·04 (-0·05 to 0·09)	0·05 (0·02 to 0·08)	0·00 (-0·02 to 0·04)
Kiribati	0·00 (0·00 to 0·00)	0·01 (0·00 to 0·01)	0·00 (0·00 to 0·00)	29·28 (21·61 to 39·53)	0·03 (-0·06 to 0·08)	0·04 (0·00 to 0·07)	-0·02 (-0·04 to 0·01)
Marshall Islands	0·00 (0·00 to 0·01)	0·05 (0·01 to 0·23)	0·00 (0·00 to 0·01)	25·61 (14·75 to 47·93)	0·07 (-0·10 to 0·19)	0·07 (-0·04 to 0·19)	0·02 (-0·08 to 0·14)
Northern Mariana Islands	0·00 (0·00 to 0·00)	0·01 (0·01 to 0·03)	0·00 (0·00 to 0·00)	20·70 (15·91 to 29·14)	0·03 (-0·06 to 0·08)	0·06 (0·02 to 0·09)	0·02 (-0·01 to 0·05)
Papua New Guinea	0·38 (0·25 to 0·57)	8·00 (6·38 to 9·41)	0·14 (0·10 to 0·20)	59·75 (53·47 to 66·33)	-0·06 (-0·10 to -0·02)	0·03 (0·01 to 0·05)	-0·10 (-0·12 to -0·07)
Samoa	0·01 (0·00 to 0·03)	0·11 (0·02 to 0·52)	0·00 (0·00 to 0·02)	26·80 (15·37 to 48·13)	0·07 (-0·10 to 0·20)	0·07 (-0·03 to 0·18)	0·02 (-0·07 to 0·13)
Solomon Islands	0·03 (0·01 to 0·09)	0·38 (0·05 to 1·72)	0·01 (0·00 to 0·08)	24·86 (14·41 to 44·40)	0·08 (-0·10 to 0·20)	0·07 (-0·03 to 0·19)	0·02 (-0·07 to 0·13)
Tonga	0·00 (0·00 to 0·01)	0·03 (0·01 to 0·06)	0·00 (0·00 to 0·00)	22·20 (17·47 to 31·33)	0·08 (-0·02 to 0·14)	0·10 (0·05 to 0·14)	0·06 (0·02 to 0·09)
Vanuatu	0·01 (0·00 to 0·05)	0·18 (0·02 to 0·77)	0·01 (0·00 to 0·04)	25·15 (14·48 to 46·16)	0·07 (-0·09 to 0·20)	0·07 (-0·04 to 0·19)	0·02 (-0·08 to 0·14)

Appendix Table 1. Country-specific estimates of new HIV infections, counts of PLWH, counts of HIV/AIDS deaths, ART coverage per person LWH in 2015, and ARCs of age-standardized incidence, prevalence, and mortality rates from 2005 to 2015 among women and girls

Location	New infections in thousands (95% UI)	PLWH in thousands (95% UI)	HIV/AIDS deaths in thousands (95% UI)	ART coverage per 100 PLWH (95% UI)	Age-standardized incidence rate ARC from 2005 to 2015 (95% UI)	Age-standardized prevalence rate ARC from 2005 to 2015 (95% UI)	Age-standardized mortality rate ARC from 2005 to 2015 (95% UI)
North Africa and Middle East	3·85 (2·67 to 5·46)	45·03 (36·49 to 56·59)	2·06 (1·70 to 2·56)	23·61 (19·56 to 27·66)	-0·02 (-0·05 to 0·01)	0·03 (0·00 to 0·04)	0·02 (-0·01 to 0·03)
North Africa and Middle East	3·85 (2·67 to 5·46)	45·03 (36·49 to 56·59)	2·06 (1·70 to 2·56)	23·61 (19·56 to 27·66)	-0·02 (-0·05 to 0·01)	0·03 (0·00 to 0·04)	0·02 (-0·01 to 0·03)
Afghanistan	0·20 (0·04 to 0·59)	1·30 (0·28 to 3·99)	0·06 (0·01 to 0·22)	4·19 (3·04 to 6·01)	0·08 (0·01 to 0·16)	0·06 (-0·04 to 0·15)	0·03 (-0·10 to 0·12)
Algeria	0·14 (0·00 to 0·44)	3·15 (1·07 to 5·47)	0·09 (0·05 to 0·15)	66·01 (58·02 to 74·07)	-0·12 (-0·47 to 0·04)	0·04 (-0·02 to 0·07)	-0·00 (-0·04 to 0·04)
Bahrain	0·01 (0·00 to 0·02)	0·07 (0·03 to 0·14)	0·00 (0·00 to 0·00)	21·27 (16·01 to 27·67)	0·04 (-0·08 to 0·11)	0·04 (-0·00 to 0·07)	-0·02 (-0·04 to 0·02)
Egypt	0·42 (0·21 to 0·76)	3·07 (1·48 to 5·28)	0·09 (0·07 to 0·12)	16·58 (14·03 to 19·55)	0·09 (0·06 to 0·13)	0·09 (0·07 to 0·11)	0·04 (0·02 to 0·06)
Iran	0·12 (0·06 to 0·23)	1·41 (0·71 to 2·62)	0·04 (0·03 to 0·06)	36·59 (32·34 to 41·42)	0·02 (-0·02 to 0·07)	0·04 (0·02 to 0·06)	0·01 (-0·03 to 0·06)
Iraq	0·20 (0·05 to 0·42)	1·47 (0·67 to 2·78)	0·04 (0·03 to 0·05)	17·68 (13·49 to 23·68)	0·07 (-0·07 to 0·14)	0·09 (0·04 to 0·13)	0·05 (0·02 to 0·09)
Jordan	0·01 (0·00 to 0·03)	0·15 (0·06 to 0·30)	0·01 (0·00 to 0·01)	21·70 (16·44 to 28·40)	0·02 (-0·13 to 0·09)	0·02 (-0·01 to 0·06)	-0·01 (-0·04 to 0·03)
Kuwait	0·01 (0·00 to 0·01)	0·06 (0·03 to 0·13)	0·00 (0·00 to 0·00)	21·21 (16·09 to 28·16)	0·03 (-0·11 to 0·09)	0·02 (-0·02 to 0·05)	-0·06 (-0·08 to -0·04)
Lebanon	0·04 (0·01 to 0·12)	0·53 (0·11 to 2·22)	0·03 (0·01 to 0·14)	20·91 (11·57 to 48·85)	0·02 (-0·04 to 0·09)	0·01 (-0·07 to 0·08)	0·03 (-0·07 to 0·09)
Libya	0·10 (0·01 to 0·42)	1·16 (0·07 to 5·14)	0·05 (0·00 to 0·23)	21·32 (14·00 to 30·75)	0·04 (-0·14 to 0·16)	0·06 (-0·05 to 0·16)	0·02 (-0·09 to 0·12)
Morocco	0·31 (0·17 to 0·52)	4·15 (1·99 to 7·46)	0·16 (0·12 to 0·21)	24·06 (21·90 to 26·38)	-0·00 (-0·03 to 0·02)	0·04 (0·03 to 0·05)	0·08 (0·05 to 0·11)
Palestine	0·02 (0·01 to 0·04)	0·17 (0·08 to 0·33)	0·01 (0·00 to 0·01)	18·81 (14·35 to 23·86)	0·03 (-0·07 to 0·10)	0·06 (0·03 to 0·10)	0·03 (0·00 to 0·07)
Oman	0·03 (0·02 to 0·04)	0·40 (0·22 to 0·63)	0·01 (0·01 to 0·01)	44·27 (38·11 to 50·80)	-0·02 (-0·05 to 0·01)	0·03 (0·01 to 0·04)	-0·02 (-0·05 to 0·02)
Qatar	0·00 (0·00 to 0·00)	0·02 (0·01 to 0·05)	0·00 (0·00 to 0·00)	21·81 (17·01 to 28·42)	0·01 (-0·11 to 0·08)	-0·02 (-0·04 to 0·01)	-0·07 (-0·09 to -0·03)
Saudi Arabia	0·37 (0·18 to 0·72)	4·05 (2·05 to 8·86)	0·14 (0·07 to 0·37)	23·99 (19·66 to 29·53)	0·02 (-0·05 to 0·07)	0·03 (-0·02 to 0·07)	-0·00 (-0·06 to 0·04)
Sudan	1·31 (0·42 to 2·59)	17·68 (13·03 to 23·35)	1·20 (0·90 to 1·49)	14·62 (10·51 to 19·85)	-0·09 (-0·19 to -0·02)	-0·00 (-0·04 to 0·02)	0·02 (-0·01 to 0·04)
Syria	0·02 (0·01 to 0·04)	0·38 (0·08 to 0·97)	0·01 (0·00 to 0·03)	18·85 (15·19 to 23·55)	0·10 (-0·02 to 0·14)	0·12 (0·01 to 0·21)	0·06 (-0·02 to 0·15)
Tunisia	0·08 (0·04 to 0·15)	0·81 (0·36 to 1·42)	0·02 (0·01 to 0·03)	29·22 (25·42 to 33·55)	0·05 (0·00 to 0·09)	0·08 (0·06 to 0·10)	0·08 (0·05 to 0·11)
Turkey	0·23 (0·09 to 0·42)	2·71 (1·24 to 4·69)	0·05 (0·04 to 0·07)	32·12 (25·93 to 39·87)	0·01 (-0·05 to 0·05)	0·08 (0·05 to 0·10)	0·02 (-0·02 to 0·04)
United Arab Emirates	0·07 (0·00 to 0·27)	0·78 (0·05 to 3·61)	0·03 (0·00 to 0·15)	18·89 (12·67 to 27·38)	0·04 (-0·15 to 0·16)	0·06 (-0·04 to 0·16)	0·02 (-0·09 to 0·12)
Yemen	0·15 (0·03 to 0·43)	1·44 (0·33 to 4·83)	0·04 (0·01 to 0·17)	45·47 (36·12 to 55·77)	0·03 (-0·04 to 0·11)	0·05 (-0·04 to 0·12)	-0·05 (-0·17 to 0·06)
South Asia	116·84 (97·20 to 140·92)	1662·43 (1548·15 to 1788·64)	80·32 (75·24 to 85·39)	16·69 (15·40 to 17·97)	-0·01 (-0·03 to 0·01)	-0·03 (-0·03 to -0·02)	-0·04 (-0·05 to -0·04)
South Asia	116·84 (97·20 to 140·92)	1 to662·43 (1548·15 to 1788·64)	80·32 (75·24 to 85·39)	16·69 (15·40 to 17·97)	-0·01 (-0·03 to 0·01)	-0·03 (-0·03 to -0·02)	-0·04 (-0·05 to -0·04)
Bangladesh	0·19 (0·04 to 0·55)	2·57 (0·53 to 8·71)	0·09 (0·01 to 0·40)	15·70 (12·16 to 20·67)	-0·02 (-0·07 to 0·06)	0·09 (0·05 to 0·14)	0·13 (0·08 to 0·19)
Bhutan	0·03 (0·01 to 0·09)	0·33 (0·07 to 1·14)	0·01 (0·00 to 0·03)	27·95 (18·32 to 42·90)	0·02 (-0·05 to 0·09)	0·04 (-0·03 to 0·12)	-0·03 (-0·17 to 0·07)
India	113·86 (95·09 to 137·53)	1 to636·81 (1528·68 to 1761·06)	79·40 (74·34 to 84·17)	16·67 (15·42 to 17·95)	-0·01 (-0·02 to 0·01)	-0·03 (-0·03 to -0·02)	-0·04 (-0·05 to -0·04)
Nepal	0·35 (0·07 to 1·02)	9·38 (2·04 to 35·95)	0·42 (0·07 to 1·72)	34·72 (25·51 to 44·64)	-0·11 (-0·17 to -0·04)	0·01 (-0·02 to 0·05)	0·03 (-0·02 to 0·08)
Pakistan	2·41 (0·46 to 7·07)	13·34 (2·70 to 40·07)	0·40 (0·06 to 1·38)	5·80 (3·80 to 9·12)	0·15 (0·07 to 0·24)	0·16 (0·07 to 0·24)	0·14 (0·01 to 0·24)
Sub-Saharan Africa	1019·26 (915·53 to 1130·26)	17047·72 (16510·29 to 17595·00)	423·25 (390·34 to 454·48)	45·06 (43·49 to 46·70)	-0·04 (-0·05 to -0·02)	0·00 (0·00 to 0·01)	-0·09 (-0·10 to -0·08)
Southern Sub-Saharan Africa	386·23 (327·26 to 455·84)	6618·90 (6291·24 to 6959·71)	115·29 (104·80 to 127·84)	52·64 (50·02 to 55·28)	-0·03 (-0·04 to -0·01)	0·02 (0·01 to 0·02)	-0·09 (-0·10 to -0·08)
Botswana	12·61 (7·66 to 18·43)	240·73 (215·23 to 267·94)	3·56 (2·01 to 4·92)	64·48 (56·46 to 72·20)	-0·01 (-0·06 to 0·04)	0·01 (0·00 to 0·02)	-0·09 (-0·13 to -0·06)
Lesotho	13·43 (9·43 to 18·59)	201·06 (177·46 to 225·87)	6·30 (4·42 to 8·41)	39·94 (35·24 to 44·86)	-0·02 (-0·05 to 0·01)	0·02 (0·01 to 0·03)	-0·04 (-0·07 to -0·02)
Namibia	7·89 (5·55 to 10·37)	149·52 (135·93 to 163·04)	2·30 (1·49 to 3·21)	54·68 (47·52 to 62·47)	-0·03 (-0·06 to 0·00)	0·01 (0·01 to 0·02)	-0·11 (-0·15 to -0·08)
South Africa	286·44 (237·36 to 341·33)	4902·72 (4597·27 to 5209·30)	78·81 (70·39 to 89·28)	52·78 (49·68 to 55·88)	-0·03 (-0·05 to -0·01)	0·02 (0·02 to 0·02)	-0·08 (-0·09 to -0·07)
Swaziland	7·45 (4·83 to 9·76)	149·26 (135·20 to 163·09)	2·90 (2·03 to 3·88)	55·00 (48·20 to 62·11)	-0·06 (-0·10 to -0·03)	0·02 (0·01 to 0·03)	-0·08 (-0·11 to -0·06)

Appendix Table 1. Country-specific estimates of new HIV infections, counts of PLWH, counts of HIV/AIDS deaths, ART coverage per person LWH in 2015, and ARCs of age-standardized incidence, prevalence, and mortality rates from 2005 to 2015 among women and girls

Location	New infections in thousands (95% UI)	PLWH in thousands (95% UI)	HIV/AIDS deaths in thousands (95% UI)	ART coverage per 100 PLWH (95% UI)	Age-standardized incidence rate ARC from 2005 to 2015 (95% UI)	Age-standardized prevalence rate ARC from 2005 to 2015 (95% UI)	Age-standardized mortality rate ARC from 2005 to 2015 (95% UI)
Zimbabwe	58·41 (29·88 to 97·92)	975·60 (860·84 to 1	21·42 (16·22 to 28·36)	51·09 (44·13 to 58·96)	-0·01 (-0·08 to 0·06)	-0·01 (-0·01 to 0·00)	-0·14 (-0·16 to -0·11)
Western Sub-Saharan Africa	246·12 (183·17 to 319·40)	3700·44 (3408·55 to 4036·75)	122·11 (97·32 to 145·12)	33·45 (29·96 to 37·04)	-0·03 (-0·06 to -0·00)	0·01 (-0·00 to 0·01)	-0·05 (-0·08 to -0·04)
Benin	3·04 (2·01 to 4·53)	47·80 (40·96 to 55·42)	1·27 (0·77 to 1·73)	43·24 (37·80 to 49·34)	-0·02 (-0·07 to 0·03)	0·00 (-0·01 to 0·01)	-0·10 (-0·15 to -0·08)
Burkina Faso	3·39 (1·79 to 5·41)	60·27 (49·61 to 72·58)	1·49 (1·06 to 1·97)	52·40 (44·77 to 61·90)	-0·01 (-0·08 to 0·05)	-0·02 (-0·04 to -0·01)	-0·17 (-0·19 to -0·15)
Cameroon	27·22 (15·87 to 42·55)	383·79 (326·60 to 445·41)	16·70 (11·47 to 22·27)	24·67 (20·38 to 29·36)	-0·03 (-0·08 to 0·01)	0·00 (-0·01 to 0·02)	-0·03 (-0·06 to -0·01)
Cape Verde	0·09 (0·04 to 0·26)	1·29 (0·97 to 1·73)	0·01 (0·01 to 0·03)	50·87 (36·28 to 66·89)	-0·01 (-0·08 to 0·12)	0·05 (0·02 to 0·08)	-0·09 (-0·16 to -0·03)
Chad	5·10 (1·95 to 9·69)	96·74 (74·43 to 124·58)	4·92 (3·02 to 6·71)	34·79 (26·16 to 45·55)	-0·09 (-0·20 to -0·02)	-0·01 (-0·03 to 0·00)	-0·05 (-0·09 to -0·03)
Cote d'Ivoire	22·81 (13·13 to 33·77)	312·93 (261·89 to 364·75)	10·04 (7·49 to 13·47)	34·14 (27·67 to 41·08)	-0·02 (-0·06 to 0·03)	-0·00 (-0·02 to 0·01)	-0·08 (-0·10 to -0·06)
The Gambia	0·52 (0·20 to 0·95)	10·79 (8·59 to 13·65)	0·31 (0·18 to 0·47)	25·67 (19·82 to 32·01)	-0·11 (-0·21 to -0·03)	0·02 (0·00 to 0·03)	-0·02 (-0·05 to 0·00)
Ghana	9·84 (5·56 to 15·18)	167·88 (140·75 to 198·60)	5·67 (3·26 to 8·05)	39·47 (32·29 to 47·33)	-0·04 (-0·09 to 0·01)	-0·02 (-0·03 to -0·01)	-0·09 (-0·15 to -0·07)
Guinea	4·89 (2·14 to 8·01)	77·85 (62·10 to 94·69)	2·54 (1·89 to 3·49)	27·75 (23·13 to 32·86)	-0·05 (-0·12 to 0·00)	0·01 (-0·01 to 0·03)	-0·04 (-0·06 to -0·02)
Guinea-Bissau	1·07 (0·38 to 2·07)	24·26 (19·95 to 29·02)	0·89 (0·49 to 1·37)	30·33 (26·18 to 34·71)	-0·11 (-0·21 to -0·03)	0·03 (0·01 to 0·05)	0·01 (-0·03 to 0·06)
Liberia	1·48 (0·69 to 2·56)	21·43 (17·90 to 25·95)	1·14 (0·88 to 1·47)	24·76 (19·46 to 30·13)	-0·02 (-0·10 to 0·08)	-0·04 (-0·05 to -0·02)	-0·07 (-0·09 to -0·05)
Mali	6·29 (3·23 to 10·04)	86·02 (64·29 to 109·56)	3·27 (2·38 to 4·56)	26·79 (22·64 to 31·33)	-0·03 (-0·09 to 0·00)	0·01 (-0·01 to 0·02)	-0·03 (-0·05 to -0·02)
Mauritania	0·07 (0·01 to 0·24)	3·87 (0·77 to 16·61)	0·22 (0·04 to 1·12)	33·27 (21·34 to 45·48)	-0·19 (-0·34 to -0·07)	-0·03 (-0·07 to 0·00)	-0·02 (-0·07 to 0·02)
Niger	1·59 (0·47 to 3·32)	39·85 (32·78 to 48·41)	1·92 (1·63 to 2·28)	33·58 (27·07 to 40·73)	-0·11 (-0·23 to -0·03)	-0·06 (-0·08 to -0·04)	-0·09 (-0·11 to -0·06)
Nigeria	150·85 (91·31 to 221·26)	2216·68 (1946·52 to 2521·16)	66·08 (42·17 to 88·49)	34·18 (28·55 to 40·07)	-0·02 (-0·07 to 0·01)	0·02 (0·00 to 0·03)	-0·05 (-0·09 to -0·02)
Sao Tome and Principe	0·00 (0·00 to 0·00)	0·01 (0·01 to 0·01)	0·00 (0·00 to 0·00)	55·38 (48·95 to 62·90)	-0·01 (-0·08 to 0·03)	0·04 (0·02 to 0·06)	-0·04 (-0·07 to -0·02)
Senegal	2·55 (0·77 to 4·29)	40·73 (31·79 to 50·42)	1·31 (0·75 to 1·71)	52·21 (44·15 to 61·53)	-0·04 (-0·16 to -0·00)	0·00 (-0·02 to 0·02)	-0·05 (-0·11 to -0·02)
Sierra Leone	2·37 (0·70 to 4·16)	36·31 (29·34 to 43·38)	1·42 (1·06 to 1·96)	21·62 (16·35 to 28·39)	-0·06 (-0·18 to 0·00)	0·01 (-0·01 to 0·03)	-0·01 (-0·03 to 0·01)
Togo	2·93 (1·43 to 5·02)	71·90 (61·94 to 82·46)	2·90 (2·09 to 3·85)	34·05 (28·63 to 39·93)	-0·09 (-0·17 to -0·02)	-0·03 (-0·04 to -0·02)	-0·07 (-0·09 to -0·05)
Eastern Sub-Saharan Africa	345·73 (293·54 to 402·33)	6 to59·09 (5789·30 to 6358·23)	152·96 (136·89 to 170·46)	45·92 (43·55 to 48·31)	-0·04 (-0·06 to -0·02)	-0·00 (-0·00 to 0·00)	-0·11 (-0·12 to -0·10)
Burundi	3·82 (1·97 to 6·64)	64·84 (53·55 to 77·10)	1·58 (1·08 to 2·24)	40·45 (33·05 to 49·25)	-0·01 (-0·09 to 0·06)	-0·02 (-0·03 to -0·00)	-0·14 (-0·16 to -0·11)
Comoros	0·02 (0·00 to 0·06)	0·13 (0·03 to 0·47)	0·00 (0·00 to 0·02)	21·34 (12·44 to 33·67)	0·01 (-0·09 to 0·12)	0·02 (-0·04 to 0·08)	0·02 (-0·06 to 0·09)
Djibouti	0·29 (0·10 to 0·57)	4·14 (2·78 to 6·04)	0·20 (0·09 to 0·28)	25·21 (20·39 to 30·49)	-0·02 (-0·10 to 0·05)	-0·02 (-0·04 to 0·01)	-0·05 (-0·12 to 0·02)
Eritrea	0·84 (0·39 to 1·41)	12·22 (9·14 to 16·37)	0·44 (0·26 to 0·69)	36·97 (25·52 to 50·46)	-0·00 (-0·08 to 0·07)	-0·01 (-0·04 to 0·01)	-0·09 (-0·13 to -0·05)
Ethiopia	22·26 (11·08 to 35·62)	444·46 (375·65 to 527·02)	14·78 (10·52 to 19·05)	52·01 (45·04 to 59·78)	0·02 (-0·10 to 0·12)	-0·04 (-0·05 to -0·03)	-0·16 (-0·18 to -0·13)
Kenya	76·79 (62·69 to 93·71)	1096·33 (1034·23 to 1155·72)	22·56 (20·44 to 24·95)	42·79 (40·36 to 45·13)	0·06 (0·04 to 0·09)	-0·00 (-0·01 to 0·00)	-0·14 (-0·15 to -0·13)
Madagascar	1·10 (0·18 to 3·43)	23·85 (4·91 to 92·61)	2·37 (0·41 to 10·20)	1·31 (0·73 to 2·35)	-0·13 (-0·22 to -0·03)	-0·05 (-0·13 to 0·02)	-0·02 (-0·10 to 0·05)
Malawi	30·53 (13·81 to 44·83)	647·56 (556·31 to 731·23)	12·58 (8·91 to 16·84)	54·69 (46·23 to 64·34)	-0·07 (-0·15 to -0·02)	-0·00 (-0·02 to 0·00)	-0·14 (-0·17 to -0·12)
Mozambique	68·16 (41·23 to 97·77)	1099·59 (950·74 to 1256·40)	35·19 (27·41 to 43·56)	33·95 (29·31 to 39·30)	-0·06 (-0·11 to -0·02)	0·02 (0·01 to 0·03)	-0·03 (-0·06 to -0·00)
Rwanda	4·71 (2·62 to 7·18)	124·49 (108·79 to 140·64)	2·27 (1·42 to 3·05)	60·84 (52·91 to 68·83)	-0·08 (-0·14 to -0·03)	-0·00 (-0·01 to 0·01)	-0·15 (-0·20 to -0·13)
Somalia	0·92 (0·39 to 1·77)	13·82 (9·36 to 19·44)	0·85 (0·62 to 1·14)	9·33 (5·95 to 14·59)	-0·08 (-0·19 to -0·00)	-0·01 (-0·05 to 0·02)	0·00 (-0·03 to 0·03)
South Sudan	5·41 (1·98 to 9·45)	70·30 (45·60 to 100·00)	4·74 (2·65 to 6·76)	10·43 (6·83 to 15·99)	-0·05 (-0·14 to 0·01)	-0·00 (-0·04 to 0·04)	0·00 (-0·04 to 0·05)
Tanzania	49·37 (29·78 to 77·37)	875·12 (757·84 to 1002·18)	22·56 (14·42 to 32·08)	49·36 (41·94 to 57·32)	-0·04 (-0·08 to 0·00)	-0·00 (-0·02 to 0·01)	-0·12 (-0·16 to -0·09)
Uganda	43·67 (19·50 to 70·63)	853·98 (738·03 to 992·71)	15·77 (11·03 to 24·04)	48·25 (41·34 to 56·07)	-0·07 (-0·15 to -0·01)	0·03 (0·01 to 0·04)	-0·10 (-0·12 to -0·07)
Zambia	37·63 (26·40 to 49·06)	723·96 (651·61 to 795·41)	16·95 (13·60 to 20·77)	55·00 (47·48 to 62·60)	-0·06 (-0·10 to -0·03)	0·01 (0·01 to 0·02)	-0·11 (-0·13 to -0·09)

Appendix Table 1. Country-specific estimates of new HIV infections, counts of PLWH, counts of HIV/AIDS deaths, ART coverage per person LWH in 2015, and ARCs of age-standardized incidence, prevalence, and mortality rates from 2005 to 2015 among women and girls							
**Location**	**New infections in thousands (95% UI)**	**PLWH in thousands (95% UI)**	**HIV/AIDS deaths in thousands (95% UI)**	**ART coverage per 100 PLWH (95% UI)**	**Age-standardized incidence rate ARC from 2005 to 2015 (95% UI)**	**Age-standardized prevalence rate ARC from 2005 to 2015 (95% UI)**	**Age-standardized mortality rate ARC from 2005 to 2015 (95% UI)**
Central Sub-Saharan Africa	41·17 (25·36 to 67·38)	669·29 (591·81 to 756·16)	32·89 (28·37 to 38·38)	26·90 (23·38 to 30·54)	-0·07 (-0·12 to -0·02)	-0·02 (-0·03 to -0·01)	-0·05 (-0·07 to -0·04)
Angola	12·61 (6·67 to 20·87)	166·47 (132·77 to 207·21)	6·19 (3·54 to 9·29)	29·09 (23·74 to 35·34)	-0·03 (-0·10 to 0·02)	0·03 (0·02 to 0·05)	-0·01 (-0·05 to 0·02)
Central African Republic	5·57 (2·65 to 9·77)	78·26 (64·01 to 95·10)	4·81 (3·76 to 5·83)	20·07 (16·33 to 24·32)	-0·02 (-0·08 to 0·04)	-0·03 (-0·05 to -0·01)	-0·05 (-0·07 to -0·03)
Congo	3·89 (1·94 to 5·94)	55·99 (42·46 to 69·13)	2·45 (1·72 to 3·24)	23·05 (19·03 to 29·76)	-0·03 (-0·07 to 0·01)	0·00 (-0·02 to 0·02)	-0·05 (-0·07 to -0·04)
Democratic Republic of the Congo	18·12 (4·96 to 45·60)	339·93 (279·48 to 415·89)	18·93 (15·40 to 22·96)	25·45 (20·12 to 31·06)	-0·12 (-0·26 to -0·01)	-0·05 (-0·06 to -0·03)	-0·06 (-0·08 to -0·04)
Equatorial Guinea	0·34 (0·08 to 0·87)	13·78 (11·62 to 16·20)	0·35 (0·17 to 0·52)	36·86 (29·03 to 46·57)	-0·18 (-0·34 to -0·06)	0·03 (0·02 to 0·05)	-0·04 (-0·09 to 0·01)
Gabon	0·63 (0·20 to 1·29)	14·86 (12·08 to 18·27)	0·16 (0·10 to 0·22)	80·17 (69·15 to 89·14)	-0·07 (-0·18 to 0·01)	0·00 (-0·02 to 0·02)	-0·14 (-0·17 to -0·11)

Appendix Table 2. Country-specific estimates of new HIV infections, counts of PLWH, counts of HIV/AIDS deaths, ART coverage per person LWH in 2015, and ARCs of age-standardized incidence, prevalence, and mortality rates from 2005 to 2015 among men and boys

Location	New infections in thousands (95% UI)	PLWH in thousands (95% UI)	HIV/AIDS deaths in thousands (95% UI)	ART coverage per 100 PLWH (95% UI)	Age-standardized incidence rate ARC from 2005 to 2015 (95% UI)	Age-standardized prevalence rate ARC from 2005 to 2015 (95% UI)	Age-standardized mortality rate ARC from 2005 to 2015 (95% UI)
Global	1207·07 (1104·56 to 1330·75)	18284·71 (17501·45 to 19305·75)	636·79 (596·50 to 689·53)	38·64 (37·17 to 39·93)	-0·02 (-0·02 to -0·01)	0·00 (-0·00 to 0·00)	-0·05 (-0·06 to -0·04)
High SDI	80·78 (58·79 to 118·47)	1706·31 (1350·16 to 2198·49)	24·20 (22·85 to 25·93)	51·33 (43·08 to 58·01)	0·01 (-0·01 to 0·05)	0·01 (0·00 to 0·02)	-0·02 (-0·02 to -0·01)
High-middle SDI	326·85 (284·02 to 374·12)	4918·41 (4598·99 to 5259·63)	136·05 (126·94 to 147·38)	45·51 (43·26 to 47·99)	-0·02 (-0·03 to -0·01)	0·01 (0·01 to 0·01)	-0·05 (-0·06 to -0·04)
Middle SDI	170·13 (131·83 to 230·98)	2222·91 (1849·66 to 2911·11)	80·18 (65·61 to 116·12)	34·98 (29·65 to 38·56)	0·00 (-0·02 to 0·03)	0·02 (0·01 to 0·03)	-0·01 (-0·03 to 0·01)
Low-middle SDI	361·81 (297·69 to 436·30)	5165·66 (4826·51 to 5645·61)	205·29 (176·55 to 236·25)	33·31 (31·45 to 35·38)	-0·01 (-0·03 to 0·00)	-0·01 (-0·01 to -0·00)	-0·06 (-0·08 to -0·05)
Low SDI	266·85 (225·68 to 311·60)	4259·29 (4031·55 to 4502·42)	190·60 (174·03 to 206·77)	34·15 (32·06 to 36·36)	-0·05 (-0·06 to -0·03)	-0·02 (-0·03 to -0·02)	-0·08 (-0·09 to -0·07)
High-income	35·02 (28·98 to 41·09)	1289·23 (1050·19 to 1551·30)	10·27 (10·12 to 10·41)	67·53 (65·28 to 70·29)	-0·01 (-0·03 to -0·00)	-0·00 (-0·01 to 0·00)	-0·06 (-0·06 to -0·05)
High-income North America	18·39 (14·18 to 23·65)	683·73 (536·76 to 881·49)	5·67 (5·58 to 5·76)	70·81 (67·61 to 74·43)	-0·02 (-0·04 to -0·01)	-0·00 (-0·01 to 0·00)	-0·07 (-0·07 to -0·07)
Canada	0·93 (0·15 to 2·39)	41·22 (13·31 to 85·91)	0·24 (0·22 to 0·26)	64·41 (56·06 to 73·70)	-0·03 (-0·15 to 0·02)	-0·01 (-0·03 to 0·01)	-0·06 (-0·07 to -0·05)
Greenland	0·00 (0·00 to 0·01)	0·18 (0·05 to 0·43)	0·00 (0·00 to 0·00)	63·15 (52·92 to 70·90)	-0·10 (-0·68 to -0·02)	-0·01 (-0·03 to 0·00)	-0·03 (-0·07 to 0·02)
United States	17·45 (13·35 to 22·67)	642·25 (499·65 to 837·41)	5·43 (5·34 to 5·52)	71·19 (67·87 to 75·04)	-0·02 (-0·04 to -0·01)	-0·00 (-0·01 to 0·00)	-0·07 (-0·07 to -0·07)
Australasia	0·40 (0·17 to 0·79)	16·43 (6·44 to 33·09)	0·08 (0·07 to 0·08)	62·61 (57·62 to 67·97)	-0·02 (-0·04 to -0·01)	-0·00 (-0·02 to 0·00)	-0·05 (-0·06 to -0·04)
Australia	0·35 (0·13 to 0·75)	14·39 (4·62 to 30·77)	0·07 (0·06 to 0·07)	62·69 (56·85 to 68·76)	-0·02 (-0·04 to -0·01)	-0·01 (-0·02 to 0·00)	-0·05 (-0·06 to -0·04)
New Zealand	0·05 (0·01 to 0·11)	2·04 (0·58 to 4·48)	0·01 (0·01 to 0·01)	61·21 (54·42 to 68·56)	-0·03 (-0·10 to -0·01)	-0·00 (-0·01 to 0·01)	-0·06 (-0·07 to -0·05)
High-income Asia Pacific	0·53 (0·40 to 0·71)	15·41 (10·49 to 24·49)	0·24 (0·24 to 0·25)	51·76 (47·67 to 55·33)	-0·03 (-0·08 to -0·00)	0·02 (0·01 to 0·03)	-0·00 (-0·01 to -0·00)
Brunei	0·01 (0·00 to 0·02)	0·20 (0·06 to 0·45)	0·00 (0·00 to 0·00)	38·44 (29·10 to 49·44)	-0·03 (-0·16 to 0·01)	0·02 (-0·00 to 0·04)	-0·03 (-0·07 to 0·01)
Japan	0·37 (0·30 to 0·45)	7·63 (6·14 to 9·32)	0·11 (0·11 to 0·11)	58·20 (55·90 to 60·88)	0·01 (-0·00 to 0·02)	0·03 (0·03 to 0·04)	-0·03 (-0·03 to -0·03)
Singapore	0·03 (0·01 to 0·07)	1·29 (0·41 to 2·88)	0·01 (0·01 to 0·01)	56·48 (46·13 to 66·79)	0·01 (-0·05 to 0·05)	0·00 (-0·02 to 0·02)	0·14 (0·14 to 0·15)
South Korea	0·12 (0·01 to 0·28)	6·30 (1·95 to 14·92)	0·12 (0·12 to 0·13)	41·44 (32·89 to 49·35)	-0·12 (-0·33 to -0·04)	0·00 (-0·02 to 0·02)	0·01 (0·01 to 0·02)
Western Europe	10·19 (7·34 to 13·52)	511·66 (347·72 to 708·95)	2·65 (2·57 to 2·72)	64·29 (61·11 to 67·58)	-0·03 (-0·04 to -0·02)	-0·01 (-0·01 to -0·00)	-0·06 (-0·06 to -0·05)
Andorra	0·00 (0·00 to 0·01)	0·17 (0·01 to 1·12)	0·00 (0·00 to 0·01)	58·11 (33·25 to 81·42)	-0·04 (-0·77 to 0·10)	0·01 (-0·04 to 0·08)	-0·01 (-0·09 to 0·07)
Austria	0·25 (0·08 to 0·58)	9·23 (2·14 to 22·88)	0·03 (0·03 to 0·03)	55·62 (48·61 to 63·22)	-0·04 (-0·09 to -0·01)	0·01 (-0·00 to 0·02)	-0·06 (-0·07 to -0·06)
Belgium	0·15 (0·04 to 0·34)	7·42 (2·02 to 17·60)	0·03 (0·03 to 0·04)	62·38 (54·93 to 69·85)	-0·03 (-0·12 to 0·00)	-0·01 (-0·02 to 0·01)	-0·05 (-0·06 to -0·04)
Cyprus	0·01 (0·00 to 0·02)	0·26 (0·07 to 0·59)	0·00 (0·00 to 0·00)	50·02 (41·33 to 61·04)	-0·06 (-0·70 to 0·02)	0·01 (-0·01 to 0·03)	-0·00 (-0·04 to 0·03)
Denmark	0·11 (0·02 to 0·25)	6·39 (1·76 to 12·71)	0·02 (0·02 to 0·02)	63·05 (55·30 to 71·07)	-0·06 (-0·19 to -0·01)	-0·01 (-0·02 to 0·01)	-0·04 (-0·05 to -0·04)
Finland	0·02 (0·00 to 0·06)	1·06 (0·29 to 2·40)	0·01 (0·01 to 0·01)	58·46 (51·32 to 65·80)	-0·06 (-0·19 to -0·02)	0·00 (-0·01 to 0·02)	-0·06 (-0·07 to -0·05)
France	0·75 (0·28 to 1·58)	61·96 (18·39 to 137·04)	0·37 (0·34 to 0·39)	63·96 (54·04 to 71·90)	-0·04 (-0·08 to -0·02)	-0·02 (-0·04 to -0·01)	-0·07 (-0·08 to -0·07)
Germany	1·45 (0·54 to 2·97)	49·33 (14·44 to 105·03)	0·35 (0·33 to 0·37)	55·94 (47·13 to 65·22)	-0·01 (-0·04 to 0·01)	0·01 (-0·00 to 0·02)	-0·03 (-0·04 to -0·03)
Greece	0·04 (0·02 to 0·07)	0·89 (0·42 to 1·64)	0·01 (0·01 to 0·02)	38·15 (28·22 to 49·11)	0·01 (-0·02 to 0·04)	0·01 (-0·01 to 0·02)	-0·03 (-0·04 to -0·02)
Iceland	0·01 (0·00 to 0·01)	0·14 (0·04 to 0·34)	0·00 (0·00 to 0·00)	50·70 (40·20 to 62·30)	-0·01 (-0·18 to 0·03)	0·01 (-0·01 to 0·03)	-0·04 (-0·05 to -0·03)
Ireland	0·04 (0·01 to 0·10)	1·76 (0·49 to 4·10)	0·01 (0·01 to 0·01)	59·32 (51·48 to 67·12)	-0·03 (-0·15 to -0·00)	-0·00 (-0·02 to 0·01)	0·04 (0·02 to 0·05)
Israel	0·08 (0·02 to 0·16)	2·28 (0·64 to 4·92)	0·03 (0·02 to 0·03)	53·95 (46·32 to 62·18)	-0·02 (-0·09 to 0·01)	0·01 (-0·01 to 0·02)	-0·01 (-0·02 to 0·00)
Italy	1·55 (0·60 to 3·36)	106·06 (33·39 to 217·43)	0·47 (0·43 to 0·50)	67·39 (61·39 to 72·70)	-0·05 (-0·07 to -0·03)	-0·01 (-0·02 to -0·00)	-0·02 (-0·03 to -0·01)
Luxembourg	0·01 (0·00 to 0·01)	0·20 (0·05 to 0·48)	0·00 (0·00 to 0·00)	53·97 (44·38 to 64·87)	0·00 (-0·10 to 0·03)	0·00 (-0·02 to 0·02)	-0·05 (-0·06 to -0·04)
Malta	0·01 (0·00 to 0·02)	0·20 (0·06 to 0·46)	0·00 (0·00 to 0·00)	49·29 (39·15 to 61·26)	0·01 (-0·09 to 0·04)	0·02 (-0·01 to 0·03)	-0·02 (-0·03 to -0·01)
Netherlands	0·17 (0·05 to 0·38)	11·93 (3·33 to 26·65)	0·04 (0·04 to 0·04)	69·88 (61·82 to 77·18)	-0·02 (-0·07 to 0·01)	-0·02 (-0·03 to -0·01)	-0·07 (-0·08 to -0·06)
Norway	0·03 (0·01 to 0·06)	1·48 (0·40 to 3·35)	0·01 (0·01 to 0·01)	65·06 (57·36 to 71·83)	-0·03 (-0·09 to -0·00)	-0·01 (-0·02 to 0·00)	-0·07 (-0·08 to -0·06)
Portugal	1·85 (0·43 to 4·06)	94·31 (26·55 to 213·62)	0·43 (0·40 to 0·45)	60·90 (53·66 to 67·52)	-0·04 (-0·13 to -0·01)	-0·01 (-0·02 to 0·00)	-0·07 (-0·08 to -0·07)

Appendix Table 2. Country-specific estimates of new HIV infections, counts of PLWH, counts of HIV/AIDS deaths, ART coverage per person LWH in 2015, and ARCs of age-standardized incidence, prevalence, and mortality rates from 2005 to 2015 among men and boys

Location	New infections in thousands (95% UI)	PLWH in thousands (95% UI)	HIV/AIDS deaths in thousands (95% UI)	ART coverage per 100 PLWH (95% UI)	Age-standardized incidence rate ARC from 2005 to 2015 (95% UI)	Age-standardized prevalence rate ARC from 2005 to 2015 (95% UI)	Age-standardized mortality rate ARC from 2005 to 2015 (95% UI)
Spain	1·94 (0·81 to 3·89)	105·58 (31·90 to 228·23)	0·66 (0·61 to 0·71)	65·77 (55·34 to 74·14)	0·01 (-0·01 to 0·02)	-0·02 (-0·03 to -0·01)	-0·08 (-0·09 to -0·07)
Sweden	0·06 (0·02 to 0·12)	2·81 (1·22 to 5·09)	0·02 (0·01 to 0·02)	76·72 (70·92 to 82·86)	-0·01 (-0·06 to 0·01)	-0·00 (-0·02 to 0·01)	-0·03 (-0·04 to -0·03)
Switzerland	0·14 (0·03 to 0·31)	8·92 (2·58 to 19·61)	0·03 (0·03 to 0·03)	70·04 (62·90 to 76·74)	0·00 (-0·10 to 0·03)	-0·02 (-0·03 to -0·01)	-0·07 (-0·08 to -0·06)
United Kingdom	1·51 (1·21 to 1·87)	38·57 (30·38 to 48·39)	0·14 (0·14 to 0·15)	62·06 (58·96 to 65·07)	-0·04 (-0·05 to -0·03)	0·02 (0·02 to 0·03)	-0·04 (-0·04 to -0·03)
Southern Latin America	5·51 (2·66 to 7·66)	62·00 (40·95 to 91·01)	1·63 (1·55 to 1·72)	61·50 (55·34 to 68·94)	0·04 (-0·05 to 0·07)	0·01 (-0·00 to 0·03)	-0·01 (-0·02 to -0·01)
Argentina	4·61 (1·87 to 6·75)	43·59 (25·05 to 66·78)	1·12 (1·04 to 1·20)	68·20 (61·33 to 76·03)	0·07 (-0·05 to 0·09)	0·03 (0·00 to 0·04)	-0·01 (-0·02 to -0·01)
Chile	0·61 (0·37 to 1·00)	13·84 (6·25 to 28·71)	0·38 (0·36 to 0·41)	45·13 (32·40 to 57·70)	-0·05 (-0·08 to -0·01)	-0·01 (-0·03 to 0·00)	-0·01 (-0·02 to 0·00)
Uruguay	0·29 (0·15 to 0·49)	4·57 (2·06 to 8·64)	0·13 (0·12 to 0·15)	42·07 (34·08 to 51·78)	-0·01 (-0·05 to 0·03)	0·00 (-0·01 to 0·02)	-0·01 (-0·02 to 0·00)
Central Europe, Eastern Europe, and Central Asia	62·09 (41·29 to 101·00)	703·39 (448·42 to 1	20·15 (18·78 to 21·67)	16·87 (14·17 to 20·26)	0·02 (-0·01 to 0·06)	0·03 (0·02 to 0·04)	0·00 (-0·00 to 0·01)
Eastern Europe	58·35 (37·47 to 96·87)	651·26 (401·38 to 1	18·40 (17·07 to 19·92)	15·63 (12·68 to 19·26)	0·03 (-0·01 to 0·07)	0·03 (0·02 to 0·05)	0·01 (0·00 to 0·02)
Belarus	1·13 (0·63 to 1·90)	13·78 (6·81 to 23·78)	0·52 (0·35 to 0·87)	33·66 (25·02 to 45·77)	0·01 (-0·03 to 0·05)	0·04 (0·02 to 0·06)	0·02 (0·00 to 0·06)
Estonia	0·09 (0·05 to 0·15)	1·24 (0·61 to 2·23)	0·03 (0·02 to 0·03)	28·46 (22·62 to 34·80)	-0·02 (-0·04 to 0·00)	0·04 (0·03 to 0·06)	-0·00 (-0·02 to 0·02)
Latvia	0·09 (0·03 to 0·19)	1·53 (0·74 to 3·01)	0·06 (0·05 to 0·07)	21·22 (15·04 to 29·84)	-0·05 (-0·16 to -0·00)	0·00 (-0·03 to 0·04)	0·03 (0·02 to 0·05)
Lithuania	0·03 (0·01 to 0·06)	0·62 (0·31 to 1·16)	0·03 (0·02 to 0·03)	36·25 (28·03 to 46·31)	-0·07 (-0·21 to -0·01)	0·01 (-0·02 to 0·04)	0·11 (0·09 to 0·13)
Moldova	0·44 (0·25 to 0·76)	6·31 (2·89 to 11·75)	0·15 (0·13 to 0·17)	20·13 (13·70 to 28·89)	-0·01 (-0·03 to 0·01)	0·03 (0·01 to 0·04)	-0·03 (-0·05 to -0·02)
Russia	47·34 (27·17 to 86·66)	484·66 (250·22 to 885·69)	12·79 (11·58 to 14·07)	12·09 (9·04 to 15·48)	0·05 (0·01 to 0·10)	0·04 (0·03 to 0·06)	0·02 (0·01 to 0·03)
Ukraine	9·22 (6·68 to 12·86)	143·12 (84·06 to 231·34)	4·83 (4·24 to 5·49)	23·51 (17·62 to 31·27)	-0·04 (-0·06 to -0·02)	0·00 (-0·01 to 0·01)	-0·02 (-0·03 to -0·00)
Central Europe	0·85 (0·58 to 1·11)	13·59 (9·85 to 18·27)	0·30 (0·28 to 0·35)	46·34 (40·63 to 52·50)	0·00 (-0·03 to 0·02)	0·01 (0·00 to 0·02)	-0·04 (-0·05 to -0·03)
Albania	0·00 (0·00 to 0·01)	0·05 (0·02 to 0·10)	0·00 (0·00 to 0·00)	46·47 (32·50 to 64·93)	-0·07 (-0·62 to 0·04)	0·00 (-0·04 to 0·04)	-0·00 (-0·04 to 0·04)
Bosnia and Herzegovina	0·00 (0·00 to 0·01)	0·04 (0·01 to 0·10)	0·00 (0·00 to 0·00)	47·98 (34·11 to 64·84)	-0·06 (-0·49 to 0·04)	-0·00 (-0·04 to 0·03)	-0·01 (-0·04 to 0·05)
Bulgaria	0·11 (0·05 to 0·22)	1·46 (0·65 to 3·07)	0·04 (0·04 to 0·05)	14·96 (10·45 to 20·26)	-0·00 (-0·06 to 0·03)	0·01 (-0·03 to 0·03)	-0·06 (-0·07 to -0·04)
Croatia	0·01 (0·00 to 0·02)	0·21 (0·10 to 0·37)	0·00 (0·00 to 0·01)	52·98 (41·74 to 66·28)	-0·02 (-0·13 to 0·01)	0·01 (-0·02 to 0·03)	-0·01 (-0·03 to 0·00)
Czech Republic	0·03 (0·01 to 0·05)	0·51 (0·24 to 0·83)	0·01 (0·01 to 0·01)	53·27 (44·94 to 62·83)	0·01 (-0·10 to 0·05)	0·02 (-0·01 to 0·03)	0·01 (-0·01 to 0·02)
Hungary	0·05 (0·03 to 0·07)	0·97 (0·52 to 1·57)	0·03 (0·03 to 0·04)	45·27 (35·74 to 56·47)	-0·02 (-0·05 to 0·00)	-0·02 (-0·04 to -0·01)	-0·08 (-0·10 to -0·06)
Macedonia	0·00 (0·00 to 0·01)	0·05 (0·02 to 0·10)	0·00 (0·00 to 0·00)	40·84 (28·45 to 61·27)	-0·06 (-0·55 to 0·04)	0·02 (-0·03 to 0·06)	0·02 (-0·02 to 0·08)
Montenegro	0·00 (0·00 to 0·00)	0·03 (0·01 to 0·05)	0·00 (0·00 to 0·00)	42·35 (29·43 to 62·49)	-0·04 (-0·54 to 0·07)	0·02 (-0·03 to 0·06)	0·01 (-0·03 to 0·07)
Poland	0·31 (0·09 to 0·51)	5·64 (2·83 to 9·23)	0·11 (0·09 to 0·12)	56·30 (47·51 to 67·16)	-0·00 (-0·11 to 0·03)	0·01 (-0·02 to 0·02)	-0·02 (-0·04 to -0·01)
Romania	0·28 (0·09 to 0·44)	3·71 (1·88 to 6·13)	0·06 (0·05 to 0·06)	44·47 (35·67 to 55·76)	0·03 (-0·07 to 0·07)	0·04 (0·01 to 0·06)	-0·06 (-0·07 to -0·04)
Serbia	0·03 (0·01 to 0·06)	0·64 (0·28 to 1·67)	0·04 (0·03 to 0·09)	27·57 (21·07 to 34·17)	-0·08 (-0·12 to -0·05)	-0·00 (-0·02 to 0·01)	0·08 (0·03 to 0·13)
Slovakia	0·01 (0·01 to 0·02)	0·17 (0·07 to 0·29)	0·00 (0·00 to 0·01)	45·83 (36·26 to 55·67)	0·02 (-0·00 to 0·04)	0·03 (0·01 to 0·05)	0·00 (-0·02 to 0·02)
Slovenia	0·01 (0·00 to 0·01)	0·12 (0·05 to 0·22)	0·00 (0·00 to 0·00)	58·33 (43·95 to 72·10)	0·01 (-0·01 to 0·04)	-0·00 (-0·03 to 0·01)	-0·03 (-0·05 to -0·01)
Central Asia	2·89 (1·92 to 4·14)	38·54 (26·78 to 54·53)	1·44 (1·15 to 2·01)	25·83 (21·45 to 31·19)	-0·00 (-0·04 to 0·03)	0·01 (-0·01 to 0·02)	-0·02 (-0·04 to -0·01)
Armenia	0·05 (0·02 to 0·10)	0·43 (0·21 to 0·80)	0·01 (0·01 to 0·02)	17·90 (14·11 to 23·74)	0·07 (-0·03 to 0·26)	0·07 (0·02 to 0·11)	0·06 (0·01 to 0·10)
Azerbaijan	0·29 (0·13 to 0·47)	3·11 (1·44 to 5·64)	0·09 (0·06 to 0·18)	30·86 (21·55 to 46·08)	0·04 (-0·08 to 0·09)	0·02 (-0·01 to 0·05)	-0·06 (-0·09 to -0·02)
Georgia	0·10 (0·05 to 0·17)	1·11 (0·63 to 1·68)	0·03 (0·02 to 0·03)	39·53 (33·64 to 46·28)	0·04 (-0·01 to 0·09)	0·13 (0·10 to 0·15)	0·12 (0·09 to 0·15)
Kazakhstan	1·26 (0·68 to 2·07)	13·22 (6·29 to 24·23)	0·22 (0·19 to 0·26)	21·80 (16·38 to 29·62)	0·09 (0·06 to 0·14)	0·03 (0·01 to 0·05)	-0·06 (-0·08 to -0·04)
Kyrgyzstan	0·25 (0·13 to 0·48)	4·96 (2·25 to 9·67)	0·26 (0·18 to 0·39)	21·38 (17·03 to 26·47)	-0·07 (-0·14 to -0·01)	0·03 (0·01 to 0·05)	0·03 (0·02 to 0·04)
Mongolia	0·00 (0·00 to 0·01)	0·05 (0·02 to 0·11)	0·00 (0·00 to 0·00)	21·21 (13·72 to 33·91)	0·03 (-0·11 to 0·19)	-0·02 (-0·07 to 0·02)	-0·05 (-0·08 to -0·02)
Tajikistan	0·15 (0·07 to 0·28)	2·20 (1·03 to 4·09)	0·08 (0·05 to 0·15)	32·82 (24·30 to 43·33)	-0·03 (-0·13 to 0·05)	-0·01 (-0·03 to 0·01)	-0·07 (-0·10 to -0·03)

Appendix Table 2. Country-specific estimates of new HIV infections, counts of PLWH, counts of HIV/AIDS deaths, ART coverage per person LWH in 2015, and ARCs of age-standardized incidence, prevalence, and mortality rates from 2005 to 2015 among men and boys

Location	New infections in thousands (95% UI)	PLWH in thousands (95% UI)	HIV/AIDS deaths in thousands (95% UI)	ART coverage per 100 PLWH (95% UI)	Age-standardized incidence rate ARC from 2005 to 2015 (95% UI)	Age-standardized prevalence rate ARC from 2005 to 2015 (95% UI)	Age-standardized mortality rate ARC from 2005 to 2015 (95% UI)
Turkmenistan	0·57 (0·07 to 1·44)	6·37 (2·15 to 13·46)	0·28 (0·17 to 0·45)	18·18 (11·42 to 30·38)	0·01 (-0·18 to 0·16)	0·02 (-0·06 to 0·07)	0·00 (-0·04 to 0·05)
Uzbekistan	0·21 (0·07 to 0·46)	7·09 (3·30 to 16·09)	0·47 (0·27 to 0·92)	32·97 (22·86 to 44·46)	-0·16 (-0·29 to -0·08)	-0·05 (-0·07 to -0·04)	-0·04 (-0·07 to -0·00)
Latin America and Caribbean	58·35 (53·25 to 63·90)	855·30 (766·64 to 957·75)	33·27 (30·51 to 36·66)	44·20 (42·59 to 45·82)	-0·00 (-0·01 to 0·01)	0·01 (0·00 to 0·01)	-0·02 (-0·02 to -0·01)
Central Latin America	22·37 (19·05 to 26·00)	291·01 (243·05 to 343·68)	9·24 (8·97 to 9·55)	39·65 (37·76 to 41·68)	0·01 (0·00 to 0·03)	0·02 (0·02 to 0·03)	-0·02 (-0·02 to -0·02)
Colombia	4·76 (2·62 to 7·79)	55·63 (27·93 to 99·05)	1·85 (1·73 to 1·97)	28·42 (22·55 to 35·74)	0·03 (-0·00 to 0·07)	0·02 (0·01 to 0·04)	-0·03 (-0·03 to -0·02)
Costa Rica	0·28 (0·18 to 0·40)	5·14 (2·60 to 8·31)	0·12 (0·11 to 0·12)	53·22 (45·54 to 60·49)	-0·04 (-0·05 to -0·02)	0·01 (0·01 to 0·02)	-0·03 (-0·04 to -0·02)
El Salvador	0·55 (0·32 to 0·84)	10·50 (5·19 to 18·19)	0·43 (0·32 to 0·60)	41·93 (35·81 to 47·52)	-0·05 (-0·07 to -0·03)	0·00 (-0·01 to 0·01)	0·03 (-0·00 to 0·05)
Guatemala	1·07 (0·54 to 1·95)	16·70 (7·67 to 29·50)	0·45 (0·43 to 0·48)	38·71 (32·48 to 44·98)	-0·03 (-0·10 to 0·03)	-0·01 (-0·03 to 0·01)	-0·06 (-0·07 to -0·05)
Honduras	0·62 (0·37 to 0·96)	8·09 (4·96 to 12·13)	0·28 (0·23 to 0·36)	39·76 (34·42 to 45·44)	0·00 (-0·04 to 0·05)	0·01 (-0·01 to 0·03)	-0·02 (-0·05 to 0·00)
Mexico	10·15 (9·15 to 11·28)	135·48 (116·96 to 155·67)	4·03 (3·97 to 4·09)	46·32 (44·36 to 48·44)	0·01 (0·01 to 0·02)	0·03 (0·02 to 0·03)	-0·03 (-0·03 to -0·02)
Nicaragua	0·70 (0·35 to 1·24)	5·56 (2·75 to 9·86)	0·15 (0·12 to 0·20)	19·57 (16·53 to 22·83)	0·09 (0·04 to 0·14)	0·08 (0·05 to 0·11)	0·03 (0·00 to 0·05)
Panama	1·36 (0·73 to 2·38)	13·84 (7·20 to 23·47)	0·41 (0·31 to 0·60)	36·72 (31·04 to 42·55)	0·12 (0·05 to 0·25)	0·02 (0·00 to 0·04)	-0·02 (-0·05 to 0·01)
Venezuela	2·89 (1·30 to 5·02)	40·08 (20·69 to 71·46)	1·51 (1·41 to 1·61)	32·46 (27·37 to 38·07)	-0·01 (-0·09 to 0·03)	0·01 (-0·01 to 0·04)	0·01 (-0·00 to 0·01)
Andean Latin America	2·84 (1·97 to 3·91)	35·23 (22·85 to 51·29)	1·42 (1·19 to 1·79)	33·10 (29·23 to 37·92)	0·01 (-0·01 to 0·04)	0·02 (0·01 to 0·04)	-0·01 (-0·02 to 0·01)
Bolivia	0·14 (0·07 to 0·22)	1·53 (0·69 to 2·88)	0·07 (0·05 to 0·11)	16·26 (13·34 to 19·29)	0·02 (-0·02 to 0·07)	0·01 (-0·01 to 0·04)	-0·00 (-0·03 to 0·02)
Ecuador	1·50 (0·83 to 2·41)	16·82 (8·79 to 28·61)	0·65 (0·51 to 0·90)	31·86 (27·62 to 36·55)	0·02 (-0·01 to 0·06)	0·04 (0·01 to 0·06)	0·02 (-0·01 to 0·04)
Peru	1·20 (0·71 to 1·85)	16·88 (8·72 to 29·99)	0·70 (0·54 to 0·95)	35·51 (29·78 to 42·37)	0·00 (-0·03 to 0·03)	0·01 (-0·01 to 0·02)	-0·03 (-0·05 to -0·01)
Caribbean	9·08 (6·99 to 11·70)	152·93 (133·64 to 174·67)	6·66 (5·83 to 7·61)	43·72 (39·69 to 47·93)	-0·02 (-0·04 to 0·01)	-0·01 (-0·02 to -0·00)	-0·06 (-0·07 to -0·05)
Antigua and Barbuda	0·01 (0·00 to 0·03)	0·22 (0·09 to 0·45)	0·01 (0·01 to 0·01)	38·73 (28·34 to 49·37)	0·01 (-0·07 to 0·15)	0·00 (-0·02 to 0·05)	-0·03 (-0·04 to -0·02)
The Bahamas	0·07 (0·04 to 0·11)	2·10 (1·11 to 3·37)	0·08 (0·05 to 0·13)	51·62 (41·97 to 62·20)	-0·07 (-0·10 to -0·03)	-0·01 (-0·03 to 0·00)	-0·05 (-0·07 to -0·03)
Barbados	0·04 (0·02 to 0·07)	0·70 (0·31 to 1·22)	0·02 (0·02 to 0·02)	45·68 (36·52 to 56·37)	0·02 (-0·02 to 0·05)	-0·00 (-0·01 to 0·01)	-0·04 (-0·05 to -0·04)
Belize	0·12 (0·07 to 0·18)	1·80 (0·90 to 3·12)	0·07 (0·04 to 0·13)	56·11 (49·64 to 63·33)	0·02 (-0·01 to 0·05)	-0·00 (-0·02 to 0·01)	0·01 (-0·03 to 0·05)
Bermuda	0·01 (0·01 to 0·03)	0·24 (0·10 to 0·53)	0·01 (0·01 to 0·01)	39·60 (28·97 to 50·46)	0·01 (-0·08 to 0·15)	-0·01 (-0·03 to 0·04)	-0·02 (-0·03 to -0·01)
Cuba	0·87 (0·53 to 1·35)	14·07 (7·10 to 22·70)	0·25 (0·24 to 0·27)	65·87 (58·91 to 73·38)	-0·00 (-0·03 to 0·02)	0·08 (0·06 to 0·10)	0·08 (0·07 to 0·09)
Dominica	0·01 (0·00 to 0·02)	0·12 (0·05 to 0·25)	0·00 (0·00 to 0·01)	36·23 (26·89 to 45·48)	0·01 (-0·08 to 0·17)	0·01 (-0·02 to 0·06)	-0·01 (-0·04 to 0·04)
Dominican Republic	1·72 (1·22 to 2·41)	30·95 (26·01 to 36·15)	1·53 (0·92 to 1·97)	32·84 (27·66 to 38·80)	0·02 (-0·05 to 0·13)	-0·05 (-0·06 to -0·04)	-0·09 (-0·14 to -0·06)
Grenada	0·01 (0·01 to 0·03)	0·20 (0·08 to 0·42)	0·01 (0·00 to 0·01)	32·45 (24·07 to 40·99)	0·00 (-0·08 to 0·13)	0·01 (-0·02 to 0·05)	-0·01 (-0·04 to 0·05)
Guyana	0·69 (0·39 to 1·07)	11·27 (5·14 to 19·70)	0·34 (0·21 to 0·53)	61·14 (51·14 to 69·87)	-0·01 (-0·04 to 0·01)	0·01 (-0·01 to 0·05)	0·02 (-0·01 to 0·06)
Haiti	4·10 (2·25 to 6·38)	62·89 (51·50 to 75·74)	3·34 (2·71 to 4·05)	40·43 (34·42 to 46·94)	-0·04 (-0·08 to 0·01)	-0·02 (-0·04 to -0·01)	-0·07 (-0·08 to -0·06)
Jamaica	0·44 (0·27 to 0·66)	6·86 (3·30 to 11·15)	0·31 (0·21 to 0·50)	37·94 (31·16 to 45·95)	-0·00 (-0·03 to 0·02)	-0·01 (-0·02 to 0·00)	-0·04 (-0·06 to -0·01)
Puerto Rico	0·31 (0·12 to 0·77)	7·87 (2·91 to 18·47)	0·20 (0·19 to 0·22)	48·45 (34·31 to 62·65)	-0·01 (-0·10 to 0·15)	-0·04 (-0·06 to 0·01)	-0·07 (-0·08 to -0·06)
Saint Lucia	0·01 (0·01 to 0·03)	0·24 (0·10 to 0·48)	0·01 (0·01 to 0·01)	36·82 (27·19 to 46·05)	0·01 (-0·08 to 0·15)	0·00 (-0·02 to 0·05)	-0·04 (-0·05 to -0·03)
Saint Vincent and the Grenadines	0·03 (0·01 to 0·07)	0·51 (0·21 to 1·04)	0·01 (0·01 to 0·01)	35·83 (27·00 to 45·33)	0·01 (-0·08 to 0·16)	0·01 (-0·02 to 0·05)	-0·03 (-0·04 to -0·02)
Suriname	0·12 (0·07 to 0·21)	2·33 (1·07 to 4·19)	0·10 (0·06 to 0·15)	43·60 (35·07 to 51·38)	-0·01 (-0·05 to 0·02)	-0·01 (-0·02 to 0·00)	0·01 (-0·03 to 0·03)
Trinidad and Tobago	0·20 (0·12 to 0·32)	4·89 (2·18 to 9·34)	0·13 (0·13 to 0·15)	34·45 (27·26 to 41·55)	-0·05 (-0·07 to -0·03)	-0·02 (-0·03 to -0·01)	-0·04 (-0·05 to -0·04)
Virgin Islands, U·S·	0·01 (0·00 to 0·02)	0·18 (0·07 to 0·38)	0·01 (0·00 to 0·01)	42·40 (30·69 to 52·76)	0·01 (-0·08 to 0·16)	0·00 (-0·02 to 0·05)	-0·01 (-0·05 to 0·04)
Tropical Latin America	24·05 (21·32 to 26·74)	376·13 (307·68 to 460·87)	15·95 (13·43 to 19·12)	48·88 (46·97 to 51·02)	-0·01 (-0·01 to -0·00)	0·01 (-0·00 to 0·01)	0·02 (0·01 to 0·03)
Brazil	23·20 (20·68 to 25·90)	367·60 (299·54 to 451·94)	15·67 (13·12 to 18·89)	49·39 (47·50 to 51·46)	-0·01 (-0·01 to -0·00)	0·00 (-0·00 to 0·01)	0·02 (0·01 to 0·03)
Paraguay	0·85 (0·33 to 1·69)	8·53 (3·96 to 15·48)	0·28 (0·20 to 0·37)	27·24 (21·48 to 34·46)	0·02 (-0·04 to 0·06)	0·06 (0·03 to 0·08)	0·05 (0·02 to 0·08)

Appendix Table 2. Country-specific estimates of new HIV infections, counts of PLWH, counts of HIV/AIDS deaths, ART coverage per person LWH in 2015, and ARCs of age-standardized incidence, prevalence, and mortality rates from 2005 to 2015 among men and boys

Location	New infections in thousands (95% UI)	PLWH in thousands (95% UI)	HIV/AIDS deaths in thousands (95% UI)	ART coverage per 100 PLWH (95% UI)	Age-standardized incidence rate ARC from 2005 to 2015 (95% UI)	Age-standardized prevalence rate ARC from 2005 to 2015 (95% UI)	Age-standardized mortality rate ARC from 2005 to 2015 (95% UI)
Southeast Asia, East Asia, and Oceania	124·35 (87·39 to 188·14)	1 to48·48 (1 to07·01 to	76·93 (57·85 to 122·76)	25·06 (20·49 to 30·98)	-0·00 (-0·03 to 0·03)	0·03 (-0·00 to 0·05)	0·03 (-0·02 to 0·06)
East Asia	39·97 (28·98 to 54·83)	549·14 (407·74 to 720·05)	30·35 (27·02 to 33·77)	17·80 (15·71 to 20·19)	-0·01 (-0·04 to 0·00)	0·04 (0·02 to 0·05)	0·08 (0·07 to 0·09)
China	39·01 (27·92 to 53·64)	536·99 (398·05 to 705·44)	29·67 (26·35 to 33·00)	17·80 (15·68 to 20·19)	-0·01 (-0·04 to 0·00)	0·04 (0·02 to 0·05)	0·08 (0·07 to 0·09)
North Korea	0·62 (0·06 to 2·15)	7·47 (1·53 to 26·43)	0·43 (0·08 to 1·67)	16·92 (6·73 to 32·60)	0·00 (-0·14 to 0·11)	0·05 (-0·02 to 0·13)	0·08 (-0·01 to 0·17)
Taiwan	0·34 (0·10 to 0·71)	4·68 (1·87 to 9·52)	0·25 (0·18 to 0·33)	17·06 (11·68 to 23·59)	-0·02 (-0·11 to 0·03)	0·04 (0·01 to 0·07)	0·09 (0·06 to 0·11)
Southeast Asia	83·28 (47·87 to 142·75)	1080·44 (663·85 to 1893·78)	45·79 (27·29 to 91·54)	28·44 (21·20 to 36·20)	-0·00 (-0·04 to 0·04)	0·02 (-0·02 to 0·05)	0·01 (-0·05 to 0·05)
Cambodia	5·46 (2·46 to 10·06)	56·86 (22·29 to 104·67)	1·90 (1·27 to 2·73)	29·86 (22·42 to 37·06)	0·04 (-0·00 to 0·06)	0·01 (-0·01 to 0·02)	0·01 (-0·02 to 0·03)
Indonesia	28·69 (5·75 to 84·38)	290·99 (61·92 to 896·28)	13·11 (2·63 to 48·42)	11·82 (8·11 to 16·36)	0·01 (-0·04 to 0·09)	0·10 (0·05 to 0·15)	0·17 (0·10 to 0·23)
Laos	0·36 (0·08 to 1·07)	4·71 (1·16 to 15·72)	0·14 (0·03 to 0·52)	31·91 (23·04 to 41·22)	-0·04 (-0·10 to 0·05)	0·06 (0·02 to 0·11)	0·09 (0·03 to 0·16)
Malaysia	1·77 (1·35 to 2·48)	33·00 (18·42 to 59·41)	2·09 (1·65 to 3·07)	25·06 (18·51 to 33·27)	-0·06 (-0·08 to -0·05)	-0·03 (-0·04 to -0·02)	-0·01 (-0·03 to 0·01)
Maldives	0·00 (0·00 to 0·00)	0·00 (0·00 to 0·01)	0·00 (0·00 to 0·00)	21·77 (15·69 to 29·55)	-0·01 (-0·04 to 0·01)	-0·02 (-0·03 to 0·00)	-0·05 (-0·07 to -0·04)
Mauritius	0·09 (0·06 to 0·15)	1·24 (0·73 to 2·00)	0·07 (0·06 to 0·08)	18·64 (14·56 to 22·81)	-0·00 (-0·03 to 0·02)	0·04 (0·03 to 0·05)	0·13 (0·11 to 0·14)
Myanmar	5·64 (1·14 to 15·84)	141·81 (31·90 to 521·58)	7·75 (1·53 to 33·41)	33·82 (25·81 to 44·08)	-0·08 (-0·14 to 0·01)	-0·02 (-0·08 to 0·04)	-0·04 (-0·12 to 0·02)
Philippines	23·68 (8·65 to 60·12)	193·57 (90·80 to 344·51)	2·47 (2·23 to 2·72)	34·76 (25·63 to 45·49)	0·09 (0·03 to 0·19)	0·09 (0·06 to 0·12)	-0·06 (-0·08 to -0·05)
Sri Lanka	0·14 (0·06 to 0·24)	1·34 (0·59 to 2·49)	0·03 (0·03 to 0·04)	22·51 (19·57 to 25·91)	0·07 (0·03 to 0·11)	0·03 (0·00 to 0·04)	-0·03 (-0·05 to -0·02)
Seychelles	0·01 (0·00 to 0·02)	0·08 (0·02 to 0·17)	0·00 (0·00 to 0·01)	27·27 (14·93 to 40·82)	-0·02 (-0·41 to 0·15)	0·02 (-0·04 to 0·10)	0·01 (-0·03 to 0·05)
Thailand	7·08 (2·04 to 15·25)	188·87 (86·42 to 342·73)	11·65 (7·82 to 16·88)	35·43 (28·18 to 45·98)	-0·07 (-0·20 to 0·01)	-0·02 (-0·04 to -0·00)	-0·00 (-0·03 to 0·02)
Timor-Leste	0·06 (0·00 to 0·26)	0·85 (0·02 to 4·08)	0·04 (0·00 to 0·24)	23·40 (8·54 to 47·42)	0·01 (-0·41 to 0·27)	0·04 (-0·08 to 0·20)	0·04 (-0·09 to 0·18)
Vietnam	10·21 (2·16 to 29·60)	165·73 (32·56 to 615·05)	6·47 (1·29 to 26·46)	31·94 (22·40 to 43·04)	-0·03 (-0·09 to 0·05)	0·03 (-0·01 to 0·08)	0·03 (-0·03 to 0·09)
Oceania	1·10 (0·74 to 1·58)	18·90 (16·08 to 21·89)	0·79 (0·63 to 0·97)	50·90 (43·67 to 58·45)	-0·05 (-0·09 to -0·01)	0·01 (-0·01 to 0·03)	-0·03 (-0·05 to -0·01)
American Samoa	0·00 (0·00 to 0·00)	0·01 (0·00 to 0·02)	0·00 (0·00 to 0·00)	28·08 (22·09 to 36·50)	0·04 (-0·06 to 0·09)	0·04 (0·00 to 0·08)	0·00 (-0·02 to 0·03)
Federated States of Micronesia	0·00 (0·00 to 0·01)	0·04 (0·01 to 0·18)	0·00 (0·00 to 0·01)	25·05 (14·85 to 45·02)	0·07 (-0·10 to 0·19)	0·06 (-0·04 to 0·18)	0·01 (-0·08 to 0·12)
Fiji	0·03 (0·02 to 0·06)	0·30 (0·13 to 0·54)	0·01 (0·01 to 0·02)	25·84 (22·14 to 29·97)	0·05 (0·03 to 0·07)	0·04 (0·03 to 0·06)	0·05 (0·02 to 0·07)
Guam	0·01 (0·00 to 0·02)	0·10 (0·04 to 0·20)	0·00 (0·00 to 0·01)	30·14 (22·64 to 40·72)	0·04 (-0·05 to 0·09)	0·04 (0·00 to 0·08)	-0·00 (-0·03 to 0·03)
Kiribati	0·00 (0·00 to 0·00)	0·01 (0·00 to 0·02)	0·00 (0·00 to 0·00)	28·83 (21·42 to 39·08)	0·03 (-0·06 to 0·08)	0·03 (-0·01 to 0·06)	-0·02 (-0·05 to 0·00)
Marshall Islands	0·00 (0·00 to 0·01)	0·04 (0·00 to 0·16)	0·00 (0·00 to 0·01)	26·32 (15·61 to 47·64)	0·07 (-0·10 to 0·19)	0·07 (-0·05 to 0·19)	0·01 (-0·09 to 0·14)
Northern Mariana Islands	0·00 (0·00 to 0·01)	0·03 (0·01 to 0·07)	0·00 (0·00 to 0·00)	25·39 (19·92 to 34·06)	0·04 (-0·06 to 0·08)	0·05 (0·01 to 0·08)	0·02 (-0·01 to 0·04)
Papua New Guinea	0·93 (0·59 to 1·38)	16·58 (14·15 to 19·02)	0·69 (0·55 to 0·84)	51·90 (44·14 to 59·90)	-0·06 (-0·10 to -0·02)	0·01 (-0·01 to 0·03)	-0·04 (-0·06 to -0·01)
Samoa	0·01 (0·00 to 0·02)	0·09 (0·01 to 0·40)	0·00 (0·00 to 0·02)	27·47 (16·54 to 47·19)	0·07 (-0·10 to 0·20)	0·07 (-0·05 to 0·18)	0·02 (-0·08 to 0·13)
Solomon Islands	0·02 (0·00 to 0·07)	0·26 (0·03 to 1·13)	0·01 (0·00 to 0·07)	26·05 (15·97 to 44·81)	0·07 (-0·10 to 0·20)	0·06 (-0·05 to 0·18)	0·01 (-0·08 to 0·13)
Tonga	0·00 (0·00 to 0·01)	0·02 (0·01 to 0·04)	0·00 (0·00 to 0·00)	23·32 (18·54 to 31·69)	0·08 (-0·02 to 0·14)	0·10 (0·04 to 0·14)	0·05 (0·02 to 0·09)
Vanuatu	0·01 (0·00 to 0·03)	0·13 (0·02 to 0·52)	0·01 (0·00 to 0·03)	26·30 (16·08 to 46·09)	0·07 (-0·09 to 0·20)	0·07 (-0·05 to 0·18)	0·01 (-0·08 to 0·13)
North Africa and Middle East	8·54 (5·78 to 12·12)	92·91 (74·63 to 120·17)	5·48 (4·46 to 6·87)	16·87 (14·02 to 20·35)	-0·02 (-0·06 to 0·01)	0·01 (-0·01 to 0·03)	0·01 (-0·02 to 0·03)
North Africa and Middle East	8·54 (5·78 to 12·12)	92·91 (74·63 to 120·17)	5·48 (4·46 to 6·87)	16·87 (14·02 to 20·35)	-0·02 (-0·06 to 0·01)	0·01 (-0·01 to 0·03)	0·01 (-0·02 to 0·03)
Afghanistan	0·50 (0·10 to 1·47)	3·20 (0·68 to 9·68)	0·15 (0·03 to 0·52)	4·16 (3·05 to 5·83)	0·09 (0·01 to 0·17)	0·05 (-0·05 to 0·14)	0·02 (-0·11 to 0·12)
Algeria	0·16 (0·00 to 0·54)	3·32 (1·08 to 6·05)	0·14 (0·07 to 0·24)	57·30 (49·90 to 64·36)	-0·13 (-0·49 to 0·04)	0·03 (-0·03 to 0·06)	0·02 (-0·03 to 0·06)
Bahrain	0·05 (0·01 to 0·11)	0·45 (0·19 to 0·89)	0·02 (0·01 to 0·03)	17·11 (13·04 to 22·48)	0·04 (-0·08 to 0·11)	0·03 (-0·01 to 0·07)	-0·01 (-0·04 to 0·02)
Egypt	0·53 (0·27 to 0·93)	3·73 (1·84 to 6·42)	0·12 (0·10 to 0·14)	18·57 (15·36 to 22·24)	0·09 (0·06 to 0·13)	0·09 (0·07 to 0·11)	0·03 (0·01 to 0·05)
Iran	1·01 (0·56 to 1·83)	10·09 (5·09 to 18·62)	0·51 (0·40 to 0·70)	12·32 (10·86 to 14·05)	0·03 (-0·02 to 0·08)	0·03 (0·01 to 0·05)	0·05 (0·01 to 0·08)

Appendix Table 2. Country-specific estimates of new HIV infections, counts of PLWH, counts of HIV/AIDS deaths, ART coverage per person LWH in 2015, and ARCs of age-standardized incidence, prevalence, and mortality rates from 2005 to 2015 among men and boys

Location	New infections in thousands (95% UI)	PLWH in thousands (95% UI)	HIV/AIDS deaths in thousands (95% UI)	ART coverage per 100 PLWH (95% UI)	Age-standardized incidence rate ARC from 2005 to 2015 (95% UI)	Age-standardized prevalence rate ARC from 2005 to 2015 (95% UI)	Age-standardized mortality rate ARC from 2005 to 2015 (95% UI)
Iraq	0·31 (0·08 to 0·64)	2·16 (1·00 to 4·10)	0·07 (0·05 to 0·10)	15·18 (11·89 to 19·77)	0·07 (-0·07 to 0·14)	0·08 (0·03 to 0·13)	0·06 (0·03 to 0·10)
Jordan	0·01 (0·00 to 0·03)	0·13 (0·06 to 0·27)	0·01 (0·00 to 0·01)	18·59 (14·41 to 24·02)	0·02 (-0·12 to 0·08)	0·02 (-0·02 to 0·05)	-0·00 (-0·03 to 0·04)
Kuwait	0·01 (0·00 to 0·02)	0·08 (0·03 to 0·16)	0·00 (0·00 to 0·00)	17·89 (13·87 to 23·57)	0·03 (-0·11 to 0·09)	0·00 (-0·04 to 0·04)	-0·12 (-0·13 to -0·10)
Lebanon	0·09 (0·02 to 0·27)	1·41 (0·30 to 5·77)	0·06 (0·01 to 0·32)	40·62 (27·18 to 66·34)	0·03 (-0·03 to 0·10)	0·01 (-0·06 to 0·08)	-0·00 (-0·07 to 0·05)
Libya	0·12 (0·01 to 0·51)	1·27 (0·08 to 5·27)	0·06 (0·00 to 0·33)	18·27 (12·58 to 25·97)	0·04 (-0·14 to 0·16)	0·05 (-0·06 to 0·16)	0·02 (-0·09 to 0·13)
Morocco	0·36 (0·21 to 0·59)	4·47 (2·15 to 7·84)	0·20 (0·15 to 0·27)	25·06 (22·70 to 27·45)	-0·01 (-0·03 to 0·02)	0·03 (0·02 to 0·04)	0·08 (0·04 to 0·10)
Palestine	0·04 (0·01 to 0·08)	0·28 (0·13 to 0·56)	0·01 (0·01 to 0·02)	16·27 (12·59 to 21·07)	0·03 (-0·08 to 0·10)	0·05 (0·02 to 0·09)	0·04 (0·01 to 0·08)
Oman	0·11 (0·07 to 0·16)	1·42 (0·72 to 2·36)	0·06 (0·05 to 0·08)	29·96 (24·03 to 36·65)	-0·02 (-0·06 to 0·01)	-0·00 (-0·02 to 0·01)	0·04 (-0·01 to 0·07)
Qatar	0·01 (0·00 to 0·02)	0·12 (0·05 to 0·22)	0·01 (0·00 to 0·01)	16·32 (12·35 to 21·53)	0·01 (-0·11 to 0·08)	-0·03 (-0·06 to -0·00)	-0·07 (-0·09 to -0·03)
Saudi Arabia	0·69 (0·32 to 1·37)	7·53 (3·72 to 16·73)	0·35 (0·18 to 0·96)	23·03 (19·12 to 28·07)	0·02 (-0·05 to 0·08)	0·03 (-0·04 to 0·07)	0·01 (-0·07 to 0·05)
Sudan	3·00 (0·83 to 6·07)	37·70 (27·91 to 48·98)	3·12 (2·23 to 3·86)	7·91 (5·73 to 10·63)	-0·09 (-0·20 to -0·02)	-0·02 (-0·05 to 0·01)	0·01 (-0·02 to 0·04)
Syria	0·02 (0·01 to 0·04)	0·28 (0·07 to 0·60)	0·01 (0·01 to 0·02)	17·41 (14·17 to 21·10)	0·10 (-0·01 to 0·14)	0·10 (0·01 to 0·14)	0·06 (-0·01 to 0·10)
Tunisia	0·20 (0·09 to 0·38)	1·81 (0·79 to 3·28)	0·07 (0·05 to 0·10)	21·12 (18·14 to 24·78)	0·05 (0·00 to 0·09)	0·07 (0·05 to 0·09)	0·10 (0·07 to 0·13)
Turkey	0·49 (0·19 to 0·87)	5·36 (2·39 to 9·22)	0·13 (0·10 to 0·18)	32·84 (26·78 to 40·50)	0·01 (-0·06 to 0·04)	0·07 (0·04 to 0·10)	0·03 (-0·01 to 0·06)
United Arab Emirates	0·46 (0·02 to 1·79)	4·91 (0·29 to 21·65)	0·24 (0·01 to 1·23)	16·67 (11·78 to 23·35)	0·04 (-0·15 to 0·16)	0·05 (-0·06 to 0·16)	0·02 (-0·09 to 0·13)
Yemen	0·36 (0·07 to 1·06)	3·08 (0·70 to 9·93)	0·12 (0·02 to 0·44)	31·24 (23·93 to 40·47)	0·04 (-0·04 to 0·12)	0·04 (-0·06 to 0·12)	-0·02 (-0·14 to 0·09)
South Asia	89·99 (74·48 to 109·24)	1303·58 (1212·89 to 1408·08)	54·93 (50·97 to 60·51)	36·87 (34·31 to 39·27)	-0·01 (-0·03 to 0·01)	-0·02 (-0·02 to -0·01)	-0·08 (-0·08 to -0·07)
South Asia	89·99 (74·48 to 109·24)	1303·58 (1212·89 to 1408·08)	54·93 (50·97 to 60·51)	36·87 (34·31 to 39·27)	-0·01 (-0·03 to 0·01)	-0·02 (-0·02 to -0·01)	-0·08 (-0·08 to -0·07)
Bangladesh	0·32 (0·07 to 0·93)	4·13 (0·86 to 14·13)	0·18 (0·03 to 0·74)	15·49 (12·03 to 19·34)	-0·02 (-0·08 to 0·06)	0·08 (0·04 to 0·14)	0·13 (0·07 to 0·19)
Bhutan	0·03 (0·01 to 0·09)	0·30 (0·06 to 1·02)	0·01 (0·00 to 0·03)	28·96 (19·23 to 46·09)	0·02 (-0·05 to 0·09)	0·03 (-0·06 to 0·11)	-0·04 (-0·16 to 0·07)
India	82·74 (69·11 to 100·19)	1244·32 (1173·59 to 1323·57)	52·16 (48·89 to 55·38)	37·86 (35·52 to 40·07)	-0·01 (-0·03 to 0·01)	-0·02 (-0·02 to -0·01)	-0·08 (-0·09 to -0·07)
Nepal	0·76 (0·16 to 2·28)	22·18 (4·87 to 82·39)	1·51 (0·30 to 6·36)	29·72 (23·29 to 37·96)	-0·14 (-0·20 to -0·06)	-0·02 (-0·05 to 0·02)	0·03 (-0·03 to 0·09)
Pakistan	6·14 (1·18 to 18·53)	32·65 (6·87 to 101·86)	1·08 (0·19 to 3·58)	5·90 (4·05 to 8·25)	0·15 (0·07 to 0·24)	0·15 (0·06 to 0·24)	0·14 (0·00 to 0·24)
Sub-Saharan Africa	828·73 (744·65 to 924·91)	12391·81 (11962·00 to 12820·88)	435·76 (403·60 to 465·11)	38·62 (37·20 to 40·02)	-0·03 (-0·04 to -0·02)	-0·01 (-0·01 to -0·01)	-0·08 (-0·09 to -0·07)
Southern Sub-Saharan Africa	323·85 (274·47 to 380·35)	4789·53 (4527·76 to 5048·97)	113·65 (105·38 to 123·76)	48·84 (46·27 to 51·46)	-0·03 (-0·04 to -0·01)	0·01 (0·00 to 0·01)	-0·08 (-0·09 to -0·07)
Botswana	10·89 (6·61 to 16·12)	191·16 (166·72 to 220·05)	4·51 (2·71 to 6·15)	58·25 (48·98 to 68·39)	-0·01 (-0·06 to 0·04)	0·00 (-0·01 to 0·01)	-0·07 (-0·10 to -0·03)
Lesotho	11·32 (7·94 to 15·70)	153·30 (134·54 to 177·00)	6·28 (4·93 to 8·20)	31·80 (27·97 to 35·94)	-0·02 (-0·05 to 0·01)	0·01 (0·00 to 0·02)	-0·03 (-0·04 to -0·01)
Namibia	6·16 (4·34 to 8·25)	103·90 (93·33 to 115·32)	2·79 (2·00 to 3·62)	47·19 (41·51 to 53·32)	-0·03 (-0·06 to 0·01)	0·01 (0·00 to 0·01)	-0·07 (-0·09 to -0·05)
South Africa	243·22 (199·99 to 290·15)	3 to506·82 (3 to252·73 to 3 to752·75)	76·37 (69·30 to 84·58)	48·40 (45·54 to 51·56)	-0·03 (-0·05 to -0·01)	0·01 (0·01 to 0·02)	-0·07 (-0·08 to -0·06)
Swaziland	6·47 (4·16 to 8·68)	113·78 (100·81 to 126·53)	2·99 (2·35 to 3·93)	49·22 (43·13 to 55·42)	-0·06 (-0·10 to -0·03)	0·01 (0·00 to 0·02)	-0·07 (-0·08 to -0·04)
Zimbabwe	45·79 (22·63 to 77·50)	720·57 (630·63 to 829·28)	20·70 (16·81 to 25·23)	52·36 (45·38 to 59·63)	-0·01 (-0·08 to 0·06)	-0·02 (-0·03 to -0·01)	-0·13 (-0·15 to -0·09)
Western Sub-Saharan Africa	198·59 (146·73 to 259·28)	2716·66 (2479·69 to 2972·77)	127·20 (101·80 to 150·52)	23·16 (21·01 to 25·68)	-0·03 (-0·06 to -0·00)	-0·01 (-0·02 to -0·00)	-0·05 (-0·07 to -0·03)
Benin	2·36 (1·51 to 3·54)	35·24 (30·16 to 41·58)	1·09 (0·66 to 1·51)	44·88 (38·97 to 50·92)	-0·02 (-0·07 to 0·03)	-0·01 (-0·02 to 0·00)	-0·11 (-0·16 to -0·08)
Burkina Faso	2·63 (1·40 to 4·24)	41·64 (33·97 to 51·23)	1·70 (1·05 to 2·13)	50·50 (42·81 to 59·16)	-0·02 (-0·08 to 0·05)	-0·05 (-0·07 to -0·04)	-0·15 (-0·20 to -0·13)
Cameroon	21·36 (12·71 to 32·41)	276·03 (228·30 to 328·55)	16·49 (11·61 to 21·43)	17·24 (14·57 to 20·33)	-0·03 (-0·08 to 0·01)	-0·01 (-0·03 to 0·00)	-0·02 (-0·04 to -0·01)
Cape Verde	0·23 (0·10 to 0·60)	2·54 (1·87 to 3·48)	0·08 (0·06 to 0·12)	22·64 (15·85 to 30·61)	-0·01 (-0·07 to 0·13)	0·02 (-0·01 to 0·06)	-0·03 (-0·06 to -0·01)
Chad	4·14 (1·67 to 7·58)	70·12 (52·00 to 90·97)	4·00 (2·26 to 5·47)	31·98 (23·45 to 42·61)	-0·09 (-0·19 to -0·02)	-0·03 (-0·05 to -0·01)	-0·06 (-0·11 to -0·03)
Cote d'Ivoire	18·89 (10·75 to 29·43)	234·34 (191·91 to 282·88)	12·35 (9·86 to 15·53)	22·29 (17·80 to 28·10)	-0·02 (-0·06 to 0·03)	-0·02 (-0·04 to -0·01)	-0·07 (-0·09 to -0·05)

Appendix Table 2. Country-specific estimates of new HIV infections, counts of PLWH, counts of HIV/AIDS deaths, ART coverage per person LWH in 2015, and ARCs of age-standardized incidence, prevalence, and mortality rates from 2005 to 2015 among men and boys

Location	New infections in thousands (95% UI)	PLWH in thousands (95% UI)	HIV/AIDS deaths in thousands (95% UI)	ART coverage per 100 PLWH (95% UI)	Age-standardized incidence rate ARC from 2005 to 2015 (95% UI)	Age-standardized prevalence rate ARC from 2005 to 2015 (95% UI)	Age-standardized mortality rate ARC from 2005 to 2015 (95% UI)
The Gambia	0·39 (0·15 to 0·72)	7·03 (5·43 to 9·04)	0·34 (0·22 to 0·48)	17·05 (13·59 to 21·61)	-0·10 (-0·21 to -0·03)	0·00 (-0·02 to 0·02)	0·00 (-0·03 to 0·02)
Ghana	7·46 (4·23 to 11·49)	114·36 (92·38 to 140·79)	5·58 (3·40 to 7·35)	33·37 (27·01 to 41·01)	-0·04 (-0·10 to 0·01)	-0·04 (-0·05 to -0·03)	-0·09 (-0·14 to -0·06)
Guinea	3·80 (1·63 to 6·14)	55·91 (44·43 to 68·54)	2·61 (2·02 to 3·32)	23·25 (19·30 to 27·61)	-0·05 (-0·12 to 0·00)	-0·00 (-0·03 to 0·02)	-0·03 (-0·05 to -0·01)
Guinea-Bissau	0·84 (0·31 to 1·61)	17·07 (14·00 to 20·61)	0·87 (0·49 to 1·21)	18·25 (15·82 to 20·97)	-0·11 (-0·21 to -0·03)	0·02 (-0·00 to 0·04)	0·03 (-0·01 to 0·09)
Liberia	1·17 (0·54 to 2·08)	15·55 (12·82 to 19·17)	1·03 (0·70 to 1·25)	19·15 (15·08 to 23·31)	-0·02 (-0·10 to 0·07)	-0·05 (-0·07 to -0·03)	-0·07 (-0·11 to -0·05)
Mali	5·13 (2·73 to 7·99)	62·10 (46·31 to 80·41)	3·47 (2·08 to 4·69)	17·92 (15·06 to 21·38)	-0·03 (-0·09 to 0·00)	-0·01 (-0·04 to 0·01)	-0·03 (-0·08 to -0·01)
Mauritania	0·05 (0·01 to 0·19)	3·14 (0·61 to 14·03)	0·13 (0·02 to 0·59)	48·97 (32·19 to 67·04)	-0·19 (-0·33 to -0·08)	-0·04 (-0·07 to -0·00)	-0·08 (-0·15 to -0·02)
Niger	0·88 (0·33 to 1·71)	24·41 (20·37 to 29·48)	1·54 (1·27 to 1·85)	34·99 (29·35 to 41·09)	-0·12 (-0·23 to -0·04)	-0·06 (-0·08 to -0·05)	-0·09 (-0·12 to -0·07)
Nigeria	123·34 (73·68 to 182·51)	1657·57 (1439·12 to 1912·32)	70·34 (45·62 to 92·87)	22·04 (18·75 to 25·87)	-0·02 (-0·07 to 0·01)	0·00 (-0·01 to 0·02)	-0·04 (-0·08 to -0·01)
Sao Tome and Principe	0·00 (0·00 to 0·00)	0·02 (0·01 to 0·03)	0·00 (0·00 to 0·00)	54·48 (47·58 to 62·64)	-0·01 (-0·07 to 0·04)	0·03 (0·01 to 0·05)	-0·06 (-0·10 to -0·02)
Senegal	1·88 (0·59 to 3·22)	25·71 (19·71 to 32·98)	1·05 (0·63 to 1·36)	38·84 (32·37 to 46·72)	-0·05 (-0·16 to -0·00)	-0·02 (-0·04 to 0·00)	-0·06 (-0·11 to -0·03)
Sierra Leone	1·77 (0·54 to 3·05)	24·89 (19·97 to 30·47)	1·37 (0·98 to 1·82)	13·90 (10·53 to 18·25)	-0·06 (-0·18 to 0·00)	-0·00 (-0·02 to 0·02)	0·01 (-0·02 to 0·03)
Togo	2·27 (1·14 to 4·16)	48·96 (41·55 to 58·17)	3·15 (2·49 to 3·84)	24·12 (19·31 to 29·05)	-0·09 (-0·17 to -0·03)	-0·04 (-0·06 to -0·03)	-0·05 (-0·06 to -0·03)
Eastern Sub-Saharan Africa	272·79 (232·76 to 314·31)	4378·48 (4163·45 to 4610·10)	165·72 (150·25 to 180·17)	38·53 (36·32 to 40·76)	-0·04 (-0·05 to -0·02)	-0·02 (-0·02 to -0·01)	-0·10 (-0·11 to -0·09)
Burundi	2·83 (1·41 to 4·88)	46·69 (38·52 to 56·60)	2·10 (1·59 to 2·55)	34·42 (28·39 to 41·23)	-0·02 (-0·09 to 0·05)	-0·04 (-0·06 to -0·03)	-0·11 (-0·13 to -0·09)
Comoros	0·04 (0·01 to 0·13)	0·27 (0·06 to 0·88)	0·02 (0·00 to 0·06)	8·71 (4·88 to 14·57)	0·02 (-0·09 to 0·13)	0·00 (-0·07 to 0·07)	0·04 (-0·05 to 0·12)
Djibouti	0·23 (0·08 to 0·44)	3·23 (2·18 to 4·75)	0·18 (0·10 to 0·25)	24·27 (19·52 to 30·10)	-0·03 (-0·10 to 0·05)	-0·03 (-0·06 to -0·01)	-0·07 (-0·13 to -0·02)
Eritrea	0·65 (0·30 to 1·11)	9·00 (6·83 to 12·23)	0·36 (0·22 to 0·54)	36·87 (26·10 to 50·41)	-0·01 (-0·08 to 0·06)	-0·03 (-0·05 to -0·00)	-0·10 (-0·14 to -0·05)
Ethiopia	16·89 (8·09 to 27·77)	323·58 (267·13 to 388·98)	13·87 (8·26 to 17·31)	51·75 (44·69 to 59·42)	0·01 (-0·10 to 0·11)	-0·06 (-0·07 to -0·05)	-0·16 (-0·22 to -0·12)
Kenya	60·41 (49·70 to 72·82)	787·62 (747·05 to 836·18)	29·13 (26·92 to 31·44)	32·76 (30·49 to 34·72)	0·06 (0·03 to 0·08)	-0·03 (-0·03 to -0·02)	-0·11 (-0·12 to -0·10)
Madagascar	0·90 (0·15 to 2·89)	18·66 (3·82 to 79·55)	2·05 (0·35 to 9·43)	1·40 (0·77 to 3·00)	-0·13 (-0·21 to -0·03)	-0·06 (-0·15 to 0·00)	-0·04 (-0·13 to 0·03)
Malawi	25·09 (10·90 to 36·49)	479·21 (410·50 to 544·02)	15·83 (12·55 to 20·09)	42·80 (35·74 to 51·18)	-0·07 (-0·15 to -0·02)	-0·02 (-0·04 to -0·01)	-0·12 (-0·14 to -0·09)
Mozambique	54·16 (32·97 to 77·68)	733·43 (626·80 to 840·00)	34·87 (28·58 to 41·88)	25·75 (21·85 to 30·24)	-0·05 (-0·10 to -0·01)	0·01 (-0·01 to 0·02)	-0·01 (-0·03 to 0·01)
Rwanda	3·32 (1·81 to 5·11)	78·21 (68·05 to 89·38)	2·25 (1·28 to 3·05)	53·63 (47·19 to 60·54)	-0·08 (-0·14 to -0·03)	-0·02 (-0·03 to -0·01)	-0·14 (-0·20 to -0·11)
Somalia	0·76 (0·35 to 1·38)	10·43 (7·33 to 14·97)	0·74 (0·48 to 0·97)	7·78 (4·85 to 12·19)	-0·08 (-0·18 to -0·01)	-0·03 (-0·07 to 0·00)	-0·02 (-0·05 to 0·02)
South Sudan	4·35 (1·66 to 7·72)	52·22 (33·40 to 75·65)	4·19 (1·79 to 5·89)	6·14 (3·81 to 9·67)	-0·05 (-0·14 to 0·01)	-0·02 (-0·06 to 0·02)	-0·01 (-0·08 to 0·05)
Tanzania	37·29 (22·01 to 58·56)	619·00 (528·73 to 723·61)	25·30 (14·42 to 31·33)	45·07 (37·24 to 53·68)	-0·04 (-0·08 to 0·00)	-0·03 (-0·04 to -0·02)	-0·11 (-0·16 to -0·08)
Uganda	34·20 (14·59 to 56·25)	637·61 (541·69 to 758·88)	20·55 (15·44 to 28·02)	37·06 (31·87 to 42·59)	-0·07 (-0·15 to -0·01)	0·00 (-0·01 to 0·01)	-0·08 (-0·10 to -0·05)
Zambia	31·51 (22·98 to 40·76)	576·32 (522·21 to 632·42)	14·17 (11·95 to 16·99)	49·94 (43·20 to 57·34)	-0·06 (-0·10 to -0·03)	0·00 (-0·01 to 0·01)	-0·12 (-0·13 to -0·10)
Central Sub-Saharan Africa	33·50 (21·33 to 54·09)	507·14 (450·87 to 571·51)	29·19 (25·56 to 32·80)	25·77 (22·51 to 28·93)	-0·07 (-0·12 to -0·02)	-0·04 (-0·05 to -0·03)	-0·07 (-0·08 to -0·05)
Angola	9·74 (5·16 to 15·82)	119·46 (94·29 to 148·35)	4·92 (2·67 to 7·16)	27·19 (21·87 to 32·85)	-0·03 (-0·10 to 0·02)	0·02 (0·00 to 0·04)	-0·01 (-0·08 to 0·04)
Central African Republic	4·30 (2·14 to 7·40)	59·26 (48·43 to 72·27)	3·39 (2·63 to 4·06)	25·03 (20·35 to 30·12)	-0·02 (-0·08 to 0·03)	-0·04 (-0·06 to -0·02)	-0·08 (-0·10 to -0·05)
Congo	3·18 (1·63 to 4·85)	41·58 (30·91 to 51·69)	2·38 (1·58 to 3·05)	18·33 (15·71 to 22·05)	-0·03 (-0·07 to 0·01)	-0·02 (-0·05 to -0·00)	-0·06 (-0·10 to -0·04)
Democratic Republic of the Congo	14·42 (4·55 to 33·68)	248·60 (204·48 to 306·31)	16·97 (14·19 to 19·89)	23·99 (18·89 to 29·48)	-0·12 (-0·25 to -0·01)	-0·07 (-0·08 to -0·05)	-0·08 (-0·09 to -0·06)
Equatorial Guinea	0·29 (0·08 to 0·69)	10·67 (8·83 to 12·88)	0·46 (0·23 to 0·69)	25·38 (21·21 to 31·11)	-0·18 (-0·32 to -0·06)	0·02 (0·01 to 0·03)	-0·00 (-0·06 to 0·06)
Gabon	1·56 (0·49 to 3·12)	27·57 (22·32 to 33·39)	1·07 (0·68 to 1·36)	49·51 (42·75 to 57·17)	-0·07 (-0·18 to 0·02)	-0·03 (-0·05 to -0·01)	-0·06 (-0·10 to -0·03)

Appendix Table 3. Data sources used for GBD2015 On-ART Mortality Analysis			
**Source names**	**Mortality and Lost to Follow Up Rates**	**Sex Hazard Ratios**	**Age Hazard Ratios**
Abaasa AM, Todd J, Ekoru K, Kalyango JN, Levin J, Odeke E, Karamagi CAS. Good adherence to HAART and improved survival in a community HIV/AIDS treatment and care programme: the experience of The AIDS Support Organization (TASO), Kampala, Uganda. BMC Health Serv Res. 2008; 241.		X	X
Akilimali PZ, Mutombo PB, Kayembe PK, Kaba DK, Mapatano MA. Les determinants de la survie des patients vivant avec le VIH sous therapie antiretrovirale dans la ville de Goma, RD-Congo. Rev Epidemiol Sante Publique. 2014; 62(3): 201–6.		X	
Allam RR, Murhekar MV, Bhatnagar T, Uthappa CK, Chava N, Rewari BB, Venkatesh S, Mehendale S. Survival probability and predictors of mortality and retention in care among patients enrolled for first-line antiretroviral therapy, Andhra Pradesh, India, 2008-2011.. Trans R Soc Trop Med Hyg. 2014; 108(4): 198–205.	X	X	X
Alvarez-Uria G, Naik PK, Pakam R, Midde M. Factors associated with attrition, mortality, and loss to follow up after antiretroviral therapy initiation: data from an HIV cohort study in India. Glob Health Action. 2013; 21682.	X	X	X
Antiretroviral Therapy Cohort Collaboration (ART-CC). Sex differences in overall and cause-specific mortality among HIV-infected adults on antiretroviral therapy in Europe, Canada and the US. Antivir Ther. 2015; 20(1): 21–8.		X	
Assefa Y, Kiflie A, Tesfaye D, Mariam DH, Kloos H, Edwin W, Laga M, Van Damme W. Outcomes of antiretroviral treatment program in Ethiopia: retention of patients in care is a major challenge and varies across health facilities. BMC Health Serv Res. 2011; 81.	X		
Auld AF, Agolory SG, Shiraishi RW, Wabwire-Mangen F, Kwesigabo G, Mulenga M, Hachizovu S, Asadu E, Tuho MZ, Ettiegne-Traore V, Mbofana F, Okello V, Azih C, Denison JA, Tsui S, Koole O, Kamiru H, Nuwagaba-Biribonwoha H, Alfredo C, Jobarteh K, Odafe S, Onotu D, Ekra KA, Kouakou JS, Ehrenkranz P, Bicego G, Torpey K, Mukadi YD, van Praag E, Menten J, Mastro T, Dukes Hamilton C, Swaminathan M, Dokubo EK, Baughman AL, Spira T, Colebunders R, Bangsberg D, Marlink R, Zee A, Kaplan J, Ellerbrock TV. Antiretroviral therapy enrollment characteristics and outcomes among HIV-infected adolescents and young adults compared with older adults--seven African countries, 2004-2013. MMWR Morb Mortal Wkly Rep. 2014; 63(47): 1097–103.			X
Auld AF, Ekra KA, Shiraishi RW, Tuho MZ, Kouakou JS, Mohamed F, Ettiègne-Traoré V, Sabatier J, Essombo J, Adjorlolo-Johnson G, Marlink R, Ellerbrock TV. Temporal trends in treatment outcomes for HIV-1 and HIV-2-infected adults enrolled in Côte d'Ivoire's national antiretroviral therapy program. PLoS One. 2014; 9(5): e98183.	X		
Auld AF, Kamiru H, Azih C, Baughman AL, Nuwagaba-Biribonwoha H, Ehrenkranz P, Agolory S, Sahabo R, Ellerbrock TV, Okello V, Bicego G. Implementation and Operational Research: Evaluation of Swaziland's Hub-and-Spoke Model for Decentralizing Access to Antiretroviral Therapy Services.. J Acquir Immune Defic Syndr. 2015; 69(1): e1–12.	X	X	
Badie BM, Nabaei G, Rasoolinejad M, Mirzazadeh A, McFarland W. Early loss to follow-up and mortality of HIV-infected patients diagnosed after the era of antiretroviral treatment scale up: a call for re-invigorating the response in Iran. Int J STD AIDS. 2013; 24(12): 926-30.		X	
Biadgilign S, Reda AA, Digaffe T. Predictors of mortality among HIV infected patients taking antiretroviral treatment in Ethiopia: a retrospective cohort study. AIDS Res Ther. 2012; 9(1): 15.		X	
Bisson GP, Gaolathe T, Gross R, Rollins C, Bellamy S, Mogorosi M, Avalos A, Friedman H, Dickinson D, Frank I, Ndwapi N. Overestimates of survival after HAART: implications for global scale-up efforts. PLoS One. 2008; 3(3): e1725.		X	
Blevins M, Jose E, Bilhete FR, Vaz LME, Shepherd BE, Audet CM, Vermund SH, Moon TD. Two-year death and loss to follow-up outcomes by source of referral to HIV care for HIV-infected patients initiating antiretroviral therapy in rural Mozambique. AIDS Res Hum Retroviruses. 2015; 31(2): 198–207.	X	X	X

Boulle A, Schomaker M, May MT, Hogg RS, Shepherd BE, Monge S, Keiser O, Lampe FC, Giddy J, Ndirangu J, Garone D, Fox M, Ingle SM, Reiss P, Dabis F, Costagliola D, Castagna A, Ehren K, Campbell C, Gill MJ, Saag M, Justice AC, Guest J, Crane HM, Egger M, Sterne JAC. Mortality in patients with HIV-1 infection starting antiretroviral therapy in South Africa, Europe, or North America: a collaborative analysis of prospective studies. PLoS Med. 2014; 11(9): e1001718.	X		
Boulle A, Van Cutsem G, Hilderbrand K, Cragg C, Abrahams M, Mathee S, Ford N, Knight L, Osler M, Myers J, Goemaere E, Coetzee D, Maartens G. Seven-year experience of a primary care antiretroviral treatment programme in Khayelitsha, South Africa. AIDS. 2010; 24(4): 563-72.	X	X	X
Brennan AT, Maskew M, Sanne I, Fox MP. The interplay between CD4 cell count, viral load suppression and duration of antiretroviral therapy on mortality in a resource-limited setting. Trop Med Int Health. 2013; 18(5): 619-31.		X	X
Brinkhof MWG, Boulle A, Weigel R, Messou E, Mathers C, Orrell C, Dabis F, Pascoe M, Egger M, International Epidemiological Databases to Evaluate AIDS (IeDEA). Mortality of HIV-infected patients starting antiretroviral therapy in sub-Saharan Africa: comparison with HIV-unrelated mortality. PLoS Med. 2009; 6(4): e1000066.		X	X
Brinkhof MWG, Dabis F, Myer L, Bangsberg DR, Boulle A, Nash D, Schechter M, Laurent C, Keiser O, May M, Sprinz E, Egger M, Anglaret X, ART-LINC, IeDEA. Early loss of HIV-infected patients on potent antiretroviral therapy programmes in lower-income countries. Bull World Health Organ. 2008; 86(7): 559-67.	X	X	X
Chang LW, Alamo S, Guma S, Christopher J, Suntoke T, Omasete R, Montis JP, Quinn TC, Juncker M, Reynolds SJ. Two-year virologic outcomes of an alternative AIDS care model: evaluation of a peer health worker and nurse-staffed community-based program in Uganda. J Acquir Immune Defic Syndr. 2009; 50(3): 276-82.	X		
Chasombat S, McConnell MS, Siangphoe U, Yuktanont P, Jirawattanapisal T, Fox K, Thanprasertsuk S, Mock PA, Ningsanond P, Lertpiriyasuwat C, Pinyopornpanich S. National expansion of antiretroviral treatment in Thailand, 2000-2007: program scale-up and patient outcomes. J Acquir Immune Defic Syndr. 2009; 50(5): 506-12.		X	X
Chen J, Yu B, Wang Y, Tang M, Hu Y, Cai T, Zhang F, Zinkernagel D von, Harwell JI, Huang ZJ. Expansion of HIV care and treatment in Yunnan Province, China: Treatment outcomes with scale up of combination antiretroviral therapy. AIDS Care. 2014; 26(5): 633–41.		X	X
Chen SC-C, Yu JK-L, Harries AD, Bong C-N, Kolola-Dzimadzi R, Tok T-S, King C-C, Wang J-D. Increased mortality of male adults with AIDS related to poor compliance to antiretroviral therapy in Malawi. Trop Med Int Health. 2008; 13(4): 513-9.		X	X
Cheng W, Wu Y, Wen Y, Ma Y, Zhao D, Dou Z, Zhang W, Bulterys M, Zhang F. Cotrimoxazole prophylaxis and antiretroviral therapy: an observational cohort study in China. Bull World Health Organ. 2015; 93(3): 152–60.		X	X
Chi BH, Mwango A, Giganti M, Mulenga LB, Tambatamba-Chapula B, Reid SE, Bolton-Moore C, Chintu N, Mulenga PL, Stringer EM, Sheneberger R, Mwaba P, Stringer JSA. Early clinical and programmatic outcomes with tenofovir-based antiretroviral therapy in Zambia. J Acquir Immune Defic Syndr. 2010; 54(1): 63-70.		X	X
Corey DM, Kim HW, Salazar R, Illescas R, Villena J, Gutierrez L, Sanchez J, Tabet SR. Brief report: effectiveness of combination antiretroviral therapy on survival and opportunistic infections in a developing world setting: an observational cohort study. J Acquir Immune Defic Syndr. 2007; 44(4): 451-5.	X		
Cornell M, Grimsrud A, Fairall L, Fox MP, van Cutsem G, Giddy J, Wood R, Prozesky H, Mohapi L, Graber C, Egger M, Boulle A, Myer L, International Epidemiologic Databases to Evaluate AIDS Southern Africa (IeDEA-SA) Collaboration. Temporal changes in programme outcomes among adult patients initiating antiretroviral therapy across South Africa, 2002-2007. AIDS. 2010; 24(14): 2263-70.	X		
Cornell M, Lessells R, Fox MP, Garone DB, Giddy J, Fenner L, Myer L, Boulle A. Mortality Among Adults Transferred and Lost to Follow-up From Antiretroviral Therapy Programmes in South Africa: A Multicenter Cohort Study. JAIDS. 2014; 67(2): 67-75	X		
Cornell M, Schomaker M, Garone DB, Giddy J, Hoffmann CJ, Lessells R, Maskew M, Prozesky H, Wood R, Johnson LF, Egger M, Boulle A, Myer L, International Epidemiologic Databases to Evaluate AIDS Southern Africa Collaboration. Gender differences in survival among adult patients starting antiretroviral therapy in South Africa: a multicentre cohort study. PLoS Med. 2012; 9(9): e1001304.		X	

DeSilva MB, Merry SP, Fischer PR, Rohrer JE, Isichei CO, Cha SS. Youth, unemployment, and male gender predict mortality in AIDS patients started on HAART in Nigeria. AIDS Care. 2009; 21(1): 70-7.		X	X
Edwards JK, Cole SR, Martin JN, Moore R, Mathews WC, Kitahata M, Eron JJ, Saag M, Mugavero MJ, CNICS Investigators. Dynamic Visual Display of Treatment Response in HIV-Infected Adults. Clin Infect Dis. 2015; 61(1): e1–4.	X		
Eguzo KN, Lawal AK, Eseigbe CE, Umezurike CC. Determinants of Mortality among Adult HIV-Infected Patients on Antiretroviral Therapy in a Rural Hospital in Southeastern Nigeria: A 5-Year Cohort Study. AIDS Res Treat. 2014.			X
Fatti G, Grimwood A, Bock P. Better antiretroviral therapy outcomes at primary healthcare facilities: an evaluation of three tiers of ART services in four South African provinces. PLoS One. 2010; 5(9): e12888.		X	
Fatti G, Mothibi E, Meintjes G, Grimwood A. Antiretroviral Treatment Outcomes amongst Older Adults in a Large Multicentre Cohort in South Africa. PLoS One. 2014; 9(6): e100273.	X		
Ford N, Kranzer K, Hilderbrand K, Jouquet G, Goemaere E, Vlahakis N, Triviño L, Makakole L, Bygrave H. Early initiation of antiretroviral therapy and associated reduction in mortality, morbidity and defaulting in a nurse-managed, community cohort in Lesotho. AIDS. 2010; 24(17): 2645-50.	X	X	X
Fregonese F, Collins IJ, Jourdain G, Lecoeur S, Cressey TR, Ngo-Giang-Houng N, Banchongkit S, Chutanunta A, Techapornroong M, Lallemant M, Program for HIV Prevention and Treatment Study Group. Predictors of 5-year mortality in HIV-infected adults starting highly active antiretroviral therapy in Thailand. J Acquir Immune Defic Syndr. 2012; 60(1): 91-8.		X	
García de Olalla P, Knobel H, Carmona A, Guelar A, López-Colomés JL, Caylà JA. Impact of adherence and highly active antiretroviral therapy on survival in HIV-infected patients. J Acquir Immune Defic Syndr. 2002; 30(1): 105-10.		X	X
Geng EH, Emenyonu N, Bwana MB, Glidden DV, Martin JN. Sampling-based approach to determining outcomes of patients lost to follow-up in antiretroviral therapy scale-up programs in Africa. JAMA. 2008; 300(5): 506-7.	X		
Greenbaum AH, Wilson LE, Keruly JC, Moore RD, Gebo KA. Effect of age and HAART regimen on clinical response in an urban cohort of HIV-infected individuals. AIDS. 2008; 22(17): 2331-9.		X	X
Greig J, Casas EC, O'Brien DP, Mills EJ, Ford N. Association between older age and adverse outcomes on antiretroviral therapy: a cohort analysis of programme data from nine countries. AIDS. 2012; S31-37.		X	X
Grimsrud A, Balkan S, Casas EC, Lujan J, Van Cutsem G, Poulet E, Myer L, Pujades-Rodriguez M. Outcomes of antiretroviral therapy over a 10-year period of expansion: a multicohort analysis of African and Asian HIV programs. J Acquir Immune Defic Syndr. 2014; 67(2): e55–66.	X		
HIV-CAUSAL Collaboration, Ray M, Logan R, Sterne JAC, Hernández-Díaz S, Robins JM, Sabin C, Bansi L, van Sighem A, de Wolf F, Costagliola D, Lanoy E, Bucher HC, von Wyl V, Esteve A, Casbona J, del Amo J, Moreno S, Justice A, Goulet J, Lodi S, Phillips A, Seng R, Meyer L, Pérez-Hoyos S, García de Olalla P, Hernán MA. The effect of combined antiretroviral therapy on the overall mortality of HIV-infected individuals. AIDS. 2010; 24(1): 123-37.	X		
Hambisa MT, Ali A, Dessie Y. Determinants of Mortality among HIV Positives after Initiating Antiretroviral Therapy in Western Ethiopia: A Hospital-Based Retrospective Cohort Study. ISRN AIDS. 2013; 491601.		X	X
Han N, Wright ST, O'Connor CC, Hoy J, Ponnampalavanar S, Grotowski M, Zhao HX, Kamarulzaman A, Australian HIV Observational Database (AHOD), TREAT Asia HIV Observational Database (TAHOD). HIV and aging: insights from the Asia Pacific HIV Observational Database (APHOD). HIV Med. 2015; 16(3): 152–60.			X
Hawkins C, Chalamilla G, Okuma J, Spiegelman D, Hertzmark E, Aris E, Ewald T, Mugusi F, Mtasiwa D, Fawzi W. Sex differences in antiretroviral treatment outcomes among HIV-infected adults in an urban Tanzanian setting. AIDS. 2011; 25(9): 1189-97.		X	
Hermans S, van Leth F, Manabe Y, Hoepelman A, Lange J, Kambugu A. Earlier initiation of antiretroviral therapy, increased tuberculosis case finding and reduced mortality in a setting of improved HIV care: a retrospective cohort study. HIV Med. 2012; 13(6): 337-44.		X	
Hoffmann CJ, Fielding KL, Charalambous S, Innes C, Chaisson RE, Grant AD, Churchyard GJ. Reducing mortality with cotrimoxazole preventive therapy at initiation of antiretroviral therapy in South Africa. AIDS. 2010; 24(11): 1709-16.		X	X

Hoffmann CJ, Fielding KL, Johnston V, Charalambous S, Innes C, Moore RD, Chaisson RE, Grant AD, Churchyard GJ. Changing predictors of mortality over time from cART start: implications for care. J Acquir Immune Defic Syndr. 2011; 58(3): 269-76.		X	
Hoffmann CJ, Schomaker M, Fox MP, Mutevedzi P, Giddy J, Prozesky H, Wood R, Garone DB, Egger M, Boulle A, IeDEA Southern Africa Collaboration. CD4 count slope and mortality in HIV-infected patients on antiretroviral therapy: multicohort analysis from South Africa. J Acquir Immune Defic Syndr. 2013; 63(1): 34-41.		X	X
Jensen-Fangel S, Pedersen L, Pedersen C, Larsen CS, Tauris P, Møller A, Sørensen HT, Obel N. Low mortality in HIV-infected patients starting highly active antiretroviral therapy: a comparison with the general population. AIDS. 2004; 18(1): 89-97.	X	X	X
Johannessen A, Naman E, Ngowi BJ, Sandvik L, Matee MI, Aglen HE, Gundersen SG, Bruun JN. Predictors of mortality in HIV-infected patients starting antiretroviral therapy in a rural hospital in Tanzania. BMC Infect Dis. 2008; 52.		X	
Kanters S, Nansubuga M, Mwehire D, Odiit M, Kasirye M, Musoke W, Druyts E, Yaya S, Funk A, Ford N, Mills EJ. Increased mortality among HIV-positive men on antiretroviral therapy: survival differences between sexes explained by late initiation in Uganda. HIV AIDS (Auckl). 2013; 111-9.		X	
Karcher H, Omondi A, Odera J, Kunz A, Harms G. Risk factors for treatment denial and loss to follow-up in an antiretroviral treatment cohort in Kenya. Trop Med Int Health. 2007; 12(5): 687-94.	X		
Keiser O, Chi BH, Gsponer T, Boulle A, Orrell C, Phiri S, Maxwell N, Maskew M, Prozesky H, Fox MP, Westfall A, Egger M, IeDEA Southern Africa Collaboration. Outcomes of antiretroviral treatment in programmes with and without routine viral load monitoring in Southern Africa. AIDS. 2011; 25(14): 1761-9.	X		
Kiboneka A, Nyatia RJ, Nabiryo C, Anema A, Cooper CL, Fernandes KA, Montaner JSG, Mills EJ. Combination antiretroviral therapy in population affected by conflict: outcomes from large cohort in northern Uganda. BMJ. 2009; b201.		X	
Koenig SP, Rodriguez LA, Bartholomew C, Edwards A, Carmichael TE, Barrow G, Cabié A, Hunter R, Vasquez-Mora G, Quava-Jones A, Adomakoh A, Peter Figueroa J, Liautaud B, Torres M, Pape JW. Long-term antiretroviral treatment outcomes in seven countries in the Caribbean. J Acquir Immune Defic Syndr. 2012; 59(4): e60-71.	X	X	
Kouanda S, Meda IB, Nikiema L, Tiendrebeogo S, Doulougou B, Kaboré I, Sanou MJ, Greenwell F, Soudré R, Sondo B. Determinants and causes of mortality in HIV-infected patients receiving antiretroviral therapy in Burkina Faso: a five-year retrospective cohort study. AIDS Care. 2012; 24(4): 478-90.		X	X
Laurent C, Bourgeois A, Mpoudi-Ngolé E, Ciaffi L, Kouanfack C, Mougnutou R, Nkoué N, Calmy A, Koulla-Shiro S, Delaporte E. Tolerability and effectiveness of first-line regimens combining nevirapine and lamivudine plus zidovudine or stavudine in Cameroon. AIDS Res Hum Retroviruses. 2008; 24(3): 393-9.		X	
Lesko CR, Cole SR, Miller WC, Westreich D, Eron JJ, Adimora AA, Moore RD, Mathews WC, Martin JN, Drozd DR, Kitahata MM, Edwards JK, Mugavero MJ. Ten-year Survival by Race/Ethnicity and Sex Among Treated, HIV-infected Adults in the United States. Clin Infect Dis. 2015; 60(11): 1700–7.		X	
Liao L, Xing H, Su B, Wang Z, Ruan Y, Wang X, Liu Z, Lu Y, Yang S, Zhao Q, Vermund SH, Chen RY, Shao Y. Impact of HIV drug resistance on virologic and immunologic failure and mortality in a cohort of patients on antiretroviral therapy in China. AIDS. 2013; 27(11): 1815-24.		X	
Lima VD, Hogg RS, Harrigan PR, Moore D, Yip B, Wood E, Montaner JSG. Continued improvement in survival among HIV-infected individuals with newer forms of highly active antiretroviral therapy. AIDS. 2007; 21(6): 685-92.		X	
Lohse N, Ladefoged K, Pedersen L, Jensen-Fangel S, Sørensen HT, Obel N. Low effectiveness of highly active antiretroviral therapy and high mortality in the Greenland HIV-infected population. Scand J Infect Dis. 2004; 36(10): 738-42.	X		
Lowrance D, Makombe S, Harries A, Yu J, Aberle-Grasse J, Eiger O, Shiraishi R, Marston B, Ellerbrock T, Libamba E. Lower early mortality rates among patients receiving antiretroviral treatment at clinics offering cotrimoxazole prophylaxis in Malawi. J Acquir Immune Defic Syndr. 2007; 46(1): 56-61.	X		

Lowrance DW, Ndamage F, Kayirangwa E, Ndagije F, Lo W, Hoover DR, Hanson J, Elul B, Ayaba A, Ellerbrock T, Rukundo A, Shumbusho F, Nash D, Mugabo J, Assimwe A. Adult clinical and immunologic outcomes of the national antiretroviral treatment program in Rwanda during 2004-2005. J Acquir Immune Defic Syndr. 2009; 52(1): 49-55.		X	
Luz PM, Bruyand M, Ribeiro S, Bonnet F, Moreira RI, Hessamfar M, Campos DP, Greib C, Cazanave C, Veloso VG, Dabis F, Grinsztejn B, Chêne G, IPEC/FIOCRUZ Cohort and the Aquitaine ANRS C03 Study Group. AIDS and non-AIDS severe morbidity associated with hospitalizations among HIV-infected patients in two regions with universal access to care and antiretroviral therapy, France and Brazil, 2000-2008: hospital-based cohort studies. BMC Infect Dis. 2014; 278.		X	X
MacPherson P, Moshabela M, Martinson N, Pronyk P. Mortality and loss to follow-up among HAART initiators in rural South Africa. Trans R Soc Trop Med Hyg. 2009; 103(6): 588-93.		X	
Maman D, Pujades-Rodriguez M, Nicholas S, McGuire M, Szumilin E, Ecochard R, Etard J-F. Response to antiretroviral therapy: improved survival associated with CD4 above 500?cells/?l. AIDS. 2012; 26(11): 1393-8.		X	X
Marston BJ, Macharia DK, Nga'nga L, Wangai M, Ilako F, Muhenje O, Kjaer M, Isavwa A, Kim A, Chebet K, Decock KM, Weidle PJ. A program to provide antiretroviral therapy to residents of an urban slum in nairobi, kenya. J Int Assoc Physicians AIDS Care (Chic). 2007; 6(2): 106-12.	X		
Martin LJ, Houston S, Yasui Y, Wild TC, Saunders LD. All-cause and HIV-related mortality rates among HIV-infected patients after initiating highly active antiretroviral therapy: the impact of Aboriginal ethnicity and injection drug use. Can J Public Health. 2011; 102(2): 90-6.			X
May M, Boulle A, Phiri S, Messou E, Myer L, Wood R, Keiser O, Sterne JAC, Dabis F, Egger M, IeDEA Southern Africa and West Africa. Prognosis of patients with HIV-1 infection starting antiretroviral therapy in sub-Saharan Africa: a collaborative analysis of scale-up programmes. Lancet. 2010; 376(9739): 449-57.		X	X
May MT, Sterne JAC, Costagliola D, Sabin CA, Phillips AN, Justice AC, Dabis F, Gill J, Lundgren J, Hogg RS, de Wolf F, Fätkenheuer G, Staszewski S, d' Arminio Monforte A, Egger M, Antiretroviral Therapy (ART) Cohort Collaboration. HIV treatment response and prognosis in Europe and North America in the first decade of highly active antiretroviral therapy: a collaborative analysis. Lancet. 2006; 368(9534): 451-8.	X		
McManus H, O'Connor CC, Boyd M, Broom J, Russell D, Watson K, Roth N, Read PJ, Petoumenos K, Law MG, Australian HIV Observational Database. Long-term survival in HIV positive patients with up to 15 Years of antiretroviral therapy. PLoS One. 2012; 7(11): e48839.		X	
Mills EJ, Bakanda C, Birungi J, Chan K, Ford N, Cooper CL, Nachega JB, Dybul M, Hogg RS. Life expectancy of persons receiving combination antiretroviral therapy in low-income countries: a cohort analysis from Uganda. Ann Intern Med. 2011; 155(4): 209-16.		X	X
Moore AL, Kirk O, Johnson AM, Katlama C, Blaxhult A, Dietrich M, Colebunders R, Chiesi A, Lungren JD, Phillips AN, EuroSIDA group. Virologic, immunologic, and clinical response to highly active antiretroviral therapy: the gender issue revisited. J Acquir Immune Defic Syndr. 2003; 32(4): 452-61.	X	X	
Mugavero MJ, Napravnik S, Cole SR, Eron JJ, Lau B, Crane HM, Kitahata MM, Willig JH, Moore RD, Deeks SG, Saag MS, Centers for AIDS Research Network of Integrated Clinical Systems (CNICS) Cohort Study. Viremia copy-years predicts mortality among treatment-naive HIV-infected patients initiating antiretroviral therapy. Clin Infect Dis. 2011; 53(9): 927-35.		X	
Mugavero MJ, Westfall AO, Cole SR, Geng EH, Crane HM, Kitahata MM, Mathews WC, Napravnik S, Eron JJ, Moore RD, Keruly JC, Mayer KH, Giordano TP, Raper JL, Centers for AIDS Research Network of Integrated Clinical Systems (CNICS). Beyond core indicators of retention in HIV care: missed clinic visits are independently associated with all-cause mortality. Clin Infect Dis. 2014; 59(10): 1471–9.		X	
Mugisha V, Teasdale CA, Wang C, Lahuerta M, Nuwagaba-Biribonwoha H, Tayebwa E, Ingabire E, Ingabire P, Sahabo R, Twyman P, Abrams EJ, Identifying Optimal Models for HIV Care in Rwanda Collaboration. Determinants of Mortality and Loss to Follow-Up among Adults Enrolled in HIV Care Services in Rwanda. PLoS One. 2014; 9(1): e85774.	X	X	X
Mujugira A, Wester CW, Kim S, Bussmann H, Gaolathe T. Patients with advanced HIV type 1 infection initiating antiretroviral therapy in Botswana: treatment response and mortality. AIDS Res Hum Retroviruses. 2009; 25(2): 127-33.		X	

Mulissa Z, Jerene D, Lindtjørn B. Patients present earlier and survival has improved, but pre-ART attrition is high in a six-year HIV cohort data from Ethiopia. PLoS One. 2010; 5(10): e13268.		X	X
Mutevedzi PC, Lessells RJ, Heller T, Bärnighausen T, Cooke GS, Newell M-L. Scale-up of a decentralized HIV treatment programme in rural KwaZulu-Natal, South Africa: does rapid expansion affect patient outcomes?. Bull World Health Organ. 2010; 88(8): 593-600.		X	
Nachega JB, Hislop M, Dowdy DW, Lo M, Omer SB, Regensberg L, Chaisson RE, Maartens G. Adherence to highly active antiretroviral therapy assessed by pharmacy claims predicts survival in HIV-infected South African adults. J Acquir Immune Defic Syndr. 2006; 43(1): 78-84.		X	X
Nglazi MD, Lawn SD, Kaplan R, Kranzer K, Orrell C, Wood R, Bekker L-G. Changes in programmatic outcomes during 7 years of scale-up at a community-based antiretroviral treatment service in South Africa. J Acquir Immune Defic Syndr. 2011; 56(1): e1-8.	X	X	X
Odafe S, Idoko O, Badru T, Aiyenigba B, Suzuki C, Khamofu H, Onyekwena O, Okechukwu E, Torpey K, Chabikuli ON. Patients' demographic and clinical characteristics and level of care associated with lost to follow-up and mortality in adult patients on first-line ART in Nigerian hospitals. J Int AIDS Soc. 2012; 15(2): 17424.	X	X	
Okonkwo P, Sagay AS, Agaba PA, Yohanna S, Agbaji OO, Imade GE, Banigbe B, Adeola J, Oyebode TA, Idoko JA, Kanki PJ. Treatment outcomes in a decentralized antiretroviral therapy program: a comparison of two levels of care in north central Nigeria. AIDS Res Treat. 2014; 560623.		X	X
Otwombe KN, Petzold M, Modisenyane T, Martinson NA, Chirwa T. Factors associated with mortality in HIV-infected people in rural and urban South Africa. Glob Health Action. 2014; 25488.		X	
Palombi L, Dorrucci M, Zimba I, Scarcella P, Mancinelli S, Buonomo E, Guidotti G, Marazzi MC, Rezza G. Immunologic response to highly active antiretroviral therapy and mortality reduction in a cohort of human immunodeficiency virus-positive persons in Mozambique. Am J Trop Med Hyg. 2010; 83(5): 1128-32.		X	
Palombi L, Marazzi MC, Guidotti G, Germano P, Buonomo E, Scarcella P, Doro Altan A, Zimba IDVM, San Lio MM, De Luca A, DREAM Program. Incidence and predictors of death, retention, and switch to second-line regimens in antiretroviral- treated patients in sub-Saharan African Sites with comprehensive monitoring availability. Clin Infect Dis. 2009; 48(1): 115-22.	X		
Peterson I, Togun O, de Silva T, Oko F, Rowland-Jones S, Jaye A, Peterson K. Mortality and immunovirological outcomes on antiretroviral therapy in HIV-1 and HIV-2-infected individuals in the Gambia. AIDS. 2011; 25(17): 2167-75.		X	
Pinoges L, Schramm B, Poulet E, Balkan S, Szumilin E, Ferreyra C, Pujades-Rodríguez M. Risk factors and mortality associated with resistance to first-line antiretroviral therapy: multicentric cross-sectional and longitudinal analyses. J Acquir Immune Defic Syndr. 2015; 68(5): 527–35.		X	X
Poka-Mayap V, Pefura-Yone EW, Kengne AP, Kuaban C. Mortality and its determinants among patients infected with HIV-1 on antiretroviral therapy in a referral centre in Yaounde, Cameroon: a retrospective cohort study. BMJ Open. 2013; 3(7).		X	
Rai S, Mahapatra B, Sircar S, Raj PY, Venkatesh S, Shaukat M, Rewari BB. Adherence to Antiretroviral Therapy and Its Effect on Survival of HIV-Infected Individuals in Jharkhand, India. PLoS One. 2013; 8(6): e66860.		X	X
Rasschaert F, Koole O, Zachariah R, Lynen L, Manzi M, Van Damme W. Short and long term retention in antiretroviral care in health facilities in rural Malawi and Zimbabwe. BMC Health Serv Res. 2012; 444.	X		
Reepalu A, Balcha TT, Skogmar S, Jemal ZH, Sturegard E, Medstrand P, Bjorkman P. High rates of virological suppression in a cohort of human immunodeficiency virus-positive adults receiving antiretroviral therapy in ethiopian health centers irrespective of concomitant tuberculosis. Open Forum Infect Dis. 2014; 1(1): ofu039.	X		
Reniers G, Slaymaker E, Nakiyingi-Miiro J, Nyamukapa C, Crampin AC, Herbst K, Urassa M, Otieno F, Gregson S, Sewe M, Michael D, Lutalo T, Hosegood V, Kasamba I, Price A, Nabukalu D, Mclean E, Zaba B, ALPHA Network. Mortality trends in the era of antiretroviral therapy: evidence from the Network for Analysing Longitudinal Population based HIV/AIDS data on Africa (ALPHA). AIDS. 2014; S533–42.		X	
Russell EC, Charalambous S, Pemba L, Churchyard GJ, Grant AD, Fielding K. Low haemoglobin predicts early mortality among adults starting antiretroviral therapy in an HIV care programme in South Africa: a cohort study. BMC Public Health. 2010; 433.		X	X

Sanne IM, Westreich D, Macphail AP, Rubel D, Majuba P, Van Rie A. Long term outcomes of antiretroviral therapy in a large HIV/AIDS care clinic in urban South Africa: a prospective cohort study. J Int AIDS Soc. 2009; 38.	X		
Semeere AS, Lwanga I, Sempa J, Parikh S, Nakasujja N, Cumming R, Kambugu A, Mayanja-Kizza H. Mortality and immunological recovery among older adults on antiretroviral therapy at a large urban HIV clinic in Kampala, Uganda. J Acquir Immune Defic Syndr. 2014; 67(4): 382–9.		X	X
Setegn T, Takele A, Gizaw T, Nigatu D, Haile D. Predictors of Mortality among Adult Antiretroviral Therapy Users in Southeastern Ethiopia: Retrospective Cohort Study. AIDS Res Treat. 2015; 148769.		X	
Seyler C, Anglaret X, Dakoury-Dogbo N, Messou E, Touré S, Danel C, Diakité N, Daudié A, Inwoley A, Maurice C, Tonwe-Gold B, Rouet F, N'Dri-Yoman T, Salamon R, ANRS 1203 Study Group. Medium-term survival, morbidity and immunovirological evolution in HIV-infected adults receiving antiretroviral therapy, Abidjan, Côte d'Ivoire. Antivir Ther. 2003; 8(5): 385-93.		X	
Silverberg MJ, Leyden W, Quesenberry CP, Horberg MA. Race/ethnicity and risk of AIDS and death among HIV-infected patients with access to care. J Gen Intern Med. 2009; 24(9): 1065–72.		X	
Somi G, Keogh SC, Todd J, Kilama B, Wringe A, van den Hombergh J, Malima K, Josiah R, Urassa M, Swai R, Zaba B. Low mortality risk but high loss to follow-up among patients in the Tanzanian national HIV care and treatment programme. Trop Med Int Health. 2012; 17(4): 497-506.	X	X	X
Stringer JSA, Zulu I, Levy J, Stringer EM, Mwango A, Chi BH, Mtonga V, Reid S, Cantrell RA, Bulterys M, Saag MS, Marlink RG, Mwinga A, Ellerbrock TV, Sinkala M. Rapid scale-up of antiretroviral therapy at primary care sites in Zambia: feasibility and early outcomes. JAMA. 2006; 296(7): 782-93.		X	X
Tadesse K, Haile F, Hiruy N. Predictors of mortality among patients enrolled on antiretroviral therapy in Aksum hospital, northern Ethiopia: a retrospective cohort study. PLoS One. 2014; 9(1): e87392.		X	X
Tsertsvadze T, Chkhartishvili N, Sharvadze L, Dvali N, Chokoshvili O, Gabunia P, Abutidze A, Nelson K, Dehovitz J, Del Rio C. Outcomes of Universal Access to Antiretroviral Therapy (ART) in Georgia. AIDS Res Treat. 2011; 621078.		X	
Tuboi SH, Schechter M, McGowan CC, Cesar C, Krolewiecki A, Cahn P, Wolff M, Pape JW, Padgett D, Madero JS, Gotuzzo E, Masys DR, Shepherd BE. Mortality during the first year of potent antiretroviral therapy in HIV-1-infected patients in 7 sites throughout Latin America and the Caribbean. J Acquir Immune Defic Syndr. 2009; 51(5): 615-23.	X	X	
Tweve EN, Kayabu D, Nassari NO, Todd J. Improvement in mortality and retention among adult HIV-infected patients in the first 12 months of antiretroviral therapy in Dodoma urban district,Tanzania. Trop Med Int Health. 2015; 20(6): 791–6.	X		
Van Griensven J, Thai S. Predictors of immune recovery and the association with late mortality while on antiretroviral treatment in Cambodia. Trans R Soc Trop Med Hyg. 2011; 105(12): 694-703.		X	X
Van der Borght SF, Clevenbergh P, Rijckborst H, Nsalou P, Onyia N, Lange JM, de Wit TFR, Van der Loeff MFS. Mortality and morbidity among HIV type-1-infected patients during the first 5 years of a multicountry HIV workplace programme in Africa. Antivir Ther. 2009; 14(1): 63-74.		X	X
Velen K, Lewis JJ, Charalambous S, Grant AD, Churchyard GJ, Hoffmann CJ. Comparison of tenofovir, zidovudine, or stavudine as part of first-line antiretroviral therapy in a resource-limited-setting: a cohort study. PLoS One. 2013; 8(5): e64459.		X	
Vinikoor MJ, Joseph J, Mwale J, Marx MA, Goma FM, Mulenga LB, Stringer JSA, Eron JJ, Chi BH. Age at Antiretroviral Therapy Initiation Predicts Immune Recovery, Death, and Loss to Follow-Up Among HIV-Infected Adults in Urban Zambia. AIDS Res Hum Retroviruses. 2014; 30(10): 949–55.	X		
Vinikoor MJ, Joseph J, Mwale J, Marx MA, Goma FM, Mulenga LB, Stringer JSA, Eron JJ, Chi BH. Age at Antiretroviral Therapy Initiation Predicts Immune Recovery, Death, and Loss to Follow-Up Among HIV-Infected Adults in Urban Zambia.. AIDS Res Hum Retroviruses. 2014; 30(10): 949–55.		X	X
Wandeler G, Keiser O, Pfeiffer K, Pestilli S, Fritz C, Labhardt ND, Mbofana F, Mudyiradima R, Emmel J, Egger M, Ehmer J, SolidarMed ART program and IeDEA-Southern Africa. Outcomes of antiretroviral treatment programs in rural Southern Africa. J Acquir Immune Defic Syndr. 2012; 59(2): e9-16.	X	X	X

Willig JH, Westfall AO, Mugavero M, Nevin CR, Correll T, Duggal A, Guyer W, Saag MS, Juday T. Effect of persistency of first-line HIV antiretroviral therapy on clinical outcomes. AIDS Res Hum Retroviruses. 2013; 29(4): 698-703.		X	
Wolff MJ, Cortés CP, Shepherd BE, Beltrán CJ, Chilean AIDS Cohort Study Group. Long-term outcomes of a national expanded access program to antiretroviral therapy: the Chilean AIDS cohort. J Acquir Immune Defic Syndr. 2010; 55(3): 368-74.		X	
Wubshet M, Berhane Y, Worku A, Kebede Y, Diro E. High loss to followup and early mortality create substantial reduction in patient retention at antiretroviral treatment program in north-west ethiopia. ISRN AIDS. 2012; 721720.	X	X	
Zhu H, Napravnik S, Eron JJ, Cole SR, Ma Y, Wohl DA, Dou Z, Zhang Y, Liu Z, Zhao D, Yu L, Liu X, Cohen MS, Zhang F. Decreasing excess mortality of HIV-infected patients initiating antiretroviral therapy: comparison with mortality in general population in China, 2003-2009. J Acquir Immune Defic Syndr. 2013; 63(5): e150-157.		X	X
Zhu Q, Wang L, Lin W, Bulterys M, Yang W, Sun D, Cui Z, Kaplan J, Kleinman N, Wei X, Chung J, Wang Z. Improved survival with co-trimoxazole prophylaxis among people living with HIV/AIDS who initiated antiretroviral treatment in Henan Province, China. Curr HIV Res. 2014; 12(5): 359–65.		X	
Zhyvytsia D. Mortality and its predictors among highly active antiretroviral therapy naive hiv-infected individuals: data from prospective cohort study in Ukraine. Georgian Med News. 2014; 69–74.		X	

Appendix Table 4. Data sources used in GBD 2015 off-ART analysis

Collaborative Group on AIDS Incubation and HIV Survival * including the CASCADE EU Concerted Action. Time from HIV-1 seroconversion to AIDS and death before widespread use of highly-active antiretroviral therapy: a collaborative re-analysis. Collaborative Group on AIDS Incubation and HIV Survival including the CASCADE EU Concerted Action. Concerted Action on SeroConversion to AIDS and Death in Europe. Lancet 2000; 355: 1131–7.
Deschamps MM, Fitzgerald DW, Pape JW, Johnson WD Jr. HIV infection in Haiti: natural history and disease progression. AIDS 2000; 14: 2515–21.
Glynn JR, Sonnenberg P, Nelson G, Bester A, Shearer S, Murray J. Survival from HIV-1 seroconversion in Southern Africa: a retrospective cohort study in nearly 2000 gold-miners over 10 years of follow-up. AIDS 2007; 21: 625–32.
Isingo R, Zaba B, Marston M, et al. Survival after HIV infection in the pre-antiretroviral therapy era in a rural Tanzanian cohort. AIDS 2007; 21 Suppl 6: S5–S13.
Kilmarx PH, Limpakarnjanarat K, Kaewkungwal J, et al. Disease progression and survival with human immunodeficiency virus type 1 subtype E infection among female sex workers in Thailand. J Infect Dis 2000; 181: 1598–606.
Lutalo T, Gray RH, Wawer M, et al. Survival of HIV-infected treatment-naive individuals with documented dates of seroconversion in Rakai, Uganda. AIDS 2007; 21 Suppl 6: S15–19.
Manaloto CR, Perrault JG, Caringal LT, et al. Natural history of HIV infection in Filipino female commercial sex workers. J Acquir Immune Defic Syndr 1994; 7: 1157–68.
Minga A, Danel C, Abo Y, et al. Progression to WHO criteria for antiretroviral therapy in a 7-year cohort of adult HIV-1 seroconverters in Abidjan, Côte d'Ivoire. Bull World Health Organ 2007; 85: 116–23.
Nelson KE, Costello C, Suriyanon V, Sennun S, Duerr A. Survival of blood donors and their spouses with HIV-1 subtype E (CRF01 A_E) infection in northern Thailand, 1992-2007. AIDS 2007; 21 Suppl 6: S47–54.
Peters PJ, Karita E, Kayitenkore K, et al. HIV-infected Rwandan women have a high frequency of long-term survival. AIDS 2007; 21 Suppl 6: S31–37.
Porter K, Zaba B. The empirical evidence for the impact of HIV on adult mortality in the developing world: data from serological studies. AIDS 2004; 18 Suppl 2: S9–S17.
Rangsin R, Piyaraj P, Sirisanthana T, Sirisopana N, Short O, Nelson KE. The natural history of HIV-1 subtype E infection in young men in Thailand with up to 14 years of follow-up. AIDS 2007; 21 Suppl 6: S39–46.
Van der Paal L, Shafer LA, Todd J, Mayanja BN, Whitworth JAG, Grosskurth H. HIV-1 disease progression and mortality before the introduction of highly active antiretroviral therapy in rural Uganda. AIDS 2007; 21 Suppl 6: S21–29.

Appendix Table 5. HIV-specific mortality for patients not receiving ART in all locations by initial CD4 and age (per 100)				
**Initial CD4**	Age groups			
	**15-24**	**25-34**	**35-44**	**45+**
<50	13.5 (1.6 - 26.3)	13.5 (1.6 - 26.3)	12.7 (1.6 - 33.4)	8.2 (2.0 - 17.4)
50-99	5.9 (1.0 - 11.6)	5.9 (1.0 - 11.6)	5.5 (0.8 - 14.2)	3.7 (0.8 - 8.0)
100-199	2.5 (0.5 - 5.3)	2.5 (0.5 - 5.3)	2.3 (0.3 - 5.7)	1.7 (0.3 - 4.0)
200-249	1.1 (0.3 - 2.4)	1.1 (0.3 - 2.4)	1.0 (0.2 - 2.5)	0.8 (0.1 - 2.1)
250-349	0.5 (0.1 - 1.1)	0.5 (0.1 - 1.1)	0.4 (0.1 - 1.2)	0.4 (0.1 - 1.1)
350-500	0.2 (0.0 - 0.5)	0.2 (0.0 - 0.5)	0.2 (0.0 - 0.6)	0.2 (0.0 - 0.6)
>500	0.1 (0.0 - 0.3)	0.1 (0.0 - 0.3)	0.1 (0.0 - 0.3)	0.1 (0.0 - 0.3)

Appendix Table 6A. HIV-specific mortality for patients on ART in all sub-Saharan African sites by time since ART initiation, sex, initial CD4 and age (per 100)

Time period	Initial CD4	Male					Female				
		**14-24**	**25-34**	**35-44**	**45-54**	**55+**	**14-24**	**25-34**	**35-44**	**45-54**	**55+**
0-6	<50	24.5 (17.5 - 33.3)	21.3 (15.2 - 29.5)	23.0 (16.7 - 31.0)	22.5 (15.4 - 31.3)	27.0 (18.7 - 37.5)	19.1 (13.2 - 26.9)	16.6 (11.7 - 23.5)	17.9 (12.8 - 24.6)	17.6 (11.9 - 25.1)	19.4 (12.4 - 28.7)
0-6	50-99	16.3 (12.5 - 20.9)	14.0 (10.7 - 18.3)	15.2 (11.8 - 18.7)	14.6 (10.9 - 19.3)	16.9 (12.2 - 22.2)	12.6 (9.5 - 16.5)	10.8 (8.1 - 14.3)	11.6 (8.9 - 14.6)	11.4 (8.5 - 15.3)	11.1 (7.1 - 15.6)
0-6	100-199	13.4 (9.9 - 17.3)	11.5 (8.3 - 15.2)	12.4 (9.3 - 15.8)	11.8 (8.4 - 16.1)	13.2 (8.7 - 18.2)	10.3 (7.5 - 13.5)	8.8 (6.3 - 11.9)	9.4 (6.9 - 12.2)	9.2 (6.5 - 12.7)	8.1 (4.5 - 12.3)
0-6	200-249	11.9 (8.6 - 15.7)	10.1 (7.2 - 13.8)	10.9 (7.9 - 14.2)	10.4 (7.0 - 14.3)	11.3 (7.1 - 16.0)	9.1 (6.5 - 12.3)	7.7 (5.4 - 10.8)	8.3 (5.9 - 11.0)	8.1 (5.5 - 11.4)	6.6 (3.3 - 10.5)
0-6	250-349	10.4 (7.2 - 14.1)	8.8 (5.8 - 12.4)	9.5 (6.5 - 12.9)	9.0 (5.7 - 13.1)	9.4 (5.3 - 14.1)	7.9 (5.4 - 10.9)	6.7 (4.4 - 9.7)	7.2 (4.9 - 10.0)	7.0 (4.4 - 10.3)	5.1 (1.8 - 8.8)
0-6	350-500	7.7 (4.4 - 11.7)	6.5 (3.5 - 9.7)	7.0 (3.8 - 10.6)	6.5 (3.1 - 10.2)	6.0 (1.4 - 11.0)	5.9 (3.4 - 9.1)	5.0 (2.6 - 7.6)	5.3 (2.7 - 8.0)	5.1 (2.5 - 8.2)	2.5 (0.0 - 6.4)
0-6	>500	6.0 (1.0 - 10.9)	4.9 (0.6 - 9.1)	5.3 (0.6 - 9.7)	4.8 (0.0 - 9.4)	3.9 (0.0 - 10.2)	4.5 (0.8 - 8.4)	3.8 (0.5 - 7.0)	4.0 (0.4 - 7.6)	3.8 (0.2 - 7.5)	1.4 (0.0 - 5.9)
7-12	<50	8.6 (5.7 - 12.7)	7.2 (4.8 - 10.6)	7.8 (5.2 - 11.1)	7.2 (4.5 - 11.0)	7.1 (3.5 - 12.0)	6.6 (4.3 - 9.8)	5.5 (3.6 - 8.2)	5.9 (3.9 - 8.7)	5.7 (3.5 - 8.6)	3.2 (0.3 - 7.3)
7-12	50-99	6.1 (4.4 - 8.2)	5.1 (3.6 - 7.0)	5.4 (4.0 - 7.2)	4.9 (3.3 - 7.0)	3.8 (1.7 - 6.6)	4.6 (3.4 - 6.3)	3.8 (2.7 - 5.4)	4.1 (2.9 - 5.5)	3.9 (2.6 - 5.5)	0.8 (0.0 - 2.9)
7-12	100-199	5.1 (3.6 - 6.9)	4.2 (2.9 - 6.0)	4.5 (3.2 - 6.2)	3.9 (2.4 - 5.9)	2.5 (0.7 - 5.1)	3.9 (2.7 - 5.3)	3.2 (2.1 - 4.6)	3.3 (2.3 - 4.6)	3.2 (2.0 - 4.7)	0.2 (0.0 - 1.7)
7-12	200-249	4.4 (3.0 - 6.2)	3.6 (2.3 - 5.2)	3.9 (2.6 - 5.4)	3.3 (1.9 - 5.1)	1.7 (0.0 - 3.9)	3.4 (2.2 - 4.8)	2.7 (1.8 - 4.0)	2.9 (1.9 - 4.1)	2.7 (1.6 - 4.0)	0.1 (0.0 - 0.9)
7-12	250-349	3.8 (2.4 - 5.4)	3.0 (1.7 - 4.6)	3.2 (1.9 - 4.6)	2.7 (1.3 - 4.3)	0.9 (0.0 - 3.0)	2.9 (1.8 - 4.2)	2.3 (1.3 - 3.5)	2.4 (1.3 - 3.5)	2.2 (1.1 - 3.6)	0.0 (0.0 - 0.1)
7-12	350-500	2.7 (1.3 - 4.4)	2.1 (0.8 - 3.6)	2.2 (0.9 - 3.8)	1.7 (0.3 - 3.3)	0.2 (0.0 - 1.7)	2.1 (0.9 - 3.4)	1.6 (0.6 - 2.7)	1.6 (0.6 - 2.9)	1.4 (0.4 - 2.7)	0.0 (0.0 - 0.0)
7-12	>500	2.0 (0.1 - 4.2)	1.5 (0.0 - 3.3)	1.6 (0.0 - 3.6)	1.1 (0.0 - 3.0)	0.1 (0.0 - 1.2)	1.5 (0.1 - 3.1)	1.2 (0.0 - 2.5)	1.2 (0.0 - 2.6)	1.0 (0.0 - 2.5)	0.0 (0.0 - 0.0)
13-24	<50	4.4 (3.1 - 6.2)	3.6 (2.4 - 5.1)	3.9 (2.7 - 5.4)	3.3 (1.9 - 4.9)	1.7 (0.0 - 3.8)	3.4 (2.3 - 4.8)	2.7 (1.8 - 3.9)	2.9 (2.0 - 4.1)	2.7 (1.6 - 4.0)	0.1 (0.0 - 0.8)
13-24	50-99	3.1 (2.3 - 4.1)	2.5 (1.8 - 3.3)	2.6 (2.0 - 3.4)	2.1 (1.3 - 3.0)	0.2 (0.0 - 1.1)	2.4 (1.8 - 3.1)	1.9 (1.3 - 2.5)	1.9 (1.4 - 2.5)	1.7 (1.1 - 2.5)	0.0 (0.0 - 0.0)
13-24	100-199	2.5 (1.7 - 3.3)	1.9 (1.3 - 2.7)	2.0 (1.4 - 2.7)	1.5 (0.7 - 2.4)	0.0 (0.0 - 0.2)	1.9 (1.3 - 2.5)	1.5 (1.0 - 2.0)	1.5 (1.0 - 2.0)	1.3 (0.7 - 2.0)	0.0 (0.0 - 0.0)
13-24	200-249	2.1 (1.4 - 2.9)	1.6 (1.0 - 2.3)	1.7 (1.0 - 2.4)	1.1 (0.4 - 1.9)	0.0 (0.0 - 0.0)	1.6 (1.0 - 2.2)	1.2 (0.7 - 1.8)	1.2 (0.7 - 1.8)	1.0 (0.4 - 1.6)	0.0 (0.0 - 0.0)
13-24	250-349	1.7 (1.0 - 2.5)	1.3 (0.6 - 2.0)	1.3 (0.6 - 2.1)	0.7 (0.0 - 1.5)	0.0 (0.0 - 0.0)	1.3 (0.7 - 1.9)	0.9 (0.4 - 1.5)	0.9 (0.4 - 1.5)	0.7 (0.2 - 1.4)	0.0 (0.0 - 0.0)
13-24	350-500	1.1 (0.4 - 2.0)	0.8 (0.1 - 1.5)	0.8 (0.1 - 1.5)	0.3 (0.0 - 1.0)	0.0 (0.0 - 0.0)	0.9 (0.3 - 1.5)	0.6 (0.1 - 1.1)	0.5 (0.0 - 1.1)	0.3 (0.0 - 0.9)	0.0 (0.0 - 0.0)
13-24	>500	0.8 (0.0 - 1.8)	0.5 (0.0 - 1.3)	0.5 (0.0 - 1.3)	0.1 (0.0 - 0.8)	0.0 (0.0 - 0.0)	0.6 (0.0 - 1.4)	0.3 (0.0 - 1.0)	0.3 (0.0 - 1.0)	0.2 (0.0 - 0.8)	0.0 (0.0 - 0.0)

Appendix Table 6B. HIV-specific mortality for patients on ART in all developed sites by time since ART initiation, sex, initial CD4 and age (per 100)

Time period	Initial CD4	Male					Female				
		**15-24**	**25-34**	**35-44**	**45-54**	**55+**	**15-24**	**25-34**	**35-44**	**45-54**	**55+**
0-6	<50	7.6 (3.1 - 16.2)	7.7 (3.9 - 13.9)	8.5 (4.6 - 15.1)	9.9 (4.6 - 19.0)	7.3 (1.9 - 16.5)	7.2 (3.0 - 15.3)	7.3 (3.7 - 13.4)	8.1 (4.4 - 13.8)	9.5 (4.6 - 17.8)	7.1 (2.0 - 15.8)
0-6	50-99	3.7 (1.5 - 7.2)	3.8 (2.0 - 6.7)	4.1 (2.3 - 6.9)	4.7 (2.1 - 9.0)	2.0 (0.0 - 6.2)	3.6 (1.4 - 6.9)	3.6 (2.0 - 6.3)	3.9 (2.3 - 6.6)	4.6 (2.3 - 9.0)	2.0 (0.0 - 6.1)
0-6	100-199	2.3 (0.9 - 4.6)	2.3 (1.2 - 3.9)	2.5 (1.4 - 4.0)	2.8 (1.2 - 5.2)	0.4 (0.0 - 2.2)	2.2 (0.9 - 4.2)	2.2 (1.2 - 3.8)	2.4 (1.4 - 3.8)	2.8 (1.4 - 5.1)	0.5 (0.0 - 2.4)
0-6	200-249	1.4 (0.5 - 2.6)	1.4 (0.7 - 2.3)	1.5 (0.8 - 2.4)	1.5 (0.5 - 3.0)	0.0 (0.0 - 0.0)	1.3 (0.5 - 2.6)	1.3 (0.7 - 2.3)	1.4 (0.8 - 2.4)	1.6 (0.7 - 3.0)	0.0 (0.0 - 0.2)
0-6	250-349	0.9 (0.3 - 1.8)	0.9 (0.4 - 1.6)	0.9 (0.4 - 1.6)	0.9 (0.2 - 2.0)	0.0 (0.0 - 0.0)	0.9 (0.3 - 1.8)	0.9 (0.4 - 1.5)	0.9 (0.4 - 1.6)	1.0 (0.3 - 2.0)	0.0 (0.0 - 0.0)
0-6	350-500	0.6 (0.2 - 1.3)	0.6 (0.2 - 1.1)	0.6 (0.2 - 1.1)	0.5 (0.0 - 1.3)	0.0 (0.0 - 0.0)	0.6 (0.2 - 1.3)	0.6 (0.2 - 1.1)	0.6 (0.3 - 1.0)	0.6 (0.2 - 1.3)	0.0 (0.0 - 0.0)
0-6	>500	0.3 (0.0 - 0.7)	0.3 (0.1 - 0.6)	0.2 (0.0 - 0.6)	0.1 (0.0 - 0.5)	0.0 (0.0 - 0.0)	0.3 (0.1 - 0.7)	0.3 (0.1 - 0.6)	0.3 (0.1 - 0.6)	0.2 (0.0 - 0.7)	0.0 (0.0 - 0.0)
7-12	<50	4.7 (1.7 - 10.7)	4.7 (2.2 - 9.2)	5.1 (2.5 - 9.7)	6.0 (2.5 - 12.4)	3.2 (0.0 - 9.6)	4.5 (1.7 - 9.8)	4.5 (2.1 - 8.9)	4.9 (2.4 - 9.1)	5.8 (2.5 - 11.7)	3.2 (0.0 - 9.5)
7-12	50-99	1.7 (0.6 - 3.3)	1.7 (0.8 - 3.1)	1.8 (0.9 - 3.2)	2.0 (0.7 - 4.0)	0.1 (0.0 - 1.2)	1.6 (0.6 - 3.2)	1.6 (0.8 - 2.9)	1.8 (0.9 - 3.1)	2.0 (0.8 - 4.0)	0.1 (0.0 - 1.3)
7-12	100-199	1.0 (0.4 - 2.0)	1.0 (0.5 - 1.7)	1.0 (0.5 - 1.8)	1.0 (0.3 - 2.1)	0.0 (0.0 - 0.0)	1.0 (0.4 - 1.9)	1.0 (0.5 - 1.7)	1.0 (0.5 - 1.8)	1.1 (0.4 - 2.2)	0.0 (0.0 - 0.0)
7-12	200-249	0.6 (0.2 - 1.2)	0.6 (0.2 - 1.1)	0.6 (0.2 - 1.1)	0.5 (0.0 - 1.2)	0.0 (0.0 - 0.0)	0.6 (0.2 - 1.2)	0.6 (0.3 - 1.0)	0.6 (0.3 - 1.1)	0.6 (0.2 - 1.3)	0.0 (0.0 - 0.0)
7-12	250-349	0.4 (0.1 - 0.8)	0.4 (0.1 - 0.7)	0.3 (0.1 - 0.7)	0.2 (0.0 - 0.7)	0.0 (0.0 - 0.0)	0.4 (0.1 - 0.8)	0.4 (0.1 - 0.7)	0.4 (0.1 - 0.7)	0.3 (0.0 - 0.8)	0.0 (0.0 - 0.0)
7-12	350-500	0.2 (0.0 - 0.6)	0.2 (0.0 - 0.5)	0.2 (0.0 - 0.5)	0.1 (0.0 - 0.4)	0.0 (0.0 - 0.0)	0.3 (0.1 - 0.6)	0.3 (0.1 - 0.5)	0.2 (0.0 - 0.5)	0.2 (0.0 - 0.5)	0.0 (0.0 - 0.0)
7-12	>500	0.1 (0.0 - 0.4)	0.1 (0.0 - 0.3)	0.1 (0.0 - 0.3)	0.0 (0.0 - 0.1)	0.0 (0.0 - 0.0)	0.2 (0.0 - 0.4)	0.1 (0.0 - 0.3)	0.1 (0.0 - 0.3)	0.1 (0.0 - 0.3)	0.0 (0.0 - 0.0)
13-24	<50	1.7 (0.6 - 3.8)	1.7 (0.8 - 3.2)	1.8 (0.9 - 3.5)	2.0 (0.7 - 4.5)	0.1 (0.0 - 1.4)	1.7 (0.7 - 3.5)	1.7 (0.8 - 3.1)	1.8 (0.9 - 3.4)	2.1 (0.8 - 4.3)	0.2 (0.0 - 1.5)
13-24	50-99	1.0 (0.4 - 2.1)	1.0 (0.5 - 1.7)	1.0 (0.5 - 1.8)	1.0 (0.3 - 2.3)	0.0 (0.0 - 0.0)	1.0 (0.4 - 2.0)	1.0 (0.5 - 1.7)	1.0 (0.6 - 1.8)	1.1 (0.5 - 2.3)	0.0 (0.0 - 0.0)
13-24	100-199	0.7 (0.2 - 1.4)	0.7 (0.3 - 1.1)	0.7 (0.3 - 1.1)	0.6 (0.1 - 1.4)	0.0 (0.0 - 0.0)	0.7 (0.3 - 1.3)	0.7 (0.3 - 1.1)	0.7 (0.4 - 1.1)	0.7 (0.3 - 1.4)	0.0 (0.0 - 0.0)
13-24	200-249	0.5 (0.2 - 1.1)	0.5 (0.2 - 0.9)	0.5 (0.2 - 0.9)	0.4 (0.0 - 1.0)	0.0 (0.0 - 0.0)	0.5 (0.2 - 1.1)	0.5 (0.2 - 0.9)	0.5 (0.3 - 0.9)	0.5 (0.1 - 1.1)	0.0 (0.0 - 0.0)
13-24	250-349	0.4 (0.1 - 0.9)	0.4 (0.1 - 0.7)	0.4 (0.1 - 0.7)	0.2 (0.0 - 0.7)	0.0 (0.0 - 0.0)	0.4 (0.2 - 0.9)	0.4 (0.2 - 0.7)	0.4 (0.2 - 0.7)	0.4 (0.1 - 0.9)	0.0 (0.0 - 0.0)
13-24	350-500	0.3 (0.1 - 0.7)	0.3 (0.1 - 0.5)	0.2 (0.0 - 0.5)	0.1 (0.0 - 0.5)	0.0 (0.0 - 0.0)	0.3 (0.1 - 0.7)	0.3 (0.1 - 0.5)	0.3 (0.1 - 0.6)	0.2 (0.0 - 0.6)	0.0 (0.0 - 0.0)
13-24	>500	0.1 (0.0 - 0.4)	0.1 (0.0 - 0.3)	0.1 (0.0 - 0.3)	0.0 (0.0 - 0.2)	0.0 (0.0 - 0.0)	0.2 (0.0 - 0.4)	0.2 (0.0 - 0.4)	0.1 (0.0 - 0.3)	0.1 (0.0 - 0.3)	0.0 (0.0 - 0.0)

Appendix Table 6C. HIV-specific mortality for patients on ART in all developing sites outside of sub-Saharan African by time since ART initiation, sex, initial CD4 and age (per 100)

Time period	Initial CD4	Male					Female				
		15-24	25-34	35-44	45-54	55+	15-24	25-34	35-44	45-54	55+
0-6	<50	25.2 (16.6 - 36.5)	23.3 (15.3 - 33.1)	24.9 (16.5 - 35.3)	25.0 (16.2 - 36.2)	29.5 (18.7 - 42.5)	16.0 (10.3 - 23.9)	14.7 (9.4 - 21.7)	15.8 (10.3 - 23.3)	15.8 (10.1 - 23.8)	18.0 (10.6 - 27.6)
0-6	50-99	18.1 (12.7 - 24.8)	16.6 (11.6 - 22.5)	17.8 (12.5 - 24.2)	17.7 (12.1 - 25.0)	20.4 (13.6 - 28.6)	11.3 (7.8 - 16.0)	10.3 (7.1 - 14.8)	11.1 (7.7 - 15.5)	11.0 (7.4 - 15.8)	11.8 (7.1 - 17.7)
0-6	100-199	15.1 (10.5 - 21.4)	13.8 (9.4 - 19.5)	14.8 (10.2 - 20.9)	14.7 (9.8 - 20.9)	16.6 (10.4 - 24.3)	9.4 (6.3 - 13.5)	8.5 (5.7 - 12.4)	9.2 (6.2 - 13.2)	9.1 (6.0 - 13.3)	9.2 (5.2 - 14.5)
0-6	200-249	13.3 (8.7 - 19.0)	12.2 (7.9 - 17.9)	13.0 (8.5 - 18.6)	12.9 (8.1 - 18.9)	14.2 (8.2 - 21.8)	8.2 (5.4 - 12.0)	7.5 (4.8 - 11.1)	8.0 (5.3 - 11.7)	7.9 (5.0 - 12.1)	7.7 (3.9 - 12.8)
0-6	250-349	11.5 (6.8 - 17.8)	10.5 (6.2 - 16.3)	11.2 (6.7 - 17.4)	11.1 (6.5 - 17.3)	11.9 (5.8 - 19.8)	7.1 (4.0 - 11.3)	6.4 (3.7 - 10.1)	6.9 (4.0 - 10.9)	6.7 (3.8 - 10.8)	6.2 (2.1 - 11.6)
0-6	350-500	8.4 (4.1 - 14.2)	7.6 (3.6 - 13.4)	8.1 (3.8 - 14.1)	7.9 (3.5 - 14.1)	7.7 (2.0 - 15.9)	5.1 (2.4 - 8.9)	4.6 (2.1 - 8.3)	4.9 (2.3 - 8.7)	4.7 (2.0 - 8.7)	3.5 (0.0 - 8.7)
0-6	>500	6.2 (0.4 - 12.8)	5.6 (0.3 - 12.2)	5.9 (0.2 - 12.8)	5.6 (0.0 - 12.9)	5.0 (0.0 - 14.1)	3.7 (0.2 - 8.0)	3.4 (0.2 - 7.4)	3.6 (0.1 - 8.0)	3.4 (0.0 - 7.8)	2.1 (0.0 - 7.7)
7-12	<50	10.0 (6.6 - 14.9)	9.1 (6.0 - 13.4)	9.7 (6.5 - 14.3)	9.5 (6.1 - 14.4)	9.9 (5.6 - 16.2)	6.1 (4.0 - 9.2)	5.5 (3.6 - 8.3)	5.9 (3.9 - 8.9)	5.7 (3.5 - 9.0)	4.9 (2.1 - 9.0)
7-12	50-99	7.6 (5.3 - 10.5)	6.9 (4.8 - 9.6)	7.3 (5.2 - 10.1)	7.1 (4.8 - 10.2)	6.7 (3.7 - 10.5)	4.6 (3.2 - 6.5)	4.2 (2.9 - 5.9)	4.4 (3.2 - 6.2)	4.2 (2.8 - 6.3)	2.9 (1.0 - 5.4)
7-12	100-199	6.6 (4.5 - 9.3)	6.0 (4.0 - 8.3)	6.3 (4.3 - 8.9)	6.1 (3.9 - 8.9)	5.4 (2.6 - 8.8)	4.0 (2.7 - 5.7)	3.6 (2.4 - 5.2)	3.8 (2.6 - 5.4)	3.6 (2.3 - 5.4)	2.0 (0.3 - 4.2)
7-12	200-249	5.9 (3.9 - 8.5)	5.3 (3.4 - 7.6)	5.6 (3.6 - 8.2)	5.4 (3.3 - 8.0)	4.4 (1.8 - 8.1)	3.6 (2.3 - 5.2)	3.2 (2.0 - 4.7)	3.4 (2.2 - 5.0)	3.2 (1.9 - 4.9)	1.5 (0.0 - 3.6)
7-12	250-349	5.2 (3.3 - 7.7)	4.7 (2.9 - 7.1)	4.9 (3.0 - 7.5)	4.7 (2.7 - 7.4)	3.5 (1.0 - 7.1)	3.1 (1.9 - 4.8)	2.8 (1.7 - 4.3)	3.0 (1.8 - 4.5)	2.7 (1.6 - 4.5)	1.0 (0.0 - 3.1)
7-12	350-500	4.0 (2.0 - 6.7)	3.6 (1.8 - 6.0)	3.7 (1.8 - 6.3)	3.4 (1.4 - 6.2)	1.9 (0.0 - 5.2)	2.4 (1.2 - 4.0)	2.1 (1.0 - 3.6)	2.2 (1.0 - 3.9)	2.0 (0.7 - 3.7)	0.3 (0.0 - 2.1)
7-12	>500	3.1 (0.4 - 6.1)	2.8 (0.3 - 5.5)	2.8 (0.2 - 5.8)	2.5 (0.0 - 5.6)	1.2 (0.0 - 4.6)	1.9 (0.2 - 3.7)	1.6 (0.1 - 3.4)	1.7 (0.1 - 3.7)	1.4 (0.0 - 3.4)	0.2 (0.0 - 1.7)
13-24	<50	8.6 (5.5 - 13.4)	7.7 (4.9 - 12.2)	8.2 (5.2 - 13.2)	8.0 (4.9 - 13.0)	8.0 (3.9 - 14.3)	5.2 (3.2 - 8.4)	4.7 (2.9 - 7.7)	5.0 (3.1 - 8.1)	4.8 (2.8 - 8.1)	3.7 (1.1 - 8.1)
13-24	50-99	6.6 (4.3 - 9.7)	5.9 (3.9 - 8.7)	6.2 (4.1 - 9.3)	6.0 (3.8 - 9.3)	5.3 (2.4 - 9.5)	4.0 (2.6 - 6.0)	3.6 (2.3 - 5.3)	3.8 (2.5 - 5.7)	3.6 (2.2 - 5.6)	2.0 (0.1 - 4.5)
13-24	100-199	5.5 (3.7 - 8.1)	4.9 (3.2 - 7.2)	5.2 (3.4 - 7.7)	4.9 (3.1 - 7.7)	3.9 (1.4 - 7.3)	3.3 (2.2 - 4.8)	3.0 (1.9 - 4.5)	3.1 (2.0 - 4.7)	2.9 (1.8 - 4.5)	1.1 (0.0 - 3.2)
13-24	200-249	4.8 (3.1 - 7.2)	4.3 (2.7 - 6.5)	4.5 (3.0 - 6.8)	4.3 (2.5 - 6.7)	3.0 (0.7 - 6.1)	2.9 (1.9 - 4.4)	2.6 (1.6 - 3.9)	2.7 (1.7 - 4.2)	2.5 (1.4 - 4.1)	0.7 (0.0 - 2.6)
13-24	250-349	4.2 (2.6 - 6.4)	3.7 (2.2 - 5.8)	3.9 (2.3 - 6.1)	3.6 (1.9 - 5.9)	2.1 (0.0 - 5.1)	2.5 (1.5 - 4.0)	2.2 (1.3 - 3.6)	2.3 (1.4 - 3.7)	2.1 (1.1 - 3.6)	0.3 (0.0 - 2.0)
13-24	350-500	3.0 (1.4 - 5.0)	2.6 (1.1 - 4.5)	2.7 (1.1 - 4.7)	2.4 (0.7 - 4.3)	0.8 (0.0 - 3.1)	1.8 (0.8 - 3.0)	1.6 (0.7 - 2.7)	1.6 (0.6 - 2.9)	1.3 (0.4 - 2.6)	0.1 (0.0 - 0.7)
13-24	>500	2.2 (0.1 - 4.6)	1.9 (0.0 - 4.2)	1.9 (0.0 - 4.2)	1.6 (0.0 - 4.0)	0.4 (0.0 - 2.7)	1.3 (0.0 - 2.7)	1.1 (0.0 - 2.5)	1.1 (0.0 - 2.5)	0.9 (0.0 - 2.4)	0.0 (0.0 - 0.3)

Appendix Table 7. Socio-Demographic Index (SDI) values for countries and territories, 1980, 1985, 1990, 1995, 2000, 2005, 2010, 2015.								
**Location**	**1980**	**1985**	**1990**	**1995**	**2000**	**2005**	**2010**	**2015**
**Global**	**0.419890257**	**0.448869963**	**0.481070827**	**0.51461565**	**0.545564774**	**0.574763351**	**0.607931078**	**0.638088616**
**Southeast Asia, East Asia, and Oceania**	**0.334507674**	**0.386478364**	**0.438002768**	**0.495739432**	**0.545405255**	**0.586854227**	**0.630096557**	**0.667211743**
**East Asia**	**0.324870066**	**0.378349089**	**0.433848362**	**0.496728185**	**0.5504169**	**0.595148343**	**0.641304049**	**0.679168909**
China	0.316029107	0.370025935	0.426490088	0.490858491	0.546349149	0.592117095	0.639340838	0.67797786
North Korea	0.531521701	0.558140029	0.581680657	0.588037136	0.557880801	0.544909739	0.553809102	0.565185237
Taiwan	0.611394882	0.663318751	0.705946966	0.745573158	0.779946626	0.822771092	0.848529005	0.864797888
**Southeast Asia**	**0.360992193**	**0.408397006**	**0.449575637**	**0.494458939**	**0.534872048**	**0.569601545**	**0.607245062**	**0.643602267**
Cambodia	0.206297496	0.207615809	0.241209413	0.282367235	0.326515416	0.382164263	0.438281569	0.486077129
Indonesia	0.361205044	0.415428	0.466757836	0.520829484	0.559426107	0.583866374	0.616653524	0.652295062
Laos	0.205809999	0.232776436	0.260227332	0.304400051	0.359807977	0.408107548	0.457248111	0.507722803
Malaysia	0.495928312	0.533137616	0.566011505	0.607094501	0.657028291	0.70262728	0.738329866	0.766902072
Maldives	0.258235896	0.289865922	0.357101519	0.434564955	0.50159269	0.553408613	0.592863823	0.622891153
Mauritius	0.482713855	0.520462471	0.558800525	0.602473574	0.639994338	0.671110794	0.703809791	0.735295853
Myanmar	0.183512584	0.230134304	0.248965591	0.271740612	0.323995308	0.399136961	0.469029233	0.519950083
Philippines	0.44857096	0.481736957	0.506207424	0.53144151	0.554331907	0.580222533	0.611482246	0.645389715
Sri Lanka	0.465829322	0.498163977	0.535458053	0.567485691	0.602854815	0.628971188	0.66405104	0.705374713
Seychelles	0.50981896	0.562151828	0.60367661	0.655336965	0.688267756	0.712016815	0.729072162	0.758544047
Thailand	0.439814384	0.490993431	0.53782009	0.586131918	0.618254763	0.646817521	0.676011278	0.704955714
Timor-Leste	0.201456447	0.233683742	0.265231568	0.288778259	0.24020074	0.326965193	0.409892818	0.449924003
Vietnam	0.293664101	0.34660481	0.389770741	0.445522579	0.504668225	0.548955285	0.590490003	0.628293292
**Oceania**	**0.330511886**	**0.353846236**	**0.377116602**	**0.404412276**	**0.424392427**	**0.43623478**	**0.459143022**	**0.494391389**
American Samoa	0.615586933	0.617712928	0.642700436	0.675309109	0.708945163	0.712817668	0.713096748	0.7135516
Federated States of Micronesia	0.421789042	0.455455893	0.488926129	0.518911645	0.547985774	0.578873624	0.603871751	0.624230673
Fiji	0.51494507	0.545566268	0.565653763	0.593209981	0.620730952	0.646434	0.67197435	0.693405746
Guam	0.704613932	0.736781418	0.768124962	0.798490891	0.82281075	0.846398009	0.867234293	0.884123883
Kiribati	0.417016933	0.39909285	0.400226909	0.408038629	0.432723924	0.453871595	0.46597802	0.477838141
Marshall Islands	0.355412888	0.389592959	0.431191762	0.479957592	0.49577	0.521367611	0.554770782	0.591553873
Northern Mariana Islands	0.646306198	0.721659486	0.774792442	0.797415957	0.833444581	0.851875562	0.847279785	0.84055174
Papua New Guinea	0.271558152	0.292515491	0.317323712	0.345548603	0.366376862	0.379691416	0.405917906	0.448066022
Samoa	0.429462288	0.465919203	0.499791402	0.528806985	0.55408114	0.5847853	0.613086632	0.636681279
Solomon Islands	0.253866136	0.283208014	0.309757249	0.35871649	0.392909632	0.395404665	0.423452452	0.461434832
Tonga	0.391012059	0.448094567	0.505564545	0.53148532	0.552248476	0.58010384	0.603786919	0.622368406
Vanuatu	0.345226973	0.373088266	0.395693064	0.422961726	0.45183673	0.479880618	0.512340212	0.535971122
**Central Europe, Eastern Europe, and Central Asia**	**0.654509482**	**0.67583287**	**0.704032485**	**0.720365581**	**0.72915229**	**0.755855134**	**0.789328603**	**0.81582412**
**Central Asia**	**0.551645216**	**0.576158611**	**0.604292276**	**0.614439724**	**0.618061109**	**0.647717376**	**0.688308085**	**0.724008874**
Armenia	0.551280295	0.57123018	0.59547785	0.589811367	0.605172934	0.659510892	0.716272041	0.755191663
Azerbaijan	0.586694403	0.62084354	0.644641549	0.650206068	0.640320699	0.675706595	0.749257918	0.788300789
Georgia	0.642993327	0.667137702	0.693835638	0.667895279	0.645079498	0.682421144	0.7266782	0.761098405
Kazakhstan	0.631179771	0.652695667	0.681825348	0.703485119	0.715619405	0.743098327	0.773780295	0.806690969
Kyrgyzstan	0.551462396	0.569068764	0.592456223	0.596051157	0.585282665	0.595452862	0.61075204	0.630739954
Mongolia	0.380441623	0.438968576	0.513275644	0.559829252	0.594365545	0.628878324	0.662046074	0.704654999
Tajikistan	0.451086338	0.473947298	0.505370557	0.502211779	0.466174896	0.497254529	0.540743068	0.574056484
Turkmenistan	0.530401284	0.563208678	0.594748489	0.625451936	0.642115132	0.680808325	0.733978061	0.78052685
Uzbekistan	0.481769935	0.511840875	0.54459616	0.566851065	0.590152478	0.620064585	0.658854035	0.699169678
**Central Europe**	**0.642839793**	**0.668235717**	**0.694622756**	**0.714775264**	**0.746447336**	**0.776725368**	**0.806660169**	**0.832664293**
Albania	0.509096373	0.541893243	0.571873464	0.574975879	0.617561434	0.670330931	0.708271367	0.73641317
Bosnia and Herzegovina	0.425901519	0.462876054	0.489123634	0.504125423	0.622612488	0.680542408	0.714392069	0.738710849
Bulgaria	0.635303769	0.669931605	0.701923457	0.718566664	0.730402531	0.753038549	0.78397064	0.807949875
Croatia	0.608400219	0.641005692	0.670180371	0.674532689	0.699493159	0.734672406	0.763702851	0.784160687
Czech Republic	0.726442528	0.760572361	0.785671046	0.812120647	0.838728422	0.856323354	0.875774793	0.8918411
Hungary	0.677125939	0.707578945	0.727444965	0.745804619	0.772879277	0.803137567	0.827830126	0.849101019
Macedonia	0.595574772	0.624413103	0.646489991	0.658176778	0.679164654	0.707288066	0.739257072	0.762163653
Montenegro	0.638204865	0.666023822	0.687017365	0.687731931	0.705608905	0.73611932	0.772722422	0.798900572
Poland	0.663197889	0.678200981	0.70573538	0.735319113	0.779513016	0.809576016	0.836763014	0.867529927
Romania	0.637579174	0.665447358	0.693119173	0.703667723	0.715834859	0.740745953	0.774353411	0.799058233
Serbia	0.603904391	0.627884789	0.653139964	0.658487503	0.674144703	0.713563264	0.750282357	0.771847675
Slovakia	0.649252953	0.682302483	0.71257612	0.738720353	0.772891817	0.803237671	0.835503416	0.86151286
Slovenia	0.678831362	0.719112946	0.752874344	0.772457759	0.800359961	0.827312944	0.846512587	0.856100693
**Eastern Europe**	**0.689161158**	**0.709226745**	**0.740287892**	**0.757962047**	**0.757499928**	**0.783618545**	**0.819220007**	**0.843078172**

Belarus	0.631506757	0.655905732	0.686830251	0.705050366	0.721433405	0.760038051	0.809352043	0.846778088
Estonia	0.658977078	0.679912134	0.712303926	0.734754307	0.763270471	0.797774284	0.831981527	0.860808166
Latvia	0.686746063	0.704005202	0.734755511	0.744670044	0.760347073	0.795892971	0.834290668	0.861362174
Lithuania	0.668298953	0.692211644	0.717504127	0.726005101	0.744894984	0.784288914	0.817348274	0.836858695
Moldova	0.574658471	0.590733167	0.615604469	0.615481674	0.597049611	0.627785133	0.66780569	0.703282651
Russia	0.715022524	0.731526392	0.761315267	0.780473321	0.779314102	0.801975224	0.834255387	0.856366681
Ukraine	0.640325597	0.669113804	0.703762814	0.719192422	0.714342897	0.745302561	0.787137041	0.811050078
**High-income**	**0.738588992**	**0.763398405**	**0.788702664**	**0.812820888**	**0.836116141**	**0.854272563**	**0.8720112**	**0.888391165**
**High-income Asia Pacific**	**0.711899448**	**0.744171035**	**0.780235385**	**0.813752522**	**0.841312036**	**0.861267599**	**0.875926108**	**0.88873281**
Brunei	0.717815348	0.746921814	0.783919893	0.823336967	0.871353347	0.893447311	0.908870499	0.923193369
Japan	0.749510994	0.774630459	0.805415953	0.835739886	0.857986037	0.873005424	0.884472252	0.895486335
South Korea	0.600778304	0.657729329	0.712724971	0.756161225	0.799008059	0.831660612	0.854493728	0.871332652
Singapore	0.640101916	0.682734482	0.712545956	0.757467893	0.795119569	0.836203949	0.865964713	0.88108014
**Australasia**	**0.754057693**	**0.779862393**	**0.805142191**	**0.827677586**	**0.85424025**	**0.872592488**	**0.889428105**	**0.910318174**
Australia	0.755817339	0.781676049	0.808119886	0.830869816	0.858038372	0.87645725	0.893793034	0.915131633
New Zealand	0.745849482	0.771123675	0.790118741	0.811955295	0.835394485	0.853542995	0.86697017	0.884248621
**Western Europe**	**0.700030903**	**0.729025292**	**0.758840118**	**0.786099143**	**0.811082716**	**0.832337606**	**0.850921541**	**0.867072666**
Andorra	0.774028975	0.801501468	0.823988797	0.843840532	0.862037177	0.880389963	0.897223852	0.918626166
Austria	0.740861541	0.765507419	0.787938074	0.810242478	0.833852954	0.855462409	0.874152858	0.888263708
Belgium	0.729441367	0.755556511	0.779707933	0.802996952	0.827072238	0.8456681	0.864137914	0.881525114
Cyprus	0.642458005	0.684624815	0.724550386	0.759622243	0.790777897	0.82591572	0.864121952	0.880491027
Denmark	0.774718447	0.802069759	0.821055146	0.839763667	0.864272398	0.880904696	0.897210551	0.909623976
Finland	0.727835231	0.757783892	0.785111559	0.802502398	0.830373857	0.854128206	0.876019581	0.892851939
France	0.688435979	0.711362407	0.735882509	0.756246712	0.77619835	0.795909928	0.81519636	0.834332875
Germany	0.726469153	0.757465426	0.791905691	0.824040372	0.847218089	0.868908179	0.887447597	0.902577139
Greece	0.638633441	0.674854939	0.708875189	0.737751809	0.766689545	0.792032049	0.81678203	0.824603165
Iceland	0.722376125	0.758337574	0.786644208	0.806779119	0.835635916	0.856528749	0.878618767	0.893330823
Ireland	0.641800194	0.690806918	0.735012055	0.771696924	0.809051333	0.841363673	0.863118508	0.885177302
Israel	0.708566486	0.731152027	0.750875848	0.775782508	0.798539126	0.814650254	0.828432228	0.842446023
Italy	0.712874088	0.74479936	0.770338081	0.79034828	0.809487242	0.825826901	0.842441116	0.855924138
Luxembourg	0.731223195	0.759887435	0.793161955	0.823849448	0.854695263	0.873576079	0.895527692	0.911109808
Malta	0.611019686	0.643491889	0.665237885	0.686422899	0.728288739	0.761091012	0.784765241	0.806204149
Netherlands	0.743403385	0.765334727	0.788175783	0.812687316	0.836646875	0.857203896	0.878180451	0.893628387
Norway	0.771239029	0.801533775	0.824762935	0.849809204	0.881190388	0.902089107	0.921560548	0.936504033
Portugal	0.513265957	0.552241686	0.591327055	0.630160053	0.665670902	0.698341666	0.727712507	0.752221525
Spain	0.581560349	0.62224933	0.662031433	0.700663089	0.733827192	0.76532698	0.795014033	0.819226743
Sweden	0.742238098	0.763222762	0.783168757	0.812871505	0.844474614	0.861064766	0.87655592	0.89208762
Switzerland	0.777928951	0.803687643	0.831431579	0.85632259	0.877441403	0.896568804	0.913826973	0.928205165
United Kingdom	0.736427014	0.760857965	0.790861574	0.818015928	0.847926445	0.870097309	0.881367513	0.89295174
**Southern Latin America**	**0.578844811**	**0.602293473**	**0.626104041**	**0.66194293**	**0.697866904**	**0.719055767**	**0.749732342**	**0.779666499**
Argentina	0.576809418	0.600561047	0.623366885	0.657617462	0.693368234	0.711717578	0.742169654	0.772020634
Chile	0.587190325	0.61001572	0.637072239	0.678597426	0.716369249	0.745259023	0.776219178	0.804796209
Uruguay	0.566195667	0.588685934	0.608551725	0.635489443	0.663570725	0.678616277	0.70864353	0.744987004
**High-income North America**	**0.837699729**	**0.850794766**	**0.862313625**	**0.873125848**	**0.888082201**	**0.900466193**	**0.91619106**	**0.9316971**
Canada	0.814370128	0.837911395	0.858142963	0.873267278	0.894857096	0.910704781	0.922647109	0.937512608
Greenland	0.578794516	0.60788725	0.605870563	0.620714949	0.662006522	0.672906496	0.702960518	0.757535445
United States	0.840275467	0.852248349	0.862831247	0.873163138	0.887390727	0.899393149	0.915517874	0.931072387
**Latin America and Caribbean**	**0.447476775**	**0.488809133**	**0.525578925**	**0.55781664**	**0.588531592**	**0.618039692**	**0.649808648**	**0.678081425**
**Caribbean**	**0.503323694**	**0.532214082**	**0.558018778**	**0.574502604**	**0.59507038**	**0.618594375**	**0.643425098**	**0.665511627**
Antigua and Barbuda	0.65741856	0.693466835	0.737397221	0.761579323	0.784172149	0.803935234	0.829663879	0.841039055
The Bahamas	0.649143836	0.685987871	0.720512387	0.742588863	0.780457869	0.811609476	0.823587612	0.834779019
Barbados	0.617770597	0.648208246	0.682913276	0.704370691	0.728768495	0.75209851	0.770844772	0.782399831
Belize	0.374255239	0.426555459	0.474487714	0.515257205	0.562584618	0.606801215	0.638520926	0.665157834
Bermuda	0.777975496	0.811198033	0.839302353	0.858987929	0.874021317	0.893341075	0.911351512	0.916446969
Cuba	0.597603116	0.635791682	0.664721645	0.667684523	0.677056878	0.704947558	0.739839338	0.766197728
Dominica	0.521972834	0.562912615	0.599221864	0.637191195	0.678421963	0.703861202	0.730039302	0.752622949
Dominican Republic	0.434398821	0.475603327	0.509555619	0.541836617	0.580264345	0.612992998	0.649486542	0.683679531
Grenada	0.476069099	0.524212872	0.54684398	0.613566364	0.661753633	0.703475946	0.732257212	0.753182169
Guyana	0.4605055	0.476211921	0.491668463	0.523899605	0.563961459	0.588476288	0.616473397	0.654552983
Haiti	0.25448157	0.276575111	0.303542337	0.321791232	0.344406896	0.366625611	0.387114034	0.411767867
Jamaica	0.530098181	0.556460971	0.588780541	0.623987031	0.65041242	0.674373131	0.699769019	0.718939443

Puerto Rico	0.705228933	0.737981664	0.768406195	0.797805783	0.826504432	0.85280005	0.869553331	0.882005119
Saint Lucia	0.480247463	0.528368517	0.573569544	0.619925813	0.664185403	0.696232586	0.718759552	0.74081607
Saint Vincent and the Grenadines	0.491753214	0.542977025	0.585478653	0.626361524	0.663078017	0.69789826	0.727934753	0.747273962
Suriname	0.487557158	0.518614951	0.547574445	0.571047119	0.599640851	0.633450595	0.670317551	0.703710724
Trinidad and Tobago	0.629324743	0.659571249	0.681253145	0.70534663	0.738480246	0.777462364	0.8114744	0.83274793
Virgin Islands, U.S.	0.696995761	0.733621555	0.762543921	0.808821021	0.844069858	0.8606661	0.873456751	0.886104177
**Andean Latin America**	**0.451301893**	**0.489868147**	**0.519708662**	**0.548248337**	**0.581544889**	**0.611914133**	**0.647666806**	**0.682353013**
Bolivia	0.376389158	0.405022335	0.430019104	0.464094758	0.502821269	0.537694329	0.574646333	0.611997688
Ecuador	0.462734025	0.503402971	0.534664945	0.566242743	0.594424276	0.622743126	0.654359927	0.68518032
Peru	0.470208229	0.510590583	0.54097985	0.566273475	0.600675411	0.631136547	0.668983198	0.705027187
**Central Latin America**	**0.457521118**	**0.502904192**	**0.53866564**	**0.573768779**	**0.605966961**	**0.634874888**	**0.666203428**	**0.693760401**
Colombia	0.468059434	0.508271648	0.544220251	0.579101394	0.608828145	0.633870264	0.667314133	0.699779414
Costa Rica	0.515586527	0.536362197	0.558721796	0.590436451	0.628851915	0.660784335	0.692996816	0.72316093
El Salvador	0.375799747	0.404952348	0.435266539	0.475920447	0.518598127	0.557152113	0.591037069	0.618650578
Guatemala	0.311748063	0.339356506	0.36801856	0.399334294	0.436599272	0.474475275	0.510383004	0.542708882
Honduras	0.309169999	0.346793995	0.37997563	0.414018673	0.451095645	0.492077179	0.534950312	0.568371081
Mexico	0.466145488	0.522288268	0.562592619	0.599924021	0.633450175	0.663827808	0.692382437	0.71760429
Nicaragua	0.344975419	0.372590781	0.399300688	0.426462303	0.464911946	0.500725091	0.531501824	0.56306356
Panama	0.529321908	0.567995833	0.594016406	0.619798058	0.6490404	0.675653311	0.70838767	0.746691153
Venezuela	0.532718005	0.563368997	0.590931589	0.622371572	0.647153588	0.668767248	0.703511462	0.728268209
**Tropical Latin America**	**0.422256248**	**0.463341708**	**0.505530966**	**0.538889349**	**0.569358422**	**0.600543122**	**0.632831711**	**0.661067663**
Brazil	0.422478243	0.46379689	0.506306633	0.539729153	0.57003497	0.601293864	0.633617238	0.661625395
Paraguay	0.413729051	0.446401184	0.477903688	0.510241626	0.54695522	0.576219533	0.607700585	0.643643256
**North Africa and Middle East**	**0.329976137**	**0.37365675**	**0.422538485**	**0.468865645**	**0.512122867**	**0.547907694**	**0.58033147**	**0.604459365**
Afghanistan	0.129097125	0.139184967	0.144020502	0.15257563	0.141621199	0.18120623	0.230305062	0.288772923
Algeria	0.31731857	0.382320732	0.443435599	0.504232992	0.54743501	0.571352796	0.57836111	0.59002177
Bahrain	0.505853207	0.549778051	0.596915733	0.650251029	0.68486968	0.718659236	0.762090477	0.776379065
Egypt	0.346982308	0.386459893	0.440895429	0.497029462	0.543893458	0.578751733	0.60124628	0.619114009
Iran	0.345590224	0.381378233	0.460032347	0.546104445	0.613362941	0.663578153	0.697666182	0.715386016
Iraq	0.32335601	0.36643763	0.399672242	0.428189951	0.480013024	0.517205975	0.537583652	0.575584738
Jordan	0.362487785	0.453074219	0.496674824	0.56574793	0.595154395	0.630249745	0.666915735	0.694911803
Kuwait	0.526398404	0.618117358	0.691112875	0.721584481	0.765211425	0.78900208	0.835891067	0.862382439
Lebanon	0.501236275	0.539436038	0.569766554	0.602629767	0.642284629	0.687003256	0.720203276	0.754653364
Libya	0.358368559	0.418231142	0.474741497	0.535822407	0.575576847	0.606770693	0.641317064	0.643012703
Morocco	0.252172814	0.291625704	0.334737671	0.374314244	0.412300926	0.44110989	0.467744738	0.495876327
Palestine	0.352812737	0.395866631	0.422927028	0.471348134	0.541018634	0.551197342	0.547132348	0.566998222
Oman	0.290477326	0.330562039	0.408928197	0.520051253	0.609809866	0.658300566	0.686291539	0.730058282
Qatar	0.496255694	0.563028464	0.616220711	0.65706465	0.684955564	0.732995474	0.776040758	0.804539365
Saudi Arabia	0.400474563	0.483200887	0.52453299	0.572616308	0.643252404	0.690103832	0.72990531	0.759303147
Sudan	0.231808703	0.251941243	0.26670437	0.288873176	0.318846736	0.358223321	0.39705192	0.42821368
Syria	0.294632684	0.341028589	0.388067472	0.4393674	0.482972442	0.525331599	0.580746752	0.579032245
Tunisia	0.340764224	0.393558629	0.450344495	0.513964496	0.564168642	0.600701033	0.629774716	0.651545393
Turkey	0.441562297	0.481111704	0.524217395	0.559389705	0.593500492	0.62471277	0.658090789	0.689996824
United Arab Emirates	0.534337721	0.587431893	0.63244933	0.727256353	0.78127272	0.823660797	0.869111705	0.874695199
Yemen	0.09604876	0.108224419	0.132943463	0.196399459	0.259493389	0.319643404	0.376581349	0.407961565
**South Asia**	**0.242146983**	**0.270360926**	**0.309553574**	**0.351932111**	**0.396311291**	**0.440658117**	**0.490680254**	**0.536162**
Bangladesh	0.198977949	0.234874084	0.274556028	0.313892619	0.351175369	0.38888892	0.430760718	0.471641721
Bhutan	0.203738284	0.229630101	0.281787327	0.326559365	0.386064922	0.440539737	0.49011301	0.532098888
India	0.251828188	0.279549794	0.319511736	0.362493992	0.408063843	0.454449607	0.507993264	0.556440237
Nepal	0.173805381	0.195203958	0.22377535	0.26219145	0.303603035	0.3395773	0.38056885	0.422731927
Pakistan	0.21389107	0.242139404	0.278605267	0.321797089	0.36563843	0.402940006	0.436316196	0.467583861
**Sub-Saharan Africa**	**0.23272326**	**0.249015188**	**0.266590519**	**0.280985561**	**0.296583374**	**0.317955066**	**0.353432557**	**0.391367307**
**Central Sub-Saharan Africa**	**0.232364659**	**0.241034246**	**0.24677737**	**0.242261403**	**0.221728364**	**0.203903321**	**0.24664615**	**0.300694995**
Angola	0.226560432	0.241467555	0.255344421	0.274645118	0.293470888	0.323362796	0.37318616	0.419097877
Central African Republic	0.191651074	0.205336962	0.21774251	0.231060649	0.243686976	0.259031128	0.282406441	0.281651944
Congo	0.324638511	0.369887186	0.404230153	0.42828619	0.447269725	0.467886713	0.49827706	0.526539538
Democratic Republic of the Congo	0.226094036	0.229081584	0.229476667	0.213641707	0.172380426	0.132408839	0.175648604	0.238770966
Equatorial Guinea	0.226961729	0.27375784	0.261089796	0.285958777	0.423392115	0.522242635	0.5743007	0.608570132
Gabon	0.425723688	0.445740141	0.471508277	0.508041147	0.546069512	0.577580615	0.607891749	0.643545692
**Eastern Sub-Saharan Africa**	**0.168161721**	**0.182926343**	**0.204094564**	**0.218125108**	**0.239228924**	**0.266034499**	**0.307846702**	**0.351505921**
Burundi	0.137057044	0.148485384	0.164878888	0.176181954	0.172776977	0.189692573	0.212910415	0.239484683

Comoros	0.204073084	0.223695409	0.252241152	0.278197473	0.300661804	0.325412011	0.345529141	0.364960388
Djibouti	0.297013334	0.29453185	0.322816616	0.343468503	0.3709505	0.395054724	0.430112437	0.461481004
Eritrea	0.14613198	0.159550507	0.182503293	0.220177204	0.27861977	0.305889171	0.308372075	0.323814306
Ethiopia	0.107762266	0.119746447	0.130930226	0.131333963	0.14670968	0.177379989	0.238002932	0.302164728
Kenya	0.22951478	0.280137945	0.337829643	0.38201419	0.398905696	0.411987898	0.437537058	0.471567253
Madagascar	0.23528442	0.252429647	0.261406741	0.274678602	0.296138905	0.322520811	0.348913862	0.369829599
Malawi	0.156751579	0.159358398	0.193788921	0.195057692	0.221755434	0.233088774	0.27032751	0.308579014
Mozambique	0.08857909	0.072896123	0.062225253	0.072741023	0.131000608	0.185976705	0.233358721	0.278020785
Rwanda	0.133896362	0.148856527	0.204950714	0.211183327	0.234729766	0.266436132	0.320436966	0.371318929
Somalia	0.109816984	0.104963834	0.115763893	0.10588294	0.103448011	0.110814297	0.131492791	0.150584034
South Sudan	0.11166708	0.120199159	0.143533549	0.154119248	0.183011634	0.206579753	0.23523972	0.261565432
Tanzania	0.246258303	0.260932732	0.275665487	0.293933434	0.310620158	0.336401845	0.371850387	0.411379875
Uganda	0.196257159	0.19624051	0.204900817	0.225255978	0.256587243	0.29200731	0.336080541	0.376838656
Zambia	0.281118556	0.305283315	0.325680567	0.336821761	0.351125681	0.374252978	0.420395357	0.46700068
**Southern Sub-Saharan Africa**	**0.478391371**	**0.51055477**	**0.540112462**	**0.561064104**	**0.586779359**	**0.613774576**	**0.63984072**	**0.668821611**
Botswana	0.293065199	0.34916673	0.420230912	0.485403595	0.534572278	0.577447212	0.610733763	0.641123102
Lesotho	0.280816408	0.304677369	0.328905897	0.365725678	0.401387619	0.43994486	0.480280813	0.521663412
Namibia	0.357417011	0.388663086	0.440826005	0.477193796	0.519019378	0.552596178	0.583745509	0.616970444
South Africa	0.544584871	0.572359578	0.593672574	0.604009845	0.624548173	0.654457405	0.688095986	0.716438318
Swaziland	0.321133181	0.351180223	0.41585485	0.469342274	0.52095924	0.545483102	0.585699532	0.623241585
Zimbabwe	0.306430981	0.36568514	0.424467548	0.468103846	0.503096094	0.509122538	0.503585332	0.538428441
**Western Sub-Saharan Africa**	**0.226500458**	**0.24231258**	**0.258099092**	**0.277679383**	**0.300328197**	**0.331559913**	**0.366846058**	**0.400933664**
Benin	0.163467384	0.181999667	0.198386195	0.22229047	0.248757189	0.283463689	0.314616909	0.344606602
Burkina Faso	0.07194045	0.073162734	0.089676396	0.111261544	0.13855732	0.169070234	0.202264159	0.237391581
Cameroon	0.264642374	0.293304279	0.322657035	0.343524349	0.369990484	0.400489295	0.43107967	0.464085129
Cape Verde	0.225582613	0.273818615	0.305640101	0.35316575	0.411979022	0.471457157	0.517603023	0.548473562
Chad	0.123523705	0.130200768	0.144665396	0.157621206	0.169565669	0.203741756	0.247774586	0.287140883
Cote d'Ivoire	0.207479861	0.236124864	0.26019882	0.284439199	0.31259033	0.331652298	0.35189144	0.380703844
The Gambia	0.201504463	0.21510983	0.238799617	0.246920943	0.267247831	0.28775029	0.306394265	0.326635138
Ghana	0.301829109	0.322537197	0.348572758	0.382941183	0.411139132	0.43926045	0.471038584	0.510965987
Guinea	0.142515342	0.157807473	0.170019356	0.194088078	0.212051109	0.231307223	0.253941347	0.277741008
Guinea-Bissau	0.147800265	0.156273378	0.174410192	0.201263223	0.226712287	0.246882358	0.269455555	0.294312883
Liberia	0.192466646	0.207251806	0.215205717	0.168195809	0.17641609	0.215972219	0.241453427	0.28267157
Mali	0.076786283	0.096540254	0.112205211	0.136452523	0.158526143	0.180965478	0.206386276	0.230865277
Mauritania	0.227911115	0.247572438	0.265409597	0.286402135	0.309008633	0.333526545	0.367283169	0.401382721
Niger	0.080409847	0.078164137	0.084561115	0.094295691	0.102632014	0.114084028	0.126975786	0.146486948
Nigeria	0.279256461	0.292785644	0.303594078	0.322055721	0.346724312	0.386685272	0.433597696	0.473959553
Sao Tome and Principe	0.262235741	0.28485838	0.302780101	0.325198007	0.34955925	0.379897116	0.415336625	0.448053868
Senegal	0.159992842	0.182111156	0.20991933	0.237234554	0.262898092	0.290232121	0.311440117	0.334101011
Sierra Leone	0.145893686	0.158653895	0.179612711	0.195177212	0.201764998	0.237460607	0.275694403	0.323014067
Togo	0.202796809	0.222754105	0.244724016	0.267282636	0.292213014	0.309313471	0.330650186	0.361684605

Appendix Table 8. HIV Comparison of GBD, Optima, and AEM										
			GBD			Optima			AEM	
Country	Year	Incidence	Prevalence	Death	Incidence	Prevalence	Death	Incidence	Prevalence	Death
Thailand	1990	55,228	117,032	872	--	--	--	150,015	315,391	1,183
Thailand	1995	25,613	287,452	8,820	--	--	--	55,452	719,772	34,787
Thailand	2000	11,915	298,667	20,598	~30000	--	~60000	26,158	642,121	54,414
Thailand	2005	19,024	297,018	13,594	~22000	--	~51000	16,513	562,243	18,843
Thailand	2010	14,249	307,661	13,107	~15000	--	~30000	--	--	--
Indonesia	2000	8,797	29,873	563	~37500	--	~22500	--	--	--
Indonesia	2005	33,526	146,556	3,321	~38000	--	~22000	--	--	--
Indonesia	2010	42,387	311,005	9,647	~41000	--	~27000	--	--	--
Zambia	2000	90,061	873,270	55,509	~95000	1,494,000	~62000	--	--	--
Zambia	2005	97,047	983,957	59,259	~10100	1,573,000	~70000	--	--	--
Zambia	2010	75,540	1,143,741	37,962	~92000	1,529,000	~48000	--	--	--
Cambodia	1990	2,684	11,946	336	--	--	--	2,895	4,087	--
Cambodia	1995	3,839	25,646	938	--	--	--	38,822	144,989	--
Cambodia	2000	5,375	43,193	1,932	--	--	--	10,553	188,975	--
Cambodia	2002	4,742	49,222	1,531	--	--	--	7,300	164,000	21,200
Uzbekistan	2012	368	12,793	776	3,300	37,712	--	--	--	--
Niger	2000	10,482	84,264	4,048	~7500	106,400	~11500	--	--	--
Niger	2005	5,874	91,267	6,422	~5800	60,750	~6500	--	--	--
Vietnam	2010	14,475	167,744	6,075	127,600	539,240	102,944	--	--	--

Appendix Figure 1. HIV Prevalence by country, 2015

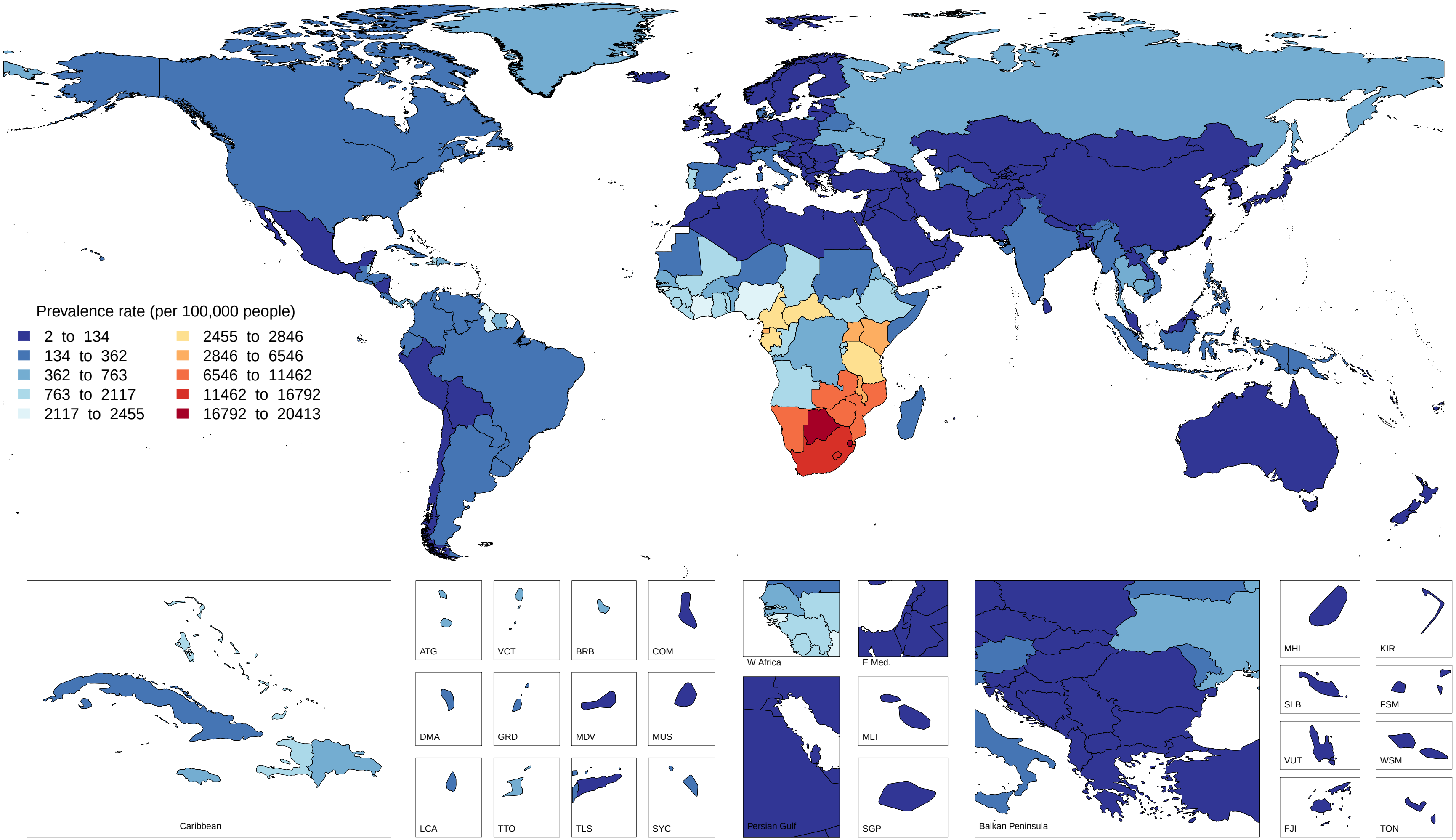

Appendix Figure 2. Difference between 81% ART coverage and ART coverage by country, 2015

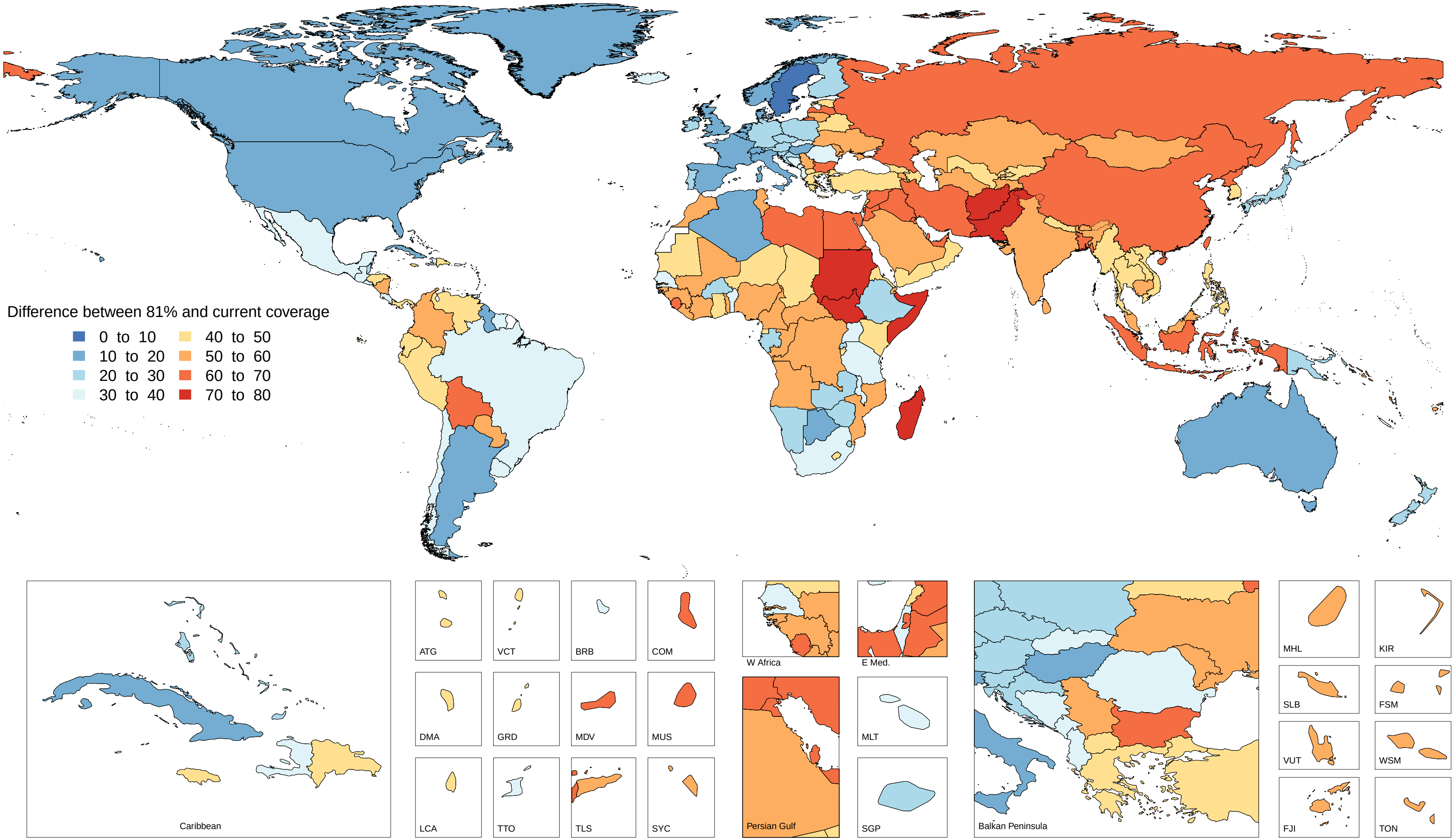

Appendix Figure 3. Global number of deaths due to HIV split by region with global uncertainty intervals from 1980 to 2015

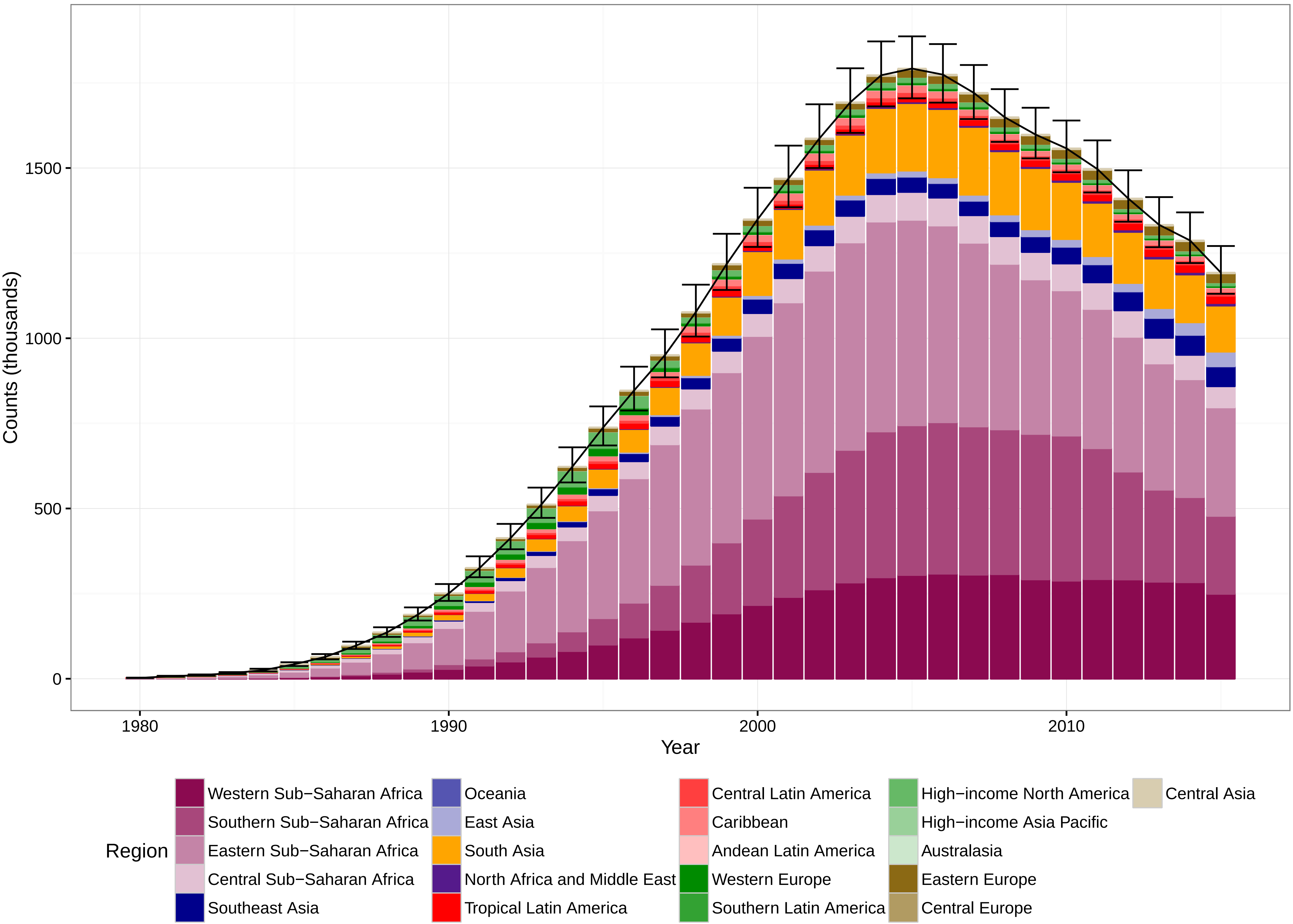

Appendix Figure 4. Global number of people living with HIV split by region with global uncertainty intervals from 1980 to 2015

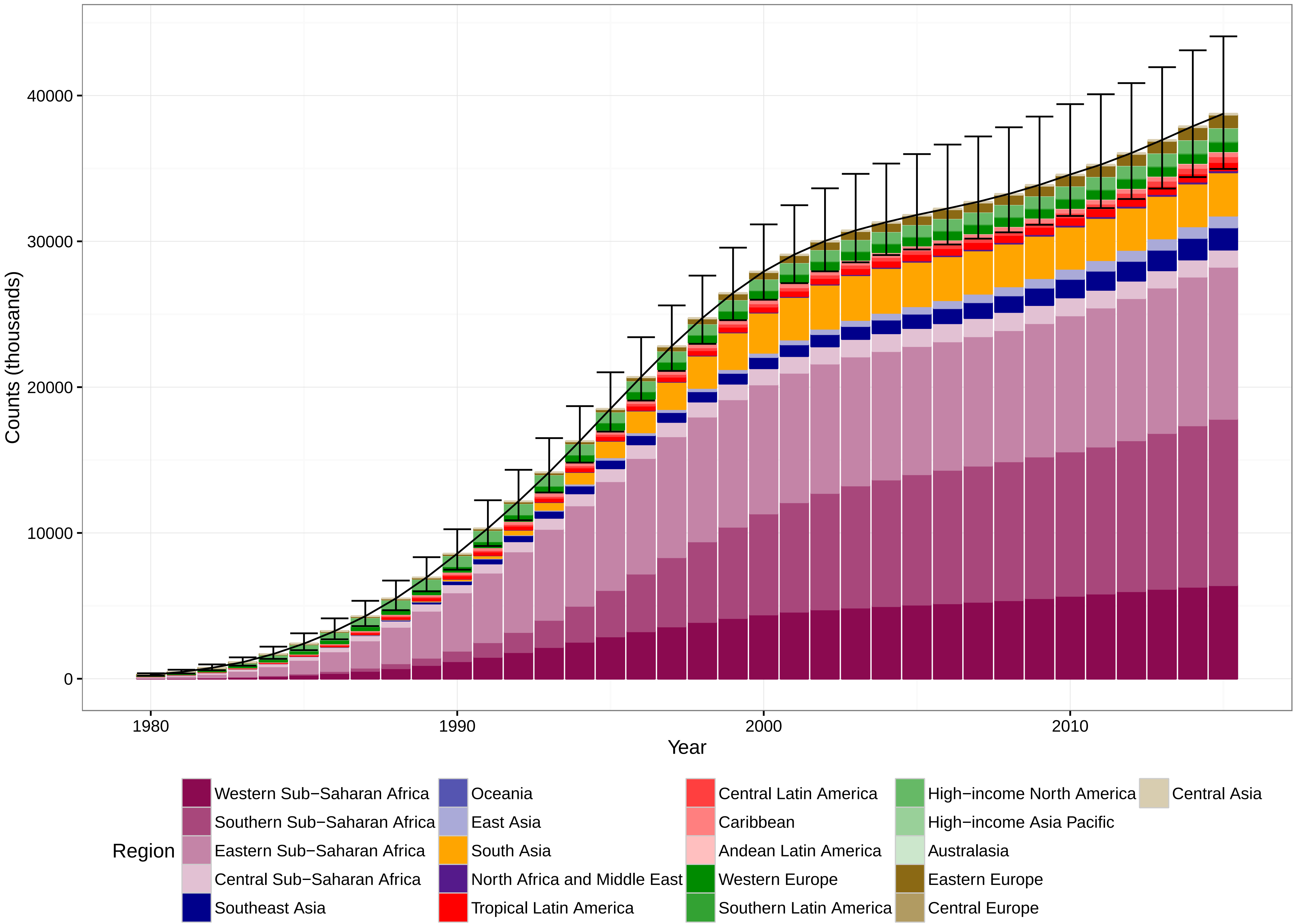

Appendix Figure 5. Comparing GBD and UNAIDS estimates of deaths due to HIV in Sub-Saharan Africa in 2014

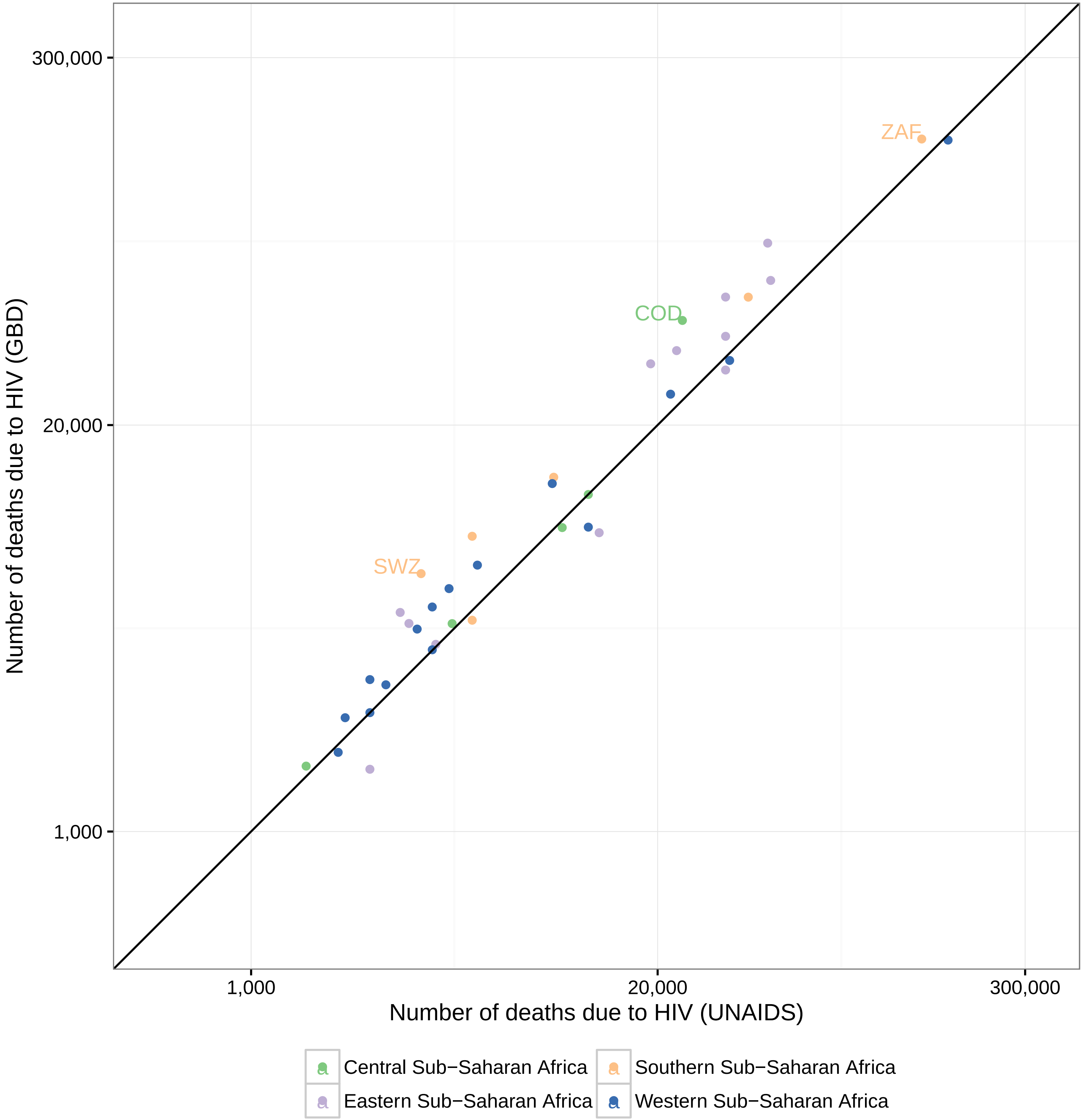

Appendix Figure 6. Comparing GBD and UNAIDS estimates of adult HIV prevalence rate in Sub-Saharan Africa in 2014

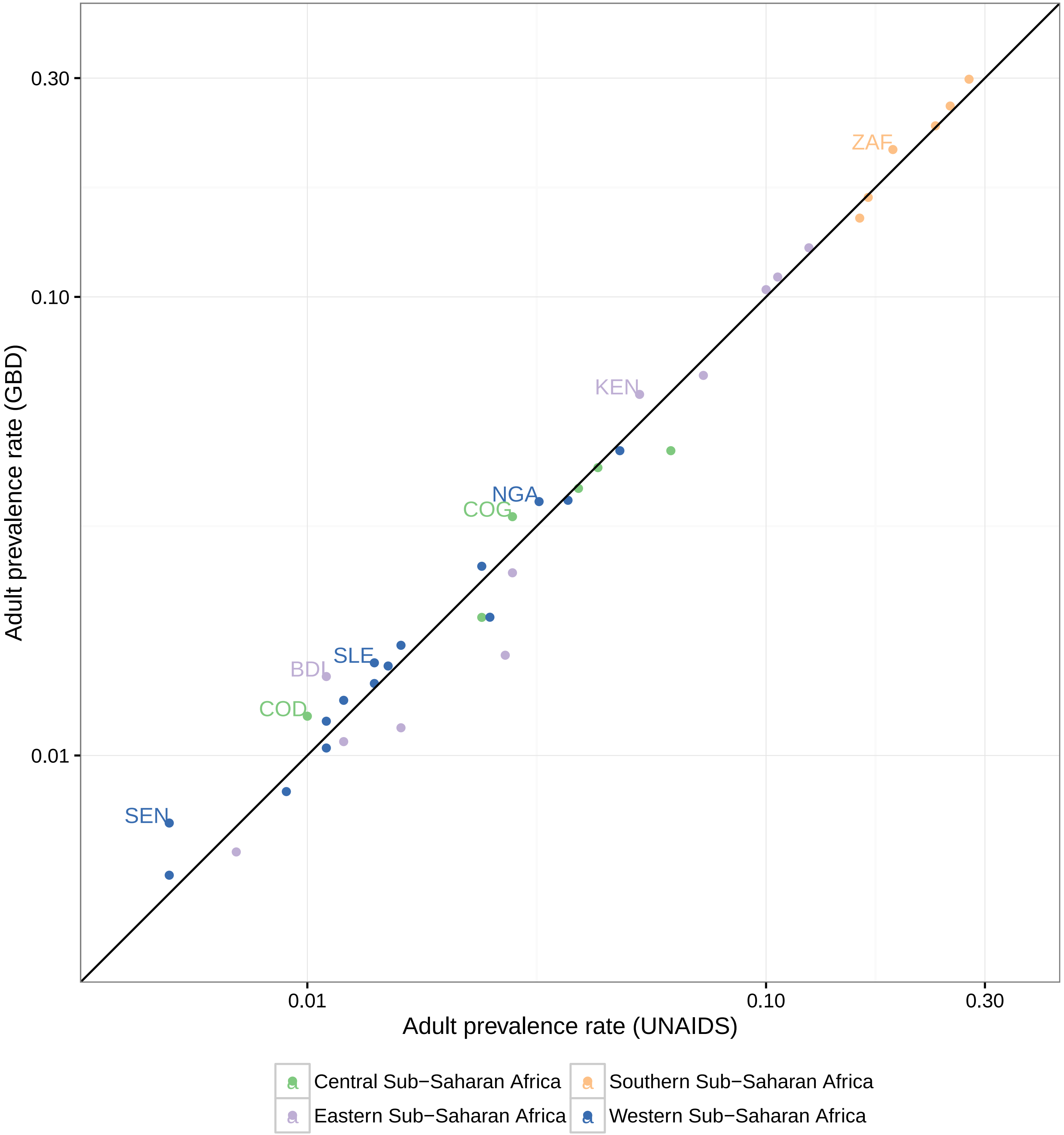

Appendix Figure 7. HIV mortality rate by country, 2015

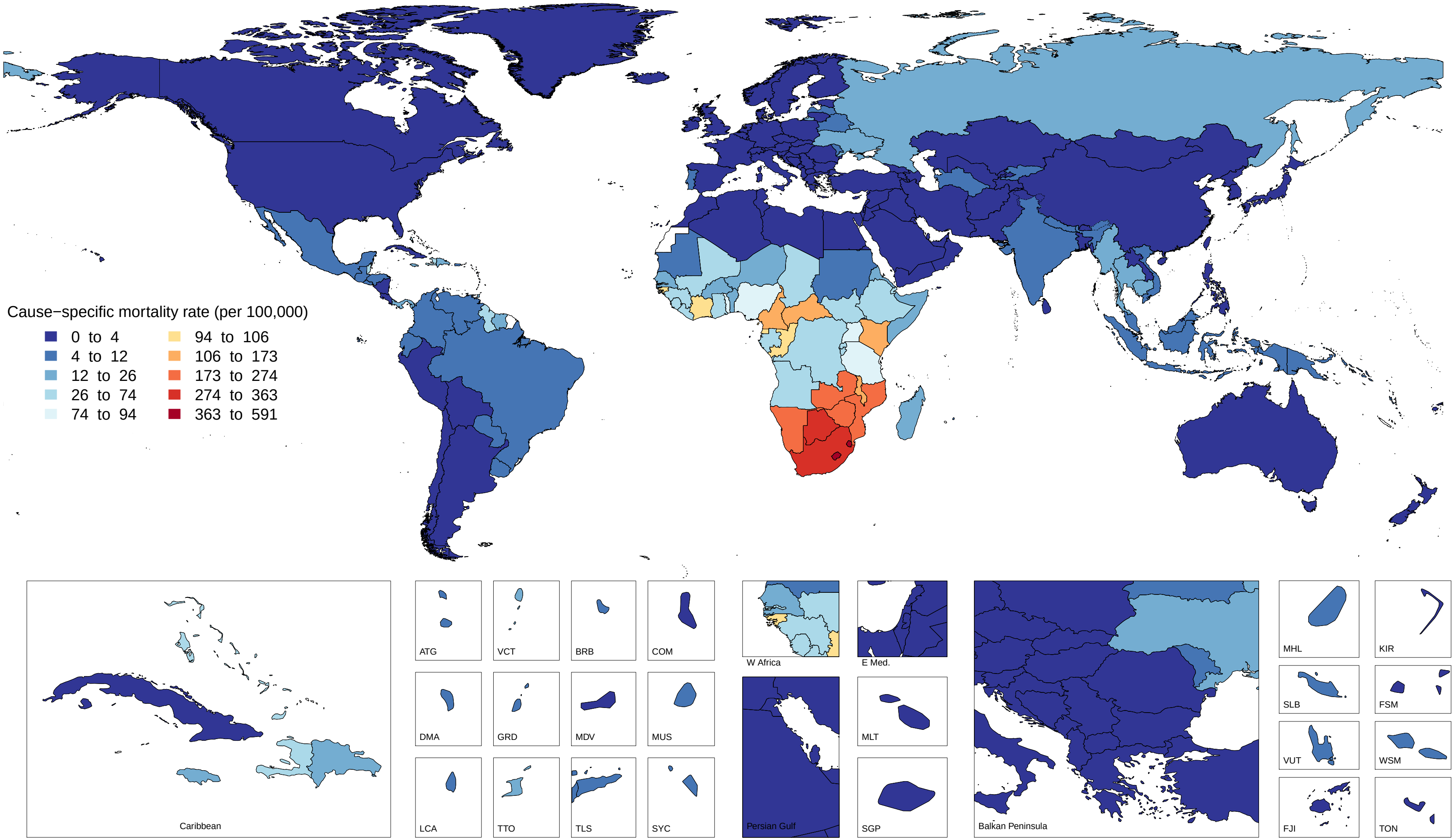

Appendix Figure 8. HIV incidence rate by country, 1995

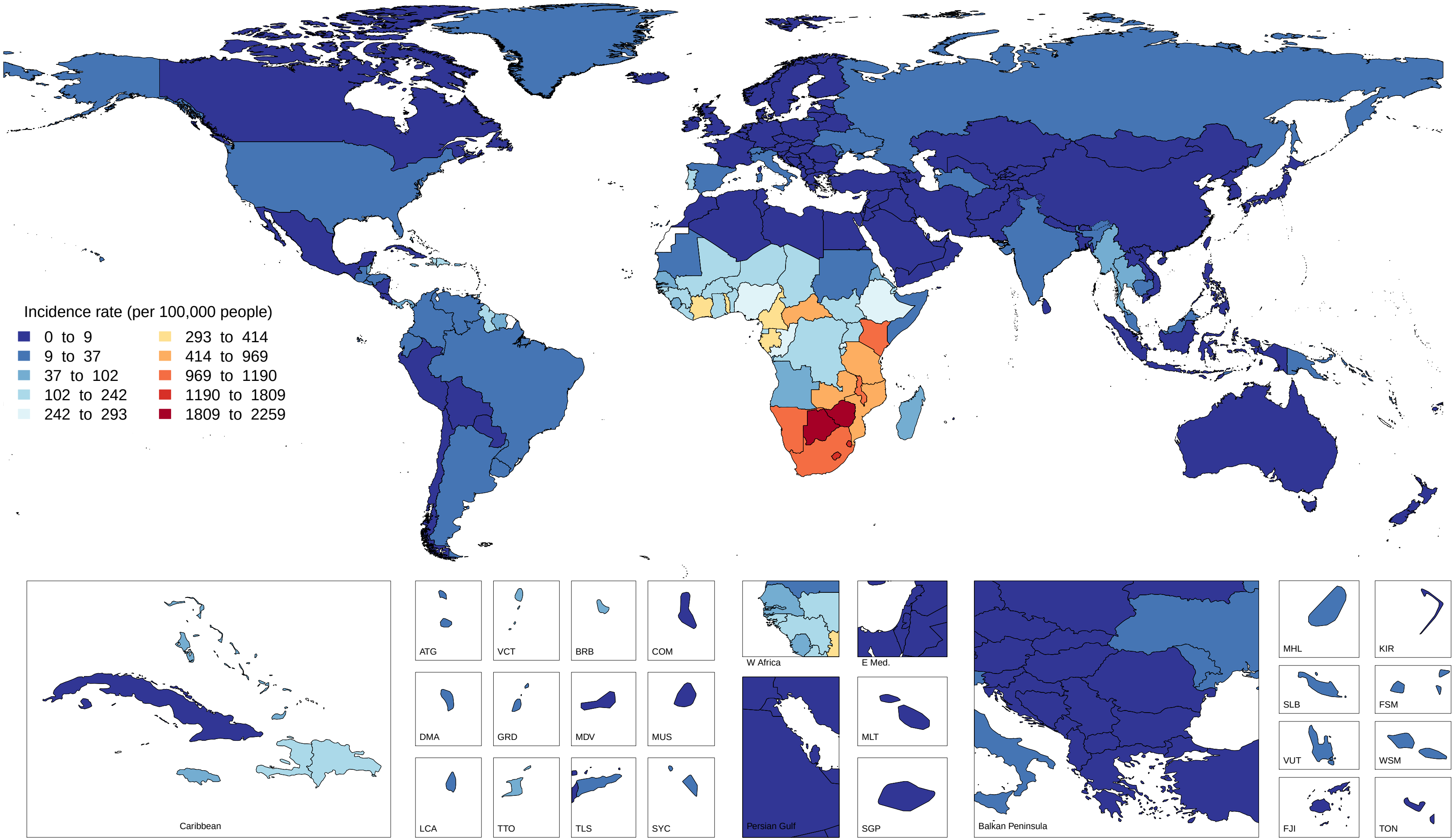

Appendix Figure 9. HIV incidence rate by country, 2005

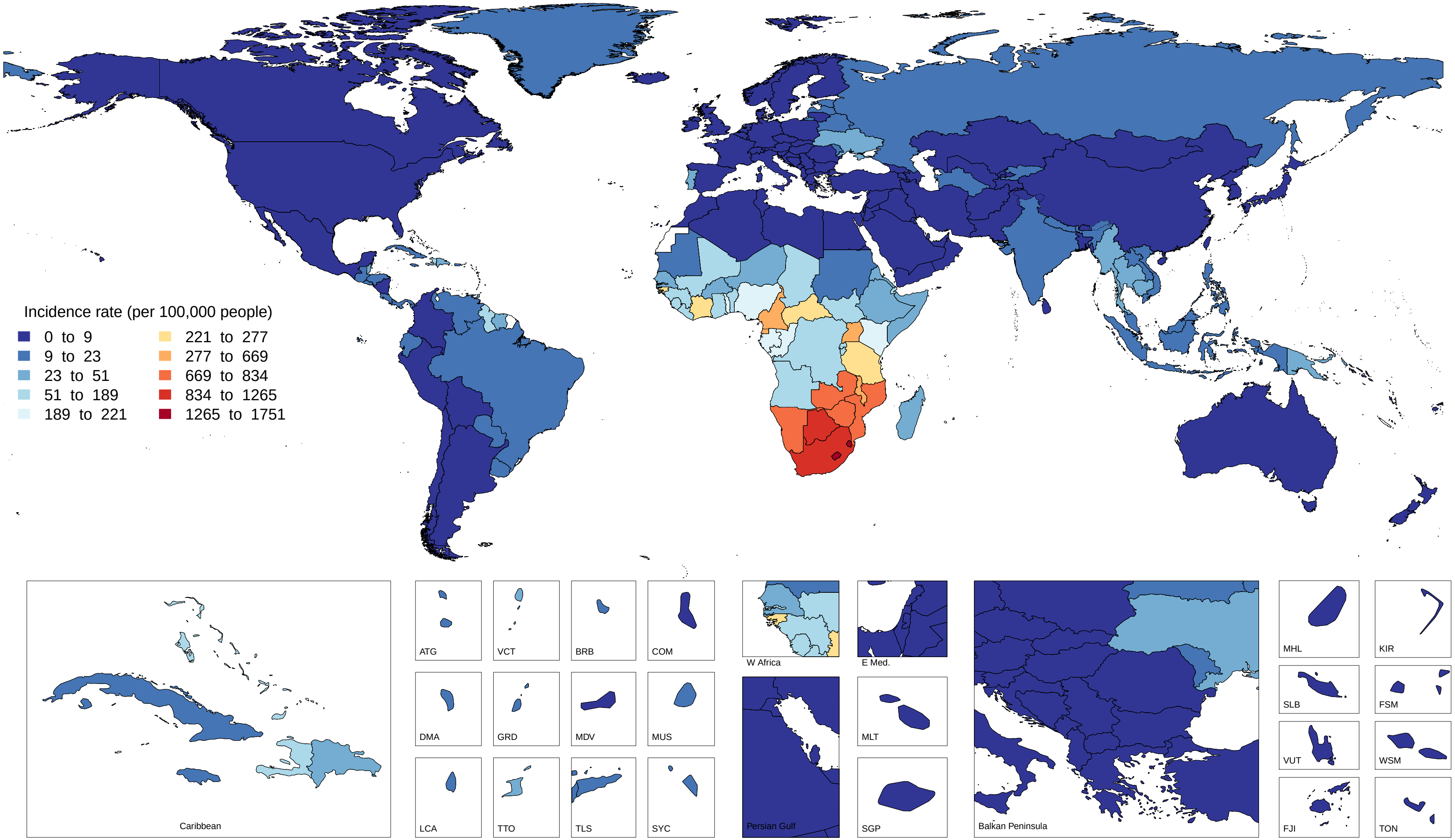

Supplement: Supplementary appendix [file mmc1.pdf]
